# Glycoconjugation of Quinoline Derivatives Using the C-6 Position in Sugars as a Strategy for Improving the Selectivity and Cytotoxicity of Functionalized Compounds

**DOI:** 10.3390/molecules27206918

**Published:** 2022-10-15

**Authors:** Monika Domińska, Gabriela Pastuch-Gawołek, Magdalena Skonieczna, Wiesław Szeja, Adrian Domiński, Piotr Kurcok

**Affiliations:** 1 Department of Organic Chemistry, Bioorganic Chemistry and Biotechnology, Silesian University of Technology, B. Krzywoustego 4, 44-100 Gliwice, Poland; 2 Biotechnology Centre, Silesian University of Technology, B. Krzywoustego 8, 44-100 Gliwice, Poland; 3 Department of Systems Biology and Engineering, Faculty of Automatic Control, Electronics and Computer Science, Silesian University of Technology, Akademicka 16, 44-100 Gliwice, Poland; 4 Centre of Polymer and Carbon Materials, Polish Academy of Sciences, M. Curie-Skłodowskiej 34, 41-819 Zabrze, Poland

**Keywords:** quinoline glycoconjugates, 1,2,3-triazole, click chemistry, anticancer activity, drug targeting, GLUT transporter, Warburg effect

## Abstract

Based on the Warburg effect and the increased demand for glucose by tumor cells, a targeted drug delivery strategy was developed. A series of new glycoconjugates with increased ability to interact with GLUT transporters, responsible for the transport of sugars to cancer cells, were synthesized. Glycoconjugation was performed using the C-6 position in the sugar unit, as the least involved in the formation of hydrogen bonds with various aminoacids residues of the transporter. The carbohydrate moiety was connected with the 8-hydroxyquinoline scaffold via a 1,2,3-triazole linker. For the obtained compounds, several in vitro biological tests were performed using HCT-116 and MCF-7 cancer cells as well as NHDF-Neo healthy cells. The highest cytotoxicity of both cancer cell lines in the MTT test was noted for glycoconjugates in which the triazole-quinoline was attached through the triazole nitrogen atom to the d-glucose unit directly to the carbon at the C-6 position. These compounds were more selective than the analogous glycoconjugates formed by the C-1 anomeric position of d-glucose. Experiments with an EDG inhibitor have shown that GLUTs can be involved in the transport of glycoconjugates. The results of apoptosis and cell cycle analyses by flow cytometry confirmed that the new type of glycoconjugates shows pro-apoptotic properties, without significantly affecting changes in the distribution of the cell cycle. Moreover, glycoconjugates were able to decrease the clonogenic potential of cancer cells, inhibit the migration capacity of cells and intercalate with DNA.

## 1. Introduction

Efficacy, as well as safety, are the main criteria influencing the approval of any new drug by regulatory agencies. The latest medicine aims for treatment that is precise and personalized with the needs of each patient, in which drugs will be delivered specifically to diseased cells, but not healthy ones. One of the attractive strategies to increase the efficacy and safety of the treatment is the use of a proven therapeutic agent with the addition of a targeting ligand, which should exhibit low affinity for healthy cells, but high for pathological cells. The efficacy of the formed conjugate is primarily determined by the activity of the drug, while the safety of the conjugate is dependent on the specificity of the targeting ligand to diseased cells. By separating the selectivity and therapeutic activity into two separate parts of the conjugate, it is possible to optimize drug action by avoiding side damage to healthy tissues [1,2,3,4,5]. The safety profile of a targeted drug is mainly dependent on the expression of a given receptor, and precisely its ratio in diseased and normal tissues. It has been found that at least 3-fold overexpression of the receptors in pathological tissues is usually sufficient to avoid high toxicity for healthy tissues. Receptors in pathological cells can be overexpressed both intracellularly and on the cell surface [1,6,7,8]. It is preferable, that the ligand targets receptors found mainly on the surface of the diseased cell where they are overexpressed relative to normal cells with low receptor expression, whereas conjugates targeting intracellular receptors must first be well permeable through the cell membrane, which may be associated with low selectivity and systemic toxicity of the drug.

New strategies for the rational design of anticancer drug candidates relate to the improvement of the transport of the active molecule directly into the cancer cell. This is achieved by binding potential drugs to appropriate ligands (sugars, peptides, vitamins, proteins, or antibodies) specific to a given receptor. The ligand-based prodrug then binds to its receptor, which should be present only in the cancer cell or be overexpressed in it. In particular, in recent years, there has been a lot of interest in research involving liposomes, polymer micelles, nanoparticles, dendrimers, monoclonal antibodies, or specialized transmembrane proteins characteristic of the target cells [9,10,11]. In the design of ligand-targeting drugs in cancer therapy, the focus is on differences in the metabolism of healthy cells and cancer cells. An important difference is the carbohydrate metabolism mechanism described by Warburg [12,13]. Compared to healthy cells, rapidly proliferating tumor cells are characterized by a high rate of the glycolysis process. To provide enough nutrients for this process and to meet their energy demand, cancer cells have an increased need for glucose. Glucose, as the basic source of energy, is actively transported inside the cells by specialized proteins, the so-called GLUT transporters [14,15]. Increased glucose consumption requires overexpression of the GLUT transporters in cancer cells as compared to normal cells [16]. This observation has become an attractive strategy for the controlled delivery of prodrugs obtained by coupling biologically active compounds with sugar derivatives. Due to the high affinity of glycoconjugates for GLUT protein, glycoconjugation can be seen as a system for the selective delivery of anti-cancer drugs [17,18,19].

The oldest example of a drug actively transported by GLUT proteins is glufosfamide, discovered in 1995 [20]. Since then, numerous clinical trials have been conducted with glufosfamide in the treatment of various types of cancer. It turned out that the glycoconjugate is safer and more selective than its aglycon, and its antiproliferative activity is similar to the original aglycone. Its mechanism of action favors cancers with overexpression of GLUT transporters [21,22]. Following the success of glufosfamide, the glycoconjugation strategy gained popularity. In targeted cancer therapy using GLUT, various glycoconjugates based on molecules such as oxaliplatin [23], chlorambucil [24], paclitaxel [25], adriamycin [26], azomycin [27], methotrexate [28], as well as derivatives of thiosemicarbazone [29] have been designed. Numerous glycoconjugates showed better cancer specificity, reduced systemic toxicity, and increased anticancer activity both in vitro and in vivo.

GLUT1 is one of the most common glucose transporters in the vicinity of neoplastic cells. Encoded by SLC2A1, it can bind to glucose, galactose, mannose, or ascorbic acid and then transport these molecules across membranes into the cell. It has been shown that increased GLUT1 expression appears to be strongly correlated with poor prognosis in many neoplastic diseases [19,30]. However, the ability of GLUT to transport a substrate may be influenced by factors such as the structure of the carbohydrate, the position of its substitution, the type of linkers, and the properties of the aglycons themselves. Recently, the crystalline structure of human GLUT1 has been published [31]. This structure revealed that all hydroxyl groups of d-glucose, except for this at the C-6 position, are involved in the formation of hydrogen bonds with various residues of aminoacids of the transporter. Therefore, modification at the C-6 position of d-glucose should not affect the binding of the receptor to the transporter. The Fernandez group synthesized several glycosyl derivatives of dopamine and tested the affinity of the resulting prodrugs for GLUT1 in human erythrocytes. Dopamine was connected with C-1, C-3, or C-6 positions of d-glucose using various linkages. The glucose absorption inhibition results showed that derivatives of glucose conjugated at the C-6 position had the best affinity for GLUT1 [32]. It was also found that C-6-glucose conjugates of 4-nitrobenzofurazane, ketoprofen, and indomethacin connect to GLUT1 with even better affinity than unmodified d-glucose. Patra et al. designed glucose-platinum conjugates in which the chelator was connected via the C-6 position of the sugar. They found that the obtained glycoconjugates show a high level of cytotoxicity against a panel of cancer cells, and GLUT transporters are involved in their transport [33].

So far, as part of the research cycle, our team has developed methods of connecting biologically active compounds with derivatives of d-glucose and d-galactose differently functionalized at the anomeric position. Our research focuses on the use of small quinoline derivative molecules as aglycones in potential anti-cancer agents [34,35,36]. The cytotoxic potential of derivatives of 8-hydroxyquinoline (8HQ) is related to its ability to complex many metal ions [37,38,39]. It is well known that cancer cells have an increased need for metal ions such as iron, copper, zinc, calcium, etc. As these microelements are involved in major cellular processes, the use of chelators appears to be an ideal way to control the level of metals in the body and is important in developing new treatment strategies [40,41,42]. However, to be effective, the glycoconjugate must meet several conditions on its way from blood vessels to a molecular target in cancer tissue. It is necessary that attaching the cytotoxic compound to the sugar does not interfere with its receptor binding specificity. Moreover, the linker between the drug and the carrier must be stable in the extracellular space and be easily degraded in target cells after uptake by receptors. Cleavage of the linker should release the unmodified drug inside the neoplastic cells. Additionally, to observe the desired cytotoxicity, sufficient intracellular drug concentration must be achieved. In the project on the targeted transport of prodrugs, we used a fragment of 1,2,3-triazole in the linker structure, a base which, due to the low extracellular pH in cancer cells, will allow for the selective placement of the prodrug in the tumor microenvironment. On the other hand, after protonation, the created cation due to the delocalization of the charge should not constitute a barrier preventing the penetration of the prodrug into the neoplastic cell [43,44,45].

**Figure 1:**
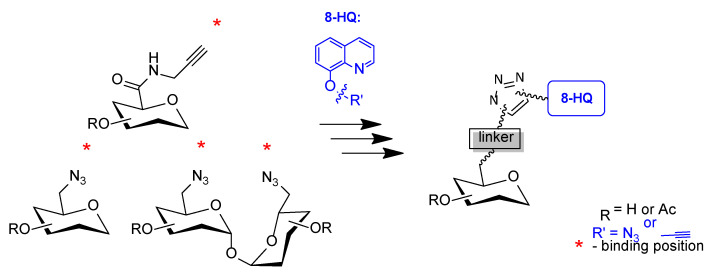
Strategies for the synthesis of quinoline glycoconjugates.

Based on literature reports and insights gained during the research on the targeted introduction of prodrugs into cancer cells, we designed a series of new compounds to better match GLUT transporters. As part of the structural modification, new glycoconjugates will be formed via the C-6 position in the sugar unit. This approach should allow increasing the selectivity in targeting glycoconjugates to tumor cells, where the units released from the prodrug will be able to induce cytotoxicity. The functionalized at the C-6 position sugar structures planned to be obtained and connected through this position to the 8HQ derivative, the fragment responsible for inducing the cytotoxic effect, are shown in Figure 1. The research includes both the synthesis of glycoconjugates and the assessment of their biological activity in vitro against selected cancer cell lines and healthy cells.

## 2. Results and Discussion

### 2.1. Synthesis

As mentioned above, the structure of the molecules presented in this work was designed based on the recently published crystal structure of the human GLUT1 transporter, according to which the least significant group for binding to the receptor is the 6-OH group. Therefore, the group at the C-6 position seems to be the best functional group to which a molecule of the drug could be attached to maintain the affinity of the glucose conjugate for the transporter. Based on assumption that the glycoconjugate will be selectively transferred to the neoplastic cell by GLUT transporters, in the first place, synthetic pathways of several sugar derivatives substituted with different groups at the C-6 position were developed. To accurately determine the route of drug absorption, both compounds with free hydroxyl groups as substrates for binding to GLUT transporters as well as compounds with acetyl protection of hydroxyl groups in the sugar unit were designed. The comparison of the effect of these two groups of compounds will allow determining whether a given compound enters the cell using passive transport (diffusion of the lipophilic compound through cell membranes) or active transport (by GLUT transporters).

**Scheme 1:**
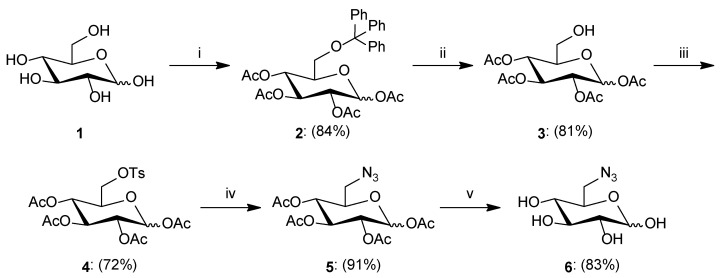
Synthesis of sugar derivatives **2**–**6**. Reagents and conditions: (**i**) 1. trityl chloride, DMAP, pyridine, r.t., 24 h; 2. CH_3_COCl, pyridine, r.t., 1 h; (**ii**) CH_3_COOH, 33% HBr/AcOH, 0 °C-r.t., 1 h; (**iii**) *p*-TsCl, DMAP, pyridine, r.t., 24 h; (**iv**) NaN_3_, DMF, 80 °C, 2 h; (**v**) 1. MeONa, MeOH, r.t., 0.5 h; 2. Amberlyst-15.

In order to obtain 6-azido-6-deoxy-d-glucopyranose, in the first approach reaction of d-glucose with *p*-toluenesulfonyl chloride in pyridine was performed [46]. The reaction was to selectively introduce into the C-6 position the tosyl as a good leaving group. The obtained product can be first treated with an acetylating agent to protect the remaining hydroxyl groups or the tosyl substitution reaction with an azide moiety can be performed right away. Unfortunately, in both approaches, a complicated, difficult to separate product mixture was obtained. Therefore, the synthesis of sugar derivatives functionalized only at the C-6 position requires other, multi-step sequences of reactions shown in Scheme 1. First, the primary hydroxyl group of d-glucose **1** at the C-6 position was selectively protected with a sterically large triphenylmethyl (trityl, Tr) group which was introduced using triphenylmethyl chloride in the presence of a catalytic amount of DMAP [47]. The reaction was carried out in pyridine until the substrate was completely converted to the product as monitored by TLC. The remaining secondary sugar hydroxyl groups were then protected with an acetyl moiety by adding acetyl chloride to the reaction mixture, resulting in the formation of fully protected 1,2,3,4-tetra-*O*-acetyl-6-*O*-triphenylmethyl-d-glucopyranose **2** with 84% yield. In the next step, the trityl protection at C-6 carbon was removed under acidic conditions with 33% HBr in acetic acid, receiving 1,2,3,4-tetra-*O*-acetyl-d-glucopyranose **3** [47]. Then, an easily leaving *p*-toluenesulfonyl group (tosyl, Ts) was introduced into the 6-OH position using *p*-toluenesulfonyl chloride [48]. This group was substituted with an azide moiety in the reaction with sodium azide in DMF to give 1,2,3,4-tetra-*O*-acetyl-6-azido-6-deoxy-d-glucopyranose **5** [46]. The introduction of the azide group required heating of the reaction mixture at 80 °C for 2 h. The lower reaction temperature did not allow the substrate to react completely even despite the significant increase in reaction time. The presence of the CH_2_N_3_ carbon signal with a shift of about 50 ppm in the ^13^C NMR spectra confirms obtaining the product with an azide moiety. Removal of acetyl protecting groups from product **5** was performed according to the classic Zemplén protocol under alkaline conditions using a solution of sodium methoxide in methanol to give the desired product 6-azido-6-deoxy-d-glucopyranose **6** with 83% yield [49]. The structures of all intermediates were confirmed by analysis of ^1^H and ^13^C NMR spectra. As a result of individual reactions, the formation of products in the form of a mixture of α and β anomers was observed.

**Scheme 2:**
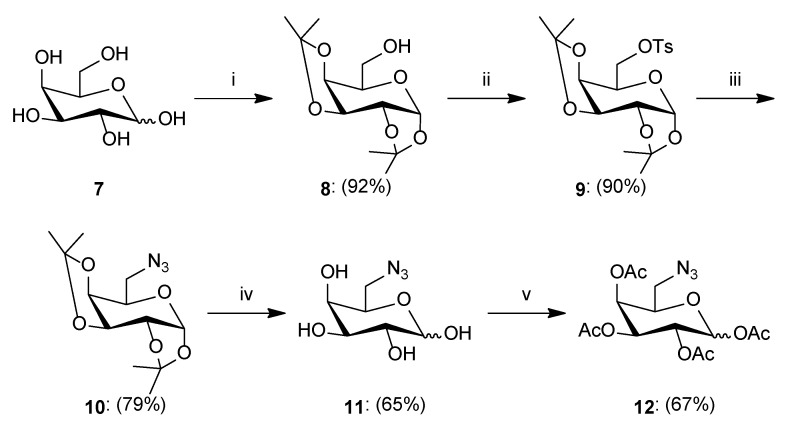
Synthesis of sugar derivatives **8**–**12**. Reagents and Conditions: (**i**) I_2_, acetone, r.t., 24 h; (**ii**) *p*-TsCl, DMAP, pyridine, r.t., 24 h; (**iii**) NaN_3_, DMF, 120 °C, 24 h; (**iv**) 80% CF_3_COOH, 0 °C-r.t., 2 h; (**v**) CH_3_COONa, Ac_2_O, reflux, 1 h.

The derivative of d-galactose containing the azide moiety at position C-6 was obtained according to the sequence shown in Scheme 2. For one-step protection of the galactose hydroxyl groups at C1–C4 positions, isopropylidene groups were applied. For this purpose, di-*O*-isopropylideneation of d-galactose was carried out using acetone both as a solvent and as a reagent. The reaction was performed in the presence of catalytic amounts of iodine at room temperature giving product **8** with a very good yield [50]. As in the case of d-glucose derivatives, activation of the hydroxyl group at the C-6 position was carried out by introducing a good leaving group using *p*-toluenesulfonyl chloride in pyridine in the presence of a catalytic amount of DMAP to give product **9**, which participated in the nucleophilic substitution reaction with sodium azide in DMF carried out at an increased temperature of 120 °C [48]. Removal of the isopropylidene groups from **10** was performed in an acidic environment with 80% trifluoroacetic acid [50] to obtain the desired 6-azido-6-deoxy-d-galactopyranose **11**. Acetylation of the latter one with acetic anhydride in the presence of sodium acetate allowed to obtain 1,2,3,4-tetra-*O*-acetyl-6-azido-6-deoxy-d-galactopyranose **12**. Product structures were confirmed by analysis of the NMR spectra. Products **11** and **12** were obtained as a mixture of α and β anomers.

**Scheme 3:**
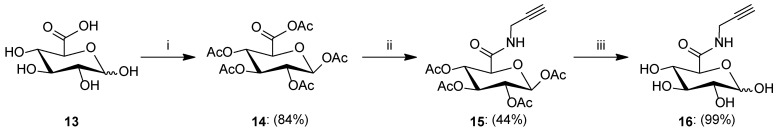
Synthesis of sugar derivatives **14**–**16**. Reagents and Conditions: (**i**) Ac_2_O, I_2_, 0 °C-r.t., 3 h; (**ii**) propargyl amine, CH_2_Cl_2_, r.t., 24 h; (**iii**) 1. MeONa, MeOH, r.t., 0.5 h; 2. Amberlyst-15.

Having glucuronic acid, i.e., a monosaccharide in which the C-6 position is oxidized to the carboxyl group, we decided to obtain derivatives containing an amide bond in the structure. The propargyl sugar derivative was prepared as shown in Scheme 3, according to the previously described procedure [51]. The synthesis started with the acetylation of commercially available d-glucuronic acid **13** using acetic anhydride in the presence of a catalytic amount of iodine as the acetyl transfer reagent. The obtained mixed anhydride **14** was treated with propargylamine in dichloromethane to give the protected amide **15** with 44% yield after column chromatographic purification. From the NMR spectra, it can be seen that compounds **14** and **15** were obtained only as a β-anomer. This is confirmed by the high coupling constant from proton H-1 equal to about *J* = 8.0 Hz observed in the ^1^H NMR spectra. In the last step, the protective groups were removed under basic conditions by the Zemplén method, giving the expected *N*-(prop-2-yn-1-yl)-d-glucopyranuronic acid amide **16** as a mixture of anomers, as confirmed by NMR analysis.

**Scheme 4:**
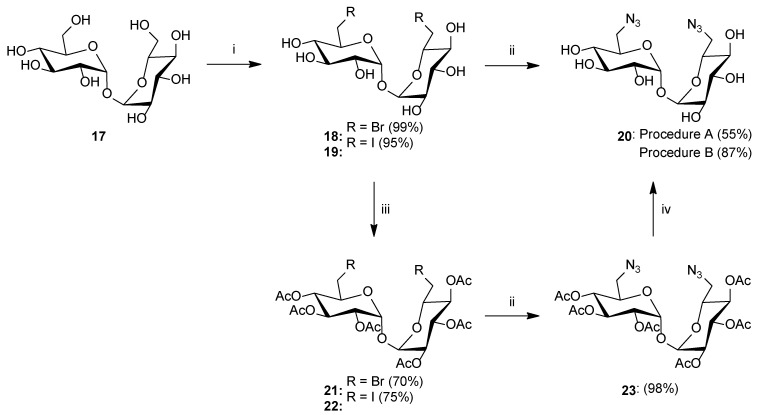
Synthesis of sugar derivatives **18**–**23**. Reagents and Conditions: (**i**) PPh_3_, NBS, DMF, 0 °C-r.t., 72 h (**18**) or PPh_3_, I_2_, DMF, 0 °C-r.t., 72 h (**19**); (**ii**) NaN_3_, DMF, 60 °C, 24 h; (**iii**) Ac_2_O, pyridine, 0 °C-r.t., 24 h; (**iv**) 1. MeONa, MeOH, r.t., 1 h; 2. Amberlyst-15.

Next, structures based on trehalose were designed. Trehalose is a disaccharide composed of two d-glucose molecules linked by α,α-1,1′-*O*-glycosidic bond. For this reason, it is quite easy to modify the C-6 position in this molecule. This disaccharide was selected because, as a result of the hydrolysis of the glycosidic bond in trehalose, two C-6 substituted glucoconjugate molecules will be released, preferred as ligands of the GLUT1 transmembrane protein responsible for glucose transport to pathological cells, where the units released from the prodrug will be able to induce cytotoxicity. The synthesis was started according to the previously described procedure, which involved tosylation of the substrate followed by displacement with azide [52]. Unexpectedly, the tosylation of trehalose led to a mixture of products. After the purification of the reaction mixture by crystallization and column chromatography, only traces of the expected product are isolated. Therefore, the reaction sequence shown in Scheme 4 was performed to obtain 6,6′-diazido-6,6′-dideoxy-d-trehalose [53,54]. Trehalose **17** was first dissolved in DMF and then brominated with NBS or iodinated with I_2_ in the presence of triphenylphosphine. As a result of these reactions, the corresponding halide derivatives **18** or **19** with high yields were obtained. Then, two synthesis variants were tested. The first was the substitution of halogen atoms in 6,6′-dibromo-6,6′-dideoxy-d-trehalose **18** with sodium azide in DMF (Procedure A). Product **20**, after purification from the multi-component reaction mixture, was isolated with a moderate yield of 55%. Modification of the temperature and reaction time also did not help in this case. The second method (Procedure B) assumes acetylation of compounds **18** or **19** with acetic anhydride in pyridine, isolation of acetylation products **21** or **22** by column chromatography, and then substitution of bromine or iodine from such protected compounds with an azide moiety using sodium azide. Nucleophilic substitution of the protected sugars **21** and **22** with sodium azide proceeds with a much better yield (98%) than in the case of the reaction with the unprotected molecule. De-*O*-acetylation of compound **23** under Zemplén conditions gave 6,6′-diazido-6,6′-dideoxy-d-trehalose **20** with 87% yield.

**Scheme 5:**
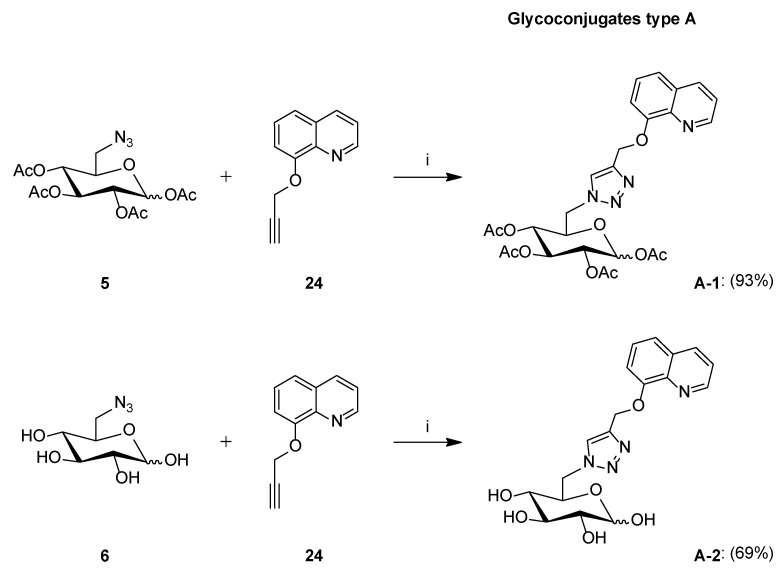
Synthesis of *type A* glycoconjugates. Reagents and Conditions: (**i**) CuSO_4_·5H_2_O, NaAsc, *i*-PrOH/THF/H_2_O (1:1:1, *v*:*v*:*v*), r.t., 24 h.

**Scheme 6:**
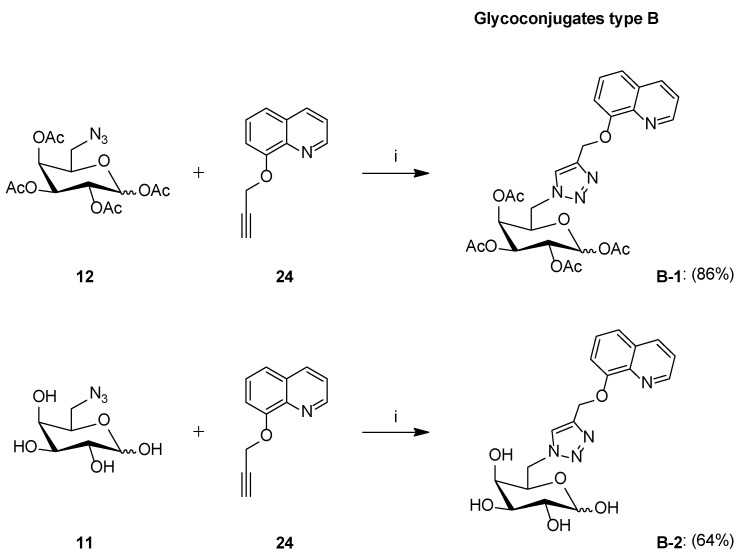
Synthesis of *type B* glycoconjugates. Reagents and Conditions: (**i**) CuSO_4_·5H_2_O, NaAsc, *i*-PrOH/THF/H_2_O (1:1:1, *v*:*v*:*v*), r.t., 24 h.

**Scheme 7:**
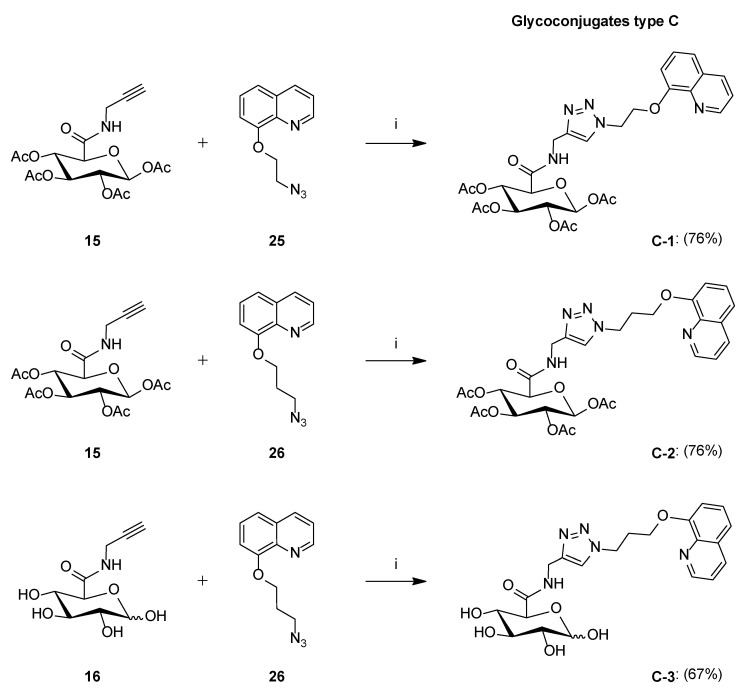
Synthesis of *type C* glycoconjugates. Reagents and Conditions: (**i**) CuSO_4_·5H_2_O, NaAsc, *i*-PrOH/THF/H_2_O (1:1:1, *v*:*v*:*v*), r.t., 24 h.

**Scheme 8:**
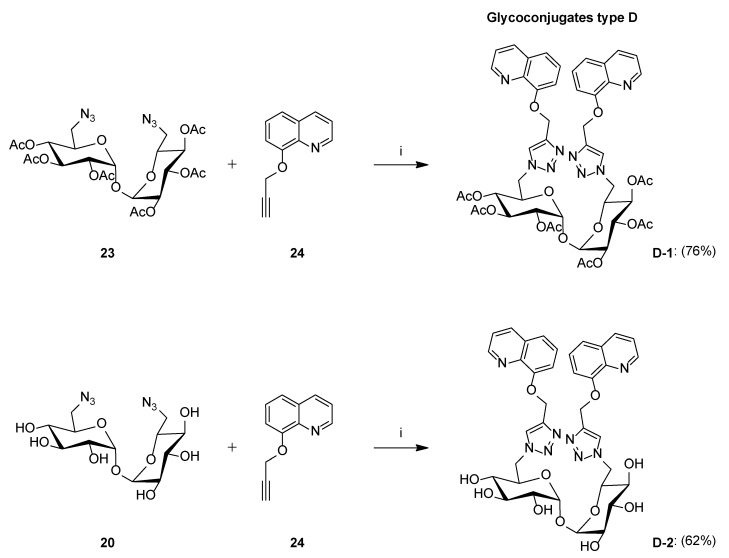
Synthesis of *type D* glycoconjugates. Reagents and Conditions: (**i**) CuSO_4_·5H_2_O, NaAsc, *i*-PrOH/THF/H_2_O (1:1:1, *v*:*v*:*v*), r.t., 24 h.

The glycoconjugates described in this paper have been divided into 4 types and their synthesis is presented in Scheme 5, Scheme 6, Scheme 7 and Scheme 8. The designed constructs contain sugar derivatives functionalized at the C-6 position linked via a linker containing a 1,2,3-triazole moiety to 8-hydroxyquinoline derivatives. The synthesis of 8-(2-propyn-1-yloxy)quinoline **24**, 8-(2-azidoethoxy)quinoline **25**, and 8-(3-azidopropoxy)quinoline **26** were described in the earlier works [34,35]. Glycoconjugates were prepared by the copper(I)-catalyzed 1,3-dipolar azide-alkyne cycloaddition, in a variant developed by Sharples [55,56]. The synthesis procedure consists of mixing the terminal alkyne and the terminal azide in an equimolar ratio in the solvent system of tetrahydrofuran and isopropanol and then adding to the mixture an aqueous catalyst system consisting of copper sulfate and sodium ascorbate. The last one is used to reduce Cu(II) to Cu(I), needed for accelerates the reaction and guarantee control over its regioselectivity. The use of such reaction conditions leads to the target products being obtained with high yields. This reaction does not generate any byproducts, only the 1,4-disubstituted isomers of 1,2,3-triazole are obtained. Already after a few hours at room temperature, TLC analysis of the reaction mixture showed almost complete conversion of the starting materials to the target product. In order to purify the crude product, it is sufficient to filter the precipitate of inorganic salts from the reaction mixture, remove the solvent in vacuo, and load onto a chromatographic column.

**Scheme 9:**
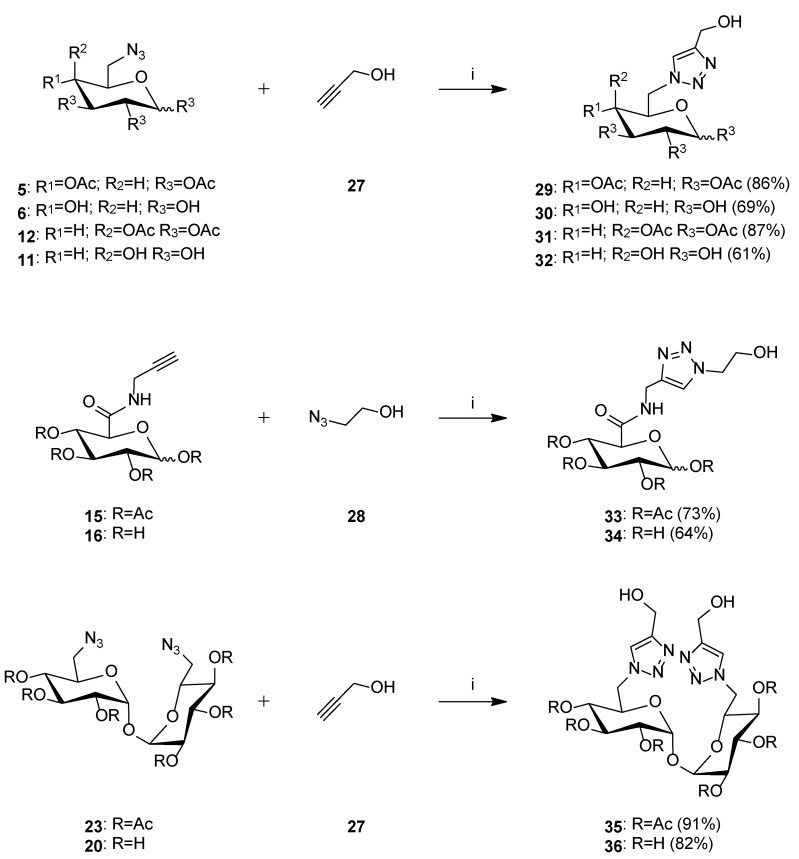
Synthesis of metabolites of sugar derivatives. Reagents and Conditions: (**i**) CuSO_4_·5H_2_O, NaAsc, *i*-PrOH/THF/H_2_O (1:1:1, *v*:*v*:*v*), r.t., 24 h.

The resulting glycoconjugates were designed as prodrugs that could be metabolized in the cell by hydrolytic enzymes into simpler compounds. Therefore, except for the assessment of the cytotoxicity of the glycoconjugates themselves, it is also worth determining the activity of potential products of their degradation [57]. For this purpose, the potential sugar metabolites **29**–**36** presented in Scheme 9 were designed and prepared. The obtained sugar derivatives were reacted with propargyl alcohol **27** or 2-azidoethanol **28**, under the same reaction conditions as described for glycoconjugates synthesis.

The structures of all newly created products were confirmed using nuclear magnetic resonance spectroscopy (^1^H NMR and ^13^C NMR) and high-resolution mass spectrometry (HRMS). The formation of the desired products was indicated by the presence of characteristic signals from the 1,2,3-triazole ring in the NMR spectra. These are the singlet signal observed at about δ = 8.0 ppm from the H-C(5) proton of triazole ring in ^1^H NMR spectra and two characteristic carbon signals at about 124 ppm and 144 ppm for C(4) and C(5) in the ^13^C NMR spectra. The double signals for the sugar part observed in the NMR spectra and the characteristic coupling constants from the H-1 protons (*J* = 8.2 Hz for the β anomer and *J* = 3.7 Hz for the α anomer) indicate the formation of glycoconjugates as a mixture of anomers. The NMR spectra of all synthesized products are presented in the Supplementary Materials.

### 2.2. Biological Evaluation

#### 2.2.1. Antiproliferative Studies

An in vitro biological test package was performed for the obtained derivatives. All newly synthesized compounds were assessed for their cytotoxic activity against human cell lines with increased GLUT expression: the colon cancer cell line (HCT-116) and the breast cancer cell line (MCF-7), as well as on normal human dermal fibroblasts cells (NHDF-Neo), using the MTT assay (3-(4,5-dimethylthiazol-2-yl)-2,5-diphenyltetrazolium bromide). The MTT test is a colorimetric method for detecting proliferating cells. It is based on the ability of the enzyme mitochondrial dehydrogenase to convert yellow tetrazole salt (MTT) into purple formazan. The amount of the product formed in the reaction, determined spectrophotometrically, is proportional to the number of living cells [58]. The antiproliferative activity was expressed as the concentration that caused 50% inhibition of the growth of the population of cells treated with the test compounds relative to the control cells (inhibitory concentration activity—IC_50_). Table 1 and Table 2 summarize the IC_50_ values calculated for all tested compounds.

The conducted cytotoxicity studies show that both sugar substrates and possible potential glycoconjugate metabolites did not show significant growth inhibitory activity on the tested cells (Table 1). This means that for the obtained constructs to show the expected activity, they should reach the interior of the cell in an unchanged form, without any degradation. The tested glycoconjugates inhibited cell growth in a dose-dependent manner, which allowed the expression of their activity through IC_50_ values (Table 2). 24 h treatment with candidate drugs of the HCT-116 cell line showed moderate cytotoxic potency of the tested glycoconjugates. After increasing the incubation time to 72 h, the HCT-116 cell line was more sensitive to the presence of the tested derivatives. Experiments for MCF-7 and NHDF-Neo cell lines were carried out for 72 h due to longer times of cell division of this line. Owing to the tests performed on healthy cells, it was possible to assess the selectivity of the tested compounds in relation to neoplastic cells, which is crucial in the design and synthesis of antiproliferative compounds. It is worth noting that most of the tested compounds showed lower cytotoxicity on normal human NHDF-Neo cells than on cancer cells. The selectivity index (SI) was calculated as a ratio of the IC_50_ value for healthy cells (NHDF-Neo) and the IC_50_ value for cancer cells (HCT-116 or MCF-7) and is presented in Table 3.

The highest growth inhibitory activity of both cancer cell lines was noted for glycoconjugates *type A* and *type D*. In these derivatives, the triazole-quinoline was attached through the triazolic nitrogen atom to the d-glucose unit directly to the carbon at the C-6 position. In the case of compounds **A-1** and **A-2**, the calculated IC_50_ values after 72 h of incubation for HCT-116 were equal to 11.98 ± 0.90 µM and 22.32 ± 0.70 µM, respectively, while for MCF-7 were equal to 52.84 ± 0.75 µM and 54.92 ± 8.71 µM. For compounds **D-1** and **D-2**, IC_50_ values were recorded of 26.33 ± 0.72 µM and 21.28 ± 0.94 µM for HCT-116 and 30.71 ± 0.99 and 34.04 ± 0.95 µM for MCF-7, whereas glycoconjugates *type A* were characterized by a higher selectivity index (SI = 10.83, 14.21 for HCT-116 and 2.46, 5.78 for MCF-7), in contrast to trehalose derivatives *type D*, which showed a similar level of antiproliferative activity against healthy NHDF-Neo cells (SI = 1.31, 3.32 for HCT-116 and 1.12, 2.08 for MCF-7). The HCT-116 cell line was characterized by higher sensitivity to the tested compounds. The selectivity indexes for this line were also more favorable. It is also worth emphasizing that the **A-2** derivative shows comparable antiproliferative activity to the **A-1** derivative against cancer cell lines. However, the selectivity of **A-2**, i.e., a conjugate containing an unprotected glucose residue and thus having an increased affinity for GLUT1, was higher compared to derivative **A-1** with acetylated sugar part. GLUT transporters are not overexpressed in NHDF-Neo cells, therefore in the case of deprotected derivatives, it was expected to observe lower cytotoxicity than in the case of acetylated derivatives, where passive transport of compounds across the cell membrane is not selective. Similarly, due to the lack of selectivity, the involvement of GLUT proteins in the transport of trehalose derivatives should be excluded. Probably in the case of *D-type* glycoconjugates, the disaccharide was not hydrolyzed to two glucose units, which made the binding to the GLUT transporter much more difficult.

*Type B* glycoconjugates derived from d-galactose cannot be considered compatible with the GLUT transporter. The deprotected **B-2** derivative was shown to be less cytotoxic than its acetylated counterpart **B-1** and had one of the highest IC_50_ values among all compounds tested. The higher anticancer activity of the **B-1** derivative with acetyl protection in the sugar part indicates that this type of compound prefers passive transport across the cell membrane. At the same time, this derivative is also cytotoxic to healthy cells, because this transport is not selective.

Tests show that glycoconjugates *type C* are also not good substrates for GLUT transporter proteins. After 72 h of incubation time, the **C-3** derivative remained inactive against the cancer cell lines tested. Probably a rigid amide fragment in the structure between the sugar moiety and the triazole ring limits the flexibility of the molecule and its possibility to fit into the GLUT transporter. The presence of the amide bond also did not improve the activity of glycoconjugates with acetyl protections, as compounds **C-1** and **C-2** did not show significant ability to inhibit cell growth and are characterized by quite high IC_50_ values. This suggests that a too stable linker is unable to release the cytotoxic charge. It was noted that the **C-2** derivative was slightly more active than the **C-1** derivative. This is due to the difference in linker length between the quinoline and triazole parts. Such regularity has already been observed during the cytotoxicity testing of previously prepared glycoconjugates [35]. Interestingly, compound **C-2** was not highly toxic to normal human skin cells (SI = 5.11 for HCT-116 and 2.03 for MCF-7). Because of these observations, it is advisable to test these structures on a different panel of tumor cell lines in the future. Additionally, for these derivatives, the reverse orientation of the 1,2,3-triazole system in the linker structure was designed compared to the other tested glycoconjugates. Perhaps this treatment also has a negative impact on the biological activity of these connections.

**Figure 2:**
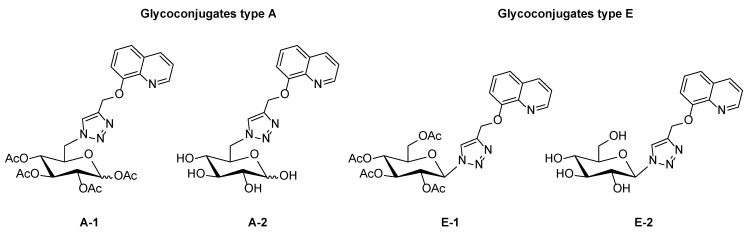
Structures of analogous quinoline glycoconjugates functionalized at the C-6 position (**A-1**, **A-2**) or the anomeric position (**E-1**, **E-2**).

**Figure 3:**
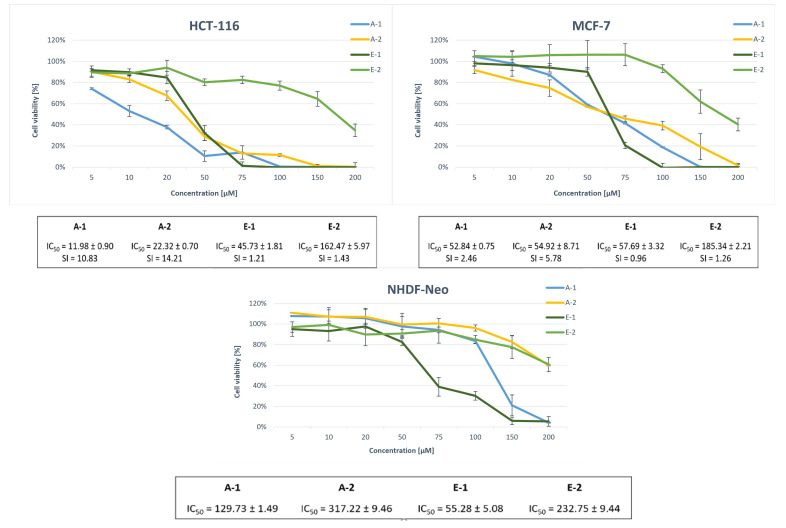
Comparison of the viability of HCT-116, MCF-7, and NHDF-Neo cells treated with different concentrations of the tested glucoconjugates: **A-1**, **A-2**, **E-1**, and **E-2**. Cell viability was measured by the MTT test. Data are presented as the mean ± standard deviation (*n* = 3).

Several additional experiments were performed to evaluate the effect of the substitution position of a given hydroxyl group of a sugar unit on the activity of glycoconjugates. The cytotoxicity of the most active derivatives described for the first time in this paper was compared with the previously obtained analogous glycoconjugates formed through the C-1 anomeric position of d-glucose [34]. Figure 2 shows the structures of analogous quinoline glycoconjugates functionalized at the C-6 position (**A-1, A-2**) and the anomeric position (**E-1, E-2**), and Figure 3 compares their cytotoxic activity as well as calculated and predicted IC_50_ values and selectivity indexes.

The presented data show that the C-6 functionalized glycoconjugates show some differences in activity compared to the analogous structures of the C-1 functionalized glycoconjugates. Namely, after 72 h of incubation of cells with tested compounds, *type A* glycoconjugates compared to *type E* glycoconjugates, show increased toxicity against HCT-116 and MCF-7 cancer cells. For both tumor cell lines, 100% inhibition of cell growth was recorded at the highest doses of **A-1**, **A-2**, and **E-1**. It should be noted that in both cell lines it is the deprotected **A-2** derivative that is characterized by much better cytotoxicity than the analogous **E-2** derivative, which practically did not affect the cell viability. This suggests an increased permeability of the cell membrane, possibly through the involvement of GLUT proteins to transport a new type of glycoconjugate across the cell membrane. In addition, both the **A-1** and **A-2** derivatives exhibit reduced toxicity to normal cells as compared to previously reported glycoconjugates modified in an anomeric position which were characterized by rather a low SI. These results indicate that the C-6 substitution of d-glucose is preferable to the C-1 substitution for cellular uptake of quinoline glycoconjugates in neoplastic cells. These properties suggest that the newly designed glycoconjugates show great potential for further research.

#### 2.2.2. Effect of the GLUT1 Inhibitor

**Figure 4:**
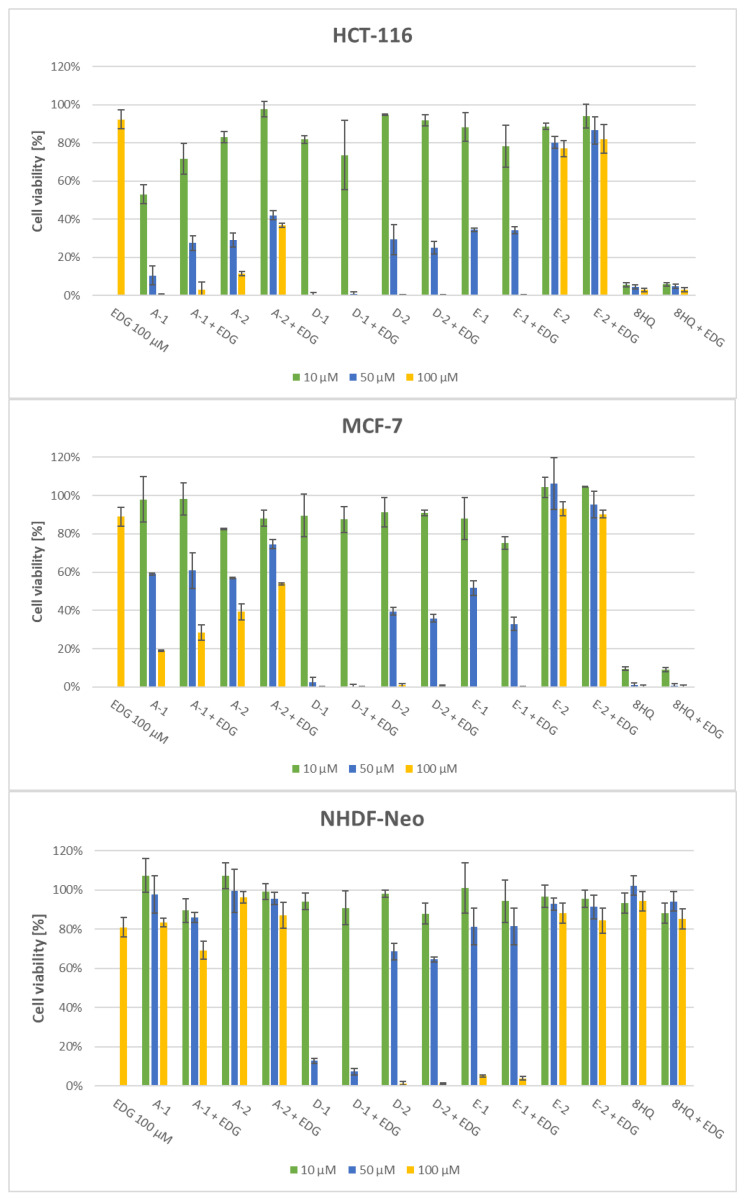
Effect of glucose transporter GLUT1 inhibitor EDG on the activity of compounds **A-1**, **A-2**, **D-1**, **D-2**, **E-1**, **E-2**, and 8HQ for 72 h incubation with HCT-116, MCF-7, and NHDF-Neo cells. Cell viability was measured by the MTT test. Data are presented as the mean ± standard deviation (*n* = 2).

To investigate the possible interaction of the tested compounds with glucose receptors and thus to check whether they target the Warburg effect, tests of the influence of the presence of the GLUT inhibitor on the effectiveness of the glycoconjugates were carried out. For this, cells were treated each time with the test compounds in the presence of 100 µM of the GLUT1 inhibitor 4,6-*O*-ethylidene-α-d-glucose (EDG). After 72 h of incubation with test compounds, cell viability was measured and presented in Figure 4.

The obtained results confirmed that GLUT transporters are involved in the transport of *type A* glycoconjugates to the cell. Following the use of an EDG inhibitor and partial inhibition of GLUT activity, probably decreased compound uptake occurred, which resulted in less accumulation in cells and less anti-cancer activity. For the HCT-116 cell line, a 13–25% increase in cell viability was observed in the concentration range tested for compound **A-2** and by 3–19% for compound **A-1**. While MCF-7 cell viability was increased by 6–15% in the concentration range tested for compound **A-2** and 2–10% for compound **A-1**. In the case of the NHDF cell line, even a slight decrease in cell viability was observed in the presence of EDG as compared to cells without the inhibitor. The increase in cytotoxicity in the presence of a GLUT inhibitor is due to the restriction of glucose transport, which is the main nutrient entry for proliferating cells. The observations for the **A-2** glycoconjugate are as expected as its structure allows for a fit with GLUT transporters. In contrast, compound **A-1** is a hydrophobic molecule, so it is unlikely that in this form it undergoes a different transport than passive diffusion through the lipid cell membrane. The possible explanation for the partial uptake of **A-1** by GLUT transporters may be the possibility of partial hydrolysis of acetyl protections by hydrolytic enzymes in the extracellular space. To determine how the position of the substitution in d-glucose influences the specific uptake of the resulting conjugates, glycoconjugates *type E* were also used for the tests. It turned out that in both tumor cell lines there was little or no significant change in cell viability in the presence of the EDG inhibitor. This result supports the hypothesis that the position of the sugar substitution is an important parameter in determining the GLUT1 specificity of the glycoconjugates. As expected, it was also found that the use of an EDG inhibitor did not have any effect on the cellular uptake of *D-type* glycoconjugates and the 8HQ aglycone.

GLUTs are the most common transporters that facilitate the recognition and uptake of glycoconjugates. As mentioned, the use of an EDG inhibitor had the effect of changing the survival of cells treated with glycoconjugates *A-type*. However, it cannot be clearly stated that the cellular uptake of the newly designed **A-2** glycoconjugate depends only on GLUT. Perhaps the dose of the inhibitor turned out to be too low and increasing its concentration would result in greater inhibition of uptake. It should also be noted that the transport of sugars is mediated not only by GLUT but also by other receptors, such as SGLT, SWEET, OCT2, or ASGPR [19,59].

#### 2.2.3. Clonogenic Assay

Then, the clonogenic potential of cells exposed to the tested compounds was investigated [60]. The ability of a cell to form colonies is one of the defining features of cancer and determines its ability to metastasize. Thus, the test is used to evaluate the efficacy of anticancer therapy in vitro by observing the ability of the treated cell to divide.

**Figure 5:**
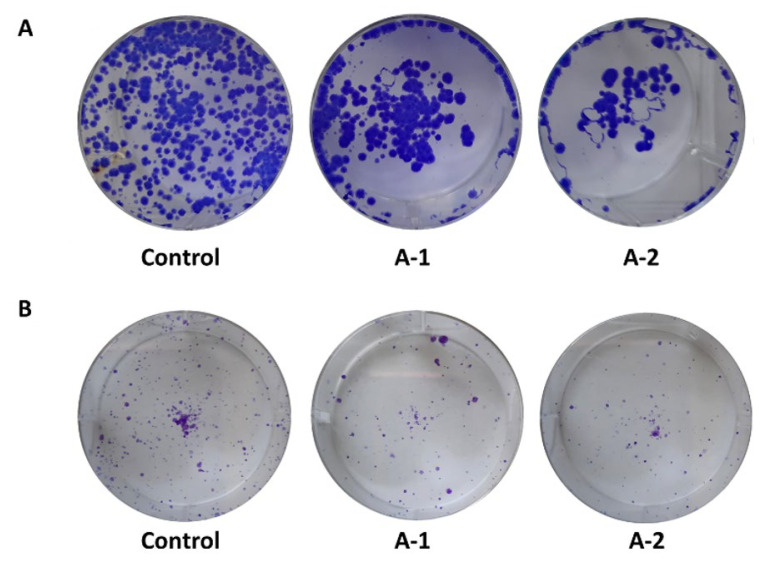
Representative colony formation images of: (**A**) HCT-116 cancer cells and (**B**) MCF-7 cancer cells, after 72 h treatment with compounds **A-1** and **A-2**.

MCF-7 and HCT-116 cells were treated with *type A* glycoconjugates and their ability to proliferate was observed. Cells were incubated with compounds at IC_50_ concentrations for 72 h, and then the cells were collected, seeded again, and incubated for 10 days in a fresh medium. The formed colonies were stained with crystal violet (Figure 5). The control shows the ability of a single cell to grow back into a large colony that can be seen with the naked eye. The results indicate that **A-1** and **A-2** derivatives were able to decrease the clonogenic potential of HCT-116 and MCF-7 cells under the experimental conditions. Similar effects were observed for both compounds.

#### 2.2.4. Wound Healing Assay

Cell migration is a hallmark of many physiological and pathological processes such as wound healing, cancer invasion, and metastasis. To investigate the migration capacity of cells after exposure to test compounds, a wound-healing assay was performed, which is a standard in vitro technique used in small molecule screening for the discovery of drug candidates [61,62].

**Figure 6:**
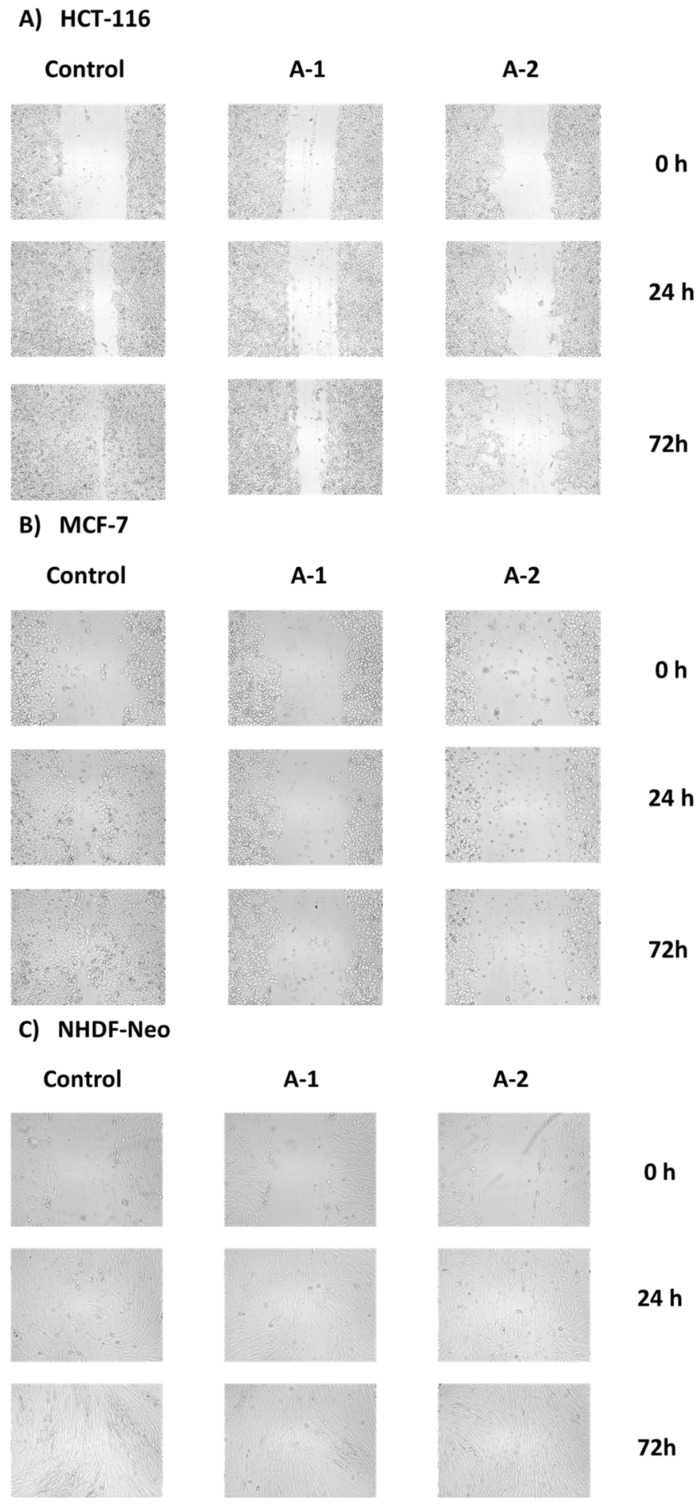
Representative images of: (**A**) HCT-116 cancer cells, (**B**) MCF-7 cancer cells, and (**C**) NHDF-Neo healthy cells, were acquired at time 0 and after 24 h and 72 h incubation with compounds **A-1** and **A-2** in wound healing assay. Better quality and larger images are included in Supplementary Materials (Figure S75).

This experiment consisted in making a “wound” in a confluent monolayer of HCT-116 and MCF-7 cells, which were next treated with the test compounds at their IC_50_ concentrations. Then, the migration of cells was observed for 72 h in order to fill the formed gap (Figure 6). In the control, cell migration from the edges of the wound is visible, gradually filling the gap. After 72 h, the wounds are almost completely covered with cells. In the same way, cell migration is observed in healthy NHDF cells. On the other hand, a significantly slower migration of HCT-116 and MCF-7 cells was observed after treatment with the tested **A-1** and **A-2** glycoconjugates under experimental conditions. After 72 h, an open wound comparable to the initial one can be noticed.

#### 2.2.5. Apoptosis and Cell Cycle Analyses by Flow Cytometry

**Figure 7:**
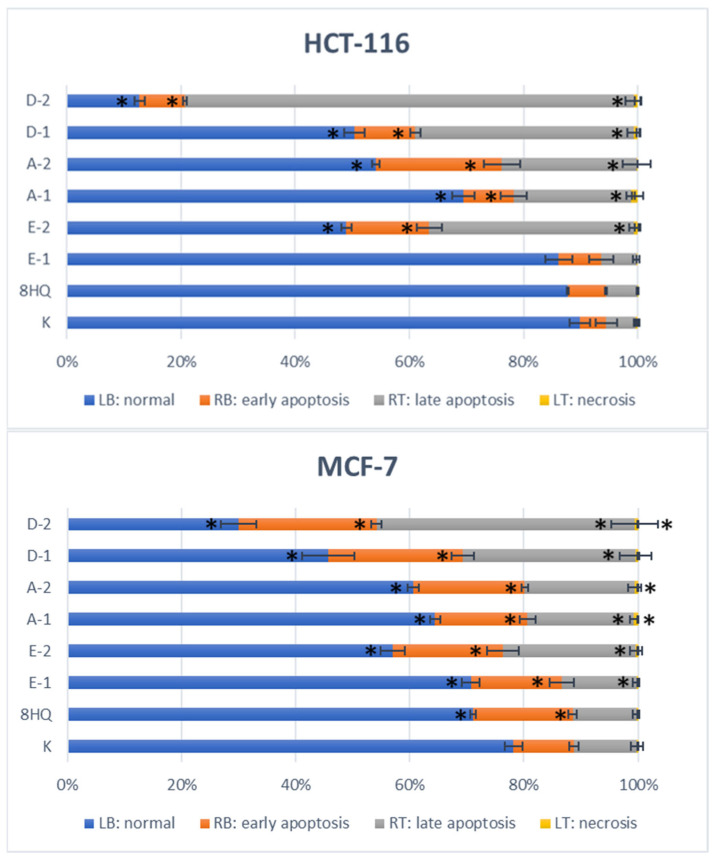

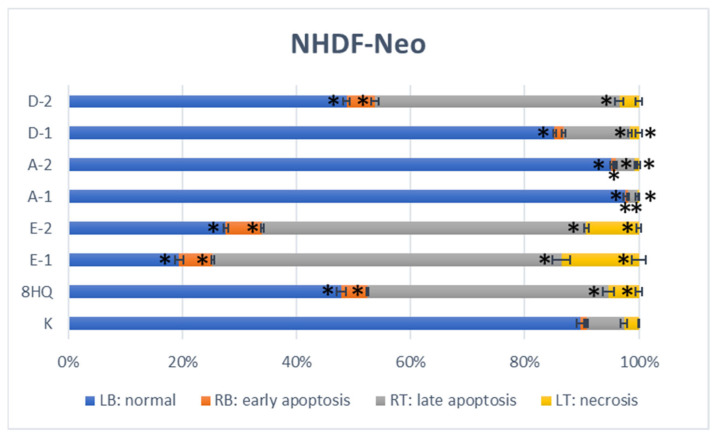
Flow cytometry analysis of HCT-116, MCF-7, and NHDF-Neo cell apoptosis after incubation for 24 h with test compounds used in their IC_50_ doses. The fractions of dead cells were detected by the FITC Annexin-V Apoptosis Detection Kit with PI. Means ± SD from three independent experiments. * *p*-value < 0.05, in comparison to the control.

After determining the cytotoxic activity of the tested compounds in the MTT test and calculating their IC_50_ parameters, cytometric measurements were started to determine what kind of cell death was caused by the most active compounds. This is important information from the point of view of drug design, as it provides detailed information on the cytotoxicity of a compound. Apoptosis is a natural biological process involving programmed cell death and plays an important role in maintaining correct homeostasis between cell proliferation and cell death. This is in contrast to necrosis, which is accompanied by acute inflammation, preceded by cell degradation due to the loss of the integrity of the cell membrane. It is possible to distinguish viable cells from apoptotic and necrotic cell fractions using Annexin V-FITC and Propidium Iodide (PI). Annexin V-FITC labels phosphatidylserine residues that, after the start of apoptosis, are located on the outer surface of the cell membrane, enabling the identification of cells in the early stages of apoptosis. PI binds stoichiometrically to intracellular DNA during late apoptosis and necrosis when the cell membrane has been damaged. This combination allows for the differentiation of early apoptotic (A+/P−), late apoptotic (A+/P+), necrosis (A−/P+), and normal (A−/P−) cells that can be quantified by flow cytometry. The experiments were performed on the HCT-116 and MCF-7 cancer cell lines and the NHDF-Neo healthy cell line. Cells were treated with compounds at concentrations equal to their previously calculated IC_50_ value and allowed to incubate for 24 h. The results of the level of apoptosis induction by the tested compounds are presented in Figure 7 and in the Supplementary Materials (Figure S73).

After 24-h incubation of HCT-116 and MCF-7 cancer cells with test compounds, the appearance of a certain percentage of apoptotic cells was observed. However, fortunately, neither compound caused necrotic cell death. This means that the applied IC_50_ dose affects the entry of cells into the apoptotic pathway and does not cause uncontrolled pathological cell death as a result of mechanical damage. Induction of apoptosis as a result of DNA damage in precancerous lesions can remove potentially pathological cells, thus inhibiting tumor proliferation. Comparing the obtained results with the control fraction, a higher percentage of apoptotic cells was noted for the HCT-116 line, which confirms the previous conclusions about the high sensitivity of this cell line to the tested compounds. In the case of the MCF-7 cell line, a lower percentage of apoptotic cells was observed under the experimental conditions, which may be due to the short incubation time of the cells with the test drugs. A noticeably higher fraction of cells in early and late apoptosis was observed during the action of compounds without the protection of the hydroxyl groups in the sugar unit, compared to their acetylated counterparts. Moreover, the glycoconjugates induce both early and late apoptosis much more efficiently than the 8HQ aglycone, for which results were similar to the control fraction. The opposite is true for the healthy NHDF-Neo cell line, where most of the glycoconjugates showed less toxicity. After 24 h of incubation of cells with test compounds, the derivative of trehalose **D-2** showed the strongest pro-apoptotic properties (87% apoptotic cells for HCT-116 and 70% apoptotic cells for MCF-7). At the same time, this compound showed high toxicity also to healthy cells (51% of the dead cell fraction for NHDF-Neo). This indicates that under the influence of this compound, healthy cells were also damaged and entered the apoptotic pathway. The glycoconjugate **E-1** more strongly inhibited the proliferation of cancer cells in the MTT assay than the glycoconjugate **E-2**, but as it turned out, it did not induce apoptosis in the HCT-116 or MCF-7 cell lines. Interestingly, it was the deprotected derivative that was characterized by better proapoptotic properties than the protected compound. In addition, in the case of the NHDF-Neo cell line, for glycoconjugates *type E*, i.e., compounds with an aglycon attached through the anomeric position of the sugar, some necrotic cells (14% for **E-1** and 9% for **E-2**) and a very large number of cells in late apoptosis (61% and 57%) were noted. Finally, cells treated with derivatives **A-1** and **A-2** showed signs of apoptosis in HCT-116 (29% and 46% apoptotic cells, respectively) and MCF-7 (35% and 39% apoptotic cells, respectively). In contrast, in the case of the NHDF-Neo cell line, no increased percentage of dead cells for these compounds was observed compared to the control. After 24 h of incubation, 98% and 95% of normal cells remained for **A-1** and **A-2**, respectively. The remaining compounds induced apoptosis in NHDF cells as compared to control. This confirms the higher safety of C-6 functionalized glycoconjugates previously noted in the MTT test compared to the glycoconjugates modified at the anomeric position.

**Figure 8:**
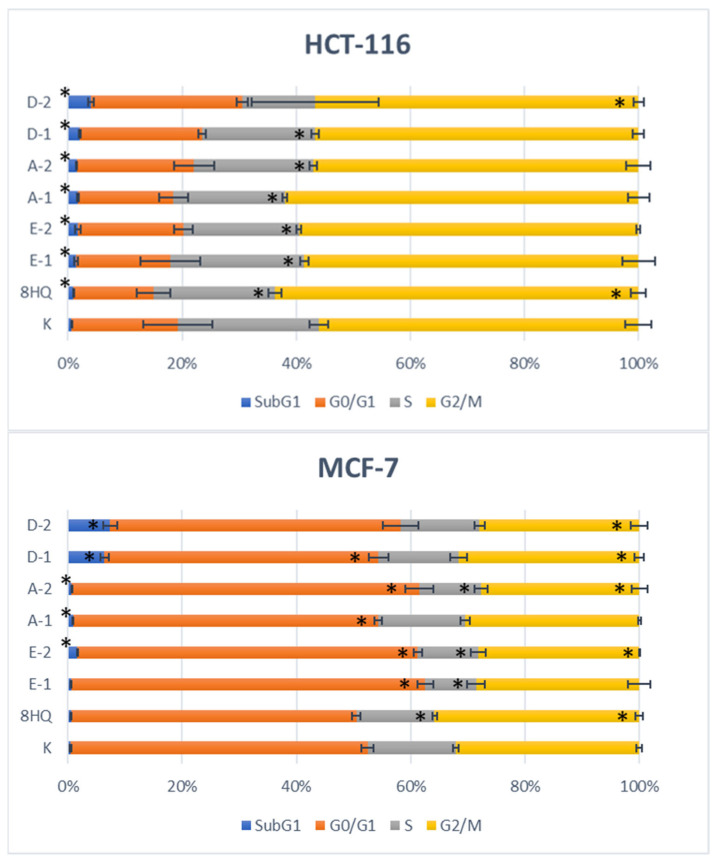

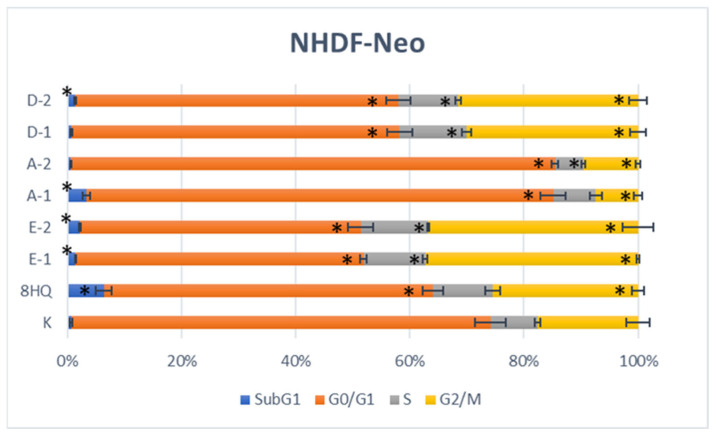
Flow cytometry analysis of HCT-116, MCF-7, and NHDF-Neo cell cycle distribution after incubation for 24 h with test compounds used in their IC_50_ doses. Means ± SD from three independent experiments. * *p*-value < 0.05, in comparison to the control.

After determining the type of cell death caused by the test compounds, the flow cytometry technique was used to study their influence on potential changes in the course of the cell cycle of the HCT-116, MCF-7, and NHDF-Neo lines. The cell cycle is the sequence of growth and division of cells, both normal and cancerous. This process begins from the resting phase (G0) through the active growth phases (G1, S, G2), leading to mitosis (M) in which the cell’s nucleus divides. For experiments, cells were incubated with test compounds for 24 h at concentrations equal to their previously calculated IC_50_ value. After this time, cells were harvested and treated with a buffer to lysate the cells and isolate the cell nuclei. Propidium iodide was used to assess the amount of DNA in particular phases of the cell cycle. The results of the distribution of the cell cycle phases after exposure to test compounds are shown in Figure 8. Histograms of DNA content in cells are presented in the Supplementary Materials (Figure S74).

The cytometric evaluation showed that the tested compounds did not significantly alter the cell cycle phase distribution of the HCT-116 line as compared to populations of untreated control. It cannot be ruled out that the incubation time of cells with solutions of potential drugs turned out to be too short and the extension of the experiment time would change the course of the cell cycle. In the MCF-7 cell line, an increase in the cells of the subG1 fraction was noted for glycoconjugates *type D*. The increase of the subG1 phase corresponds to the apoptotic and necrotic cell fractions. These results are consistent with cytotoxicity and apoptosis studies, in which trehalose derivatives showed the highest cytotoxicity within this cell line. Whereas for glycoconjugates of *type A* and *type E*, an increase in the number of cells in the G0/G1 phase was observed, this also indicates the cytostatic potential of these compounds. In the case of the NHDF-Neo cell line, compounds **A-1** and **A-2** slightly decreased the number of cells in the G2/M phase with a simultaneous slight increase in the percentage of cells in the G0/G1 phase. The other compounds changed the progression of the cell cycle oppositely. Namely, an increased G2/M fraction and a decreased G0/G1 fraction were observed for them. It follows that, under the influence of the tested compounds, the cell cycle was arrested and cell division was inhibited. Changes in this phase of the cycle may also result from the initiation of repair processes of the damaged cell. Moreover, for the healthy cell line, cell’s death and damage, presented as the subG1 phase increasing was noted for compound 8HQ, which is consistent with the results of the apoptosis assays.

#### 2.2.6. Intercalation Study

**Figure 9:**
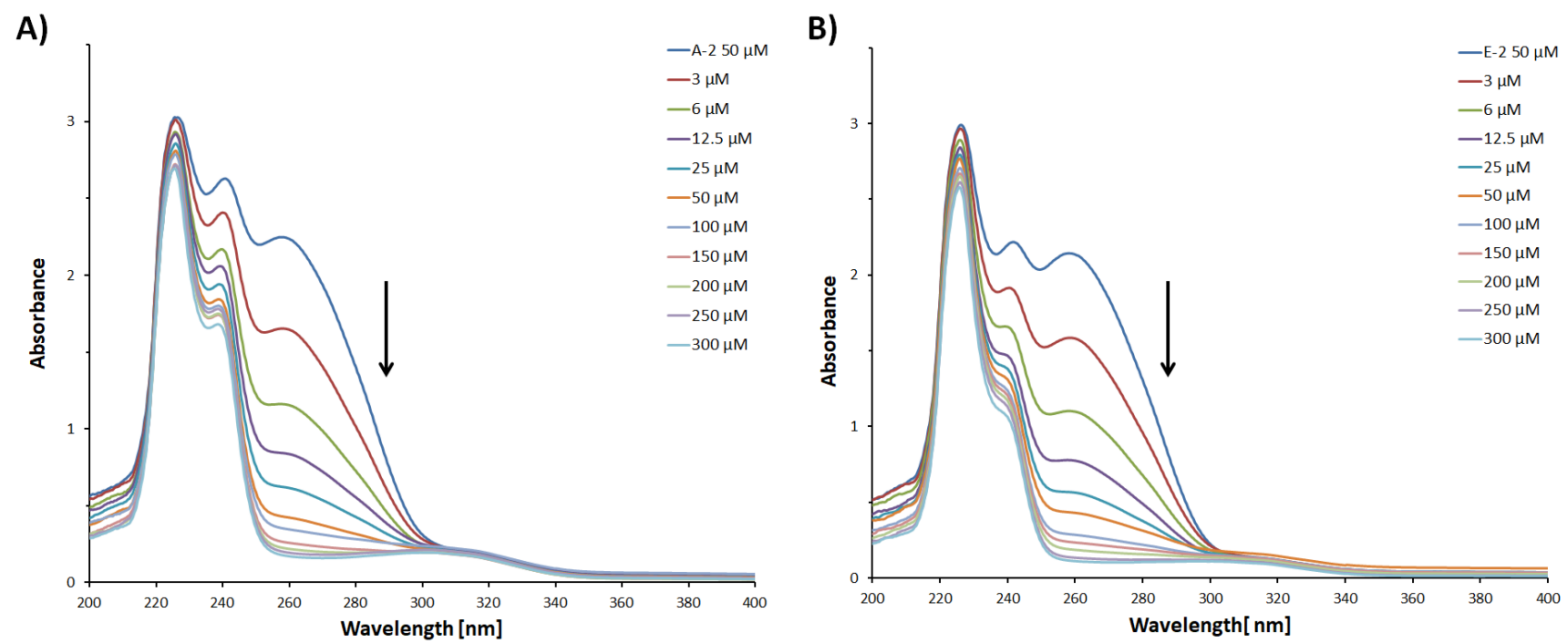
UV-Vis titrations of compounds: (**A**) **A-2** and (**B**) **E-2** with ctDNA.

Due to the steric and electron features, quinoline-based compounds are well-known DNA intercalating agents [63]. Therefore, the antiproliferative action of quinoline-related compounds can be related among others to intercalating between two adjacent base pairs of duplex DNA and inhibiting nucleic acid synthesis [64]. In order to evaluate the ability of DNA intercalation by synthesized glycoconjugates, we performed DNA-binding studies on the example of compounds **A-2** and **E-2**. The binding of the **A-2** and **E-2** to ctDNA has been characterized through absorption titration, which is an effective technique for DNA intercalation studies [65]. The experiments were carried out for a constant concentration of glycoconjugates with increasing concentrations of ctDNA. As shown in UV-Vis absorption spectrums (Figure 9) the glycoconjugates show the hypochromic and bathochromic effects that were observed upon binding to increasing concentrations of ctDNA. Both the hypochromic and bathochromic effects strongly suggest that synthesized glycoconjugates intercalate DNA, due to a strong interaction between the electronic states of the 8HQ scaffold and those of the DNA bases [66]. Accordingly introducing a sugar unit with a triazole linker does not interfere DNA intercalation ability of the 8HQ scaffold.

## 3. Materials and Methods

### 3.1. General Information

All used reagents and solvents were purchased from Sigma-Aldrich (Saint Louis, MO, USA), ACROS Organics (Geel, Belgium), and Avantor Performance Materials (Gliwice, Poland) and were used without further purification. All evaporations were performed on a rotary evaporator under reduced pressure at 40 °C. Thin layer chromatography (TLC) was used to monitor the progress of the reaction. The TLC plates precoated of silica gel 60 F254 (Merck Millipore, Burlington, MA, USA) were visualized under UV light (λ = 254 nm) or by charring after spraying with 10% solution of sulfuric acid in ethanol. Column chromatography was carried out on Silica Gel 60 (70–230 mesh, Fluka, St. Louis, MI, USA). NMR spectra were recorded on an Agilent spectrometer at a frequency of 400 MHz using CDCl_3_, DMSO-d6, or CD_3_OD as the solvents and tetramethylsilane (TMS) as an internal standard, which were purchased from ACROS Organics (Geel, Belgium). The chemical shifts (*δ*) values were reported in ppm and the coupling constants (*J*) in Hz. The following abbreviations were used to explain the observed multiplicities: s: singlet, bs: broad singlet, d: doublet, dd: doublet of doublets, ddd: doublet of doublet of doublets, t: triplet, dd~t: doublet of doublets resembling a triplet (with similar values of coupling constants), q: quintet, m: multiplet. High-resolution mass spectra (HRMS) were recorded on a WATERS LCT Premier XE system using the electrospray ionization (ESI) technique. Optical rotations were measured on a JASCO P-2000 polarimeter using a sodium lamp (λ = 589.3 nm) at room temperature. Melting points were determined on an OptiMelt (MPA 100) Stanford Research Systems. The absorbance on the MTT assay was measured spectrophotometrically at the 570 nm wavelength using a plate reader (Epoch, BioTek, Winooski, VT, USA).

8-(2-Propyn-1-yloxy)quinoline **24** [34], 8-(2-azidoethoxy)quinoline **25** [35], 8-(3-azidopropoxy)quinoline **26** [35] and 2-azidoethanol **28** [67] were synthesized according to the respective published procedures. d-Glucose **1**, d-galactose **7**, d-glucuronic acid **13**, trehalose **17**, propargyl alcohol **27**, 8-hydroxyquinoline (8HQ), and 4,6-*O*-ethylidene-α-d-glucose (EDG) are commercially available (Sigma-Aldrich).

### 3.2. Chemistry

#### 3.2.1. General Procedure for the Synthesis of Sugar Derivatives

*Synthesis of 1,2,3,4-Tetra-O-acetyl-6-O-triphenylmethyl-d-glucopyranose***2**: d-Glucose **1** (1.507 g, 8.365 mmol) was dissolved in pyridine (8 mL), and then trityl chloride (2.350 g, 8.430 mmol) and DMAP (0.180 g, 1.473 mmol) were added. The reaction mixture was stirred for 24 h at room temperature. After this time, acetyl chloride (4 mL) was added in portions to the reaction mixture and stirred for 1 h at room temperature. Then, the reaction diluted with dichloromethane (100 mL) and washed with brine (3 × 30 mL). The organic layer was dried over anhydrous MgSO_4_, filtered off, and the solvent was concentrated under reduced pressure. The crude product was purified by column chromatography (toluene/ethyl acetate; gradient 100:1 to 10:1) to give product **2** as a white solid (4.155 g, 84%); ratio of anomers (α:β = 1:1.3). ^1^H NMR (400 MHz, CDCl_3_): δ 1.73, 1.74, 2.00, 2.01, 2.03, 2.04, 2.15, 2.15 (8s, 24H, 4 × CH_3_-α, 4 × CH_3_-β), 3.02 (dd, 1H, *J* = 3.9 Hz, *J* = 10.7 Hz, H6a-α), 3.07 (dd, 1H, *J* = 4.2 Hz, *J* = 10.6 Hz, H6a-β), 3.32 (dd, 1H, *J* = 2.2 Hz, *J* = 10.7 Hz, H6b-α), 3.34 (dd, 1H, *J* = 2.5 Hz, *J* = 10.6 Hz, H6a-β), 3.69 (ddd, 1H, *J* = 2.5 Hz, *J* = 4.2 Hz, *J* = 9.8 Hz, H5-β), 4.02 (ddd, 1H, *J* = 2.2 Hz, *J* = 3.9 Hz, *J* = 10.2 Hz, H5-α), 5.14–5.29 (m, 4H, H2-α, H4-α, H2-β, H4-β), 5.31 (dd~t, 1H, *J* = 9.6 Hz, *J* = 10.1 Hz, H3-β), 5.41 (dd~t, 1H, *J* = 9.7 Hz, *J* = 10.0 Hz, H3-α), 5.72 (d, 1H, *J* = 8.0 Hz, H1-β), 6.44 (d, 1H, *J* = 3.7 Hz, H1-α), 7.12–7.46 (m, 30H, 3 × Ph-α, 3 × Ph-β); ^13^C NMR (100 MHz, CDCl_3_): δ 20.42, 20.45, 20.49, 20.60, 20.64, 20.74, 20.87, 20.93 (4 × CH_3_-α, 4 × CH_3_-β), 61.24, 61.69 (C6-α, C6-β), 68.29, 68.34, 69.50, 70.36, 70.56, 71.26, 73.19, 74.07 (C2-α, C3-α, C4-α, C5-α, C2-β, C3-β, C4-β, C5-β), 86.54, 86.66 (C-Ph_3_-α, C-Ph_3_-β), 89.36, 91.94 (C1-α, C1-β), 127.02, 127.02, 127.79, 127.81, 128.70, 128.72 (C2_Ph_-α, C3_Ph_-α, C4_Ph_-α, C2_Ph_-β, C3_Ph_-β, C4_Ph_-β), 143.46, 143.48 (C1_Ph_-α, C1_Ph_-β), 168.88, 168.89, 168.93, 168.97, 169.33, 169.76, 170.24, 170.43 (4 × CO-α, 4 × CO-β); HRMS (ESI-TOF): calcd for C_33_H_34_O_10_Na ([M + Na]^+^): *m*/*z* 613.2050; found: *m*/*z* 613.2050.
*Synthesis of 1,2,3,4-Tetra-O-acetyl-d-glucopyranose***3**: Compound **2** (1.251 g, 2.116 mmol) was suspended in glacial acetic acid (10 mL). The solution was cooled to 0 °C and 33% solution of hydrogen bromide in acetic acid (0.370 mL, 6.494 mmol) was added. Stirring was continued at room temperature for 1 h. After completion, the reaction was diluted with chloroform (50 mL) and water (50 mL). The aqueous phase was washed with chloroform (2 × 30 mL), and the combined organic phases were washed with NaHCO_3_ (2 × 50 mL) and brine (1 × 50 mL). Next, the organic layer was dried over anhydrous MgSO_4_, filtered off, and the solvent was concentrated under reduced pressure. The crude product was purified by column chromatography (toluene/ethyl acetate; gradient 20:1 to 1:1) to give product **3** as a yellow oil (0.597 g, 81%); ratio of anomers (α:β = 1:3.3). ^1^H NMR (400 MHz, CDCl_3_): δ 2.02, 2.03, 2.03, 2.04, 2.07, 2.08, 2.11, 2.18 (8s, 24H, 4 × CH_3_-α, 4 × CH_3_-β), 3.54–3.62 (m, 2H, H6a-α, H6a-β), 3.65 (ddd, 1H, *J* = 2.2 Hz, *J* = 4.2 Hz, *J* = 9.9 Hz, H5-β), 3.69–3.81 (m, 2H, H6b-α, H6b-β), 3.93 (ddd, 1H, *J* = 2.3 Hz, *J* = 4.0 Hz, *J* = 10.2 Hz, H5-α), 5.04–5.15 (m, 4H, H2-α, H4-α, H2-β, H4-β), 5.31 (dd~t, 1H, *J* = 9.5 Hz, *J* = 9.5 Hz, H3-β), 5.53 (dd~t, 1H, *J* = 9.9 Hz, *J* = 10.0 Hz, H3-α), 5.73 (d, 1H, *J* = 8.3 Hz, H1-β), 6.35 (d, 1H, *J* = 3.7 Hz, H1-α); ^13^C NMR (100 MHz, CDCl_3_): δ 20.46, 20.56, 20.60, 20.63, 20.69, 20.81, 20.89, 21.33 (4 × CH_3_-α, 4 × CH_3_-β), 60.85, 62.44 (C6-α, C6-β), 68.20, 68.31, 69.38, 69.60, 70.43, 72.05, 72.64, 74.92 (C2-α, C3-α, C4-α, C5-α, C2-β, C3-β, C4-β, C5-β), 89.12, 91.74 (C1-α, C1-β), 168.89, 168.98, 169.06, 169.26, 169.66, 170.09, 170.19, 170.26 (4 × CO-α, 4 × CO-β); HRMS (ESI-TOF): calcd for C_14_H_20_O_10_Na ([M + Na]^+^): *m*/*z* 371.0954; found: *m*/*z* 371.0951.
*Synthesis of 1,2,3,4-Tetra-O-acetyl-6-O-p-toluenesulfonyl-d-glucopyranose***4**: To a solution of **3** (0.338 g, 0.970 mmol) in pyridine (5 mL), *p*-toluenosulfonyl chloride (0.461 g, 2.418 mmol) and DMAP (23.8 mg, 0.195 mmol) were added. The reaction mixture was stirred at room temperature for 24 h. After completion, the reaction was diluted with water (50 mL) and washed with chloroform (3 × 50 mL). The organic layer was dried over anhydrous MgSO_4_, filtered off, and the solvent was concentrated under reduced pressure. The crude product was purified by column chromatography (toluene/ethyl acetate; gradient 30:1 to 2:1) to give product **4** as a white solid (0.352 g, 72%); ratio of anomers (α:β = 1:4). ^1^H NMR (400 MHz, CDCl_3_): δ 1.98, 1.99, 2.00, 2.01, 2.02, 2.02, 2.09, 2.16 (8s, 24H, 4 × CH_3_-α, 4 × CH_3_-β), 2.46 (s, 6H, Ar-CH_3_-α, Ar-CH_3_-β), 3.84 (m, 1H, H5-β), 4.04–5.25 (m, 5H, H5-α, H6a-α, H6b-α, H6a-β, H6b-β), 4.93–5.09 (m, 4H, H2-α, H4-α, H2-β, H4-β), 5.20 (dd~t, 1H, *J* = 9.4 Hz, *J* = 9.4 Hz, H3-β), 5.42 (dd~t, 1H, *J* = 9.6 Hz, *J* = 10.2 Hz, H3-α), 5.65 (d, 1H, *J* = 8.2 Hz, H1-β), 6.21 (d, 1H, *J* = 3.7 Hz, H1-α), 7.32–7.38 (m, 4H, Ar-H-α, Ar-H-β), 7.75–7.80 (m, 4H, Ar-H-α, Ar-H-β); ^13^C NMR (100 MHz, CDCl_3_): δ 20.40, 20.49, 20.52, 20.55, 20.58, 20.64, 20.74, 20.84 (4 × CH_3_-α, 4 × CH_3_-β), 21.68 (Ar-CH_3_-α, Ar-CH_3_-β), 66.74, 67.16, 67.95, 68.11, 69.00, 69.61, 70.05, 70.29, 72.17, 72.58 (C2-α, C3-α, C4-α, C5-α, C6-α, C2-β, C3-β, C4-β, C5-β, C6-β), 88.70, 91.53 (C1-α, C1-β), 128.16, 129.85, 132.43, 145.13 (Ar-α, Ar-β), 168.62, 168.74, 169.11, 169.26, 169.29, 169.55, 170.08, 170.20 (4 × CO-α, 4 × CO-β); HRMS (ESI-TOF): calcd for C_21_H_26_O_12_SNa ([M + Na]^+^): *m*/*z* 525.1043; found: *m*/*z* 525.1043.
*Synthesis of 1,2,3,4-Tetra-O-acetyl-6-azido-6-deoxy-d-glucopyranose***5**: To a solution of **4** (0.319 g, 0.635 mmol) in dry DMF (5 mL), sodium azide (0.208 g, 3.200 mmol) was added. The reaction mixture was stirred at 80 °C for 2 h. After this time, the solution was diluted with chloroform (50 mL) and water (50 mL). The aqueous phase was washed with chloroform (2 × 30 mL), and the combined organic phases were washed with brine (2 × 30 mL). Next, the organic layer was dried over anhydrous MgSO_4_, filtered off, and the solvent was concentrated at reduced pressure. The crude product was purified by column chromatography (toluene/ethyl acetate; gradient 30:1 to 10:1) to give product **5** as a white solid (0.216 g, 91%); ratio of anomers (α:β = 1.7:1). ^1^H NMR (400 MHz, CDCl_3_): δ 2.02, 2.02, 2.03, 2.04, 2.04, 2.05, 2.12, 2.19 (8s, 24H, 4 × CH_3_-α, 4 × CH_3_-β), 3.27–3.45 (m, 4H, H6a-α, H6b-α, H6a-β, H6b-β), 3.81 (ddd, 1H, *J* = 3.4 Hz, *J* = 5.2 Hz, *J* = 9.9 Hz, H5-β), 4.08 (ddd, 1H, *J* = 2.8 Hz, *J* = 5.5 Hz, *J* = 10.1 Hz, H5-α), 5.04–5.17 (m, 4H, H2-α, H4-α, H2-β, H4-β), 5.25 (dd~t, 1H, *J* = 9.4 Hz, *J* = 9.4 Hz, H3-β), 5.47 (dd~t, 1H, *J* = 9.7 Hz, *J* = 10.0 Hz, H3-α), 5.73 (d, 1H, *J* = 8.3 Hz, H1-β), 6.36 (d, 1H, *J* = 3.7 Hz, H1-α); ^13^C NMR (100 MHz, CDCl_3_): δ 20.44, 20.55, 20.57, 20.59, 20.66, 20.75, 20.82, 20.85 (4 × CH_3_-α, 4 × CH_3_-β), 50.65, 50.73 (C6-α, C6-β), 69.01, 69.07, 69.17, 69.68, 70.15, 70.92, 72.69, 73.84 (C2-α, C3-α, C4-α, C5-α, C2-β, C3-β, C4-β, C5-β), 88.88, 91.54 (C1-α, C1-β), 168.69, 168.93, 169.20, 169.42, 169.61, 169.93, 170.11, 170.24 (4 × CO-α, 4 × CO-β); HRMS (ESI-TOF): calcd for C_14_H_19_N_3_O_9_Na ([M + Na]^+^): *m*/*z* 396.1019; found: *m*/*z* 396.1022.
*Synthesis of 6-Azido-6-deoxy-d-glucopyranose***6**: Compound **5** (0.479 g, 1.283 mmol) was suspended in MeOH (10 mL), and then 1 M solution of MeONa in MeOH (1.3 mL, 1.3 mmol) was added. The reaction mixture was stirred for 0.5 h at room temperature. The reaction progress was monitored on TLC. After the reaction was complete, the mixture was neutralized with *Amberlyst*-15 ion exchange resin, filtered off, and the filtrate was concentrated under reduced pressure to give product **6** as a white solid (0.218 g, 83%); ratio of anomers (α:β = 1:1). ^1^H NMR (400 MHz, DMSO): δ 2.93 (dd, 1H, *J* = 7.7 Hz, *J* = 8.9 Hz, H2-β), 2.99–3.05 (m, 2H, H4-α, H4-β), 3.10–3.17 (m, 2H, H2-α, H3-β), 3.23–3.31 (m, 2H, H3-α, H5-β), 3.32–3.42 (m, 2H, H6a-α, H6a-β), 3.43–3.52 (m, 2H, H6b-α, H6b-β), 3.74 (ddd, 1H, *J* = 2.4 Hz, *J* = 6.4 Hz, *J* = 9.3 Hz, H5-α), 4.31 (d, 1H, *J* = 7.7 Hz, H1-β), 4.93 (d, 1H, *J* = 3.6 Hz, H1-α); ^13^C NMR (100 MHz, DMSO): δ51.60, 51.69 (C6-α, C6-β), 70.45, 70.93, 71.34, 72.22, 72.71, 74.60, 74.64, 76.34 (C2-α, C3-α, C4-α, C5-α, C2-β, C3-β, C4-β, C5-β), 92.43, 97.06 (C1-α, C1-β); HRMS (ESI-TOF): calcd for C_6_H_11_N_3_O_5_Na ([M + Na]^+^): *m*/*z* 228.0596; found: *m*/*z* 228.0604.
*Synthesis of 1,2:3,4-Di-O-isopropylidene-α-d-galactopyranose***8**: To a solution of d-galactose **7** (3.122 g, 17.329 mmol) in dry acetone (150 mL), iodine (0.923 g, 3.637 mmol) was added. The reaction mixture was stirred at room temperature for 24 h. After the completion reaction, 10% Na_2_S_2_O_3_ aqueous solution (18 mL) was added to the reaction mixture and then the solvent was evaporated under reduced pressure. The residue was diluted with dichloromethane (150 mL) and washed with brine (2 × 50 mL). The organic layer was dried over anhydrous MgSO_4_, filtered off, and the solvent was concentrated under reduced pressure. The crude product was purified by column chromatography (toluene/ethyl acetate; gradient 44:1 to 2:1) to give product **8** as a yellow oil (4.150 g, 92%). [α]^26^_D_ = −50.4 (c = 1.0, CHCl_3_); ^1^H NMR (400 MHz, CDCl_3_): δ 1.34, 1.34, 1.46, 1.54 (4s, 12H, 4 × CH_3_), 3.75 (m, 1H, H5), 3.83–3.91 (m, 2H, H6a, H6b), 4.28 (dd, 1H, *J* = 1.7 Hz, *J* = 7.9 Hz, H4), 4.34 (dd, 1H, *J* = 2.4 Hz, *J* = 5.0 Hz, H2), 4.62 (dd, 1H, *J* = 2.4 Hz, *J* = 7.9 Hz, H3), 5.57 (d, 1H, *J* = 5.0 Hz, H1); ^13^C NMR (100 MHz, CDCl_3_): δ 24.45, 25.08, 26.07, 26.18 (4xCH_3_), 62.49 (C6), 68.23, 70.73, 70.91, 71.76 (C2, C3, C4, C5), 96.44 (C1), 108.82, 109.62 (2 × C(CH_3_)_2_); HRMS (ESI-TOF): calcd for C_12_H_20_O_6_Na ([M + Na]^+^): *m*/*z* 283.1158; found: *m*/*z* 283.1162.
*Synthesis of 1,2:3,4-Di-O-isopropylidene-6-O-p-toluenesulfonyl-α-d-galactopyranose***9**: To a solution of compound **8** (1.324 g, 5.087 mmol) in pyridine (10 mL), *p*-toluenosulfonyl chloride (2.378 g, 12.473 mmol) and DMAP (0.120 g, 0.982 mmol) were added. The reaction mixture was stirred at room temperature for 24 h. After completion, the reaction was diluted with water (80 mL) and washed with chloroform (3 × 80 mL). The organic layer was dried over anhydrous MgSO_4_, filtered off, and the solvent was concentrated under reduced pressure. The crude product was purified by column chromatography (toluene/ethyl acetate; gradient 100:1 to 5:1) to give product **9** as a yellow oil (1.886 g, 90%). [α]^26^_D_ = −24.4 (c = 1.0, CHCl_3_); ^1^H NMR (400 MHz, CDCl_3_): δ 1.28, 1.32, 1.35, 1.50 (4s, 12H, 4 × CH_3_), 2.44 (s, 3H, Ar-CH_3_), 4.02–4.13 (m, 2H, H6a, H6b), 4.17–4.23 (m, 2H, H4, H5), 4.29 (dd, 1H, *J* = 2.5 Hz, *J* = 5.0 Hz, H2), 4.59 (dd, 1H, *J* = 2.5 Hz, *J* = 7.9 Hz, H3), 5.46 (d, 1H, *J* = 5.0 Hz, H1), 7.33 (d, 2H, *J* = 8.2 Hz, Ar-H), 7.81 (d, 2H, *J* = 8.2 Hz, Ar-H); ^13^C NMR (100 MHz, CDCl_3_): δ 21.63 (Ar-CH_3_), 24.36, 24.92, 25.82, 25.99 (4 × CH_3_), 65.88 (C6), 68.20, 70.38, 70.42, 70.53 (C2, C3, C4, C5), 96.13 (C1), 108.95, 109.59 (2 × C(CH_3_)_2_), 128.12, 129.74, 132.84, 144.75 (Ar); HRMS (ESI-TOF): calcd for C_19_H_26_O_8_SNa ([M + Na]^+^): *m*/*z* 437.1246; found: *m*/*z* 473.1248.
*Synthesis of 1,2:3,4-Di-O-isopropylidene-6-azido-6-deoxy-α-d-galactopyranose***10**: To a solution of compound **9** (0.798 g, 1.925 mmol) in dry DMF (7 mL), sodium azide (0.507 g, 7.799 mmol) was added. The reaction mixture was stirred at 120 °C for 24 h. After this time, the reaction was diluted with chloroform (30 mL) and water (30 mL). The aqueous phase was washed with chloroform (2 × 30 mL), and the combined organic phases were washed with water (1 × 30 mL) and brine (1 × 30 mL). Next, the organic layer was dried over anhydrous MgSO_4_, filtered off, and the solvent was concentrated under reduced pressure. The crude product was purified by column chromatography (toluene/ethyl acetate; gradient 30:1 to 10:1) to give product **10** as a colorless oil (0.432 g, 79%). [α]^24^_D_ = −104.2 (c = 1.0, CHCl_3_); ^1^H NMR (400 MHz, CDCl_3_): δ 1.34, 1.35, 1.46, 1.55 (4s, 12H, 4 × CH_3_), 3.37 (dd, 1H, *J* = 5.4 Hz, *J* = 12.7 Hz, H6a), 3.52 (dd, 1H, *J* = 7.8 Hz, *J* = 12.7 Hz, H6b), 3.92 (ddd, 1H, *J* = 2.0 Hz, *J* = 5.4 Hz, *J* = 7.8 Hz, H5), 4.20 (dd, 1H, *J* = 2.0 Hz, *J* = 7.9 Hz, H4), 4.34 (dd, 1H, *J* = 2.5 Hz, *J* = 5.0 Hz, H2), 4.63 (dd, 1H, *J* = 2.5 Hz, *J* = 7.9 Hz, H3), 5.55 (d, 1H, *J* = 5.0 Hz, H1); ^13^C NMR (100 MHz, CDCl_3_): δ 24.44, 24.89, 25.95, 26.03 (4 × CH_3_), 50.68 (C6), 67.00, 70.40, 70.81, 71.17 (C2, C3, C4, C5), 96.35 (C1), 108.81, 109.63 (2 × C(CH_3_)_2_); HRMS (ESI-TOF): calcd for C_12_H_19_N_3_O_5_Na ([M + Na]^+^): *m*/*z* 308.1222; found: *m*/*z* 308.1224.
*Synthesis of 6-Azido-6-deoxy-d-galactopyranose***11**: Compound **10** (0.410 g, 1.437 mmol) was suspended in water (0.9 mL). The solution was cooled to 0 °C and trifluoroacetic acid (3.5 mL) was added in portions. Stirring was continued at room temperature for 2 h. After completion, the reaction mixture was concentrated under reduced pressure with the addition of *i*-propanol. Next, the residue was purified by column chromatography (dry loading: chloroform/methanol; gradient 50:1 to 5:1) to give product **11** as a white solid (0.193 g, 65%); ratio of anomers (α:β = 6.3:1). ^1^H NMR (400 MHz, DMSO): δ 3.17 (m, 1H, H6a-β), 3.28 (dd, 1H, *J* = 4.3 Hz, *J* = 12.7 Hz, H6a-α), 3.37–3.47 (m, 2H, H6b-α, H6b-β), 3.48–3.59 (m, 4H, H2-α, H2-β, H3-α, H3-β), 3.61 (m, 1H, H5-α), 3.68 (m, 1H, H5-β), 3.95 (dd, 1H, *J* = 4.2 Hz, *J* = 8.6 Hz, H4-α), 4.09 (dd, 1H, *J* = 5.2 Hz, *J* = 10.5 Hz, H4-β), 4.26 (t, 1H, *J* = 7.1 Hz, OH-β), 4.33 (d, 1H, *J* = 6.6 Hz, OH-α), 4.53 (d, 1H, *J* = 5.2 Hz, OH-α), 4.54 (d, 1H, *J* = 6.7 Hz, OH-α), 4.58 (d, 1H, *J* = 4.5 Hz, OH-β), 4.71 (d, 1H, *J* = 5.2 Hz, OH-β), 4.75 (d, 1H, *J* = 4.3 Hz, OH-β), 4.95 (t, 1H, *J* = 4.1 Hz, OH-α), 6.29 (d, 1H, *J* = 4.8 Hz, H1-α), 6.62 (d, 1H, *J* = 6.9 Hz, H1-β); ^13^C NMR (100 MHz, DMSO): δ 51.36, 51.46 (C6-α, C6-β), 68.40, 68.83, 68.97, 69.60 (C2-α, C3-α, C4-α, C5-α), 71.72, 71.74, 72.97, 73.08 (C2-β, C3-β, C4-β, C5-β), 92.71 (C1-α), 97.42 (C1-β); HRMS (ESI-TOF): calcd for C_6_H_11_N_3_O_5_Na ([M + Na]^+^): *m*/*z* 228.0596; found: *m*/*z* 228.0595.
*Synthesis of 1,2,3,4-Tetra-O-acetyl-6-azido-6-deoxy-d-galactopyranose***12**: Unprotected compound **11** (0.308 g, 1.501 mmol) was suspended in acetic anhydride (5 mL) and sodium acetate (0.286 g, 3.487 mmol) was added. The reaction mixture was heated to reflux for 1 h. After completion, the reaction was diluted with water (30 mL) and chloroform (30 mL). The aqueous phase was washed with chloroform (3 × 30 mL), and the combined organic phases were washed with NaHCO_3_ (1 × 30 mL) and brine (1 × 30 mL). Next, the organic layer was dried over anhydrous MgSO_4_, filtered off, and the solvent was concentrated under reduced pressure. The crude product was purified by column chromatography (toluene/ethyl acetate; gradient 30:1 to 10:1) to give product **12** as a colorless oil (0.377 g, 67%); ratio of anomers (α:β = 1:2.6). ^1^H NMR (400 MHz, CDCl_3_): δ 2.00, 2.05, 2.12, 2.19 (4s, 12H, 4xCH_3_-β), 2.01, 2.02, 2.17, 2.18 (4s, 12H, 4 × CH_3_-α), 3.22 (dd, 1H, *J* = 5.4 Hz, *J* = 12.8 Hz, H6a-β), 3.23 (dd, 1H, *J* = 5.5 Hz, *J* = 12.8 Hz, H6a-α), 3.45 (dd, 1H, *J* = 7.4 Hz, *J* = 12.8 Hz, H6b-α), 3.53 (dd, 1H, *J* = 7.4 Hz, *J* = 12.8 Hz, H6b-β), 3.95 (m, 1H, H5-β), 4.23 (m, 1H, H5-α), 5.08 (dd, 1H, *J* = 3.4 Hz, *J* = 10.4 Hz, H3-β), 5.30–5.37 (m, 3H, H2-β, H2-α, H3-α), 5.41 (dd, 1H, *J* = 1.1 Hz, *J* = 3.4 Hz, H4-β), 5.48 (m, 1H, H4-α), 5.72 (d, 1H, *J* = 8.3 Hz, H1-β), 6.40 (bs, 1H, H1-α); ^13^C NMR (100 MHz, CDCl_3_): δ 20.52, 20.53, 20.61, 20.63, 20.65, 20.68, 20.76, 20.88 (4 × CH_3_-α, 4 × CH_3_-β), 50.08, 50.32 (C6-α, C6-β), 66.36, 67.37, 67.52, 67.69, 68.14, 70.13, 70.83, 73.20 (C2-α, C3-α, C4-α, C5-α, C2-β, C3-β, C4-β, C5-β), 89.63, 92.13 (C1-α, C1-β), 168.86, 168.88, 169.36, 169.39, 169.87, 169.88, 170.05, 170.08 (4 × CO-α, 4 × CO-β); HRMS (ESI-TOF): calcd for C_14_H_19_N_3_O_9_Na ([M + Na]^+^): *m*/*z* 396.1019; found: *m*/*z* 396.1020.
*Synthesis of 1,2,3,4-Tetra-O-acetyl-β-d-glucopyranuronic acetic anhydride***14**: d-Glucuronic acid **13** (2.003 g, 10.317 mmol) was suspended in acetic anhydride (30 mL). The solution was cooled to 0 °C and iodine (150.0 mg, 0.591 mmol) was added. Stirring was continued at 0 °C for 1 h and further at room temperature for 3 h. After the completion reaction, acetic anhydride was removed under reduced pressure, and the residue was diluted with dichloromethane (40 mL) and washed with 1M Na_2_S_2_O_3_ (2 × 40 mL). The organic layer was dried over anhydrous MgSO_4_, filtered off, and the solvent was concentrated under reduced pressure and recrystallized from dichloromethane/hexane to give product **14** as a white solid (3.511 g, 84%). ^1^H NMR (400 MHz, CDCl_3_): δ 2.04, 2.06, 2.07, 2.13, 2.28 (5s, 15H, 5 × CH_3_), 4.34 (d, 1H, *J* = 9.2 Hz, H5), 5.13 (dd, 1H, *J* = 6.9 Hz, *J* = 8.6 Hz, H2), 5.30 (dd~t, 1H, *J* = 8.6 Hz, *J* = 9.1 Hz, H3), 5.38 (dd~t, 1H, *J* = 9.1 Hz, *J* = 9.2 Hz, H4), 5.82 (d, 1H, *J* = 6.9 Hz, H1); ^13^C NMR (100 MHz, CDCl_3_): δ 20.58, 20.64, 20.64, 20.81, 22.19 (5 × CH_3_), 68.20, 70.19, 71.44, 73.13 (C2, C3, C4, C5), 91.49 (C1), 162.69 (C6), 164.87, 168.80, 169.33, 169.48, 169.97 (5 × COCH_3_); HRMS (ESI-TOF): calcd for C_16_H_20_O_12_Na ([M + Na]^+^): *m*/*z* 427.0852; found: *m*/*z* 427.0851.
*Synthesis of 1,2,3,4-Tetra-O-acetyl-N-(prop-2-yn-1-yl)-β-d-glucopyranuronic acid amide***15**: To a solution of anhydride **14** (1.7 g, 4.205 mmol) in dry dichloromethane (20 mL), propargyl amine (400 µL, 6.245 mmol) was added. The reaction mixture was stirred overnight at room temperature. Afterward, the solvent was evaporated under reduced pressure, and the residue was purified by column chromatography (toluene/ethyl acetate; gradient 50:1 to 5:1) to give product **15** as a white solid (0.745 g, 44%). ^1^H NMR (400 MHz, CDCl_3_): δ 2.03, 2.05, 2.08, 2.15 (4s, 12H, 4 × CH_3_), 2.26 (t, 1H, *J* = 2.6 Hz, CCH), 4.03 (m, 2H, CH_2_), 4.12 (d, 1H, *J* = 9.6 Hz, H5), 5.11 (dd, 1H, *J* = 7.9 Hz, *J* = 8.9 Hz, H2), 5.22 (dd~t, 1H, *J* = 9.2 Hz, *J* = 9.6 Hz, H4), 5.31 (dd~t, 1H, *J* = 8.9 Hz, *J* = 9.2 Hz, H3), 5.77 (d, 1H, *J* = 7.9 Hz, H1), 6.50 (t, 1H, *J* = 5.1 Hz, NH); ^13^C NMR (100 MHz, CDCl_3_): δ 20.54, 20.54, 20.68, 20.78 (4 × CH_3_), 28.92 (CH_2_), 68.75, 70.23, 71.94, 72.12 (C2, C3, C4, C5), 72.88 (CCH), 78.62 (CCH), 91.40 (C1), 165.71 (CONH), 168.78, 169.23, 169.61, 169.81 (4 × COCH_3_); HRMS (ESI-TOF): calcd for C_17_H_21_NO_10_Na ([M + Na]^+^): *m*/*z* 422.1063; found: *m*/*z* 422.1064.
*Synthesis of N-(prop-2-yn-1-yl)-d-glucopyranuronic acid amide***16**: Acetylated amide **15** (0.526 g, 1.317 mmol) was suspended in MeOH (20 mL), and then 1 M solution of MeONa in MeOH (1.3 mL, 1.3 mmol) was added. The reaction mixture was stirred for 0.5 h at room temperature. The reaction progress was monitored on TLC. After the reaction was complete, the mixture was neutralized with *Amberlyst*-15 ion exchange resin, filtered off, and the filtrate was concentrated under reduced pressure to give product **16** as a white solid (0.301 g, 99%); ratio of anomers (α:β = 1.3:1). ^1^H NMR (400 MHz, CD_3_OD): δ 2.55–2.60 (m, 2H, CCH-α, CCH-β), 3.18 (dd, 1H, *J* = 7.8 Hz, *J* = 9.2 Hz, H2-β), 3.36–3.52 (m, 4H, H2-α, H3-β, H4-α, H4-β), 3.69 (d, 1H, *J* = 9.7 Hz, H5-β), 3.72 (dd~t, 1H, *J* = 9.2 Hz, *J* = 9.6 Hz, H3-α), 3.98–4.02 (m, 4H, CH_2_-α, CH_2_-β), 4.18 (d, 1H, *J* = 9.9 Hz, H5-α), 4.53 (d, 1H, *J* = 7.8 Hz, H1-β), 5.18 (d, 1H, *J* = 3.7 Hz, H1-α), 8.52 (s, 2H, NH-α, NH-β); ^13^C NMR (100 MHz, CD_3_OD): δ 29.17, 29.24 (CH_2_-α, CH_2_-β), 71.91, 72.19, 73.30, 73.50, 73.98, 74.41, 75.80, 76.29 (C2-α, C3-α, C4-α, C5-α, C2-β, C3-β, C4-β, C5-β), 72.13, 72.33 (CCH-α, CCH-β), 80.32, 80.43 (CCH-α, CCH-β), 94.27, 98.52 (C1-α, C1-β), 169.97, 171.69 (CONH-α, CONH-β); HRMS (ESI-TOF): calcd for C_9_H_13_NO_6_Na ([M + Na]^+^): *m*/*z* 254.0641; found: *m*/*z* 254.0641.
*Synthesis of 6,6′-Dibromo-6,6′-dideoxy-d-trehalose***18**: To a solution of d-trehalose **17** (1.008 g, 2.945 mmol) in dry DMF (10 mL), triphenylphosphine (3.325 g, 12.677 mmol) and NBS (2.268 g, 12.742 mmol) were added. The reaction mixture was stirred at room temperature for 72 h. Afterward, the solvent was evaporated under reduced pressure, and the residue was purified by column chromatography (chloroform/methanol; gradient 50:1 to 1:1) to give product **18** as a white solid (1.368 g, 99%). m.p.: 131–132 °C; [α]^24^_D_ = 109.0 (c = 1.0, DMSO); ^1^H NMR (400 MHz, DMSO): δ 3.11 (dd~t, 2H, *J* = 9.1 Hz, *J* = 9.2 Hz, 2 × H4), 3.27 (dd, 2H, *J* = 3.6 Hz, *J* = 9.5 Hz, 2 × H2), 3.52–3.63 (m, 4H, 2 × H3, 2 × H6a), 3.69 (dd, 2H, *J* = 2.3 Hz, *J* = 10.8 Hz, 2 × H6b), 3.87 (ddd, 2H, *J* = 2.3 Hz, *J* = 5.5 Hz, *J* = 9.3 Hz, 2 × H5), 4.92 (d, 2H, *J* = 3.6 Hz, 2 × H1); ^13^C NMR (100 MHz, DMSO): δ 35.57 (2 × C6), 70.35, 71.35, 72.02, 72.41 (2 × C2, 2 × C3, 2 × C4, 2 × C5), 93.40 (2 × C1); HRMS (ESI-TOF): calcd for C_12_H_20_Br_2_O_9_Na ([M + Na]^+^): *m*/*z* 488.9372; found: *m*/*z* 488.9367.
*Synthesis of 6,6′-Diiodo-6,6′-dideoxy-d-trehalose***19**: To a solution of d-trehalose **17** (1.018 g, 2.974 mmol) in dry DMF (10 mL), triphenylphosphine (3.281 g, 12.509 mmol) and iodine (3.224 g, 12.702 mmol) were added. The reaction mixture was stirred at room temperature for 72 h. Afterward, the solvent was evaporated under reduced pressure, and the residue was purified by column chromatography (chloroform/methanol; gradient 50:1 to 1:1) to give product **19** as a white solid (1.588 g, 95%). m.p.: 62–63 °C; [α]^24^_D_ = 95.0 (c = 1.0, DMSO); ^1^H NMR (400 MHz, DMSO): δ 3.00 (m, 2H, 2 × H4), 3.26–3.35 (m, 4H, 2 × H2, 2 × H6a), 3.45–3.52 (m, 4H, 2 × H3, 2 × H6b), 3.59 (m, 2H, 2 × H5), 4.81 (d, 2H, *J* = 6.2 Hz, 2 × OH), 4.91 (m, 2H, 2 × OH), 4.96 (d, 2H, *J* = 3.7 Hz, 2 × H1), 5.16 (d, 2H, *J* = 5.4 Hz, 2 × OH); ^13^C NMR (100 MHz, DMSO): δ 10.59 (2 × C6), 69.92, 71.39, 72.12, 73.96 (2 × C2, 2 × C3, 2 × C4, 2 × C5), 93.19 (2 × C1); HRMS (ESI-TOF): calcd for C_12_H_20_I_2_O_9_Na ([M + Na]^+^): *m*/*z* 584.9094; found: *m*/*z* 584.9090.
*Synthesis of 6,6′-Diazido-6,6′-dideoxy-d-trehalose***20***(Procedure A)*: To a solution of **18** (1.234 g, 2.637 mmol) in dry DMF (20 mL), sodium azide (1.030 g, 15.844 mmol) was added. The reaction mixture was stirred at 60 °C for 24 h. After completion reaction, the residual salt was filtered off and the solvent was concentrated under reduced pressure. The residue was purified by column chromatography (chloroform/methanol; gradient 20:1 to 1:1) to give product **20** as a white solid (0.568 g, 55%).
*Synthesis of 6,6′-Diazido-6,6′-dideoxy-d-trehalose***20***(Procedure B)*: Compound **23** (0.202 g, 0.313 mmol) was suspended in MeOH (10 mL), and then 1 M solution of MeONa in MeOH (0.5 mL, 0.5 mmol) was added. The reaction mixture was stirred for 1 h at room temperature. The reaction progress was monitored on TLC. After the reaction was complete, the mixture was neutralized with *Amberlyst*-15 ion exchange resin, filtered off, and the filtrate was concentrated under reduced pressure to give product **20b** as a white solid (0.107 g, 87%). m.p.: 144 °C; [α]^24^_D_ = 47.0 (c = 1.0, DMSO); ^1^H NMR (400 MHz, DMSO): δ 3.10 (m, 2H, 2 × H4), 3.29 (m, 2H, 2 × H2), 3.39 (m, 4H, 2 × H6a, 2 × H6b), 3.54 (m, 2H, 2 × H3), 3.95 (m, 2H, 2 × H5), 4.86–4.93 (m, 6H, 2 × H1, 4 × OH), 5.12 (d, 2H, *J* = 5.3 Hz, 2 × OH); ^13^C NMR (100 MHz, DMSO): δ 51.26 (2 × C6), 70.95, 71.30, 71.37, 72.46 (2 × C2, 2 × C3, 2 × C4, 2 × C5), 93.91 (2 × C1); HRMS (ESI-TOF): calcd for C_12_H_20_N_6_O_9_Na ([M + Na]^+^): *m*/*z* 415.1189; found: *m*/*z* 415.1193.
*Synthesis of 2,3,4,2′,3′,4′-Hexa-O-acetyl-6,6′-dibromo-6,6′-dideoxy-d-trehalose***21**: Compound **18** (1 g, 2.136 mmol) was dissolved in pyridine (3 mL), the solution was cooled to 0 °C and Ac_2_O (3 mL) was added. Stirring was continued overnight at room temperature. After completion, the reaction solution was poured into ice water. The precipitated crystals are filtered off under reduced pressure and recrystallized from methanol to give product **21** as a white solid (1.072 g, 70%). m.p.: 161 °C; [α]^24^_D_ = 114.0 (c = 1.0, CHCl_3_); ^1^H NMR (400 MHz, CDCl_3_): δ 2.03, 2.08, 2.13 (3s, 18H, 6 × CH_3_), 3.32 (dd, 2H, *J* = 7.8 Hz, *J* = 11.2 Hz, 2 × H6a), 3.39 (dd, 2H, *J* = 2.6 Hz, *J* = 11.2 Hz, 2 × H6b), 4.12 (ddd, 2H, *J* = 2.6 Hz, *J* = 7.8 Hz, *J* = 10.1 Hz, 2 × H5), 4.97 (dd, 2H, *J* = 9.2 Hz, *J* = 10.1 Hz, 2 × H4), 5.16 (dd, 2H, *J* = 3.9 Hz, *J* = 10.2 Hz, 2 × H2), 5.38 (d, 2H, *J* = 3.9 Hz, 2 × H1), 5.49 (dd, 2H, *J* = 9.2 Hz, *J* = 10.2 Hz, 2 × H3); ^13^C NMR (100 MHz, CDCl_3_): δ 20.66, 20.68, 20.94 (6 × CH_3_), 30.40 (2 × C6), 69.36, 69.80, 69.96, 71.23 (2 × C2, 2 × C3, 2 × C4, 2 × C5), 91.91 (2 × C1), 169.47, 169.57, 169.92 (6 × CO); HRMS (ESI-TOF): calcd for C_24_H_32_Br_2_O_15_Na ([M + Na]^+^): *m*/*z* 741.0006; found: *m*/*z* 741.0008.
*Synthesis of 2,3,4,2′,3′,4′-Hexa-O-acetyl-6,6′-diiodo-6,6′-dideoxy-d-trehalose***22**: Compound **19** (1.646 g, 2.928 mmol) was dissolved in pyridine (5 mL), the solution was cooled to 0 °C and Ac_2_O (5 mL) was added. Stirring was continued overnight at room temperature. After completion, the reaction solution was poured into ice water. The precipitated crystals are filtered off under reduced pressure and recrystallized from methanol to give product **22** as a white solid (1.788 g, 75%). m.p.: 190 °C; [α]^23^_D_ = 84.2 (c = 1.0, CHCl_3_); ^1^H NMR (400 MHz, CDCl_3_): δ 2.02, 2.08, 2.15 (3s, 18H, 6 × CH_3_), 3.07 (dd, 2H, *J* = 9.0 Hz, *J* = 10.9 Hz, 2 × H6a), 3.23 (dd, 2H, *J* = 2.5 Hz, *J* = 10.9 Hz, 2 × H6b), 3.96 (ddd, 2H, *J* = 2.5 Hz, *J* = 9.0 Hz, *J* = 9.8 Hz, 2 × H5), 4.90 (dd, 2H, *J* = 9.2 Hz, *J* = 9.8 Hz, 2 × H4), 5.20 (dd, 2H, *J* = 3.9 Hz, *J* = 10.2 Hz, 2 × H2), 5.42 (d, 2H, *J* = 3.9 Hz, 2 × H1), 5.48 (dd, 2H, *J* = 9.2 Hz, *J* = 10.2 Hz, 2 × H3); ^13^C NMR (100 MHz, CDCl_3_): δ 2.49 (2 × C6), 20.64, 20.70, 21.20 (6 × CH_3_), 69.30, 69.75, 69.96, 72.34 (2 × C2, 2 × C3, 2 × C4, 2 × C5), 91.78 (2 × C1), 169.45, 169.58, 169.91 (6 × CO); HRMS (ESI-TOF): calcd for C_24_H_32_I_2_O_15_Na ([M + Na]^+^): *m*/*z* 836.9728; found: *m*/*z* 836.9733.
*Synthesis of 2,3,4,2′,3′,4′-Hexa-O-acetyl-6,6′-diazido-6,6′-dideoxy-d-trehalose***23**: To a solution of **21** or **22** (0.484 g, 0.672 mmol) in dry DMF (10 mL), sodium azide (0.273 g, 4.199 mmol) was added. The reaction mixture was stirred at 60 °C for 24 h. After completion, the reaction was diluted with ethyl acetate (30 mL) and water (30 mL). The aqueous phase was washed with ethyl acetate (2 × 30 mL), and the combined organic phases were washed with water (1 × 30 mL) and brine (1 × 30 mL). Next, the organic layer was dried over anhydrous MgSO_4_, filtered, and the solvent was concentrated under reduced pressure. The crude product was purified by column chromatography (toluene/ethyl acetate; gradient 20:1 to 1:1) to give product **23** as a white solid (0.425 g, 98%). m.p.: 150–151 °C; [α]^24^_D_ = 39.5 (c = 1.0, CHCl_3_); ^1^H NMR (400 MHz, CDCl_3_): δ 2.03, 2.07, 2.13 (3s, 18H, 6 × CH_3_), 3.17 (dd, 2H, *J* = 2.5 Hz, *J* = 13.3 Hz, 2 × H6a), 3.37 (dd, 2H, *J* = 7.3 Hz, *J* = 13.3 Hz, 2 × H6b), 4.09 (ddd, 2H, *J* = 2.5 Hz, *J* = 7.3 Hz, *J* = 10.2 Hz, 2 × H5), 5.00 (dd, 2H, *J* = 9.3 Hz, *J* = 10.2 Hz, 2 × H4), 5.09 (dd, 2H, *J* = 3.9 Hz, *J* = 10.3 Hz, 2 × H2), 5.34 (d, 2H, *J* = 3.9 Hz, 2 × H1), 5.48 (dd, 2H, *J* = 9.3 Hz, *J* = 10.3 Hz, 2 × H3); ^13^C NMR (100 MHz, CDCl_3_): δ 20.64, 20.66, 20.67 (6 × CH_3_), 50.99 (2 × C6), 69.67, 69.72, 69.83, 69.92 (2 × C2, 2 × C3, 2 × C4, 2 × C5), 93.01 (2 × C1), 169.62, 169.65, 169.96 (6 × CO); HRMS (ESI-TOF): calcd for C_24_H_32_N_6_O_15_Na ([M + Na]^+^): *m*/*z* 667.1823; found: *m*/*z* 667.1819.

#### 3.2.2. General Procedure for the Synthesis of Glycoconjugates

##### Synthesis of Glycoconjugates *Type A*

The 1,2,3,4-tetra-*O*-acetyl-6-azido-6-deoxy-d-glucopyranose **5** (0.118 g, 0.316 mmol) or 6-azido-6-deoxy-d-glucopyranose **6** (0.100 g, 0.487 mmol) and 8-(2-propyn-1-yloxy)quinoline **24** (1 equiv) were dissolved in *i*-PrOH (3 mL) and THF (3 mL). Then, sodium ascorbate (0.4 equiv) dissolved in H_2_O (1.5 mL) and CuSO_4_·5H_2_O (0.2 equiv) dissolved in H_2_O (1.5 mL), mixed together and added to the obtained solution. The reaction mixture was stirred overnight at room temperature. The reaction progress was monitored on TLC in an eluent system CHCl_3_:MeOH (10:1 for protected or 2:1 for unprotected compounds). After completion of the reaction, the precipitate of inorganic salts was filtered off, and the filtrate was concentrated under reduced pressure. The crude product was purified by column chromatography (toluene:ethyl acetate, 2:1 and chloroform:methanol, 70:1 for fully protected glycoconjugates or chloroform:methanol, gradient: 50:1 to 10:1 for unprotected glycoconjugates).

- Glycoconjugate **A-1**

The product was obtained as a green solid (0.163 g, 93%); ratio of anomers (α:β = 1:1.4); ^1^H NMR (400 MHz, CDCl_3_): δ 1.99, 2.00, 2.01, 2.02, 2.04, 2.05, 2.09, 2.09 (8s, 24H, 4 × CH_3_-α, 4 × CH_3_-β), 4.00 (m, 1H, H5-β), 4.32 (m, 1H, H5-α), 4.36–4.44 (m, 2H, H6a-α, H6a-β), 4.51–4.64 (m, 2H, H6b-α, H6b-β), 4.82 (dd~t, 1H, *J* = 9.4 Hz, *J* = 10.0 Hz, H4-β), 4.83 (dd~t, 1H, *J* = 9.4 Hz, *J* = 10.1 Hz, H4-α), 4.99 (dd, 1H, *J* = 3.7 Hz, *J* = 10.2 Hz, H2-α), 5.06 (dd, 1H, *J* = 8.3 Hz, *J* = 9.5 Hz, H2-β), 5.23 (dd~t, 1H, *J* = 9.4 Hz, *J* = 9.5 Hz, H3-β), 5.45 (dd, 1H, *J* = 9.4 Hz, *J* = 10.2 Hz, H3-α), 5.56, 5.58 (2s, 4H, CH_2_O-α, CH_2_O-β), 5.59 (d, 1H, *J* = 8.3 Hz, H1-β), 6.24 (d, 1H, *J* = 3.7 Hz, H1-α), 7.28–7.35 (m, 2H, H7_quin_-α, H7_quin_-β), 7.37–7.48 (m, 6H, H3_quin_-α, H5_quin_-α, H6_quin_-α, H3_quin_-β, H5_quin_-β, H6_quin_-β), 7.81, 7.83 (2s, 2H, H5_triaz_-α, H5_triaz_-β), 8.10–8.16 (m, 2H, H4_quin_-α, H4_quin_-β), 8.91–8.96 (m, 2H, H2_quin_-α, H2_quin_-β); ^13^C NMR (100 MHz, CDCl_3_): δ 19.73, 20.39, 20.51, 20.52, 20.63, 20.70, 20.95, 21.05 (4 × CH_3_-α, 4 × CH_3_-β), 50.44, 50.55 (C6-α, C6-β), 62.78, 62.83 (CH_2_O-α, CH_2_O-β), 68.97, 69.04, 69.27, 69.51, 69.99, 70.27, 72.45, 73.15 (C2-α, C3-α, C4-α, C5-α, C2-β, C3-β, C4-β, C5-β), 88.62, 91.61 (C1-α, C1-β), 110.06, 110.06 (C7_quin_-α, C7_quin_-β), 120.16, 120.28 (C5_quin_-α, C5_quin_-β), 121.57, 121.64 (C3_quin_-α, C3_quin_-β), 124.68, 124.81 (C5_triaz_-α, C5_triaz_-β), 126.68, 126.79 (C6_quin_-α, C6_quin_-β), 129.49, 129.57 (C4a_quin_-α, C4a_quin_-β), 135.95, 136.49 (C4_quin_-α, C4_quin_-β), 140.39, 140.39 (C8a_quin_-α, C8a_quin_-β), 144.37, 144.50 (C4_triaz_-α, C4_triaz_-β), 149.29, 149.38 (C2_quin_-α, C2_quin_-β), 153.84, 153.85 (C8_quin_-α, C8_quin_-β), 168.46, 168.62, 169.10, 169.45, 169.49, 169.55, 169.96, 170.09 (4 × CO-α, 4 × CO-β); HRMS (ESI-TOF): calcd for C_26_H_29_N_4_O_10_ ([M + H]^+^): *m*/*z* 557.1884; found: *m*/*z* 557.1887.

- Glycoconjugate **A-2**

The product was obtained as a brown solid (0.130 g, 69%); ratio of anomers (α:β = 1.3:1); ^1^H NMR (400 MHz, DMSO): δ 2.91–3.08 (m, 3H, H2-β, H4-α, H4-β), 3.15–3.21 (m, 2H, H2-α, H3-β), 3.49 (m, 1H, H3-α), 3.63 (m, 1H, H5-β), 4.05 (m, 1H, H5-α), 4.30 (t, 1H, *J* = 6.3 Hz, OH-α), 4.38–4.48 (m, 2H, H6a-α, H6a-β), 4.52 (d, 1H, *J* = 4.8 Hz, OH-β), 4.57 (t, 1H, *J* = 6.5 Hz, OH-α), 4.73 (dd, 1H, *J* = 2.2 Hz, *J* = 9.0 Hz, H6b-α), 4.77 (dd, 1H, *J* = 2.2 Hz, *J* = 9.1 Hz, H6b-β), 4.83 (d, 1H, *J* = 4.0 Hz, OH-α), 4.90 (t, 1H, *J* = 4.2 Hz, OH-α), 4.95 (d, 1H, *J* = 4.6 Hz, OH-β), 5.03 (d, 1H, *J* = 4.4 Hz, OH-β), 5.31, 5.32 (2s, 4H, CH_2_O-α, CH_2_O-β), 5.37 (d, 1H, *J* = 5.5 Hz, OH-β), 6.55 (d, 1H, *J* = 4.9 Hz, H1-α), 6.89 (d, 1H, *J* = 6.3 Hz, H1-β), 7.40–7.46 (m, 2H, H7_quin_-α, H7_quin_-β), 7.50–7.58 (m, 6H, H3_quin_-α, H5_quin_-α, H6_quin_-α, H3_quin_-β, H5_quin_-β, H6_quin_-β), 8.23, 8.26 (2s, 2H, H5_triaz_-α, H5_triaz_-β), 8.30–8.34 (m, 2H, H4_quin_-α, H4_quin_-β), 8.79–8.83 (m, 2H, H2_quin_-α, H2_quin_-β); ^13^C NMR (100 MHz, DMSO): δ 51.29, 51.41 (C6-α, C6-β), 61.68, 61.70 (CH_2_O-α, CH_2_O-β), 70.13, 71.53, 72.07, 72.61, 74.26, 74.53, 76.02, 76.13 (C2-α, C3-α, C4-α, C5-α, C2-β, C3-β, C4-β, C5-β), 92.34, 96.81 (C1-α, C1-β), 109.76, 109.81 (C7_quin_-α, C7_quin_-β), 119.94, 119.98 (C5_quin_-α, C5_quin_-β), 121.88, 121.92 (C3_quin_-α, C3_quin_-β), 125.89, 125.97 (C5_triaz_-α, C5_triaz_-β), 126.75, 126.78 (C6_quin_-α, C6_quin_-β), 129.05, 129.08 (C4a_quin_-α, C4a_quin_-β), 135.80, 135.84 (C4_quin_-α, C4_quin_-β), 139.60, 139.63 (C8a_quin_-α, C8a_quin_-β), 142.04, 142.16 (C4_triaz_-α, C4_triaz_-β), 148.93, 148.96 (C2_quin_-α, C2_quin_-β), 153.90, 153.93 (C8_quin_-α, C8_quin_-β); HRMS (ESI-TOF): calcd for C_18_H_21_N_4_O_6_ ([M + H]^+^): *m*/*z* 389.1461; found: *m*/*z* 389.1461.

##### Synthesis of Glycoconjugates *Type B*

The 1,2,3,4-tetra-*O*-acetyl-6-azido-6-deoxy-d-galactopyranose **12** (0.086 g, 0.230 mmol) or 6-azido-6-deoxy-d-galactopyranose **11** (0.106 g, 0.517 mmol) and 8-(2-propyn-1-yloxy)quinoline **24** (1 equiv) were dissolved in *i*-PrOH (3 mL) and THF (3 mL). Then, sodium ascorbate (0.4 equiv) dissolved in H_2_O (1.5 mL) and CuSO_4_·5H_2_O (0.2 equiv) dissolved in H_2_O (1.5 mL), mixed together and added to the obtained solution. The reaction mixture was stirred overnight at room temperature. The reaction progress was monitored on TLC in an eluent system CHCl_3_:MeOH (10:1 for protected or 2:1 for unprotected compounds). After completion of the reaction, the precipitate of inorganic salts was filtered off, and the filtrate was concentrated under reduced pressure. The crude product was purified by column chromatography (toluene:ethyl acetate, 2:1 and chloroform:methanol, 70:1 for fully protected glycoconjugates or chloroform:methanol, gradient: 50:1 to 10:1 for unprotected glycoconjugates).

- Glycoconjugate **B-1**

The product was obtained as a brown solid (0.110 g, 86%); ratio of anomers (α:β = 1:2); ^1^H NMR (400 MHz, CDCl_3_): δ 1.98, 2.03, 2.05, 2.16 (4s, 12H, 4 × CH_3_-β), 1.99, 2.01, 2.03, 2.16 (4s, 12H, 4 × CH_3_-α), 4.22 (m, 1H, H5-β), 4.31–4.44 (m, 2H, H6a-α, H6a-β), 4.50 (dd, 1H, *J* = 4.2 Hz, *J* = 14.1 Hz, H6b-α), 4.55 (m, 1H, H5-α), 4.59 (dd, 1H, *J* = 3.9 Hz, *J* = 14.4 Hz, H6b-β), 5.07 (dd, 1H, *J* = 3.4 Hz, *J* = 10.4 Hz, H3-β), 5.28–5.36 (m, 3H, H2-α, H2-β, H3-α), 5.42 (m, 1H, H4-β), 5.49 (m, 1H, H4-α), 5.53–5.58 (m, 4H, CH_2_O-α, CH_2_O-β), 5.59 (d, 1H, *J* = 8.3 Hz, H1-β), 6.32 (bs, 1H, H1-α), 7.29–7.35 (m, 2H, H7_quin_-α, H7_quin_-β), 7.36–7.60 (m, 6H, H3_quin_-α, H5_quin_-α, H6_quin_-α, H3_quin_-β, H5_quin_-β, H6_quin_-β), 7.77 (s, 1H, H5_triaz_-α), 7.79 (s, 1H, H5_triaz_-β), 8.07–8.19 (m, 2H, H4_quin_-α, H4_quin_-β), 8.88–8.99 (m, 2H, H2_quin_-α, H2_quin_-β); ^13^C NMR (100 MHz, CDCl_3_): δ 20.48, 20.50, 20.58, 20.60, 20.62, 20.69, 20.75, 20.83 (4 × CH_3_-α, 4 × CH_3_-β), 50.06, 50.14 (C6-α, C6-β), 62.70, 62.77 (CH_2_O-α, CH_2_O-β), 66.22, 67.18, 67.51, 67.62, 68.16, 69.85, 70.56, 72.74 (C2-α, C3-α, C4-α, C5-α, C2-β, C3-β, C4-β, C5-β), 89.43 (C1-α), 92.09 (C1-β), 109.51, 109.99 (C7_quin_-α, C7_quin_-β), 120.12, 120.24 (C5_quin_-α, C5_quin_-β), 121.59, 121.61 (C3_quin_-α, C3_quin_-β), 124.45, 124.55 (C5_triaz_-α, C5_triaz_-β), 126.73, 126.81 (C6_quin_-α, C6_quin_-β), 129.48, 129.50 (C4a_quin_-α, C4a_quin_-β), 135.96, 136.03 (C4_quin_-α, C4_quin_-β), 140.34, 140.40 (C8a_quin_-α, C8a_quin_-β), 144.31, 144.44 (C4_triaz_-α, C4_triaz_-β), 149.27, 149.33 (C2_quin_-α, C2_quin_-β), 153.77, 153.80 (C8_quin_-α, C8_quin_-β), 168.62, 168.67, 169.33, 169.74, 169.84, 169.89, 169.92, 169.96 (4 × CO-α, 4 × CO-β); HRMS (ESI-TOF): calcd for C_26_H_29_N_4_O_10_ ([M + H]^+^): *m*/*z* 557.1884; found: *m*/*z* 557.1885.

- Glycoconjugate **B-2**

The product was obtained as a brown solid (0.129 g, 64%); ratio of anomers (α:β = 1.4:1); ^1^H NMR (400 MHz, DMSO): δ 3.17 (m, 1H, H6a-β), 3.28 (m, 1H, H6a-α), 3.53–3.66 (m, 2H, H2-α, H3-α), 3.68 (m, 1H, H5-β), 3.76 (m, 1H, H5-α), 3.97 (dd, 1H, *J* = 3.5 Hz, *J* = 8.9 Hz, H3-β), 4.23 (dd, 1H, *J* = 6.5 Hz, *J* = 7.2 Hz, H2-β), 4.29–4.39 (m, 3H, H4-α, H4-β, H6b-β), 4.47–4.61 (m, 4H, 3 × OH-β, H6b-α), 4.63 (m, 1H, OH-α), 4.74–4.82 (m, 3H, 2 × OH-α, OH-β), 4.93 (m, 1H, OH-α), 5.28–5.36 (m, 4H, CH_2_O-α, CH_2_O-β), 6.41 (d, 1H, *J* = 4.5 Hz, H1-α), 6.79 (d, 1H, *J* = 6.4 Hz, H1-β), 7.39–7.46 (m, 2H, H7_quin_-α, H7_quin_-β), 7.49–7.60 (m, 6H, H3_quin_-α, H5_quin_-α, H6_quin_-α, H3_quin_-β, H5_quin_-β, H6_quin_-β), 8.30–8.35 (m, 2H, H4_quin_-α, H4_quin_-β), 8.30 (s, 1H, H5_triaz_-α), 8.31 (s, 1H, H5_triaz_-β), 8.78–8.84 (m, 2H, H2_quin_-α, H2_quin_-β); ^13^C NMR (100 MHz, DMSO): δ 51.23 (C6-α, C6-β), 61.73 (CH_2_O-α, CH_2_O-β), 68.32, 68.89, 68.92, 69.06, 69.69, 71.51, 72.86, 72.91 (C2-α, C3-α, C4-α, C5-α, C2-β, C3-β, C4-β, C5-β), 92.66 (C1-α), 97.25 (C1-β), 109.78, 109.81 (C7_quin_-α, C7_quin_-β), 119.97, 119.99 (C5_quin_-α, C5_quin_-β), 121.85, 121.88 (C3_quin_-α, C3_quin_-β), 125.68, 125.78 (C5_triaz_-α, C5_triaz_-β), 126.76, 126.79 (C6_quin_-α, C6_quin_-β), 129.03, 129.05 (C4a_quin_-α, C4a_quin_-β), 135.79, 135.84 (C4_quin_-α, C4_quin_-β), 139.62, 139.63 (C8a_quin_-α, C8a_quin_-β), 142.02, 142.15 (C4_triaz_-α, C4_triaz_-β), 148.95, 148.97 (C2_quin_-α, C2_quin_-β), 153.90, 153.91 (C8_quin_-α, C8_quin_-β); HRMS (ESI-TOF): calcd for C_18_H_21_N_4_O_6_ ([M + H]^+^): *m*/*z* 389.1464; found: *m*/*z* 389.1461.

##### Synthesis of Glycoconjugates *Type C*

The 1,2,3,4-tetra-*O*-acetyl-*N*-(prop-2-yn-1-yl)-β-d-glucopyranuronic acid amide **15** (0.085 g, 0.213 mmol) or *N*-(prop-2-yn-1-yl)-d-glucopyranuronic acid amide **16** (0.095 g, 0.411 mmol) and 8-(2-azidoethoxy)quinolone **25** (1 equiv) or 8-(3-azidopropoxy)quinolone **26** (1 equiv) were dissolved in *i*-PrOH (3 mL) and THF (3 mL). Then, sodium ascorbate (0.4 equiv) dissolved in H_2_O (1.5 mL) and CuSO_4_·5H_2_O (0.2 equiv) dissolved in H_2_O (1.5 mL), mixed together and added to the obtained solution. The reaction mixture was stirred overnight at room temperature. The reaction progress was monitored on TLC in an eluent system CHCl_3_:MeOH (10:1 for protected or 2:1 for unprotected compounds). After completion of the reaction, the precipitate of inorganic salts was filtered off, and the filtrate was concentrated under reduced pressure. The crude product was purified by column chromatography (toluene:ethyl acetate, 2:1 and chloroform:methanol, 80:1 for fully protected glycoconjugates or chloroform:methanol, gradient: 50:1 to 5:1 for unprotected glycoconjugates).

- Glycoconjugate **C-1**

The product was obtained as a white solid (0.0989 g, 76%); ^1^H NMR (400 MHz, CDCl_3_): δ 2.01, 2.03, 2.04, 2.11 (4s, 12H, 4 × CH_3_), 4.04 (d, 1H, *J* = 9.8 Hz, H5), 4.46 (dd, 1H, *J* = 5.3 Hz, *J* = 15.2 Hz, CH_2_), 4.51 (dd, 1H, *J* = 5.6 Hz, *J* = 15.2 Hz, CH_2_), 4.66 (m, 2H, CH_2_), 4.97 (t, 2H, *J* = 5.2 Hz, CH_2_), 5.08 (dd, 1H, *J* = 8.2 Hz, *J* = 9.2 Hz, H2), 5.14 (dd~t, 1H, *J* = 9.3 Hz, *J* = 9.8 Hz, H4), 5.28 (dd~t, 1H, *J* = 9.2 Hz, *J* = 9.3 Hz, H3), 5.73 (d, 1H, *J* = 8.2 Hz, H1), 6.89 (m, 1H, NH), 7.04 (d, 1H, *J* = 7.2 Hz, H7_quin_), 7.41–7.50 (m, 3H, H3_quin_, H5_quin_, H6_quin_), 8.12 (s, 1H, H5_triaz_), 8.17 (d, 1H, *J* = 8.2 Hz, H4_quin_), 8.98 (m, 1H, H2_quin_); ^13^C NMR (100 MHz, CDCl_3_): δ 20.54, 20.54, 20.69, 20.75 (4 × CH_3_), 34.59, 49.64, 67.56 (3 × CH_2_), 68.93, 70.17, 72.01, 72.90 (C2, C3, C4, C5), 91.21 (C1), 109.99 (C7_quin_), 121.03 (C5_quin_), 121.91 (C3_quin_), 124.14 (C5_triaz_), 126.52 (C6_quin_), 129.62 (C4a_quin_), 136.05 (C4_quin_), 140.32 (C8a_quin_), 143.96 (C4_triaz_), 149.64 (C2_quin_), 153.78 (C8_quin_), 165.86 (CONH), 168.75, 169.19, 169.60, 169.80 (4 × COCH_3_); HRMS (ESI-TOF): calcd for C_28_H_32_N_5_O_11_ ([M + H]^+^): *m*/*z* 614.2098; found: *m*/*z* 614.2091.

- Glycoconjugate **C-2**

The product was obtained as a white solid (0.101 g, 76%); ^1^H NMR (400 MHz, CDCl_3_): δ 2.01, 2.03, 2.04, 2.12 (4s, 12H, 4 × CH_3_), 2.63 (q, 2H, *J* = 6.2 Hz, CH_2_), 4.03 (d, 1H, *J* = 9.9 Hz, H5), 4.26 (m, 2H, CH_2_), 4.43 (dd, 1H, *J* = 5.5 Hz, *J* = 15.2 Hz, CH_2_), 4.53 (dd, 1H, *J* = 6.2 Hz, *J* = 15.2 Hz, CH_2_), 4.72 (t, 2H, *J* = 6.6 Hz, CH_2_), 5.09 (dd, 1H, *J* = 8.2 Hz, *J* = 9.3 Hz, H2), 5.14 (dd~t, 1H, *J* = 9.3 Hz, *J* = 9.9 Hz, H4), 5.29 (dd~t, 1H, *J* = 9.3 Hz, *J* = 9.3 Hz, H3), 5.73 (d, 1H, *J* = 8.2 Hz, H1), 6.90 (t, 1H, *J* = 5.5 Hz, NH), 7.05 (m, 1H, H7_quin_), 7.41–7.48 (m, 3H, H3_quin_, H5_quin_, H6_quin_), 7.70 (s, 1H, H5_triaz_), 8.16 (m, 1H, H4_quin_), 8.96 (m, 1H, H2_quin_); ^13^C NMR (100 MHz, CDCl_3_): δ 20.53, 20.53, 20.70, 20.75 (4 × CH_3_), 29.67, 34.53, 47.36, 65.37 (4 × CH_2_), 68.93, 70.15, 71.98, 72.86 (C2, C3, C4, C5), 91.21 (C1), 109.47 (C7_quin_), 120.26 (C5_quin_), 121.71 (C3_quin_), 123.22 (C5_triaz_), 126.73 (C6_quin_), 129.57 (C4a_quin_), 136.16 (C4_quin_), 140.21 (C8a_quin_), 143.96 (C4_triaz_), 149.33 (C2_quin_), 154.24 (C8_quin_), 165.93 (CONH), 168.77, 169.19, 169.64, 169.79 (4 × COCH_3_); HRMS (ESI-TOF): calcd for C_29_H_34_N_5_O_11_ ([M + H]^+^): *m*/*z* 628.2255; found: *m*/*z* 628.2252.

- Glycoconjugate **C-3**

The product was obtained as a white solid (0.127 g, 67%); ratio of anomers (α:β = 1:1.1); ^1^H NMR (400 MHz, DMSO): δ 2.34–2.44 (m, 4H, CH_2_-α, CH_2_-β), 2.96 (m, 1H, H2-β), 3.14 (m, 1H, H4-β), 3.21 (m, 1H, H2-α), 3.29 (m, 1H, H4-α), 3.38 (m, 1H, H3-β), 3.44 (m, 1H, H3-α), 3.58 (d, 1H, *J* = 9.7 Hz, H5-β), 4.00 (d, 1H, *J* = 9.8 Hz, H5-α), 4.15–4.23 (m, 4H, CH_2_-α, CH_2_-β), 4.29–4.35 (m, 5H, CH_2_-α, CH_2_-β, OH), 4.56–4.64 (m, 5H, CH_2_-α, CH_2_-β, OH), 4.80 (m, 1H, OH), 4.93 (m, 1H, OH), 4.95 (m, 1H, OH), 4.97 (m, 1H, OH), 5.04 (m, 1H, OH), 5.09 (m, 1H, OH), 6.45 (d, 1H, *J* = 4.7 Hz, H1-α), 6.76 (d, 1H, *J* = 6.6 Hz, H1-β), 7.20 (m, 2H, H7_quin_-α, H7_quin_-β), 7.47–7.61 (m, 6H, H3_quin_-α, H5_quin_-α, H6_quin_-α, H3_quin_-β, H5_quin_-β, H6_quin_-β), 7.99, 8.02 (2s, 2H, H5_triaz_-α, H5_triaz_-β), 8.33 (m, 2H, H4_quin_-α, H4_quin_-β), 8.41 (m, 2H, NH-α, NH-β), 8.90 (bs, 2H, H2_quin_-α, H2_quin_-β); ^13^C NMR (100 MHz, DMSO): δ 29.66, 29.66, 34.22, 34.22, 46.56, 46.56, 65.40, 65.40 (4 × CH_2_-α, 4 × CH_2_-β), 71.27, 71.48, 71.88, 72.20, 72.71, 74.46, 75.56, 76.32 (C2-α, C3-α, C4-α, C5-α, C2-β, C3-β, C4-β, C5-β), 92.77, 97.36 (C1-α, C1-β), 109.82, 109.88 (C7_quin_-α, C7_quin_-β), 119.86, 119.94 (C5_quin_-α, C5_quin_-β), 121.78, 121.87 (C3_quin_-α, C3_quin_-β), 122.97, 123.03 (C5_triaz_-α, C5_triaz_-β), 126.81, 126.89 (C6_quin_-α, C6_quin_-β), 129.04, 129.10 (C4a_quin_-α, C4a_quin_-β), 135.77, 135.86 (C4_quin_-α, C4_quin_-β), 139.77, 139.82 (C8a_quin_-α, C8a_quin_-β), 144.74, 144.92 (C4_triaz_-α, C4_triaz_-β), 149.01, 149.05 (C2_quin_-α, C2_quin_-β), 154.15, 154.21 (C8_quin_-α, C8_quin_-β), 168.78, 169.82 (CONH-α, CONH-β); HRMS (ESI-TOF): calcd for C_21_H_26_N_5_O_7_ ([M + H]^+^): *m*/*z* 460.1832; found: *m*/*z* 460.1829.

##### Synthesis of Glycoconjugates *Type D*

The 2,3,4,2′,3′,4′-hexa-*O*-acetyl-6,6′-diazido-6,6′-dideoxy-d-trehalose **23** (0.095 g, 0.147 mmol) or 6,6′-diazido-6,6′-dideoxy-d-trehalose **20** (0.203 g, 0.517 mmol) and 8-(2-propyn-1-yloxy)quinoline **24** (2 equiv) were dissolved in *i*-PrOH (4 mL) and THF (4 mL). Then, sodium ascorbate (0.4 equiv) dissolved in H_2_O (2 mL) and CuSO_4_·5H_2_O (0.2 equiv) dissolved in H_2_O (2 mL), mixed together and added to the obtained solution. The reaction mixture was stirred overnight at room temperature. The reaction progress was monitored on TLC in an eluent system CHCl_3_:MeOH (10:1 for protected or 1:1 for unprotected compounds). After completion of the reaction, the precipitate of inorganic salts was filtered off, and the filtrate was concentrated under reduced pressure. The crude product was purified by column chromatography (toluene:ethyl acetate, 2:1 and chloroform:methanol, 50:1 for fully protected glycoconjugates or chloroform:methanol, gradient: 50:1 to 5:1 for unprotected glycoconjugates).

- Glycoconjugate **D-1**

The product was obtained as a yellow solid (0.113 g, 76%); m.p.: 118 °C; [α]^23^_D_ = 15.8 (c = 1.0, CHCl_3_); ^1^H NMR (400 MHz, CDCl_3_): δ 2.00, 2.03, 2.09 (3s, 18H, 6 × CH_3_), 3.88 (dd, 2H, *J* = 9.4 Hz, *J* = 13.9 Hz, 2 × H6a), 3.99 (ddd, 2H, *J* = 1.3 Hz, *J* = 9.4 Hz, *J* = 10.0 Hz, 2 × H5), 4.32 (d, 2H, *J* = 3.8 Hz, 2 × H1), 4.44 (dd, 2H, *J* = 1.3 Hz, *J* = 13.9 Hz, 2 × H6b), 4.70 (dd~t, 2H, *J* = 9.7 Hz, *J* = 10.0 Hz, 2 × H4), 4.72 (dd, 2H, *J* = 3.8 Hz, *J* = 9.7 Hz, 2 × H2), 5.31 (dd~t, 2H, *J* = 9.7 Hz, *J* = 9.7 Hz, 2 × H3), 5.53 i 5.57 (qAB, 4H, *J* = 13.2 Hz, 2 × CH_2_O), 7.32 (m, 2H, 2 × H7_quin_), 7.39 (dd, 2H, *J* = 4.1 Hz, *J* = 8.3 Hz, 2 × H5_quin_), 7.44 (m, 2H, 2 × H6_quin_), 7.50 (t, 2H, *J* = 7.9 Hz, 2 × H3_quin_), 7.64 (s, 2H, 2 × H5_triaz_), 8.14 (dd, 2H, *J* = 1.2 Hz, *J* = 8.3 Hz, 2 × H4_quin_), 8.88 (m, 2H, 2 × H2_quin_); ^13^C NMR (100 MHz, CDCl_3_): δ 20.51, 20.59, 20.65 (6 × CH_3_), 50.56 (2 × C6), 62.72 (2 × CH_2_OH), 68.54, 68.83, 69.63, 69.73 (2 × C2, 2 × C3, 2 × C4, 2 × C5), 90.97 (2 × C1), 109.87 (2 × C7_quin_), 120.10 (2 × C5_quin_), 121.71 (2 × C3_quin_), 124.78 (2 × C5_triaz_), 127.07 (2 × C6_quin_), 129.49 (2 × C4a_quin_), 136.00 (2 × C4_quin_), 140.38 (2 × C8a_quin_), 144.48 (2 × C4_triaz_), 149.30 (2 × C2_quin_), 153.91 (2 × C8_quin_), 169.24, 169.70, 169.81 (6 × CO); HRMS (ESI-TOF): calcd for C_48_H_51_N_8_O_17_ ([M + H]^+^): *m*/*z* 1011.3372; found: *m*/*z* 1011.3370.

- Glycoconjugate **D-2**

The product was obtained as a brown solid (0.244 g, 62%); m.p.: 182–183 °C; [α]^24^_D_ = 40.0 (c = 1.0, DMSO); ^1^H NMR (400 MHz, DMSO): δ 3.00 (m, 2H, 2 × H4), 3.22 (m, 2H, 2 × H2), 3.61 (m, 2H, 2 × H3), 4.25 (m, 2H, 2 × H5), 4.47 (dd, 2H, *J* = 8.2 Hz, *J* = 14.2 Hz, 2 × H6a), 4.62 (d, 2H, *J* = 3.6 Hz, 2 × H1), 4.65 (dd, 2H, *J* = 2.3 Hz, *J* = 14.2 Hz, 2 × H6b), 5.11 (bs, 2H, 2 × OH), 5.32 i 5.35 (qAB, 4H, *J* = 12.0 Hz, 2 × CH_2_O), 5.36 (bs, 2H, 2 × OH), 5.44 (bs, 2H, 2 × OH), 7.38 (m, 2H, *J* = 2.7 Hz, *J* = 6.3 Hz, 2 × H7_quin_), 7.47–7.56 (m, 6H, 2 × H3_quin_, 2 × H5_quin_, 2 × H6_quin_), 8.27 (s, 2H, 2 × H5_triaz_), 8.32 (dd, 2H, *J* = 1.7 Hz, *J* = 8.3 Hz, 2 × H4_quin_), 8.89 (dd, 2H, *J* = 1.7 Hz, *J* = 4.2 Hz, 2 × H2_quin_); ^13^C NMR (100 MHz, DMSO): δ 50.80 (2 × C6), 62.17 (2 × CH_2_OH), 69.75, 70.97, 71.34, 72.64 (2 × C2, 2 × C3, 2 × C4, 2 × C5), 94.57 (2 × C1), 109.94 (2 × C7_quin_), 119.96 (2 × C5_quin_), 121.78 (2 × C3_quin_), 124.53 (2 × C5_triaz_), 126.73 (2 × C6_quin_), 129.05 (2 × C4a_quin_), 136.01 (2 × C4_quin_), 139.46 (2 × C8a_quin_), 142.68 (2 × C4_triaz_), 149.32 (2 × C2_quin_), 153.70 (2 × C8_quin_); HRMS (ESI-TOF): calcd for C_36_H_39_N_8_O_11_ ([M + H]^+^): *m*/*z* 759.2738; found: *m*/*z* 759.2735.

#### 3.2.3. General Procedure for the Synthesis of Metabolites of Glycoconjugates

The appropriate sugar derivatives **5**, **6**, **11**, **12**, **15**, **16**, **20**, or **23** (1 equiv) and propargyl alcohol **27** or 2-azidoethanol **28** (1 equiv) were dissolved in *i*-PrOH (4 mL) and THF (4 mL). Then, sodium ascorbate (0.4 equiv) dissolved in H_2_O (2 mL) and CuSO_4_·5H_2_O (0.2 equiv) dissolved in H_2_O (2 mL), mixed together and added to the obtained solution. The reaction mixture was stirred overnight at room temperature. The reaction progress was monitored on TLC in an eluent system CHCl_3_:MeOH (10:1 for protected or 2:1 for unprotected compounds). After completion of the reaction, the precipitate of inorganic salts was filtered off, and the filtrate was concentrated under reduced pressure. The crude product was purified by column chromatography (toluene:ethyl acetate, 2:1 and chloroform:methanol, 20:1 for protected products or chloroform:methanol, gradient: 50:1 to 1:1 for unprotected products) to give metabolites **29**–**36**.

- Metabolite **29**

Starting from 1,2,3,4-tetra-*O*-acetyl-6-azido-6-deoxy-d-glucopyranose **5** (0.527 g, 1.412 mmol) and propargyl alcohol **27** (83 µL, 1.421 mmol). The product was obtained as a white solid (0.522 g, 86%); ratio of anomers (α:β = 1.5:1); ^1^H NMR (400 MHz, DMSO): δ 1.93, 1.97, 1.98, 2.00, 2.00, 2.01, 2.05, 2.14 (8s, 24H, 4 × CH_3_-α, 4 × CH_3_-β), 4.40–4.48 (m, 2H, H5-α, H5-β), 4.50, 4.51 (2s, 4H, CH_2_O-α, CH_2_O-β), 4.52–4.63 (m, 4H, H6a-α, H6b-α, H6a-β, H6b-β), 4.82 (dd~t, 1H, *J* = 9.4 Hz, *J* = 9.5 Hz, H4-β), 4.91 (dd, 1H, *J* = 9.4 Hz, *J* = 10.1 Hz, H4-α), 4.92 (dd, 1H, *J* = 8.3 Hz, *J* = 9.8 Hz, H2-β), 4.99 (dd, 1H, *J* = 3.7 Hz, *J* = 10.3 Hz, H2-α), 5.15–5.19 (m, 2H, OH-α, OH-β), 5.32 (dd, 1H, *J* = 9.4 Hz, *J* = 10.3 Hz, H3-α), 5.42 (dd~t, 1H, *J* = 9.5 Hz, *J* = 9.8 Hz, H3-β), 5.93 (d, 1H, *J* = 8.3 Hz, H1-β), 6.14 (d, 1H, *J* = 3.7 Hz, H1-α), 7.80 (s, 1H, H5_triaz_-β), 7.88 (s, 1H, H5_triaz_-α); ^13^C NMR (100 MHz, DMSO): δ 20.24, 20.25, 20.30, 20.33, 20.43, 20.45, 20.49, 20.52 (4 × CH_3_-α, 4 × CH_3_-β), 49.42, 49.54 (C6-α, C6-β), 54.95, 54.97 (CH_2_OH-α, CH_2_OH-β), 68.44, 68.80, 69.03, 69.28, 69.30, 69.80, 71.76, 71.78 (C2-α, C3-α, C4-α, C5-α, C2-β, C3-β, C4-β, C5-β), 88.02, 90.77 (C1-α, C1-β), 123.36, 123.47 (C5_triaz_-α, C5_triaz_-β), 147.95, 147.97 (C4_triaz_-α, C4_triaz_-β), 168.59, 168.81, 169.05, 169.08, 169.24, 169.48, 169.51, 169.68 (4 × CO-α, 4 × CO-β); HRMS (ESI-TOF): calcd for C_17_H_24_N_3_O_10_ ([M + H]^+^): *m*/*z* 430.1462; found: *m*/*z* 430.1463.

- Metabolite **30**

Starting from 6-azido-6-deoxy-d-glucopyranose **6** (0.093 g, 0.453 mmol) and propargyl alcohol **27** (30 µL, 0.514 mmol). The product was obtained as a brown solid (0.82 g, 69%); ratio of anomers (α:β = 1:1); ^1^H NMR (400 MHz, DMSO): δ 2.87–3.03 (m, 3H, H2-β, H4-α, H4-β), 3.09–3.15 (m, 2H, H2-α, H3-β), 3.19 (m, 1H, H3-α), 3.40–3.46 (m, 2H, H6a-α, H6a-β), 3.47–3.55 (m, 2H, H6b-α, H6b-β), 3.93 (ddd, 1H, *J* = 2.2 Hz, *J* = 8.4 Hz, *J* = 10.2 Hz, H5-α), 4.25 (d, 1H, *J* = 7.8 Hz, H1-β), 4.35 (ddd, 1H, *J* = 3.3 Hz, *J* = 8.3 Hz, *J* = 14.3 Hz, H5-β), 4.50, 4.51 (2s, 4H, CH_2_O-α, CH_2_O-β), 4.66 (m, 2H, OH-α, OH-β), 4.88 (d, 1H, *J* = 3.4 Hz, H1-α), 7.83, 7.87 (2s, 2H, H5_triaz_-α, H5_triaz_-β); ^13^C NMR (100 MHz, DMSO): δ 50.92, 50.99 (C6-α, C6-β), 54.97, 54.98 (CH_2_OH-α, CH_2_OH-β), 70.01, 71.35, 71.82, 72.06, 72.62, 74.21, 74.54, 76.13 (C2-α, C3-α, C4-α, C5-α, C2-β, C3-β, C4-β, C5-β), 92.30, 96.84 (C1-α, C1-β), 123.45, 123.51 (C5_triaz_-α, C5_triaz_-β), 147.55, 147.64 (C4_triaz_-α, C4_triaz_-β); HRMS (ESI-TOF): calcd for C_9_H_16_N_3_O_6_ ([M + H]^+^): *m*/*z* 262.1039; found: *m*/*z* 262.1040.

- Metabolite **31**

Starting from 1,2,3,4-tetra-*O*-acetylo-6-azido-6-deoxy-d-galactopyranose **12** (0.111 g, 0.297 mmol) and propargyl alcohol **27** (20 µL, 0.342 mmol). The product was obtained as a white solid (0.111 g, 87%); ratio of anomers (α:β = 1:2.5); ^1^H NMR (400 MHz, DMSO): δ 1.91, 2.00, 2.05, 2.16 (4s, 12H, 4 × CH_3_-β), 1.94, 2.00, 2.11, 2.15 (4s, 12H, 4 × CH_3_-α), 4.45–4.52 (m, 6H, H6a-α, H6a-β, CH_2_-α, CH_2_-β), 4.53 (m, 1H, H6b-β), 4.57 (m, 1H, H6b-α), 4.65 (m, 1H, H5-β), 4.73 (m, 1H, H5-α), 5.06–5.14 (m, 2H, H2-α, H2-β), 5.15–5.22 (m, 4H, H4-α, H4-β, OH-α, OH-β), 5.26 (dd, 1H, *J* = 3.3 Hz, *J* = 10.9 Hz, H3-α), 5.32 (dd, 1H, *J* = 3.5 Hz, *J* = 10.5 Hz, H3-β), 5.86 (d, 1H, *J* = 8.3 Hz, H1-β), 6.21 (d, 1H, *J* = 3.7 Hz, H1-α), 7.87 (s, 1H, H5_triaz_-β), 7.90 (s, 1H, H5_triaz_-α); ^13^C NMR (100 MHz, DMSO): δ 20.19, 20.21, 20.26, 20.29, 20.33, 20.35, 20.37, 20.42 (4 × CH_3_-α, 4 × CH_3_-β), 48.48, 48.68 (C6-α, C6-β), 54.86, 54.89 (CH_2_OH-α, CH_2_OH-β), 65.90, 66.77, 67.26, 67.34, 67.44, 69.09, 69.82, 71.69 (C2-α, C3-α, C4-α, C5-α, C2-β, C3-β, C4-β, C5-β), 88.53 (C1-α), 91.17 (C1-β), 123.04, 123.09 (C5_triaz_-α, C5_triaz_-β), 147.88, 147.97 (C4_triaz_-α, C4_triaz_-β), 168.57, 168.81, 169.11, 169.16, 169.30, 169.41, 169.67, 169.70 (4 × CO-α, 4 × CO-β); HRMS (ESI-TOF): calcd for C_17_H_24_N_3_O_10_ ([M + H]^+^): *m*/*z* 430.1462; found: *m*/*z* 430.1461.

- Metabolite **32**

Starting from 6-azido-6-deoxy-d-galactopyranose **11** (0.126 g, 0.614 mmol) and propargyl alcohol **27** (36 µL, 0.616 mmol). The product was obtained as a brown solid (0.098 g, 61%); ratio of anomers (α:β = 2.2:1); ^1^H NMR (400 MHz, DMSO): δ 3.17 (m, 1H, H6a-β), 3.28 (m, 1H, H6a-α), 3.50–3.59 (m, 4H, H2-α, H3-α, H2-β, H3-β), 3.67 (m, 1H, H5-α), 3.87 (m, 1H, H5-β), 4.19 (m, 1H, H4-β), 4.23 (m, 1H, H4-α), 4.35 (m, 1H, H6b-α), 4.40–4.53 (m, 9H, H6b-β, CH_2_-α, CH_2_-β, 4 × OH-β), 4.61 (d, 1H, *J* = 5.1 Hz, OH-α), 4.70–4.80 (m, 2H, 2 × OH-α), 4.90 (m, 1H, OH-α), 5.11–5.21 (m, 2H, CH_2_OH-α, CH_2_OH-β), 6.24 (d, 1H, *J* = 4.8 Hz, H1-α), 6.63 (d, 1H, *J* = 6.7 Hz, H1-β), 7.90 (s, 1H, H5_triaz_-α), 7.92 (s, 1H, H5_triaz_-β); ^13^C NMR (100 MHz, DMSO): δ 50.81, 50.86 (C6-α, C6-β), 55.01, 55.06 (CH_2_OH-α, CH_2_OH-β), 68.34, 68.89, 68.91, 69.53 (C2-α, C3-α, C4-α, C5-α), 71.60, 71.66, 72.89, 72.94 (C2-β, C3-β, C4-β, C5-β), 92.66 (C1-α), 97.33 (C1-β), 123.32, 123.35 (C5_triaz_-α, C5_triaz_-β), 147.58, 147.67 (C4_triaz_-α, C4_triaz_-β); HRMS (ESI-TOF): calcd for C_9_H_16_N_3_O_6_ ([M + H]^+^): *m*/*z* 262.1039; found: *m*/*z* 262.1039.

- Metabolite **33**

Starting from 1,2,3,4-tetra-*O*-acetyl-*N*-(prop-2-yn-1-yl)-β-d-glucopyranuronic acid amide **15** (0.104 g, 0.260 mmol) and 2-azidoethanol **28** (0.023 g, 0.264 mmol). The product was obtained as a white solid (0.092 g, 73%); ^1^H NMR (400 MHz, CDCl_3_): δ 2.00, 2.03, 2.06, 2.13 (4s, 12H, 4 × CH_3_), 4.04 (d, 1H, *J* = 9.9 Hz, H5), 4.05 (m, 2H, CH_2_), 4.30–4.45 (m, 2H, CH_2_), 4.51–4.66 (m, 2H, CH_2_), 5.06 (dd~t, 1H, *J* = 9.4 Hz, *J* = 9.9 Hz, H4), 5.09 (dd, 1H, *J* = 8.2 Hz, *J* = 9.4 Hz, H2), 5.30 (dd~t, 1H, *J* = 9.4 Hz, *J* = 9.4 Hz, H3), 5.74 (d, 1H, *J* = 8.2 Hz, H1), 7.10 (m, 1H, NH), 7.67 (s, 1H, H5_triaz_); ^13^C NMR (100 MHz, CDCl_3_): δ 20.52, 20.52, 20.71, 20.75 (4 × CH_3_), 34.31, 53.07, 61.23 (3 × CH_2_), 69.15, 70.07, 71.76, 72.87 (C2, C3, C4, C5), 91.16 (C1), 123.77 (C5_triaz_), 144.09 (C4_triaz_), 166.04 (CONH), 168.85, 169.21, 169.75, 170.71 (4 × COCH_3_); HRMS (ESI-TOF): calcd for C_19_H_27_N_4_O_11_ ([M + H]^+^): *m*/*z* 487.1676; found: *m*/*z* 487.1672.

- Metabolite **34**

Starting from *N*-(prop-2-yn-1-yl)-d-glucopyranuronic acid amide **16** (0.318 g, 1.375 mmol) and 2-azidoethanol **28** (0.120 g, 1.378 mmol). The product was obtained as a brown solid (0.280 g, 64%); ratio of anomers (α:β = 1:1); ^1^H NMR (400 MHz, DMSO): δ 2.95 (dd, 1H, *J* = 8.2 Hz, *J* = 9.0 Hz, H2-β), 3.14 (m, 1H, H4-β), 3.19 (dd, 1H, *J* = 3.6 Hz, *J* = 9.2 Hz, H2-α), 3.31 (m, 1H, H4-α), 3.36 (m, 1H, H3-β), 3.44 (dd~t, 1H, *J* = 9.0 Hz, *J* = 9.2 Hz, H3-α), 3.58 (d, 1H, *J* = 9.6 Hz, H5-β), 3.72–3.77 (m, 4H, CH_2_-α, CH_2_-β), 4.00 (d, 1H, *J* = 9.6 Hz, H5-α), 4.29–4.33 (m, 4H, CH_2_-α, CH_2_-β), 4.31 (d, 1H, *J* = 8.2 Hz, H1-β), 4.33–4.37 (m, 4H, CH_2_-α, CH_2_-β), 4.95 (d, 1H, *J* = 3.6 Hz, H1-α), 7.81, 7.84 (2s, 2H, H5_triaz_-α, H5_triaz_-β), 8.42 (m, 2H, NH-α, NH-β); ^13^C NMR (100 MHz, DMSO): δ 34.04, 34.09, 51.97, 52.04, 59.71, 59.76 (3 × CH_2_-α, 3 × CH_2_-β), 71.17, 71.39, 71.78, 72.11, 72.62, 74.35, 75.46, 76.22 (C2-α, C3-α, C4-α, C5-α, C2-β, C3-β, C4-β, C5-β), 92.67, 97.26 (C1-α, C1-β), 123.07, 123.13 (C5_triaz_-α, C5_triaz_-β), 144.29, 144.49 (C4_triaz_-α, C4_triaz_-β), 168.67, 169.71 (CONH-α, CONH-β); HRMS (ESI-TOF): calcd for C_11_H_18_N_4_O_7_Na ([M + Na]^+^): *m*/*z* 341.1073; found: *m*/*z* 341.1072.

- Metabolite **35**

Starting from 2,3,4,2′,3′,4′-hexa-*O*-acetyl-6,6′-diazido-6,6′-dideoxy-d-trehalose **23** (0.076 g, 0.118 mmol) and propargyl alcohol **27** (15 µL, 0.257 mmol). The product was obtained as a yellow solid (0.081 g, 91%); m.p.: 92–93 °C; [α]^23^_D_ = 72.9 (c = 0.27, CHCl_3_); ^1^H NMR (400 MHz, DMSO): δ 1.95, 2.00, 2.02 (3s, 18H, 6 × CH_3_), 4.23 (ddd, 2H, *J* = 2.8 Hz, *J* = 7.8 Hz, *J* = 10.4 Hz, 2 × H5), 4.48 (d, 4H, *J* = 5.6 Hz, 2 × CH_2_O), 4.50 (dd, 2H, *J* = 7.8 Hz, *J* = 14.4 Hz, 2 × H6a), 4.60 (dd, 2H, *J* = 2.8 Hz, *J* = 14.4 Hz, 2 × H6b), 4.85 (d, 2H, *J* = 3.6 Hz, 2 × H1), 4.95 (dd~t, 2H, *J* = 9.4 Hz, *J* = 10.4 Hz, 2 × H4), 5.01 (dd, 2H, *J* = 3.6 Hz, *J* = 10.3 Hz, 2 × H2), 5.12 (t, 2H, *J* = 5.6 Hz, 2 × OH), 5.27 (dd, 2H, *J* = 9.4 Hz, *J* = 10.3 Hz, 2 × H3), 7.84 (s, 2H, 2 × H5_triaz_); ^13^C NMR (100 MHz, DMSO): δ 20.33, 20.36, 20.46 (6 × CH_3_), 49.42 (2 × C6), 54.96 (2 × CH_2_OH), 68.05, 68.43, 69.14, 69.53 (2 × C2, 2 × C3, 2 × C4, 2 × C5), 90.92 (2 × C1), 123.67 (2 × C5_triaz_), 148.01 (2 × C4_triaz_), 169.00, 169.30, 169.69 (6 × CO); HRMS (ESI-TOF): calcd for C_30_H_41_N_6_O_17_ ([M + H]^+^): *m*/*z* 757.2528; found: *m*/*z* 757.2526.

- Metabolite **36**

Starting from 6,6′-diazido-6,6′-dideoxy-d-trehalose **20** (0.097 g, 0.247 mmol) and propargyl alcohol **27** (30 µL, 0.514 mmol). The product was obtained as a yellow solid (0.102 g, 82%); m.p.: 118 °C; [α]^24^_D_ = 102.0 (c = 1.0, DMSO); ^1^H NMR (400 MHz, DMSO): δ 2.95 (dd~t, 2H, *J* = 9.1 Hz, *J* = 9.9 Hz, 2 × H4), 3.17 (dd, 2H, *J* = 3.6 Hz, *J* = 9.2 Hz, 2 × H2), 3.55 (dd~t, 2H, *J* = 9.1 Hz, *J* = 9.2 Hz, 2 × H3), 4.12 (ddd, 2H, *J* = 2.6 Hz, *J* = 7.2 Hz, *J* = 9.9 Hz, 2 × H5), 4.43 (dd, 2H, *J* = 7.2 Hz, *J* = 14.3 Hz, 2 × H6a), 4.49 (s, 4H, 2 × CH_2_O), 4.56 (dd, 2H, *J* = 2.6 Hz, *J* = 14.3 Hz, 2 × H6b), 4.60 (d, 2H, *J* = 3.6 Hz, 2 × H1), 7.84 (s, 2H, 2 × H5_triaz_); ^13^C NMR (100 MHz, DMSO): δ 50.46 (2 × C6), 54.97 (2 × CH_2_OH), 69.77, 71.14, 71.19, 72.60 (2 × C2, 2 × C3, 2 × C4, 2 × C5), 93.74 (2 × C1), 123.13 (2 × C5_triaz_), 147.76 (2 × C4_triaz_); HRMS (ESI-TOF): calcd for C_18_H_29_N_6_O_11_ ([M + H]^+^): *m*/*z* 505.1894; found: *m*/*z* 505.1895.

### 3.3. Biological Evaluation

#### 3.3.1. Cell Cultures

Three cell lines were used for biological assays. The human colon adenocarcinoma cell line (HCT-116) was purchased from the American Type Culture Collection (ATCC, Manassas, VA, USA). The human breast adenocarcinoma cell line (MCF-7) was obtained from the collection of the Maria Skłodowska-Curie Memorial Cancer Center and the National Institute of Oncology branch in Gliwice, Poland. The Normal Human Dermal Fibroblasts-Neonatal (NHDF-Neo) was purchased from Lonza (Cat. No. CC-2509, NHDF-Neo, Dermal Fibroblasts, Neonatal, Lonza, Warsaw, Poland). All cells were cultured as a monolayer in a complete medium under standard conditions in a humidified incubator with 5% CO_2_ at 37 °C. The complete culture media consisted of RPMI 1640 or DMEM/F12 medium (HyClone Laboratories, Inc., Logan, UT, USA), supplemented with 10% of heat-inactivated fetal bovine serum (FBS; EURx, Gdańsk, Poland) and 1% of Antibiotic Antimycotic Solution: penicillin (10,000 U/mL) and streptomycin (10,000 µg/mL) (Sigma-Aldrich, Taufkirchen, Germany).

#### 3.3.2. MTT Cytotoxicity Assay

The in vitro cytotoxicity of the test compounds was determined by the MTT (3-[4,5–dimethylthiazol-2-yl]-2,5-diphenyltetrazolium bromide) test (Sigma-Aldrich, Taufkirchen, Germany) according to the manufacturer’s protocol [58]. At the beginning, for each cell line were determined the optimal seeding densities to ensure exponential growth throughout the experimental period. The confluent layer of cells were trypsinized, collected, and suspended in a growth medium. The cell suspension were then seeded into 96-well plates at a density of 1 × 10^4^ or 2 × 10^3^ (HCT-116) or 5 × 10^3^ (MCF-7, NHDF-Neo) per well and incubated for 24 h under standard conditions (5% CO_2_, 37 °C). Then, the culture medium was replaced with solutions of different concentrations of test compounds in a fresh medium. Stock solutions of test compounds were prepared in DMSO and diluted to final concentrations with appropriate volumes of the growth medium directly before the experiment. Cells suspended in a medium supplemented with 0.5% DMSO (the amount of DMSO necessary to dissolve the highest dose of the respective sample) were used as a control of the experiment. The final DMSO concentration did not affect cell viability. The compound-treated cells were incubated for a further 24–72 h. After that, the medium was removed and the MTT solution (50 µL, 0.5 mg/mL in PBS) was added into each well and incubated for another 3 h under standard conditions (5% CO_2_, 37 °C). After this time, the MTT dye was carefully removed, and the acquired formazan crystals were dissolved by DMSO (Chempur, Piekary Śląskie, Poland). The proliferation was evaluated by measuring the formazan product’s absorbance at a wavelength of 570 nm with a microplate spectrophotometer (Epoch, BioTek, Winooski, VT, USA). All experiments were conducted in triplicate with four technical repeats for each tested concentration. Results were expressed as the percentage change in the viability of the test cells relative to the untreated control cells and calculated as the inhibitory concentration (IC_50_). The IC_50_ values were defined as the drug concentration that was necessary to reduce cell proliferation to 50% of the untreated control and this parameter was calculated using CalcuSyn software (version 2.0, Biosoft, Cambridge, UK). The results are expressed as the average value ± standard deviation (SD).

#### 3.3.3. Clonogenic Assay

To determine the number of colonies forming by cells after exposure to the test compounds, a clonogenic assay was performed as reported in the literature [60]. The cells were seeded into 6-well plates at a density of 1 × 10^5^ (HCT-116) or 2 × 10^5^ (MCF-7, NHDF-Neo) per well (3 mL) and allowed to attach for 24 h under standard conditions with 5% CO_2_ at 37 °C. The following day, the medium was removed and cells were treated with solutions of test compounds in fresh medium at a dose equal to their IC_50_ values. Control cells were grown with fresh medium supplemented with 0.5% DMSO. After 72 h of incubation, the cells were collected by trypsinization and then reseeded into new 6-well plates at a density of 1000 cells/well/3 mL. The plates with cells were incubated for 10 days under standard conditions with 5% CO_2_ at 37 °C. After this time, the medium was removed and the cells were washed with PBS, fixed with ice-cold ethanol (-20 °C) for 3 min, and washed again with PBS. Finally, cells were stained with crystal violet (0.01% in H_2_O; Sigma-Aldrich, Taufkirchen, Germany) for 20 min. The plates were then washed with H_2_O and allowed to air-dry.

#### 3.3.4. Wound-Healing Assay

To analyze the migration properties of cells exposed to test compounds, a wound-healing assay was performed as reported in the literature [61,62]. The cells were seeded into 6-well plates at a density of 3 × 10^5^ (HCT-116) or 4 × 10^5^ (MCF-7, NHDF-Neo) per well (3 mL) and allowed to attach for 24 h under standard conditions with 5% CO_2_ at 37 °C. After forming a confluent monolayer, a linear scratch in each sample with a 200 µL pipette tip was made and the first photo at time zero was taken. The cells were washed with PBS and then treated with solutions of test compounds in a fresh medium at a dose equal to their IC_50_ values. Control cells were grown with fresh medium supplemented with 0.5% DMSO. The cells were incubated and monitored for 72 h under standard conditions with 5% CO_2_ at 37 °C. Cell monitoring and photos were taken using Live Cell Movie Analyzer, JuLI™ Br & FL (NanoEnTek Inc., Seoul, Korea).

#### 3.3.5. Apoptosis and Cell Cycle Analyses by Flow Cytometry

The cell cycle and the type of cell death induced by the test compounds were measured by using flow cytometry according to the manufacturer’s protocol. The cells were seeded into 6-well plates at a density of 3 × 10^5^ (HCT-116) or 4 × 10^5^ (MCF-7, NHDF-Neo) per well (3 mL) and allowed to attach for 24 h under standard conditions with 5% CO_2_ at 37 °C. The following day, the medium was removed and cells were treated with solutions of test compounds in fresh medium at a dose equal to their IC_50_ values. Control cells were grown with fresh medium supplemented with 0.5% DMSO. After 24 h of incubation, the cells were collected by trypsinization. Then, samples were centrifuged (3 min, 2000 rpm, RT) and washed with PBS, after which the cells were centrifuged again (3 min, 2000 rpm, RT) and the supernatant was carefully removed. The resulting precipitate contained cells, which were used for flow cytometry experiments.

The fraction of dead cells after treatment with the test compounds were detected using the FITC Annexin-V Apoptosis Detection Kit with PI (BioLegend, San Diego, CA, USA). First, cells were suspended in 50 μL of cold Annexin-V Binding Buffer (BioLegend, San Diego, CA, USA) and incubated in the dark with 2.5 μL of antibody Annexin-V FITC and 10 μL of propidium iodide (PI) at 37 °C for 20 min. 250 μL of the Annexin-V Binding Buffer was then added and the samples were incubated in the dark on ice for 15 min. Cytometric analyses were performed using an Aria III flow cytometer (Becton Dickinson, Franklin Lakes, NJ, USA) with the FITC configuration (488 nm excitation; emission: LP mirror 503, BP filter 530/30) or PE configuration (547 nm excitation; emission: 585 nm) and at least 10,000 cells were counted.

For cell cycle analysis, the cells were suspended in 300 μL of hypotonic buffer (consisting of: sodium citrate dihydrate 1 g/L, propidium iodide (PI) 1 mg/mL, RNAse A 10 mg/mL (EURx, Gdańsk, Poland), Triton X-100 1:9), mixed gently and incubated in the dark at room temperature for 20–30 min. Cytometric analyses were performed immediately using an Aria III flow cytometer (Becton Dickinson, Franklin Lakes, NJ, USA), using at least 10,000 cells per sample. The cytofluorimetric configurations (PE) used are described above.

The experiment was conducted in three independent repetitions. The obtained results were analyzed using Flowing Software (Cell Imaging Core, Turku Center for Biotechnology, Turku, Finland).

#### 3.3.6. Intercalation Study

For the DNA binding studies, all experiments were carried out in molecular biology grade Tris-HCl buffer at pH 7.5 (EURx, Poland). The purity of the calf thymus DNA (ctDNA) (Sigma-Aldrich, St. Louis, MO, USA) was determined by measuring the ratio of UV absorbance at 260 and 280 nm. Here, the ratio was in the range of 1.8–1.9, indicating the DNA was sufficiently free from protein. The concentration of ctDNA was determined from the absorbance at 260 nm and using a molar absorption coefficient ε = 6600 M^−1^ cm^−1^ [68]. The glycoconjugates **A-2** and **E-2** were dissolved in DMSO to a concentration of 10 mM, followed by diluting with Tris-HCl buffer to a concentration of 1 mM, which were used as the stock solutions, respectively. The absorption titration experiments were carried out by keeping the concentration of the glycoconjugates at a constant concentration of 50 µM while varying the ctDNA concentration from 3 to 300 µM. The samples were incubated for 2 h at 37 °C with gently shaking and the absorption spectra were measured using a UV–Vis-NIR spectrophotometer JASCO V-570 (Tokyo, Japan) in the range of 200–400 nm.

#### 3.3.7. Statistical Analysis

For biological evaluation, at least three replicates were performed for every kind of experiment. The results were presented as means ± standard deviation (SD). The statistical analysis was based on a *t*-test, and a *p*-value less than 0.05 was considered statistically significant, indicated on the figures with an asterisk (*).

## 4. Conclusions

Focusing on the characteristic feature of cancer, which is increased demand for glucose, an anti-cancer treatment strategy based on the use of GLUT transporters for the targeted transport of drugs to the tumor cell has been developed. This strategy is a promising way to avoid the systemic toxicity of potential pharmaceuticals.

First, the synthesis pathways of sugar derivatives functionalized at the C-6 position were designed. d-Glucose, d-galactose, as well as glucuronic acid, and trehalose were used for this purpose. New glycoconjugates were prepared by the copper(I)-catalyzed 1,3-dipolar azide-alkyne cycloaddition between sugar derivatives and appropriately functionalized 8-hydroxyquinoline derivatives. The structures of all created products were confirmed using spectroscopy of nuclear magnetic resonance (^1^H NMR and ^13^C NMR) and high-resolution mass spectrometry (HRMS). Glycoconjugates were assessed in vitro for their cytotoxic activity against HCT-116 and MCF-7 cell lines, as well as against healthy NHDF-Neo cells. The results show that the most active are glycoconjugates derivatives of d-glucose in which the triazole-quinoline was attached through the triazole nitrogen atom to the d-glucose unit directly to the carbon at the C-6 position. These glycoconjugates are more cytotoxic and selective than the analogous glycoconjugates formed by the C-1 anomeric position of d-glucose. Following the use of an EDG inhibitor and partial inhibition of GLUT activity, probably decreased compound uptake occurred, which resulted in less accumulation in cells and less anti-cancer activity. Additionally, glycoconjugates containing an unprotected glucose residue are more selective compared to the derivative with an acetylated sugar moiety. This proves the use of GLUT proteins in the transport of glycoconjugates to cancer cells. Compounds with acetyl protection in the sugar part are characterized by high lipophilicity, so this type of compound prefers passive transport across the cell membrane. This transport is non-selective, so these compounds also damage healthy cells. The results of the apoptosis and cell cycle analyses by flow cytometry confirmed the anticancer potential of the tested compounds. Experiments have shown that the new type of glycoconjugates shows pro-apoptotic properties, without causing inflammatory processes and without significantly affecting changes in the distribution of the cell cycle. The ability of glycoconjugates to interact with DNA by intercalation, which may subsequently induce damage to nucleic acids, was also confirmed. Moreover, glycoconjugates were able to reduce the clonogenic potential of cancer cells and inhibit their migration process.

In conclusion, the glycoconjugates formed through the C-6 position of d-glucose should be considered promising in therapy for the targeted transport of drugs into the tumor cell. It has been proven that the new type of glycoconjugates shows an increased affinity for GLUT, which means that they can be transported directly to tumor cells, avoiding systemic toxicity. The strategies for the synthesis of sugar derivatives using the C-6 position developed here may be used in the further design of new active sugar derivatives.

## Figures and Tables

**Table 1:** Summary of cytotoxicity of substrates used for glycoconjugation and metabolites of sugar derivatives.

Compound	Activity IC_50_ [µM] ^a^	
	HCT-116 ^b^	MCF-7 ^c^
**5**	>800	201.12 ± 2.09
**6**	>800	>800
**11**	>800	>800
**12**	>800	641.24 ± 1.79
**15**	584.30 ± 9.39	713.59 ± 15.69
**16**	>800	>800
**20**	>800	>800
**23**	>800	>800
**24**	>800	95.95 ± 4.29
**25**	83.02 ± 1.83	27.27 ± 0.06
**26**	461.39 ± 1.34	244.44 ± 1.34
**29**	>800	>800
**30**	>800	>800
**31**	>800	>800
**32**	>800	>800
**33**	>800	>800
**34**	>800	>800
**35**	>800	>800
**36**	>800	>800

^a^ Cytotoxicity was evaluated using the MTT assay; ^b^ Incubation time 24 h; ^c^ Incubation time 72 h. Data are presented as the mean ± standard deviation (*n* = 3).

**Table 2:** Cytotoxicity of quinoline glycoconjugates evaluated by the MTT test.

Compound	Activity IC_50_ [µM] ^a^			
	HCT-116 ^b^	HCT-116 ^c^	MCF-7 ^c^	NHDF-Neo ^c^
**A-1**	83.51 ± 1.91	11.98 ± 0.90	52.84 ± 0.75	129.73 ± 1.49
**A-2**	191.15 ± 2.46	22.32 ± 0.70	54.92 ± 8.71	317.22 ± 9.46
**B-1**	75.08 ± 1.30	-	36.67 ± 0.03	81.42 ± 0.52
**B-2**	293.19 ± 8.00	-	155.73 ± 7.40	271.60 ± 0.35
**C-1**	173.40 ± 6.68	81.49 ± 3.36	223.67 ± 7.34	215.24 ± 3.10
**C-2**	94.24 ± 1.48	62.35 ± 0.32	157.15 ± 7.86	318.53 ± 3.09
**C-3**	>800	767.00 ± 2.39	706.60 ± 2.87	>800
**D-1**	46.26 ± 3.12	26.33 ± 0.72	30.71 ± 0.99	34.48 ± 0.39
**D-2**	53.44 ± 5.14	21.28 ± 0.94	34.04 ± 0.95	70.66 ± 0.75
**E-1**	69.00 ± 2.53	45.73 ± 1.81	57.69 ± 3.32	55.28 ± 5.08
**E-2**	212.00 ± 7.71	162.47 ± 5.97	185.34 ± 2.21	232.75 ± 9.44

^a^ Cytotoxicity was evaluated using the MTT assay; ^b^ Incubation time 24 h; ^c^ Incubation time 72 h. Data are presented as the mean ± standard deviation (*n* = 3).

**Table 3:** Selectivity index (SI) of tested compounds after 72 h incubation time.

Compound	Selectivity Index (SI) ^a^	
	HCT-116	MCF-7
**A-1**	10.83	2.46
**A-2**	14.21	5.78
**B-1**	1.08 ^b^	2.22
**B-2**	0.93 ^b^	1.74
**C-1**	2.64	0.96
**C-2**	5.11	2.03
**C-3**	-	-
**D-1**	1.31	1.12
**D-2**	3.32	2.08
**E-1**	1.21	0.96
**E-2**	1.43	1.26

^a^ Selectivity index (SI) was calculated as a ratio of the IC_50_ value for healthy cells (NHDF-Neo) and the IC_50_ value for cancer cells (HCT-116 or MCF-7); ^b^ Incubation time 24 h.

## Data Availability

Data are contained within the article.

## Supplementary Materials

The following supporting information can be downloaded at: https://www.mdpi.com/article/10.3390/molecules27206918/s1, Figures S1–S72: ^1^H NMR and ^13^C NMR spectra of all synthesized compounds; Figure S73: Representative graphs of Annexin V/PI double staining apoptosis assay; Figure S74: Representative histograms of PI-stained DNA content; Figure S75: Representative images of cells in wound healing assay.

## Sample Availability

Samples of all compounds are available from the authors.
