# Supplementary material for: Glycoconjugation of Quinoline Derivatives Using the C-6 Position in Sugars as a Strategy for Improving the Selectivity and Cytotoxicity of Functionalized Compounds
Source: Molecules. 2022 Oct 15;27(20):6918. doi: 10.3390/molecules27206918 (PMC9607644; doi:10.3390/molecules27206918)
Supplement: Supplementary file 1 [file molecules-27-06918-s001.zip › molecules-1965050-supplementary.pdf]

Supplementary Materials

Glycoconjugation of Quinoline Derivatives Using the C-6 Position in Sugars as a Strategy for Improving the Selectivity and Cytotoxicity of Functionalized Compounds

Monika Domińska [1,2,*], Gabriela Pastuch-Gawołek [1,2], Magdalena Skonieczna [2,3], Wiesław Szeja [1], Adrian Domiński [4] and Piotr Kurcok [4]

[1] Department of Organic Chemistry, Bioorganic Chemistry and Biotechnology, Silesian University of Technology, B. Krzywoustego 4, 44-100 Gliwice, Poland
[2] Biotechnology Centre, Silesian University of Technology, B. Krzywoustego 8, 44-100 Gliwice, Poland
[3] Department of Systems Biology and Engineering, Faculty of Automatic Control, Electronics and Computer Science, Silesian University of Technology, Akademicka 16, 44-100 Gliwice, Poland
[4] Centre of Polymer and Carbon Materials, Polish Academy of Sciences, M. Curie-Skłodowskiej 34, 41-819 Zabrze, Poland
* Correspondence: monika.dominska@polsl.pl; Tel.: +48-32-237-1759

1. ^{1}H NMR and ^{13}C NMR spectra of all synthesized compounds.. S1-S72
2. Representative graphs of Annexin V/PI double staining apoptosis assay S73
3. Representative histograms of PI-stained DNA content S74
4. Representative images of cells in wound healing assay S75

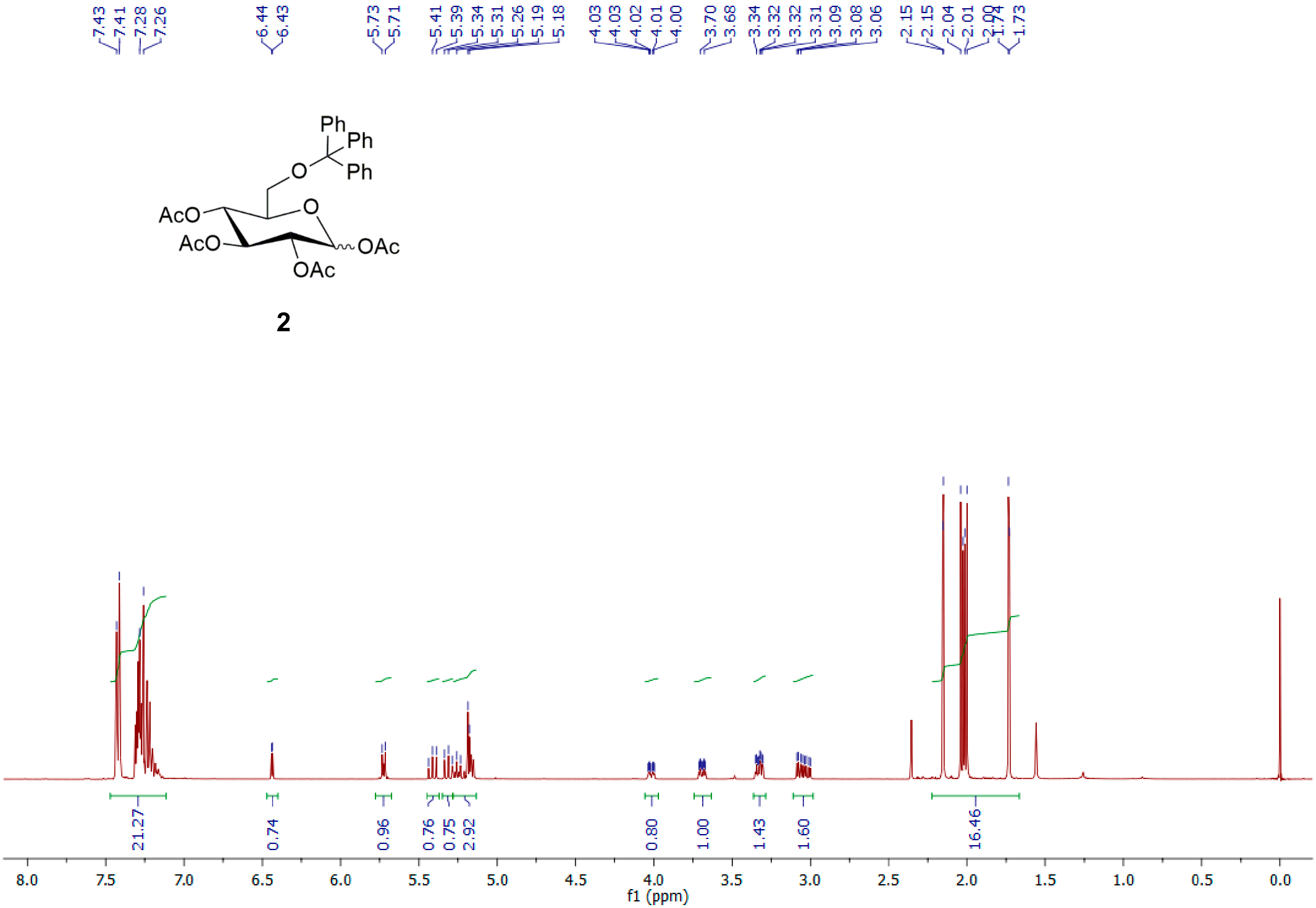

Fig. S1: ^{1}H NMR spectrum of 1,2,3,4-tetra-*O*-acetyl-6-*O*-triphenylmethyl-D-glucopyranose **2** (400 MHz/$CDCl_3$/TMS; δ (ppm)).

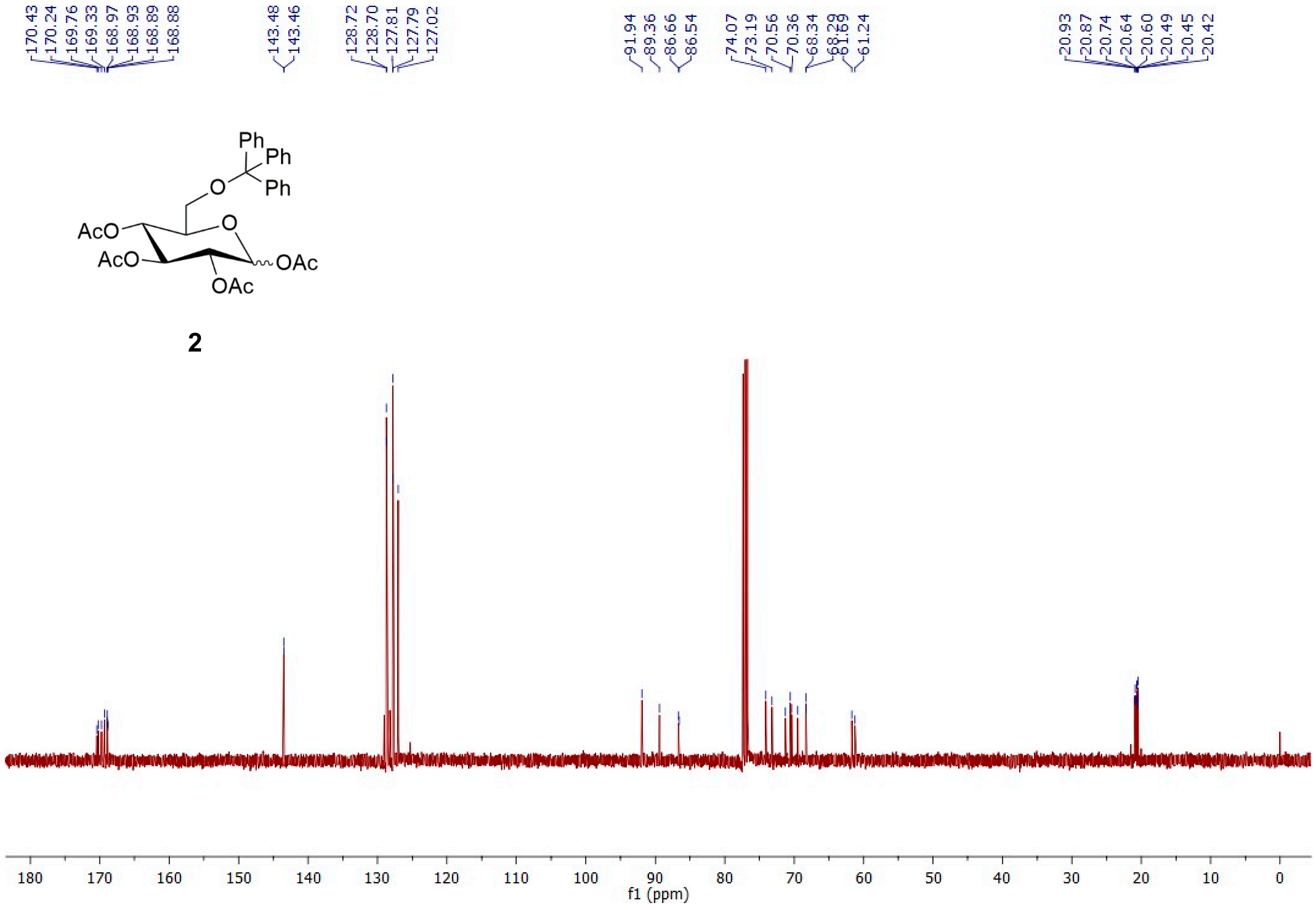

Fig. S2: ^{13}C NMR spectrum of 1,2,3,4-tetra-*O*-acetyl-6-*O*-triphenylmethyl-D-glucopyranose **2** (100 MHz/$CDCl_3$/TMS; δ (ppm)).

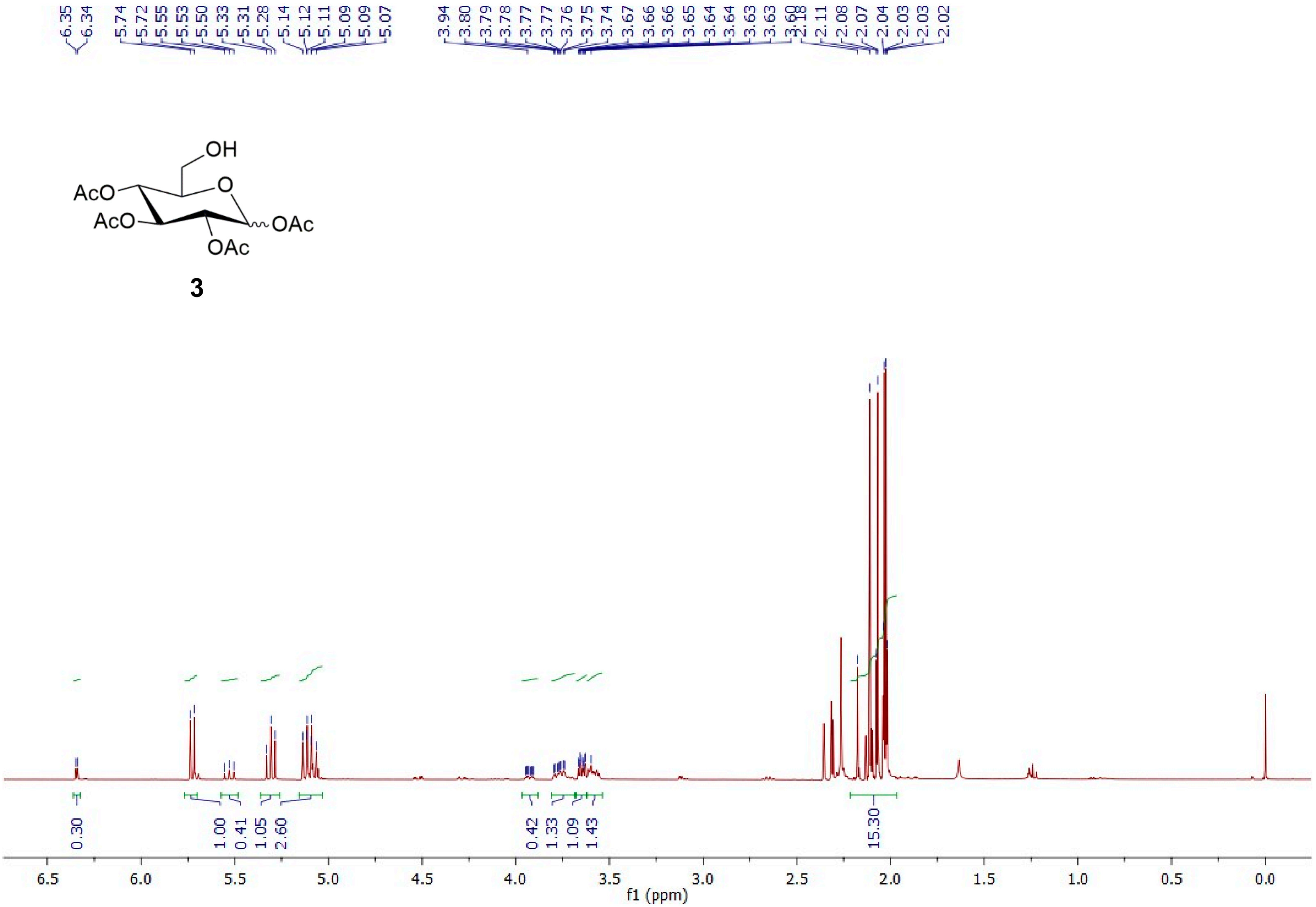

Fig. S3: ¹H NMR spectrum of 1,2,3,4-tetra-*O*-acetyl-D-glucopyranose **3** (400 MHz/$CDCl_3$/TMS; δ (ppm)).

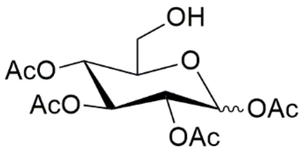

3

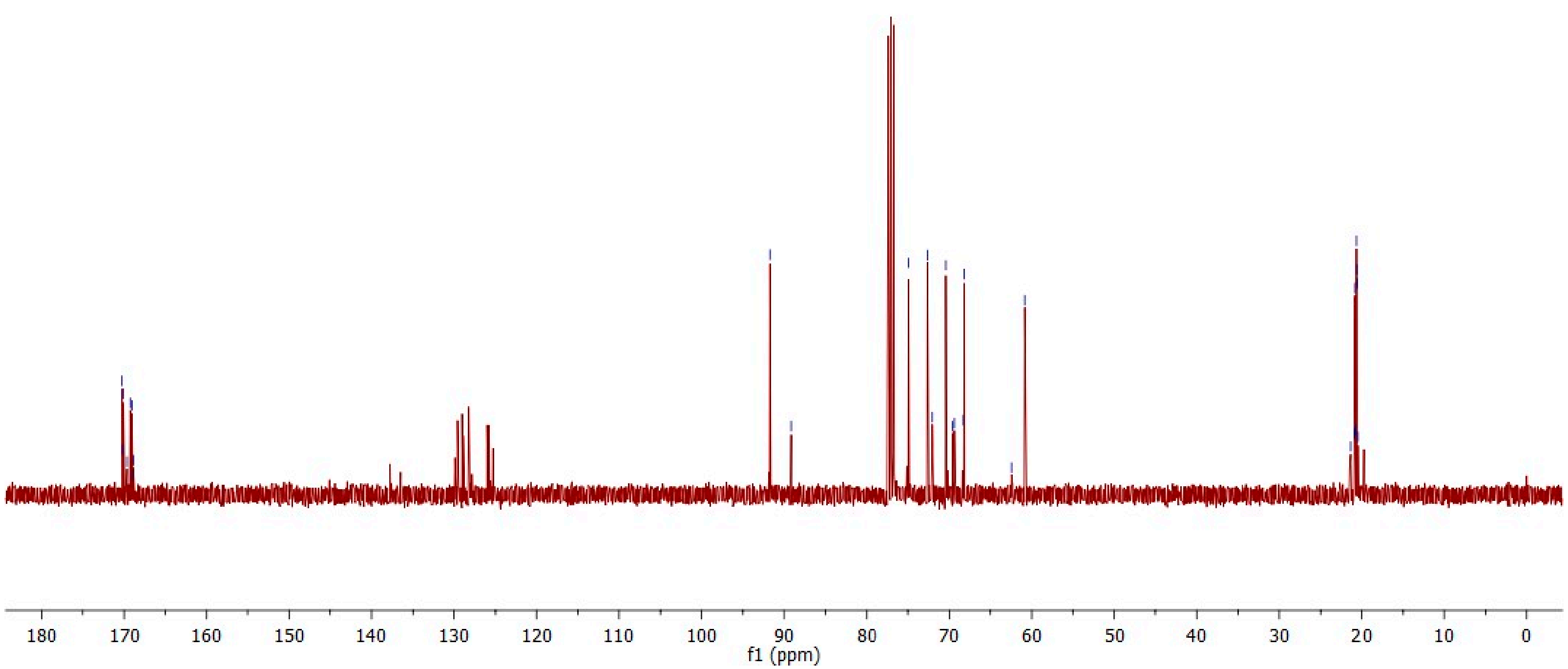

Fig. S4: ^{13}C NMR spectrum of 1,2,3,4-tetra-*O*-acetyl-D-glucopyranose **3** (100 MHz/$CDCl_3$/TMS; δ (ppm)).

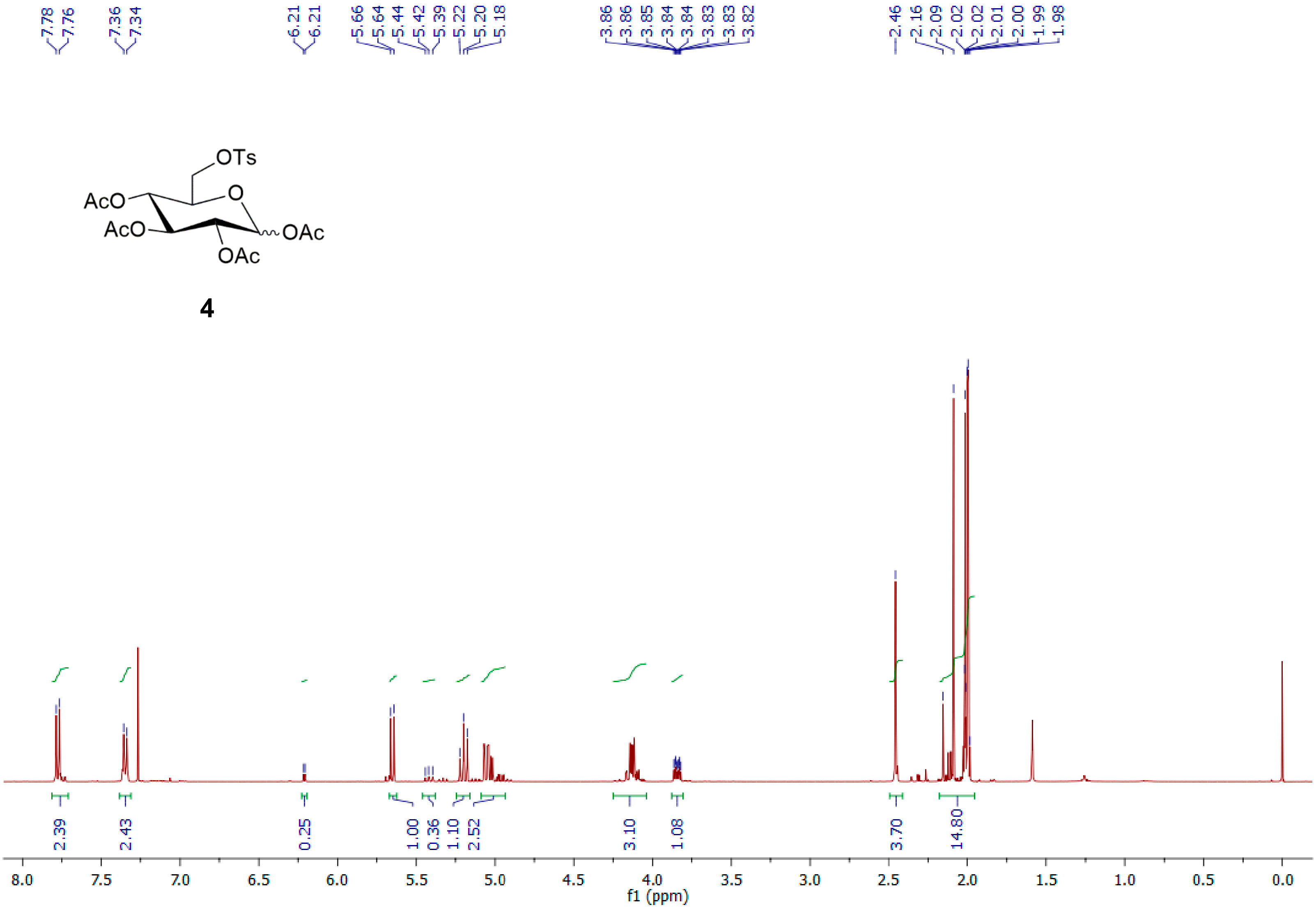

Fig. S5: ^{1}H NMR spectrum of 1,2,3,4-tetra-*O*-acetyl-6-*O*-*p*-toluenesulfonyl-D-glucopyranose **4** (400 MHz/$CDCl_3$/TMS; δ (ppm)).

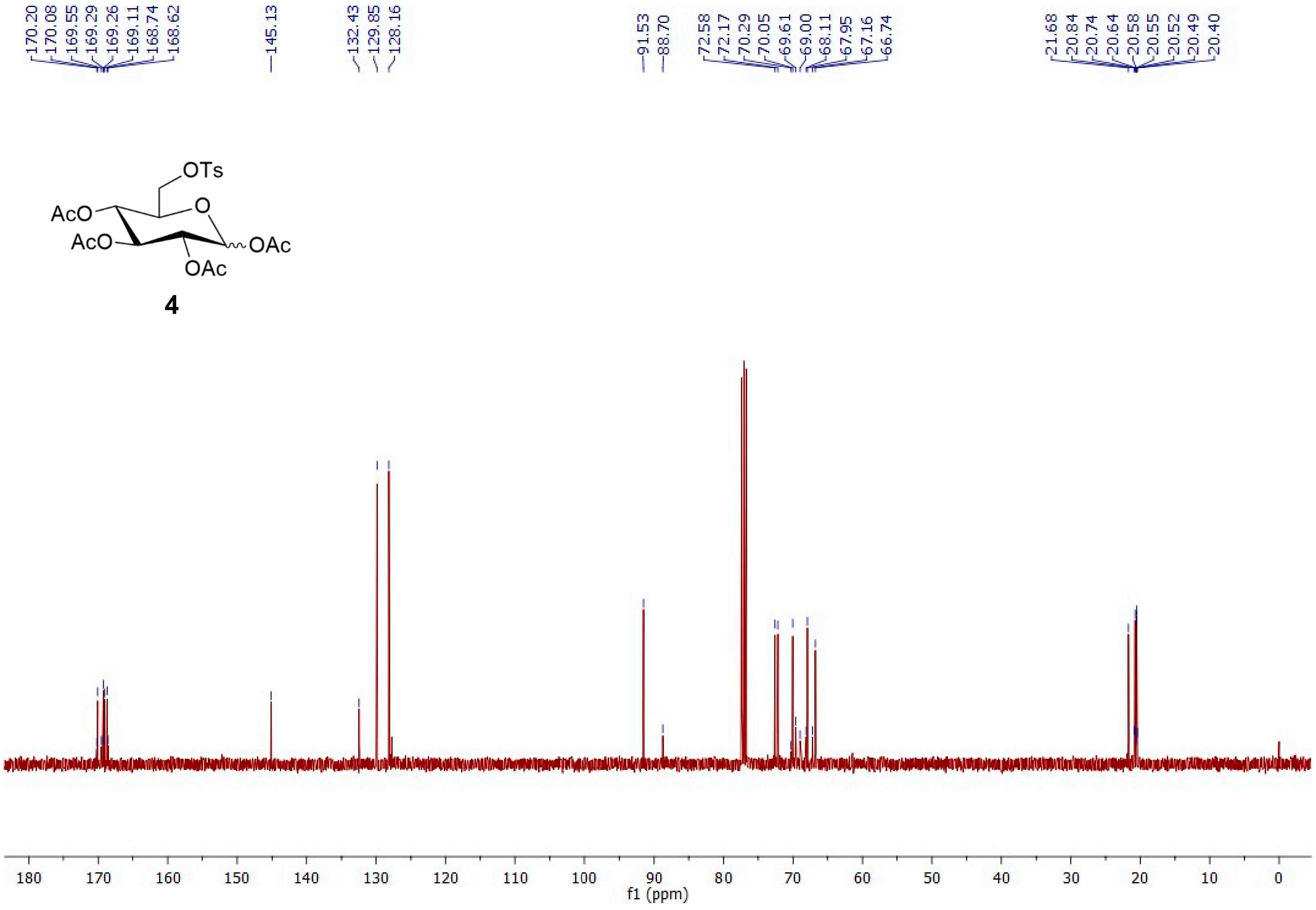

Fig. S6: ^{13}C NMR spectrum of 1,2,3,4-tetra-*O*-acetyl-6-*O*-*p*-toluenesulfonyl-D-glucopyranose **4** (100 MHz/$CDCl_3$/TMS; δ (ppm)).

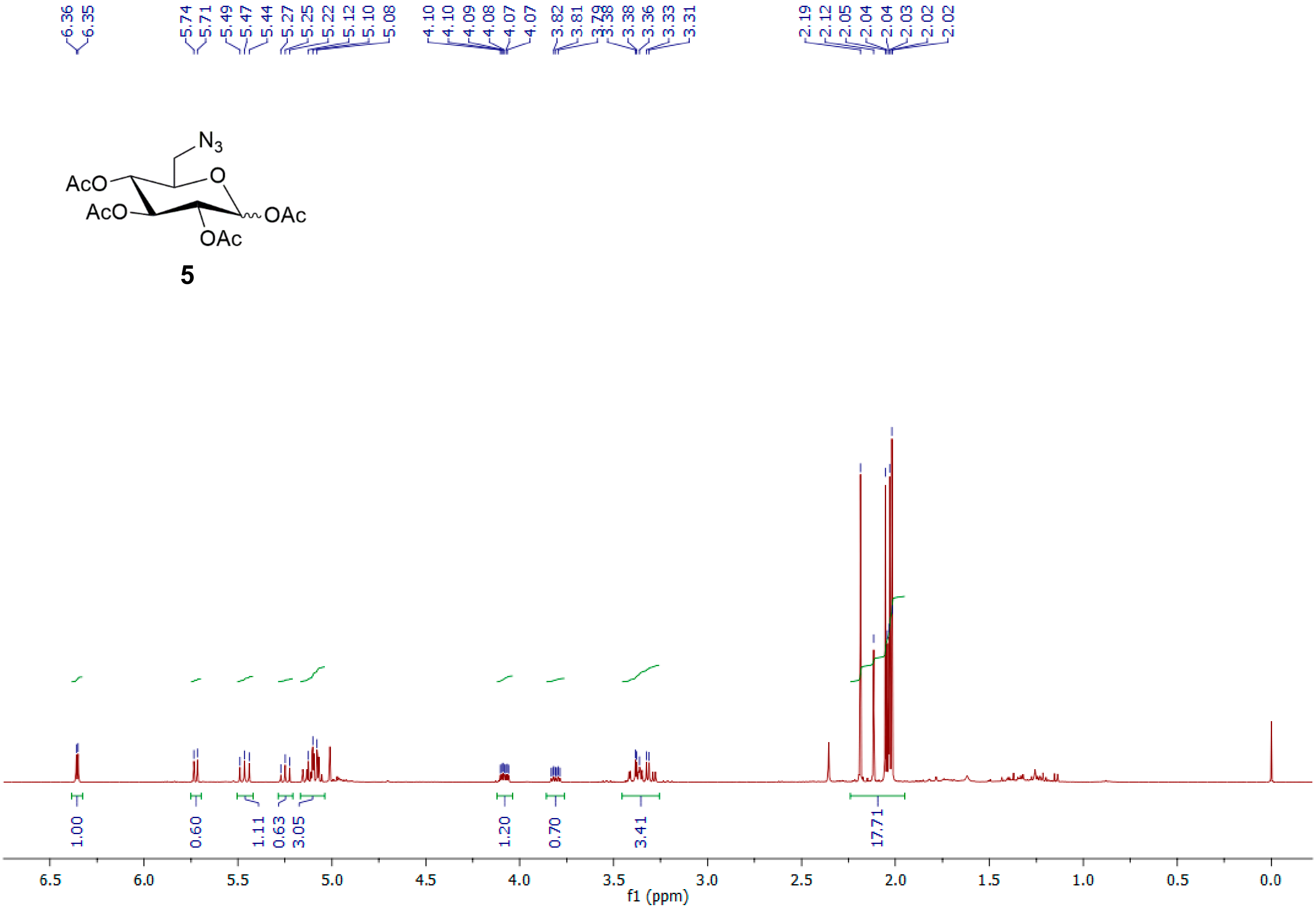

Fig. S7: ^{1}H NMR spectrum of 1,2,3,4-tetra-*O*-acetyl-6-azido-6-deoxy-D-glucopyranose **5** (400 MHz/$CDCl_3$/TMS; δ (ppm)).

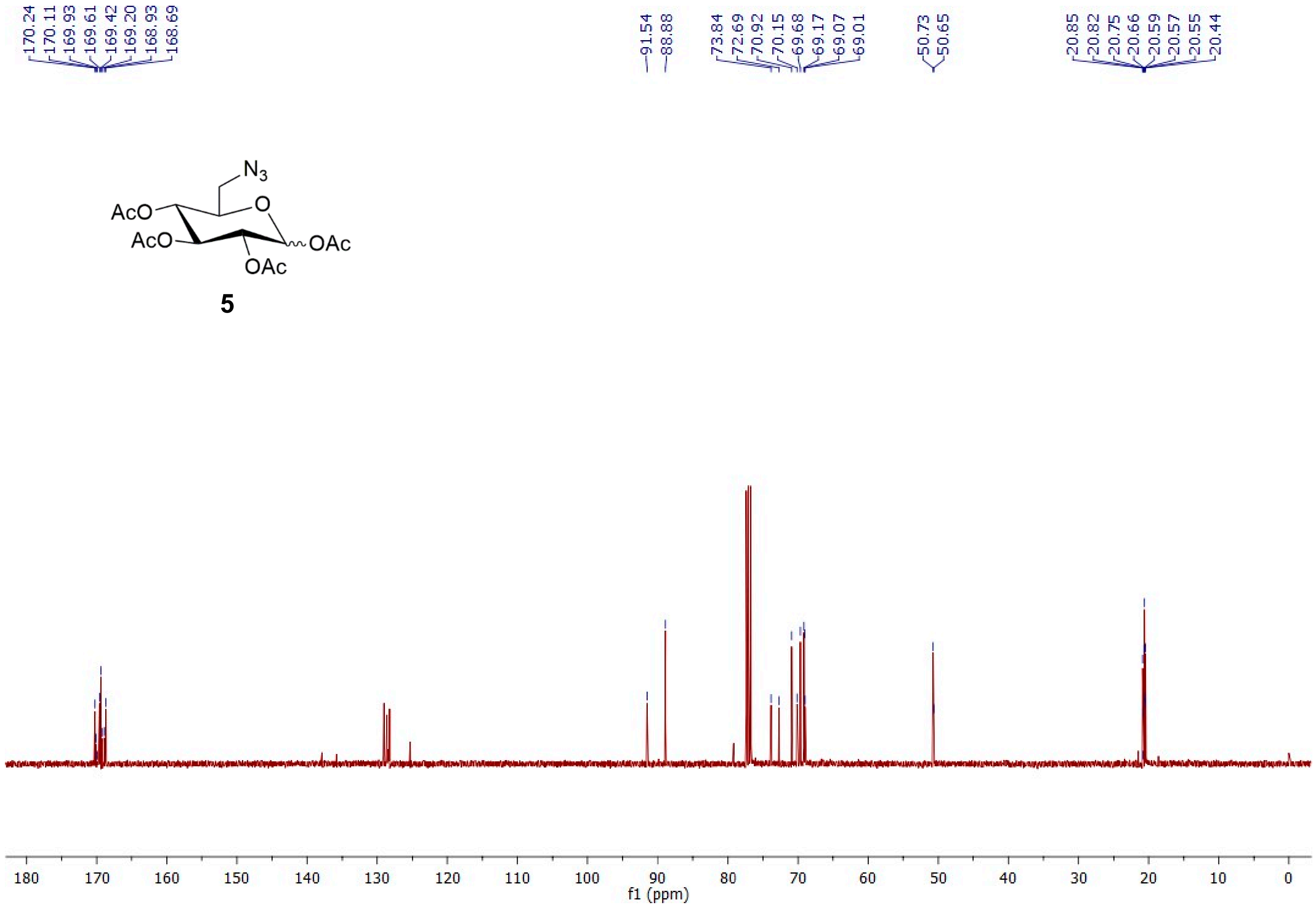

Fig. S8: 13C NMR spectrum of 1,2,3,4-tetra-*O*-acetyl-6-azido-6-deoxy-D-glucopyranose **5** (100 MHz/$CDCl_3$/TMS; δ (ppm)).

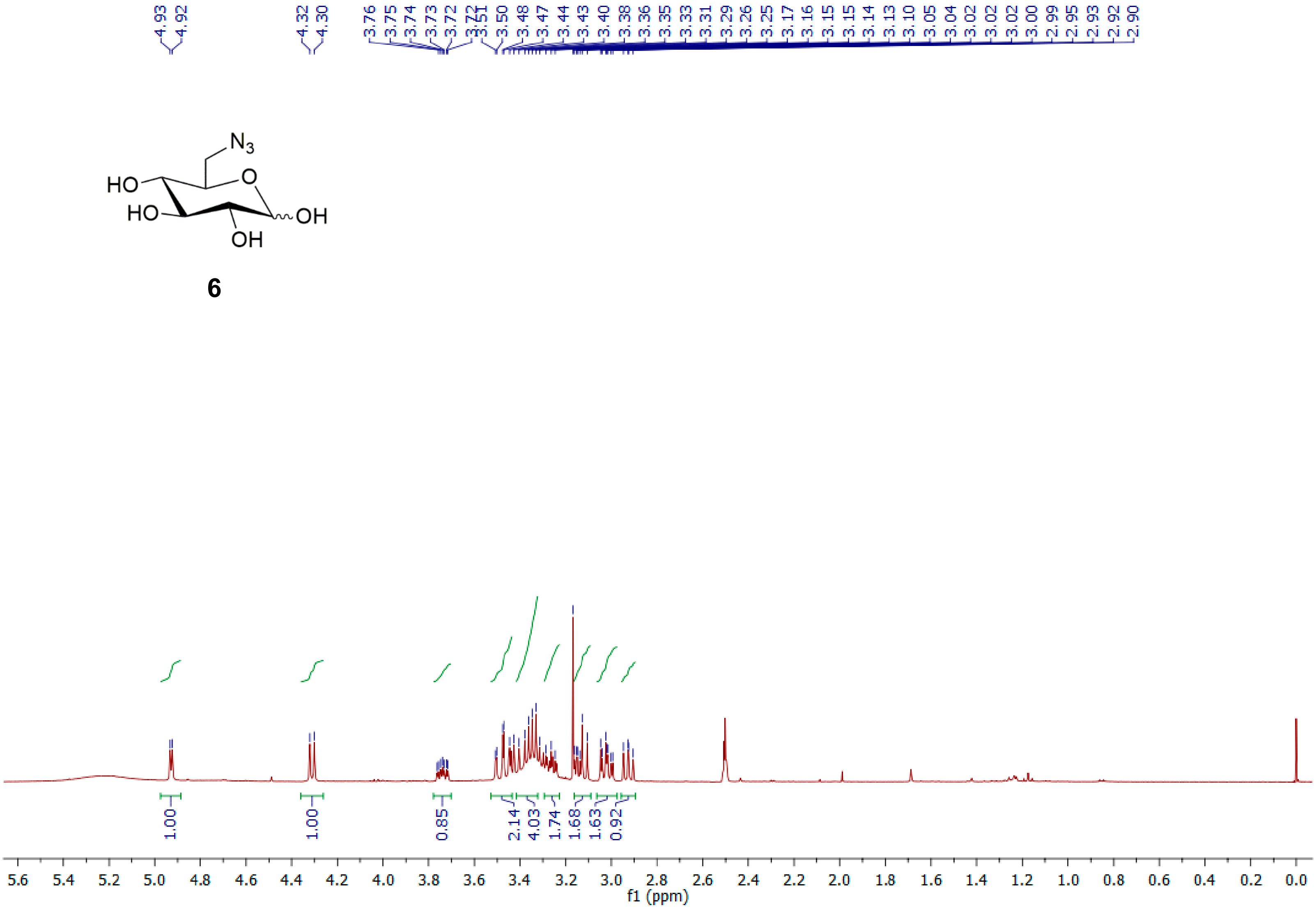

Fig. S9: ^{1}H NMR spectrum of 6-azido-6-deoxy-D-glucopyranose **6** (400 MHz/DMSO/TMS; δ (ppm)).

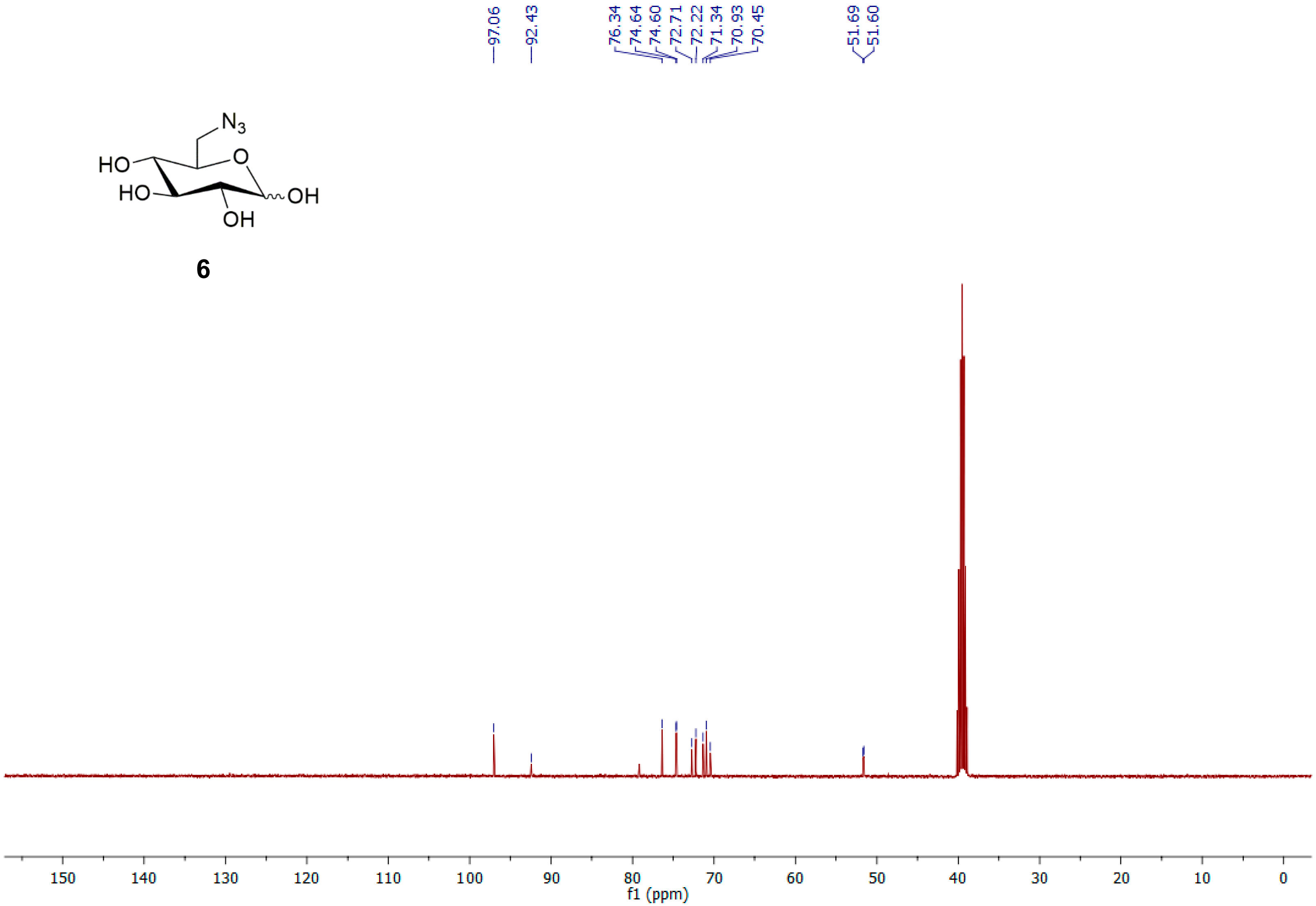

Fig. S10: ^{13}C NMR spectrum of 6-azido-6-deoxy-D-glucopyranose **6** (100 MHz/DMSO/TMS; δ (ppm)).

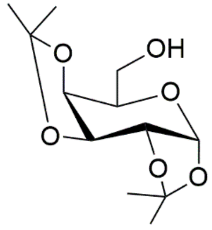

8

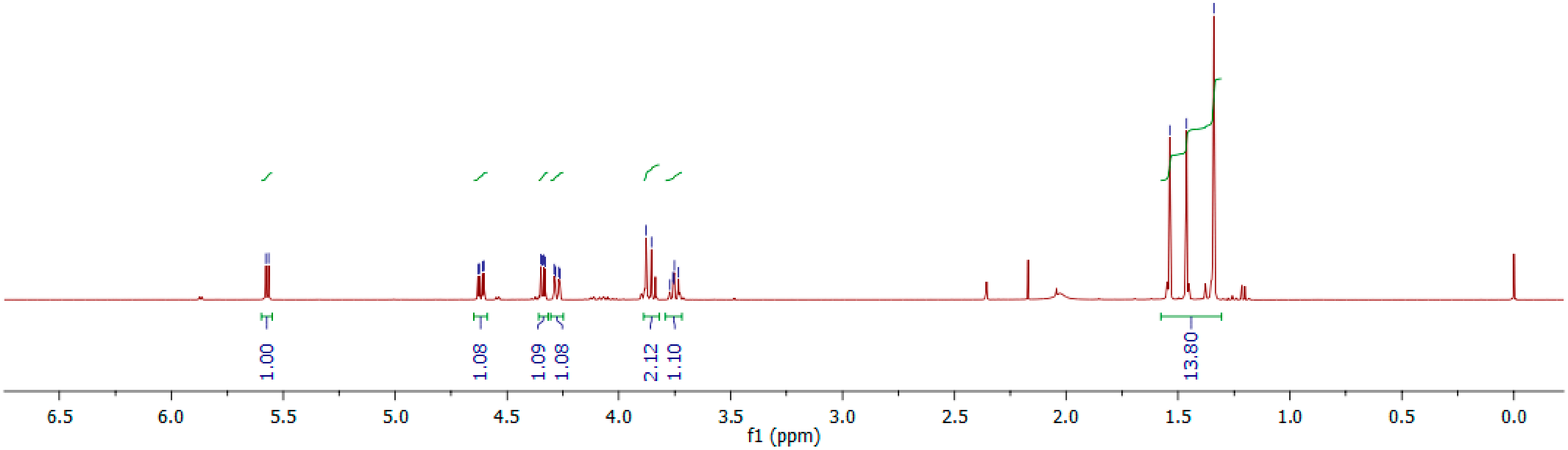

Fig. S11: ^{1}H NMR spectrum of 1,2:3,4-di-*O*-isopropylidene-α-D-galactopyranose **8** (400 MHz/$CDCl_3$/TMS; δ (ppm)).

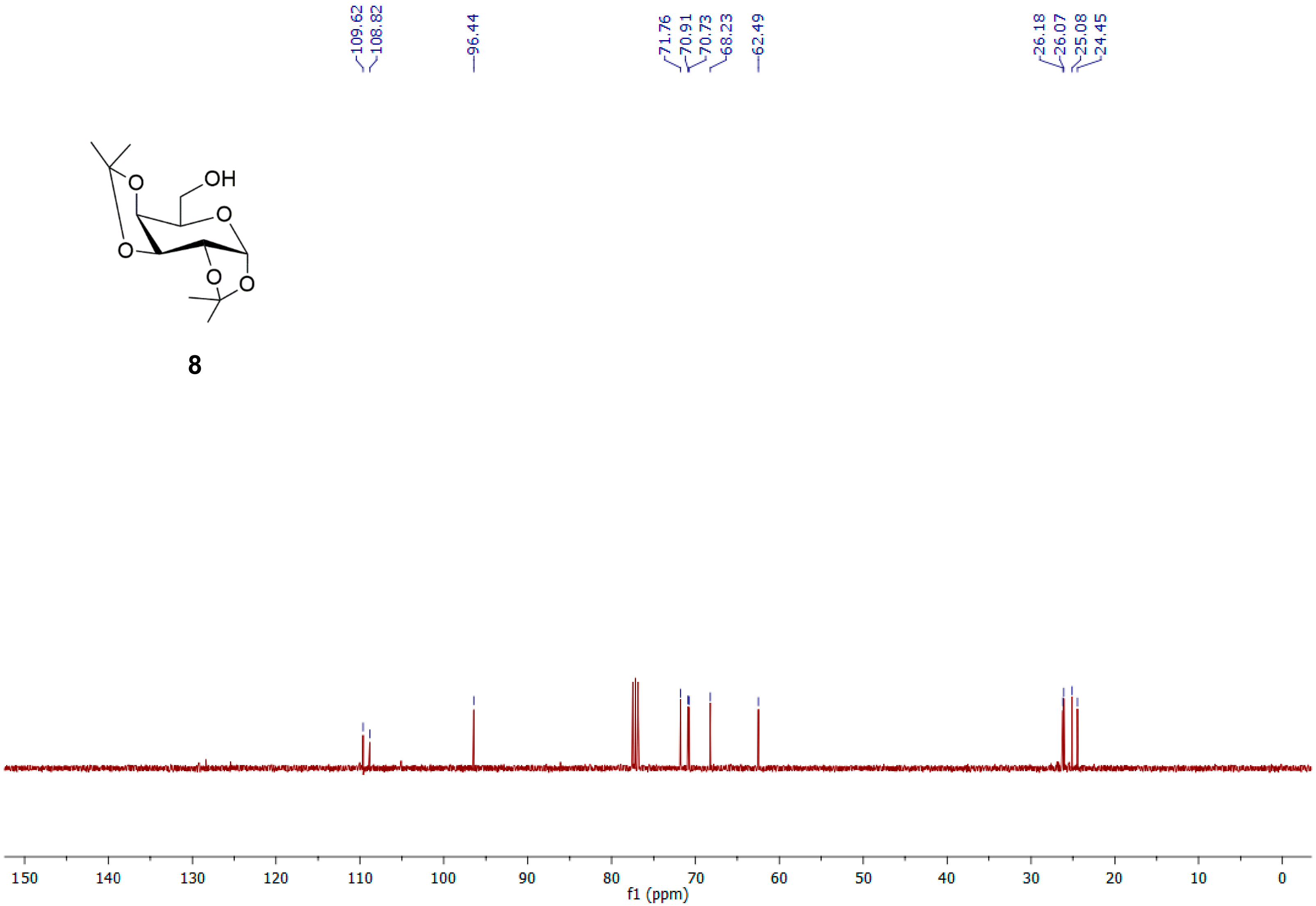

Fig. S12: ^{13}C NMR spectrum of 1,2:3,4-di-*O*-isopropylidene-α-D-galactopyranose **8** (100 MHz/$CDCl_3$/TMS; δ (ppm)).

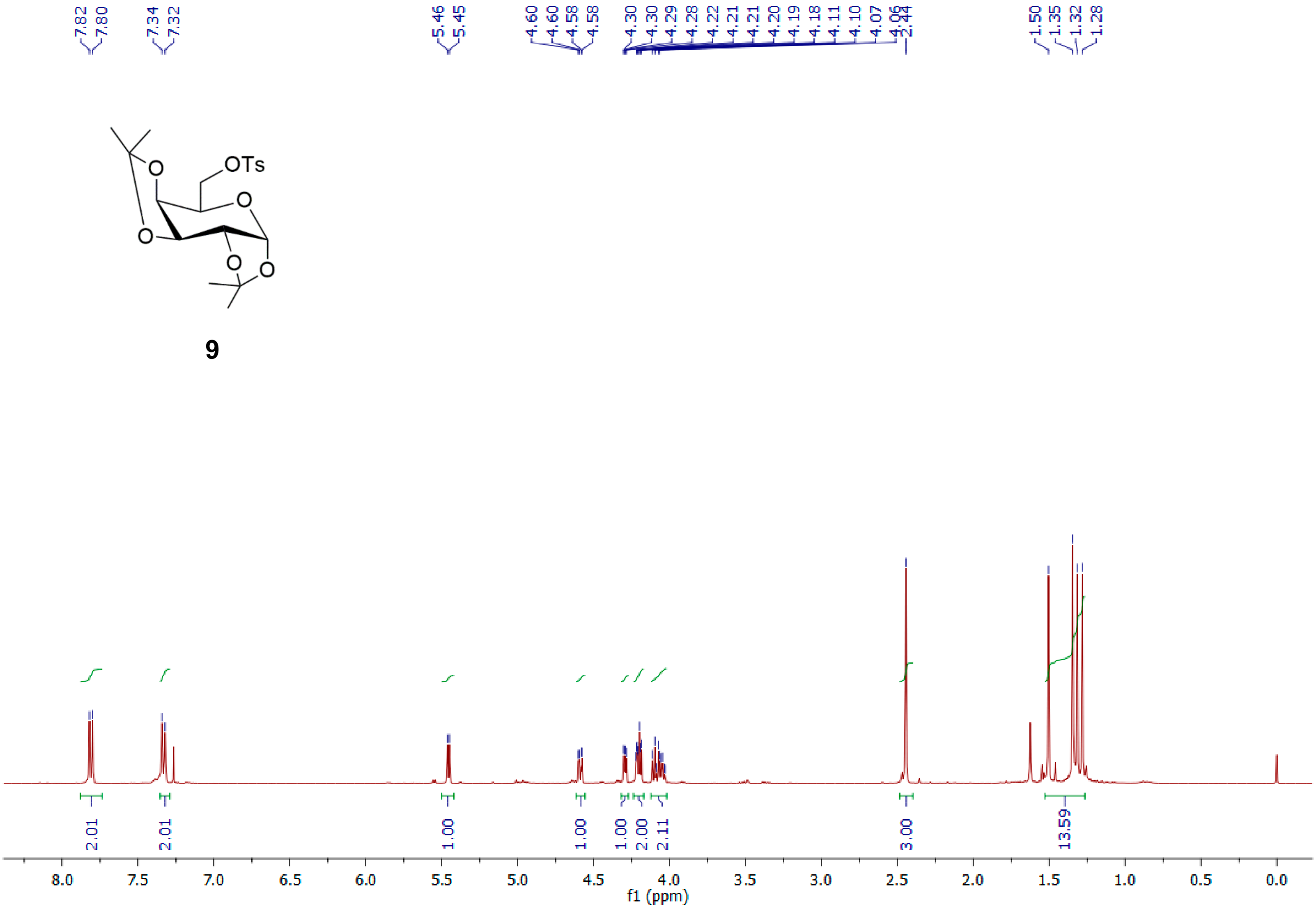

Fig. S13: ^{1}H NMR spectrum of 1,2:3,4-di-*O*-isopropylidene-6-*O*-*p*-toluenesulfonyl-α-D-galactopyranose **9** (400 MHz/$CDCl_3$/TMS; δ (ppm)).

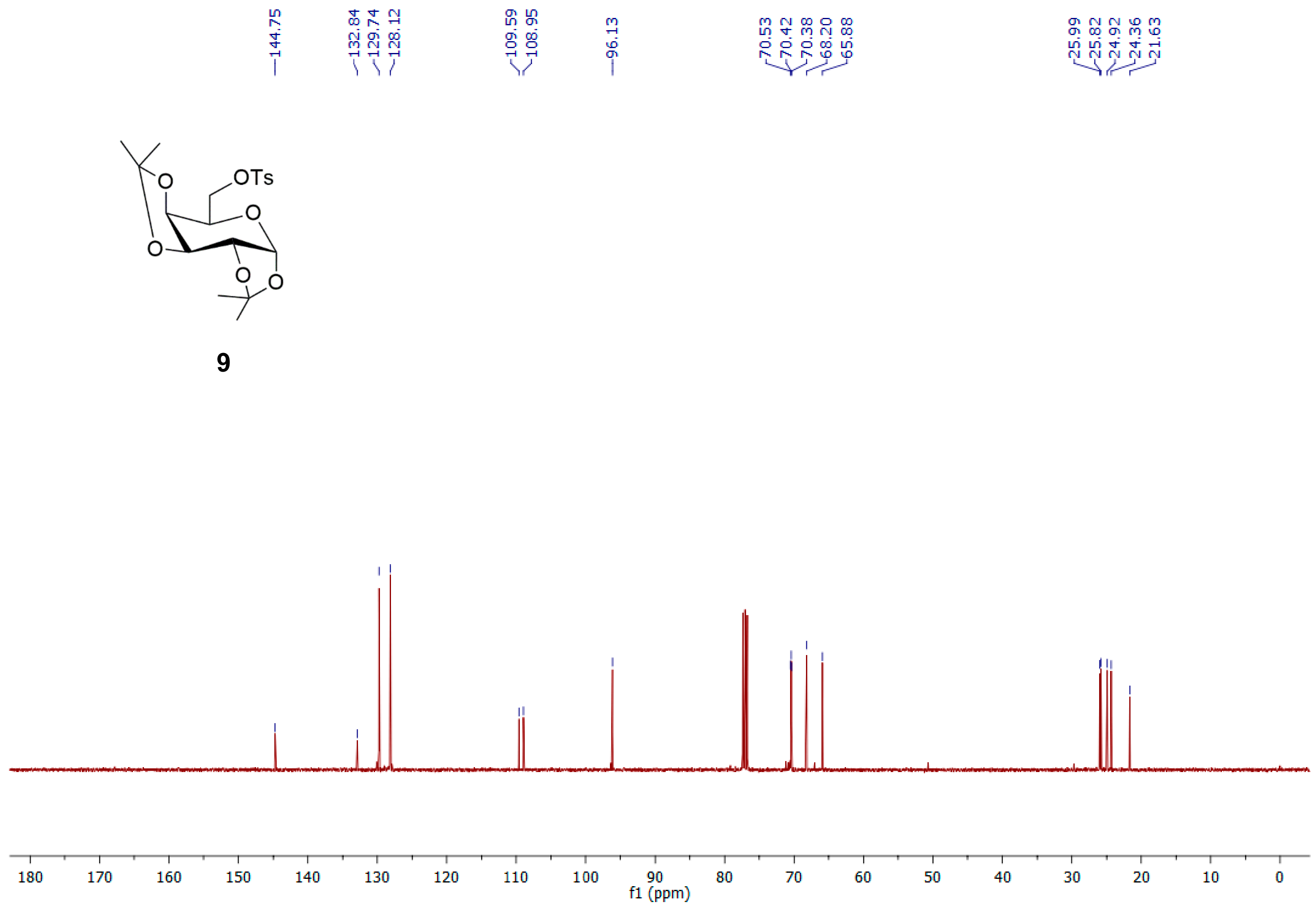

Fig. S14: ^{13}C NMR spectrum of 1,2:3,4-di-*O*-isopropylidene-6-*O*-*p*-toluenesulfonyl-α-D-galactopyranose **9** (100 MHz/$CDCl_3$/TMS; δ (ppm)).

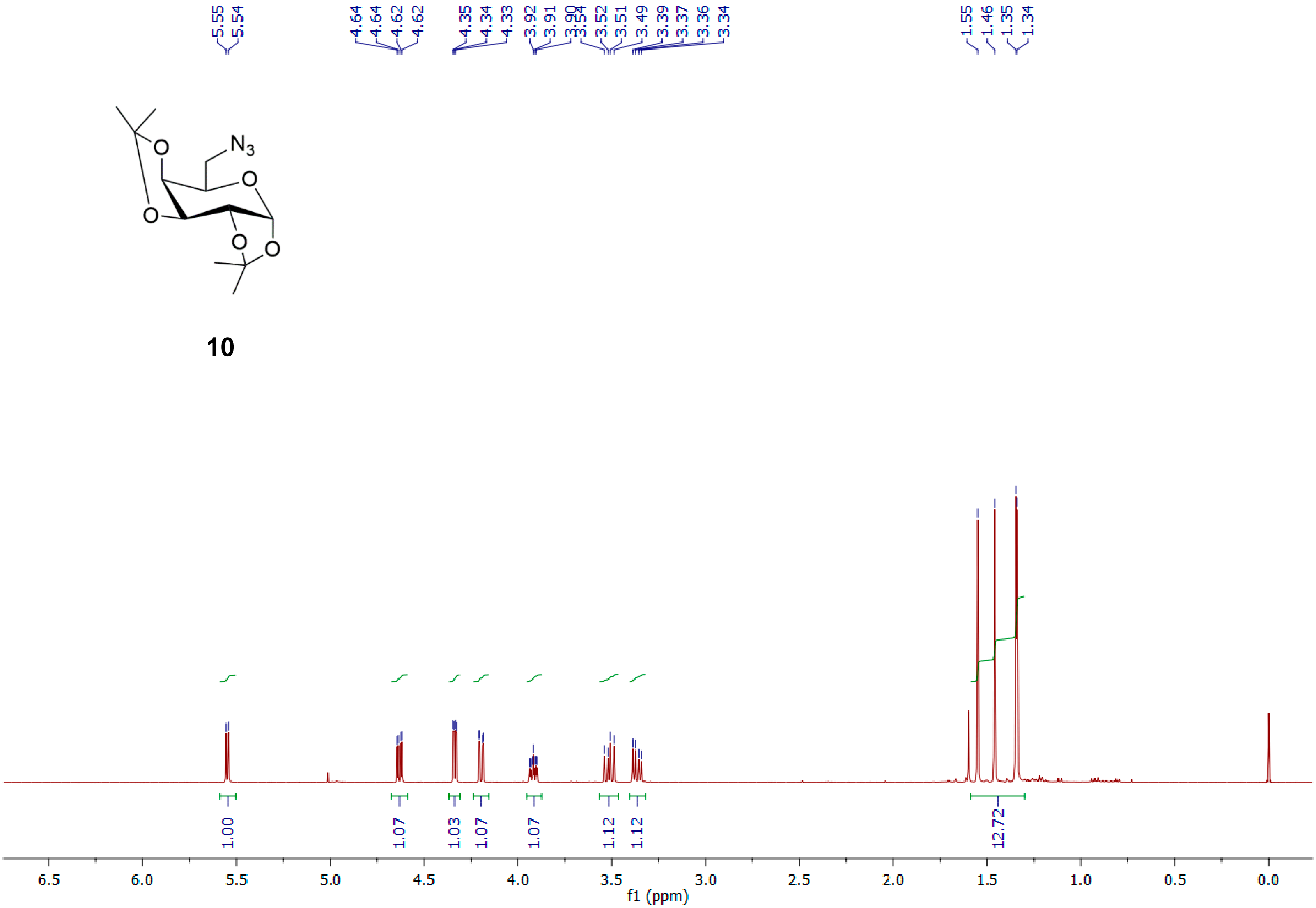

Fig. S15: ^{1}H NMR spectrum of 1,2:3,4-di-*O*-isopropylidene-6-azido-6-deoxy-α-D-galactopyranose **10** (400 MHz/$CDCl_3$/TMS; δ (ppm)).

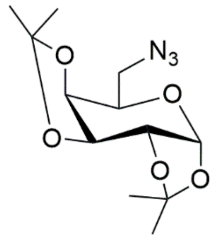

10

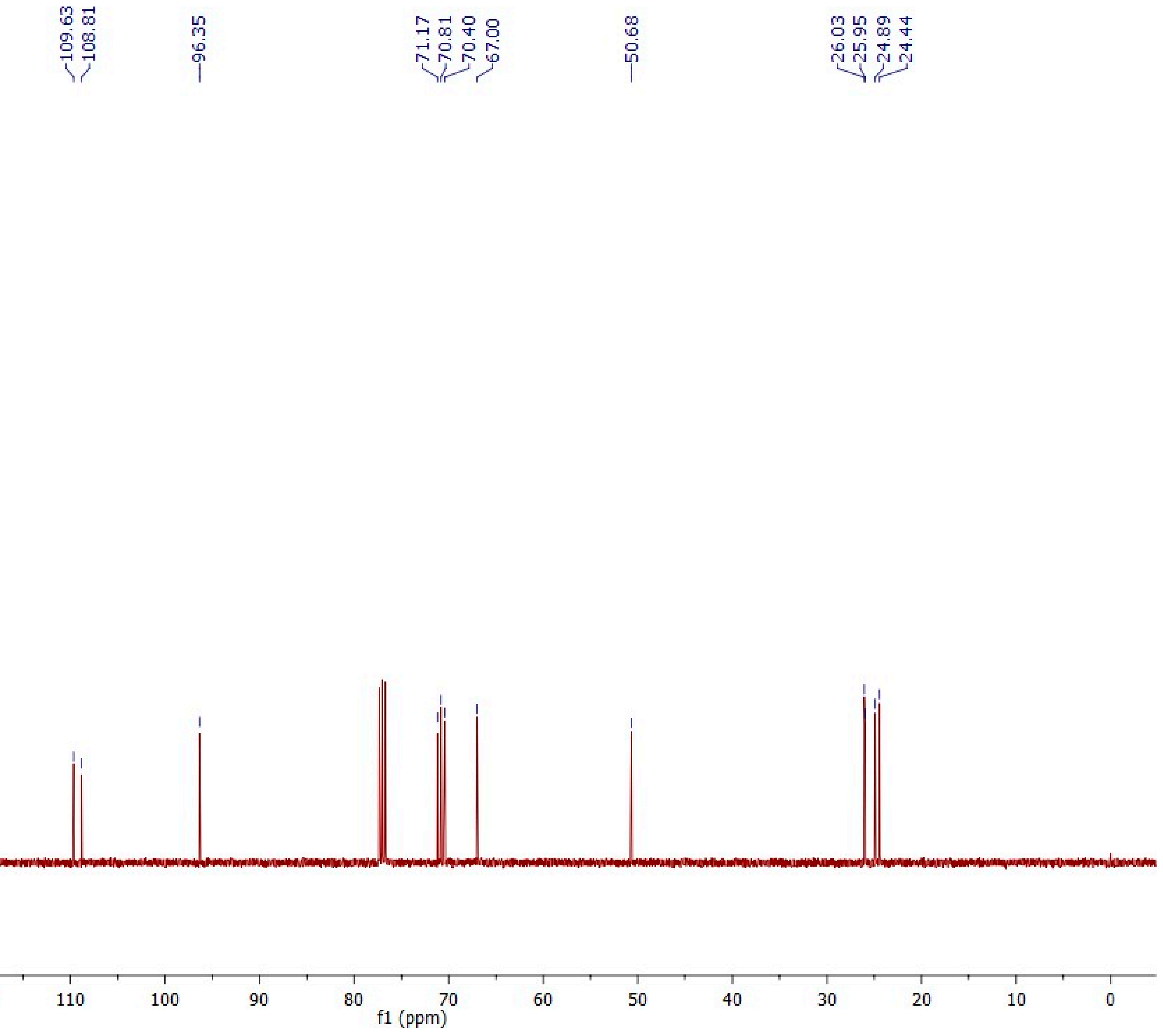

Fig. S16: ^{13}C NMR spectrum of 1,2:3,4-di-*O*-isopropylidene-6-azido-6-deoxy-α-D-galactopyranose **10** (100 MHz/CDCl$_3$/TMS; δ (ppm)).

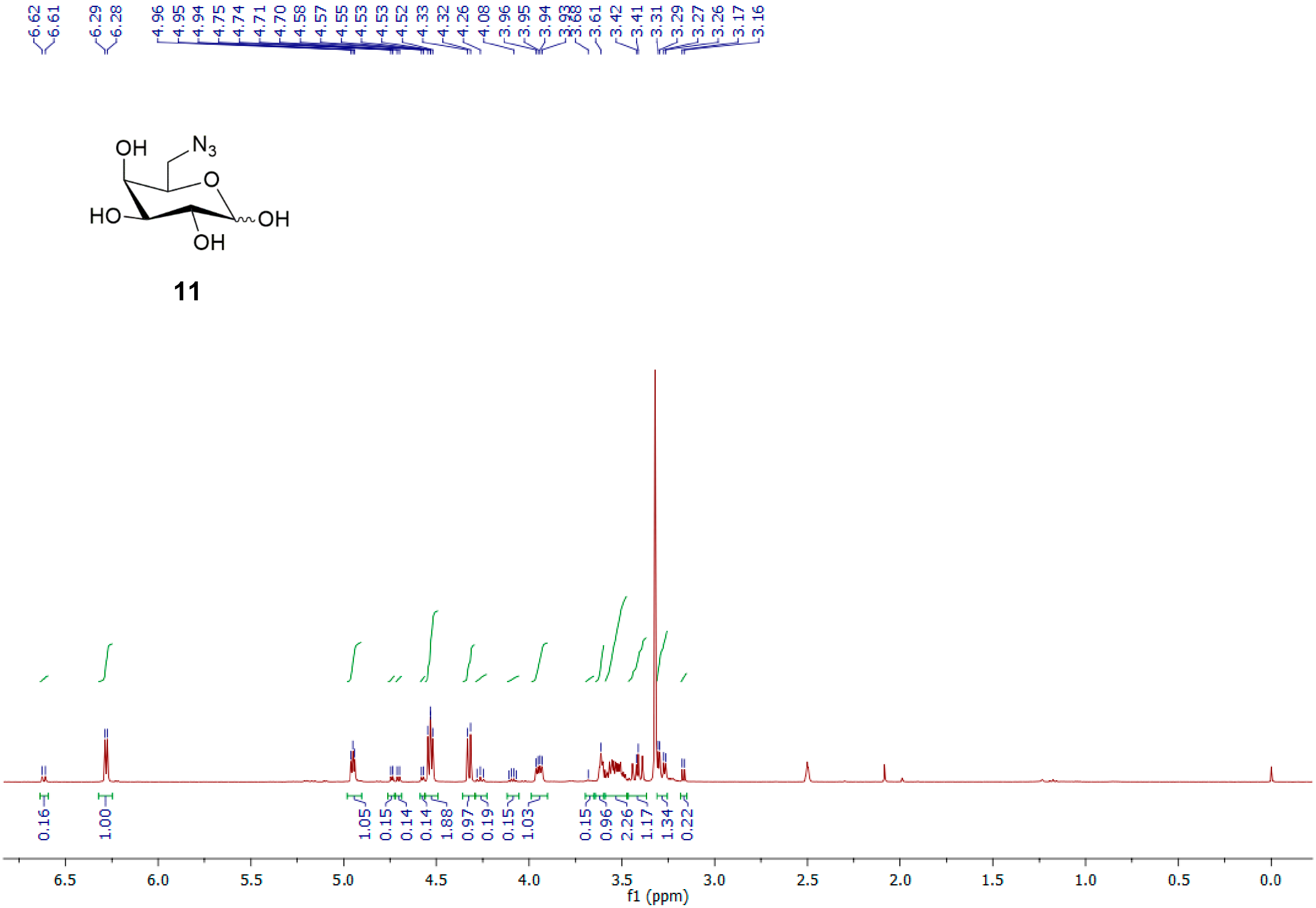

Fig. S17: ^{1}H NMR spectrum of 6-azido-6-deoxy-D-galactopyranose **11** (400 MHz/DMSO/TMS; δ (ppm)).

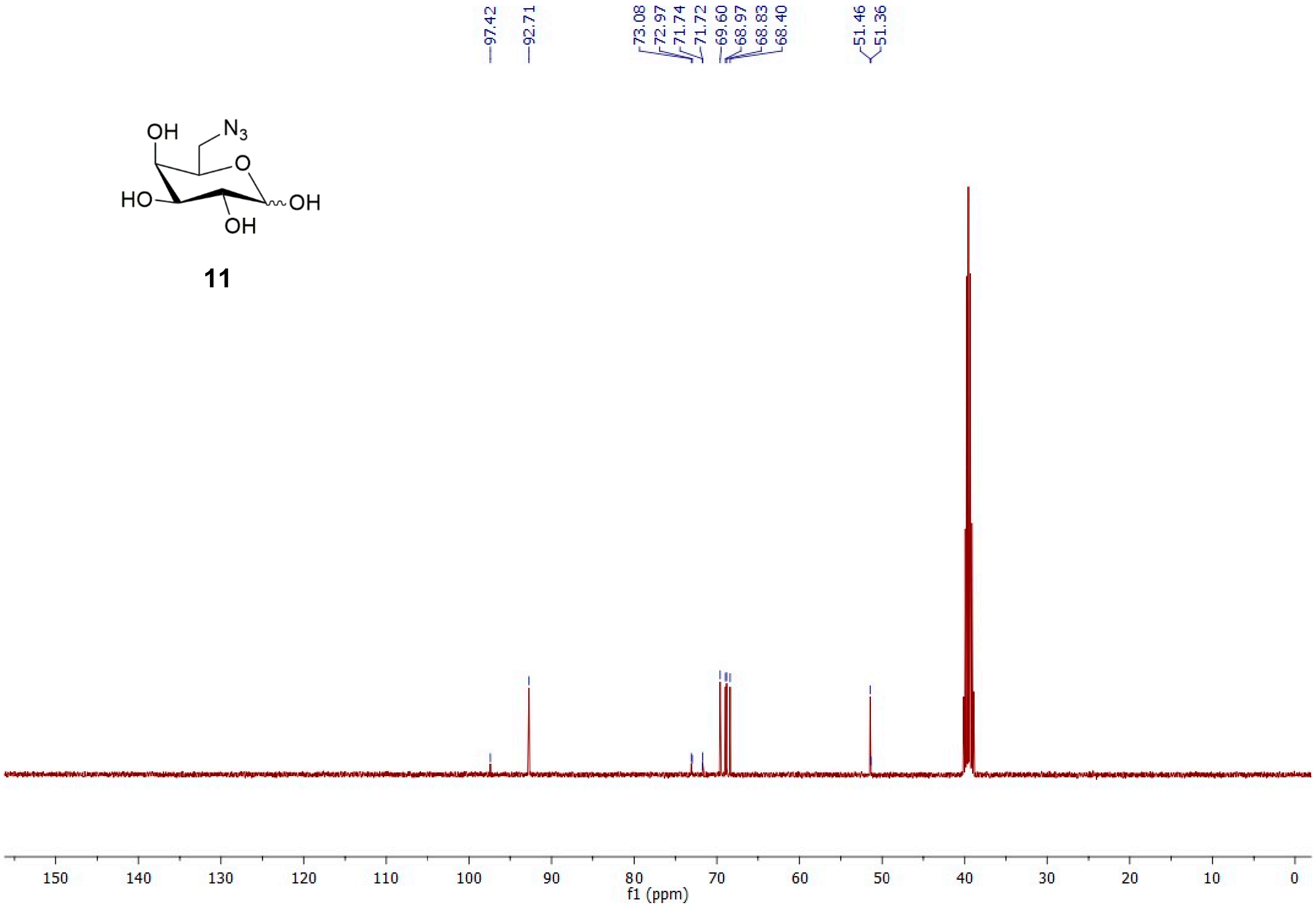

Fig. S18: ^{13}C NMR spectrum of 6-azido-6-deoxy-D-galactopyranose **11** (100 MHz/DMSO/TMS; δ (ppm)).

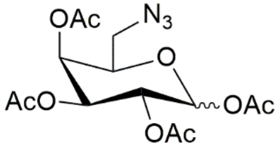

12

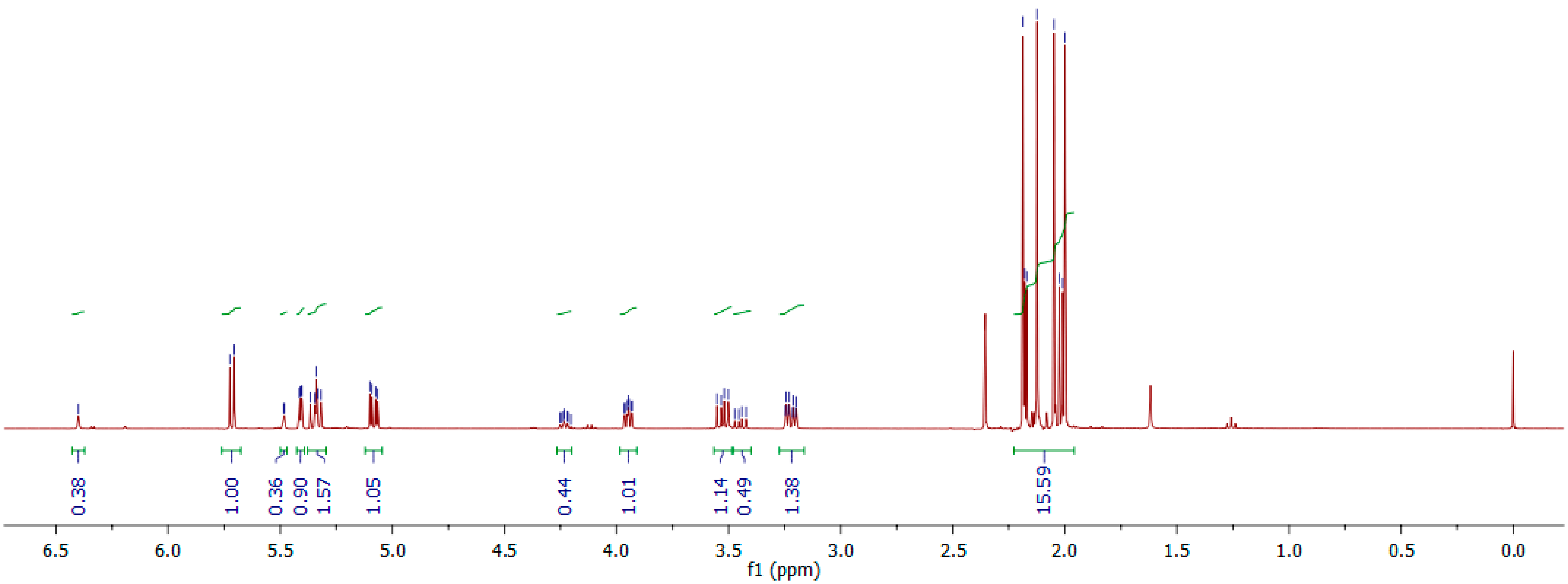

Fig. S19: ^{1}H NMR spectrum of 1,2,3,4-tetra-*O*-acetylo-6-azido-6-deoxy-D-galactopyranose **12** (400 MHz/$CDCl_3$/TMS; δ (ppm)).

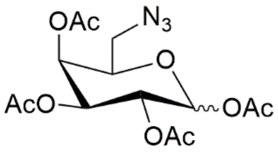

12

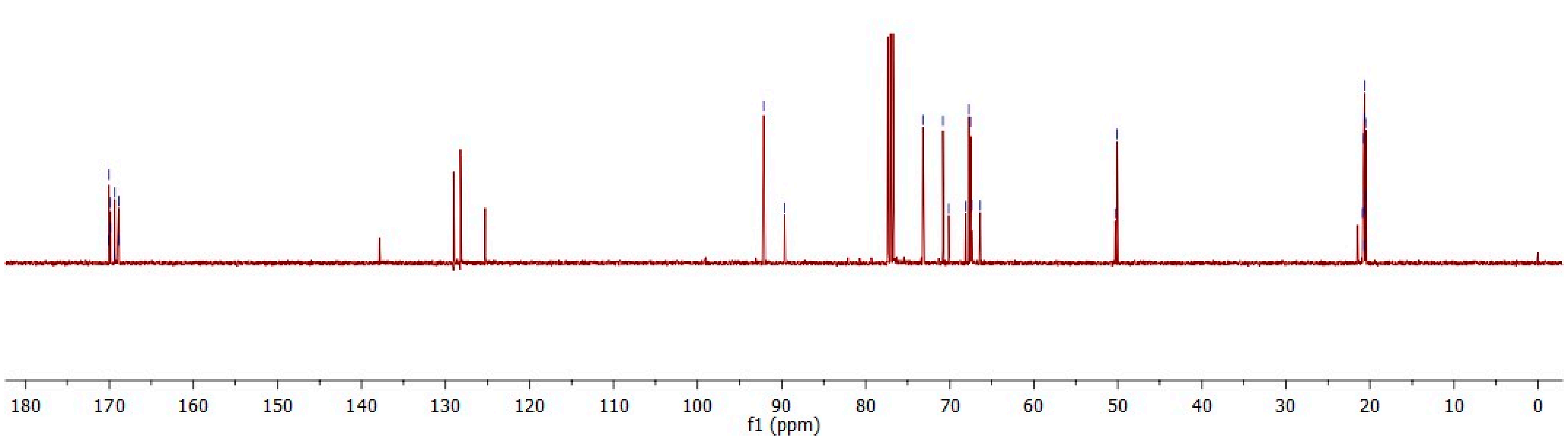

Fig. S20: ^{13}C NMR spectrum of 1,2,3,4-tetra-*O*-acetylo-6-azido-6-deoxy-D-galactopyranose **12** (100 MHz/$CDCl_3$/TMS; δ (ppm)).

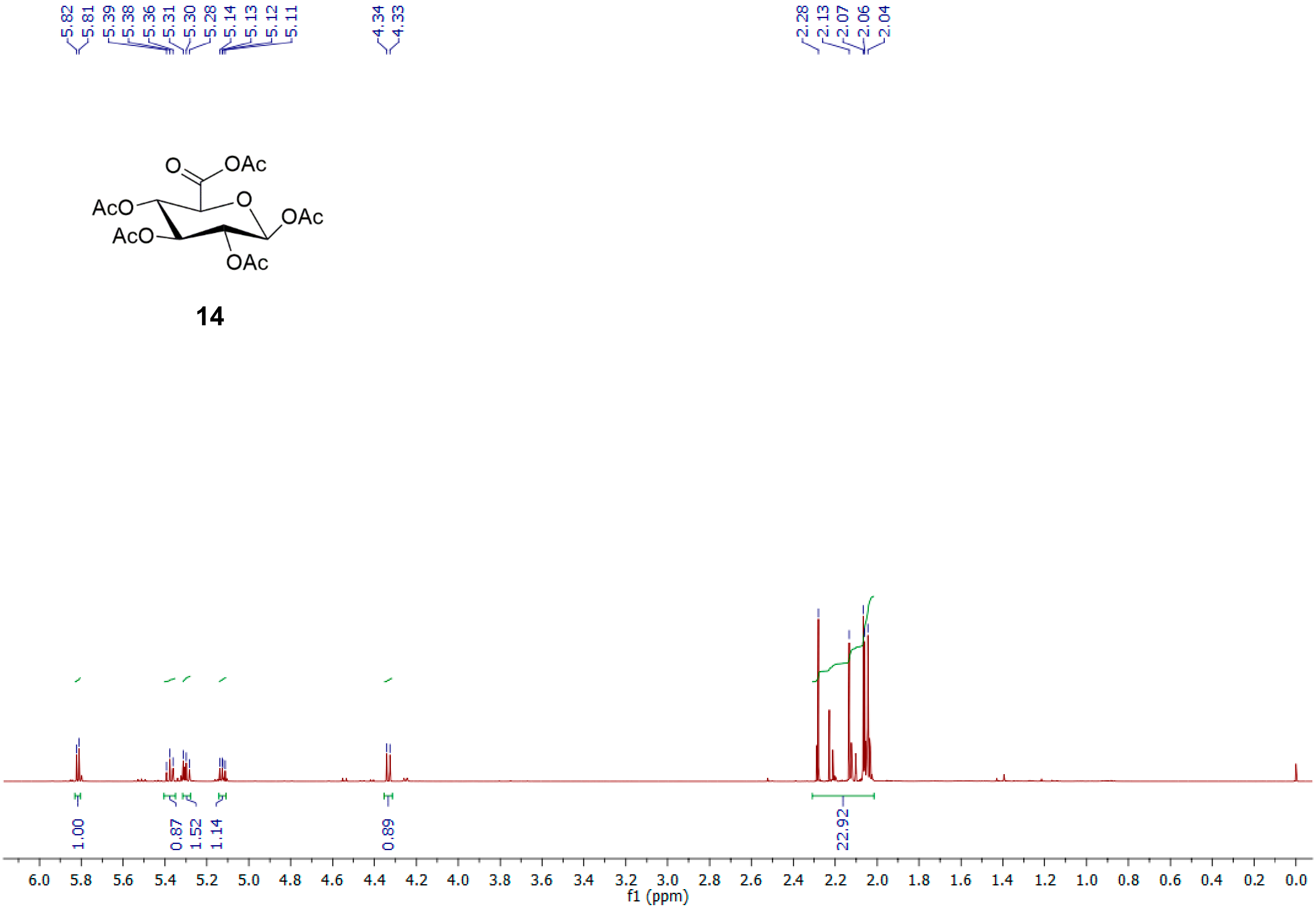

Fig. S21: ^{1}H NMR spectrum of 1,2,3,4-tetra-*O*-acetyl-β-D-glucopyranuronic acetic anhydride **14** (400 MHz/$CDCl_3$/TMS; δ (ppm)).

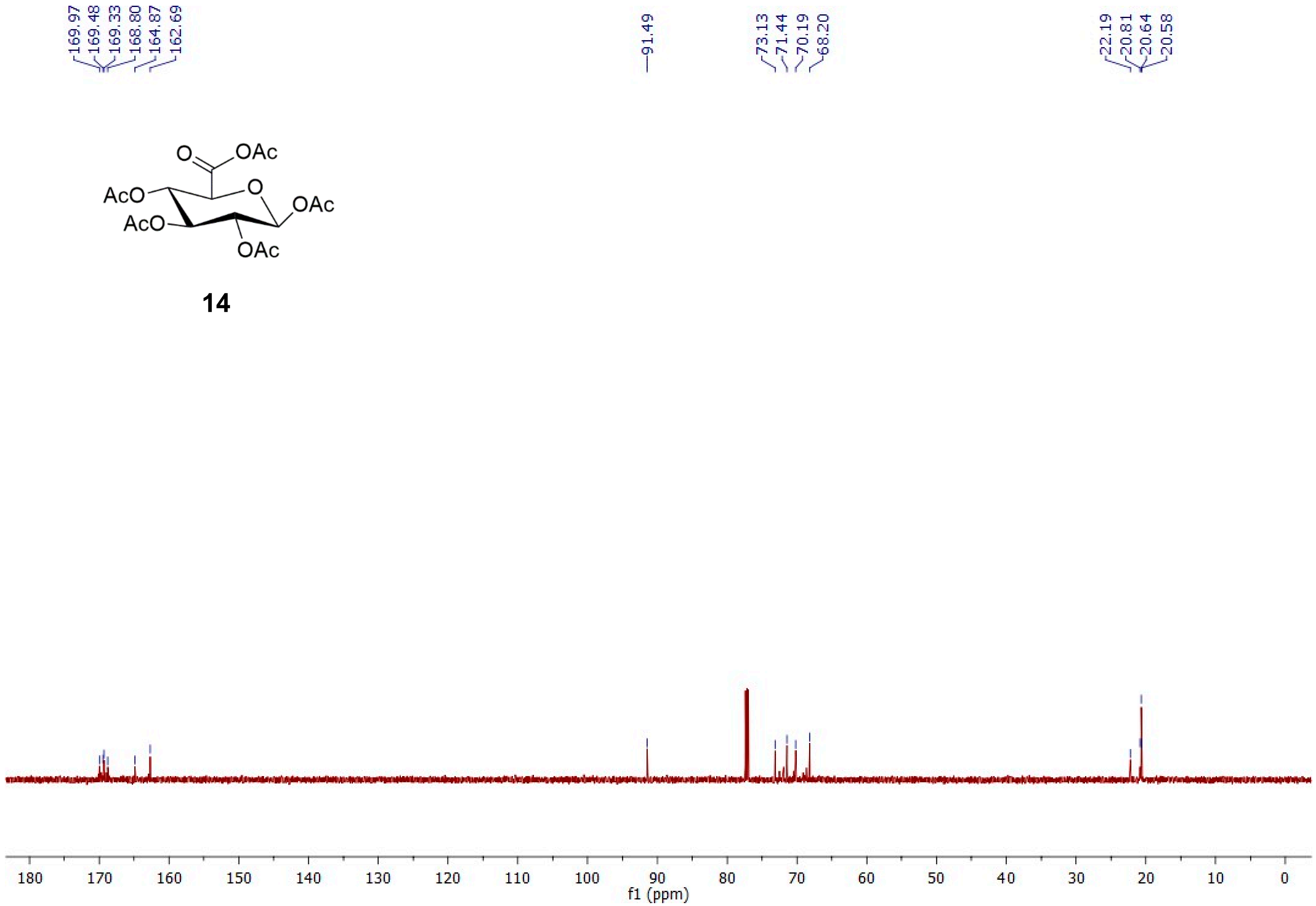

Fig. S22: ^{13}C NMR spectrum of 1,2,3,4-tetra-*O*-acetyl-β-D-glucopyranuronic acetic anhydride **14** (100 MHz/$CDCl_3$/TMS; δ (ppm)).

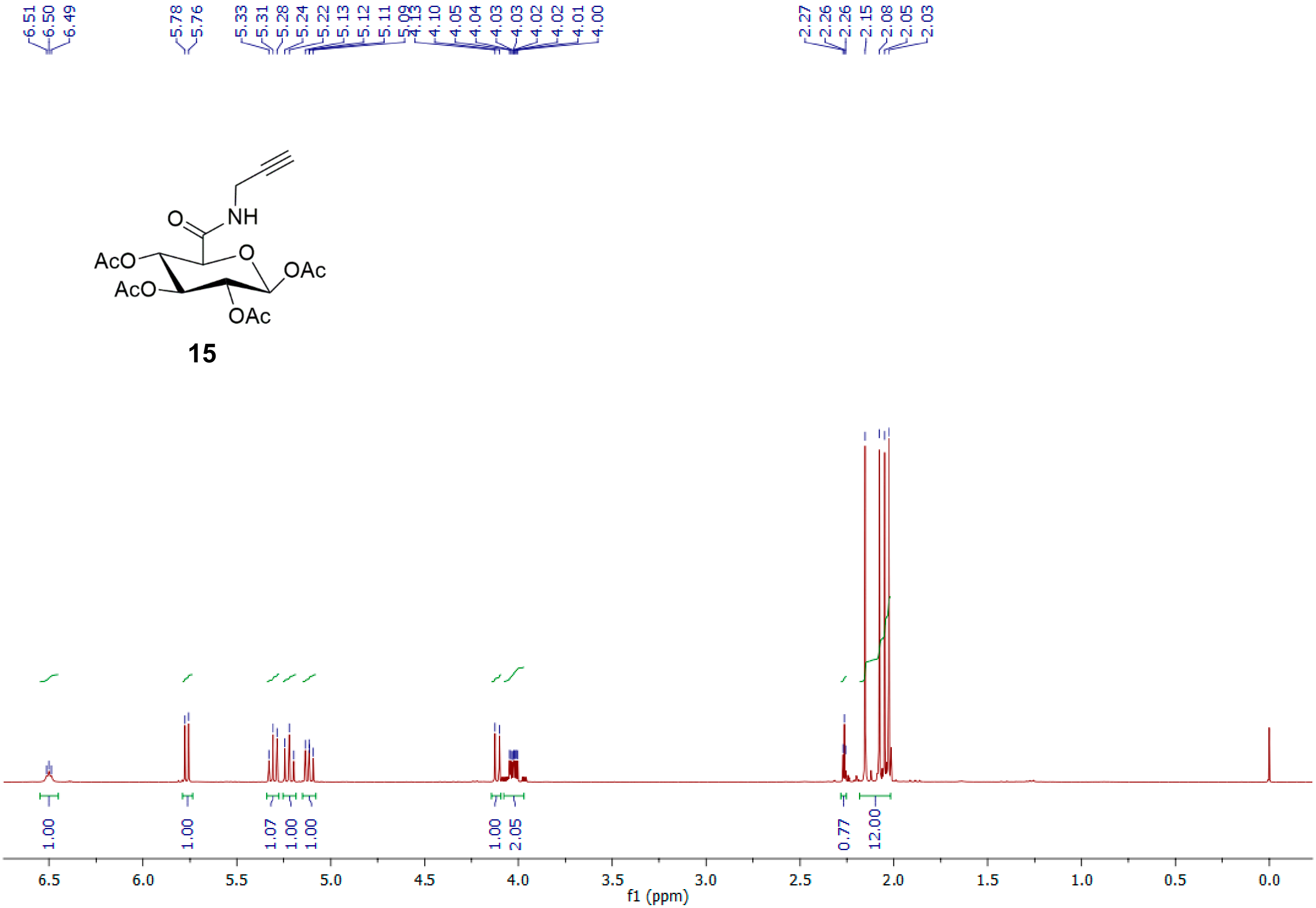

Fig. S23: ^{1}H NMR spectrum of 1,2,3,4-tetra-*O*-acetyl-*N*-(prop-2-yn-1-yl)-β-D-glucopyranuronic acid amide **15** (400 MHz/$CDCl_3$/TMS; δ (ppm)).

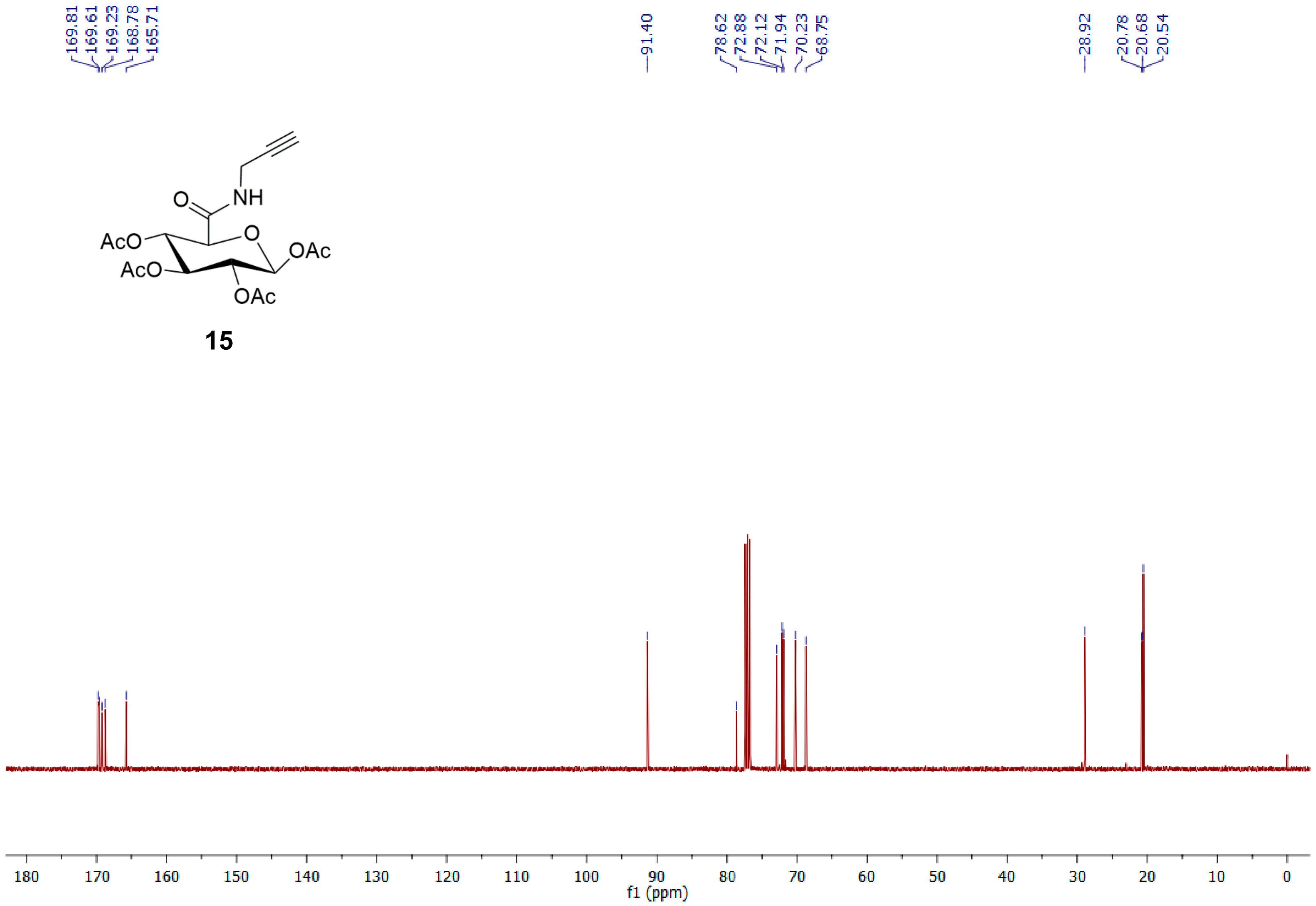

Fig. S24: ^{13}C NMR spectrum of 1,2,3,4-tetra-*O*-acetyl-*N*-(prop-2-yn-1-yl)-β-D-glucopyranuronic acid amide **15** (100 MHz/$CDCl_3$/TMS; δ (ppm)).

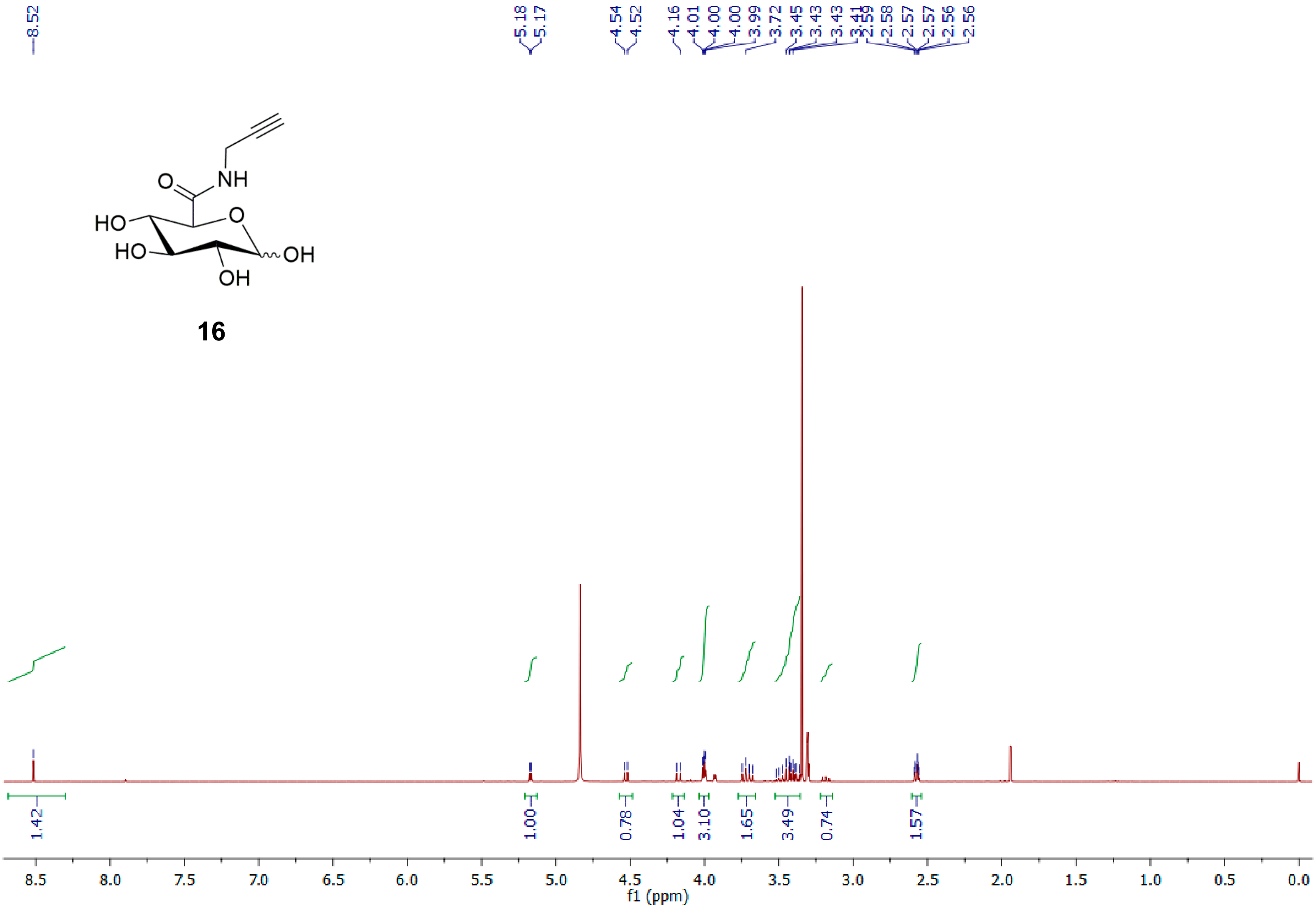

Fig. S25: ^{1}H NMR spectrum of *N*-(prop-2-yn-1-yl)-D-glucopyranuronic acid amide **16** (400 MHz/CD_3OD/TMS; δ (ppm)).

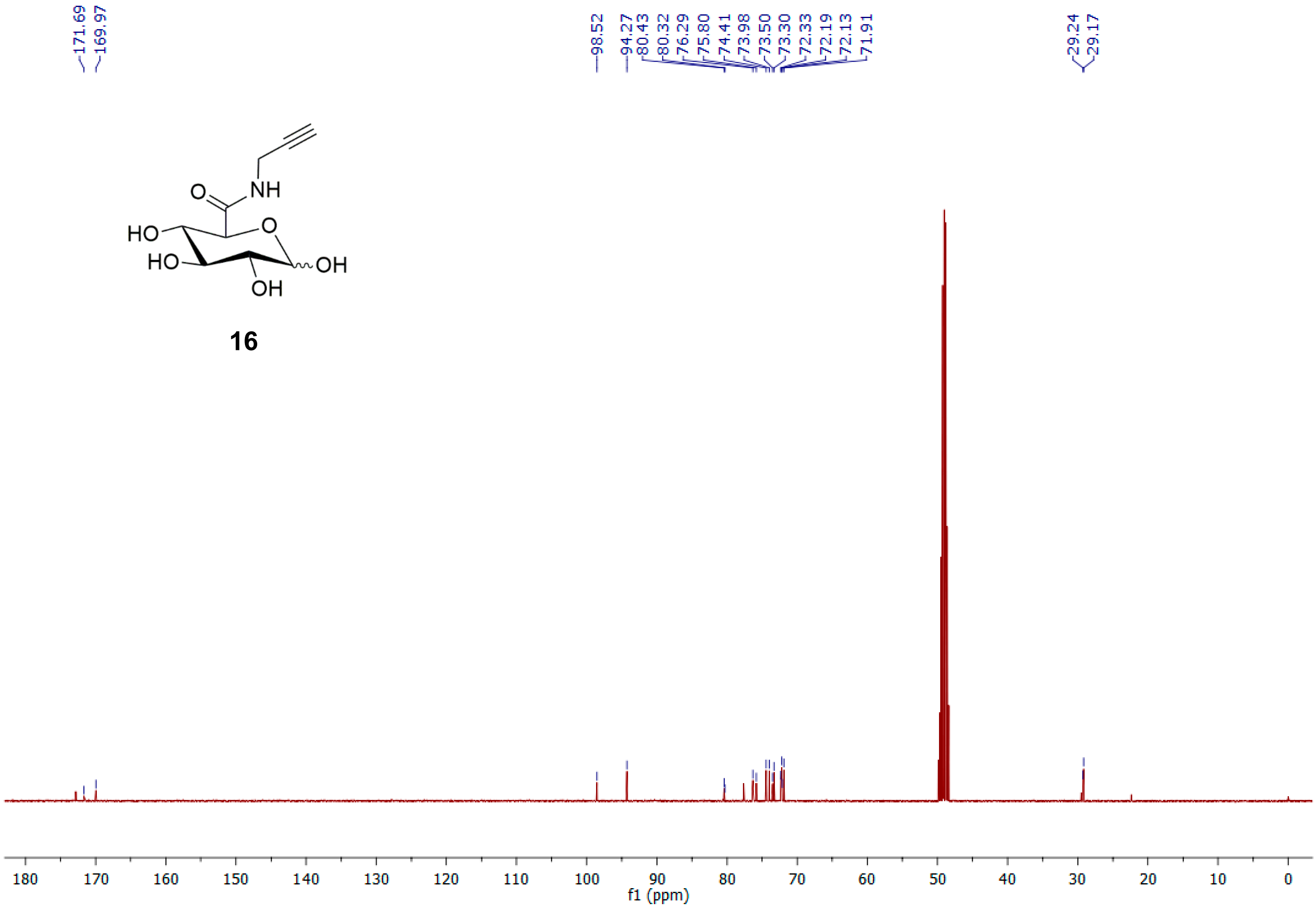

Fig. S26: ^{13}C NMR spectrum of *N*-(prop-2-yn-1-yl)-D-glucopyranuronic acid amide **16** (100 MHz/CD_3OD/TMS; δ (ppm)).

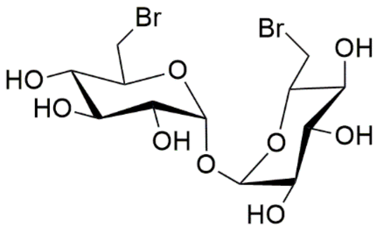

18

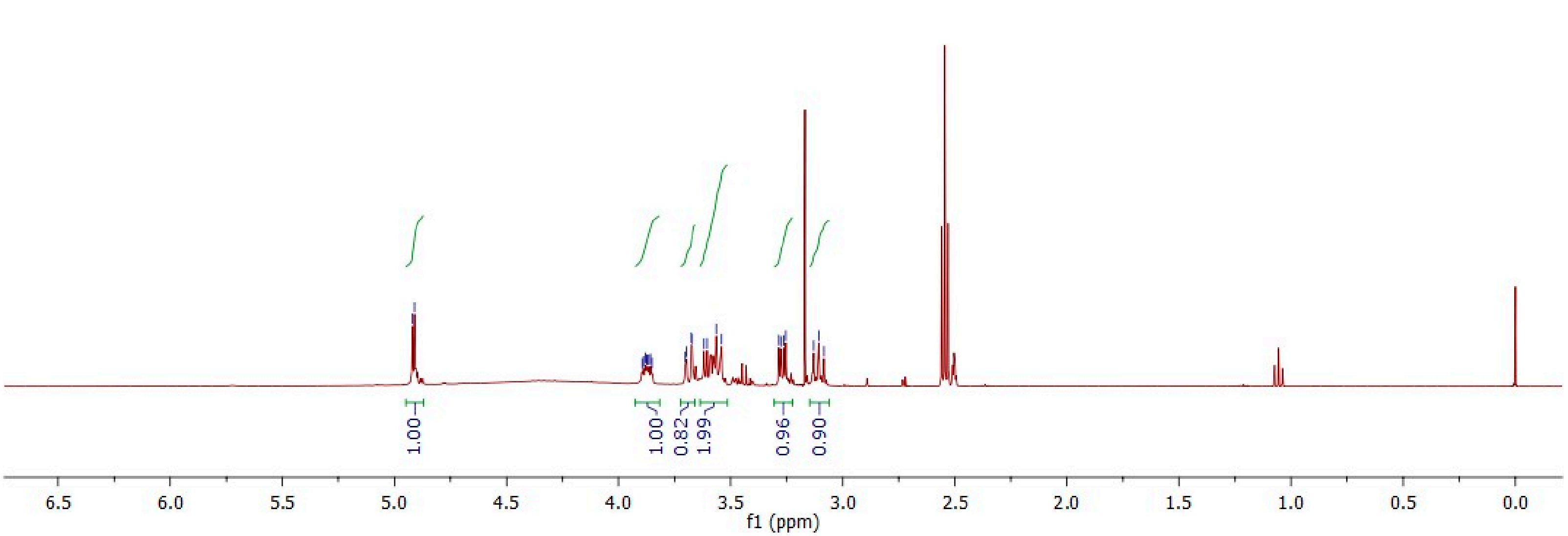

Fig. S27: ^{1}H NMR spectrum of 6,6′-dibromo-6,6′-dideoxy-D-trehalose **18** (400 MHz/DMSO/TMS; δ (ppm)).

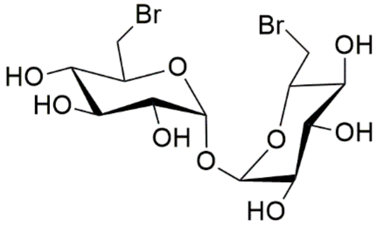

18

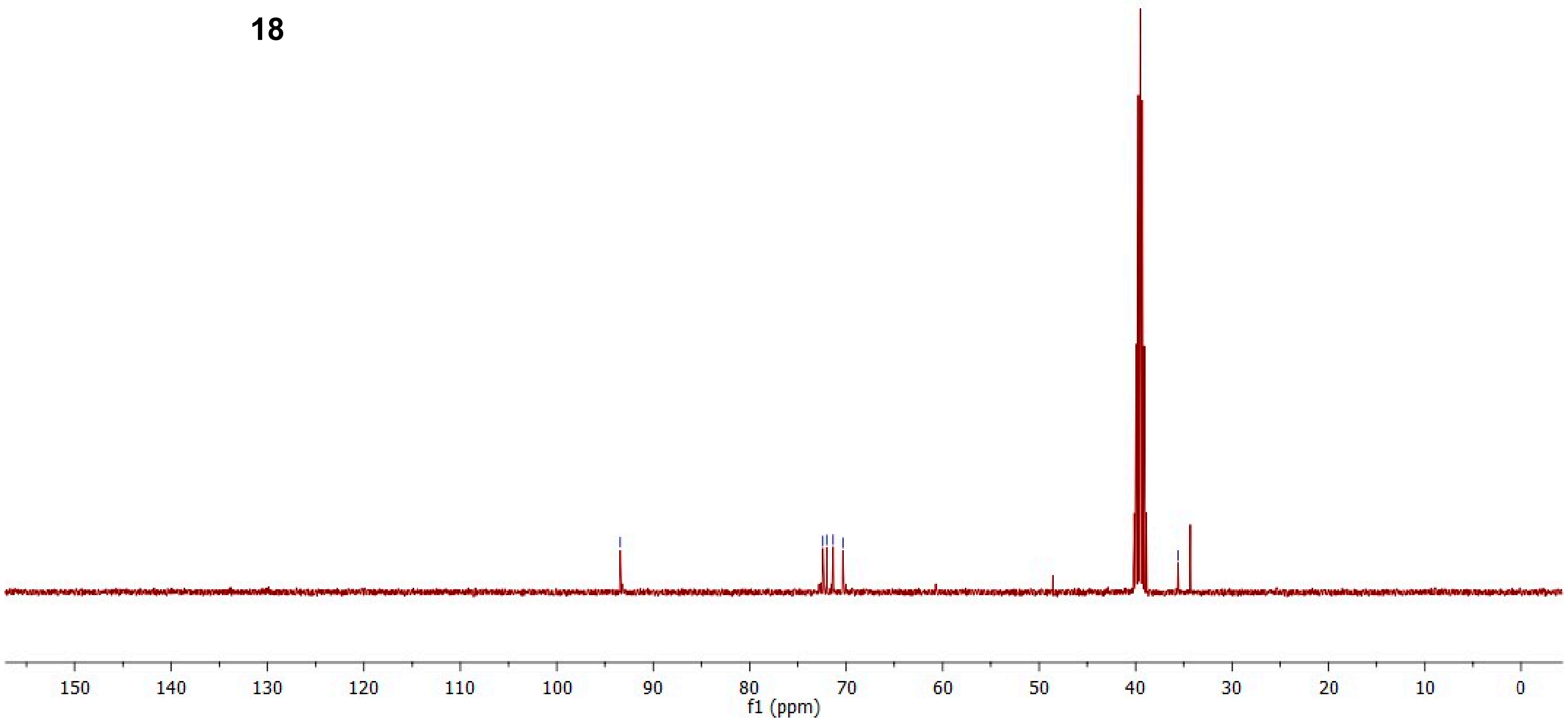

Fig. S28: ^{13}C NMR spectrum of 6,6′-dibromo-6,6′-dideoxy-D-trehalose **18** (100 MHz/DMSO/TMS; δ (ppm)).

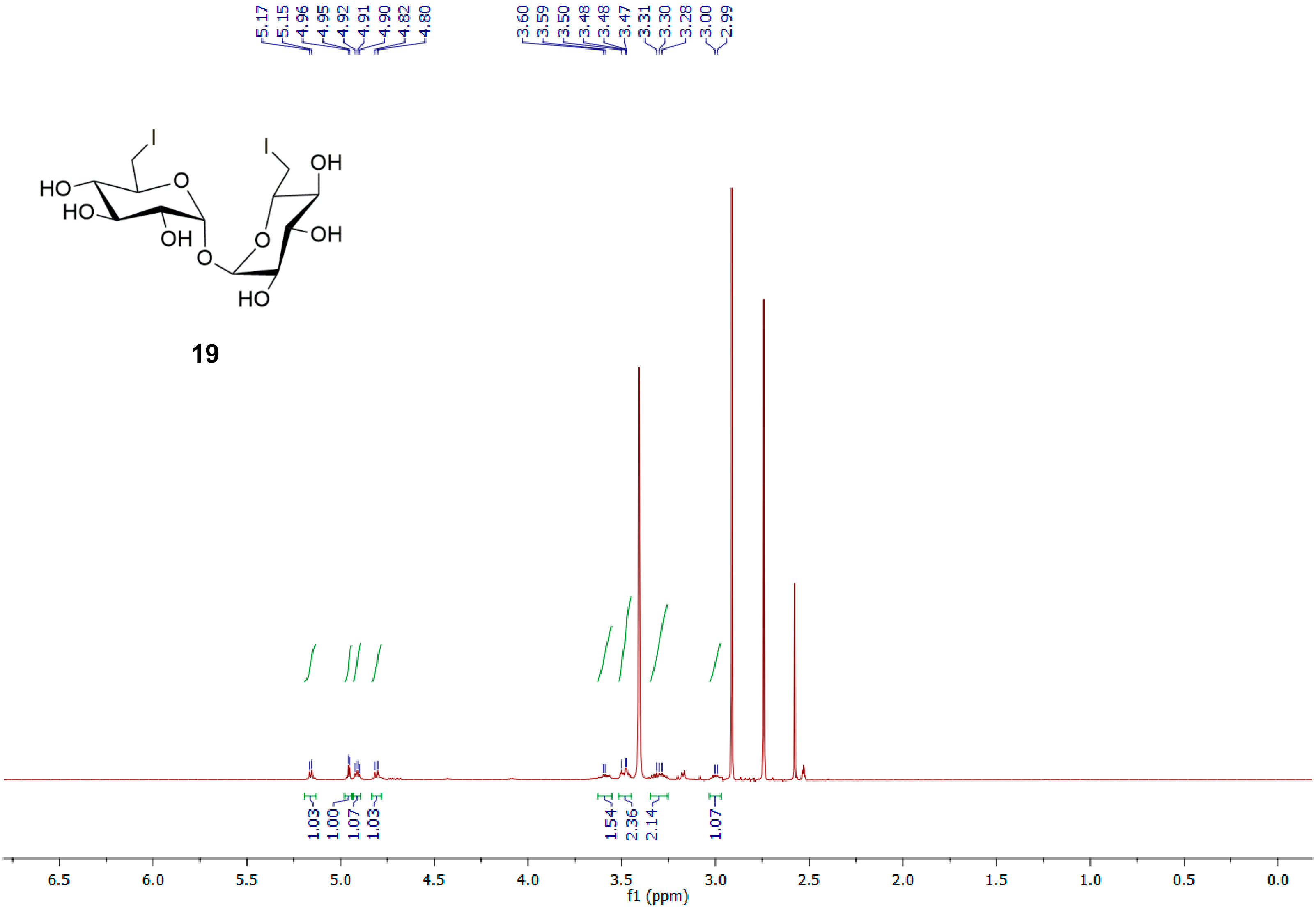

Fig. S29: ^{1}H NMR spectrum of 6,6′-diiodo-6,6′-dideoxy-D-trehalose **19** (400 MHz/DMSO/TMS; δ (ppm)).

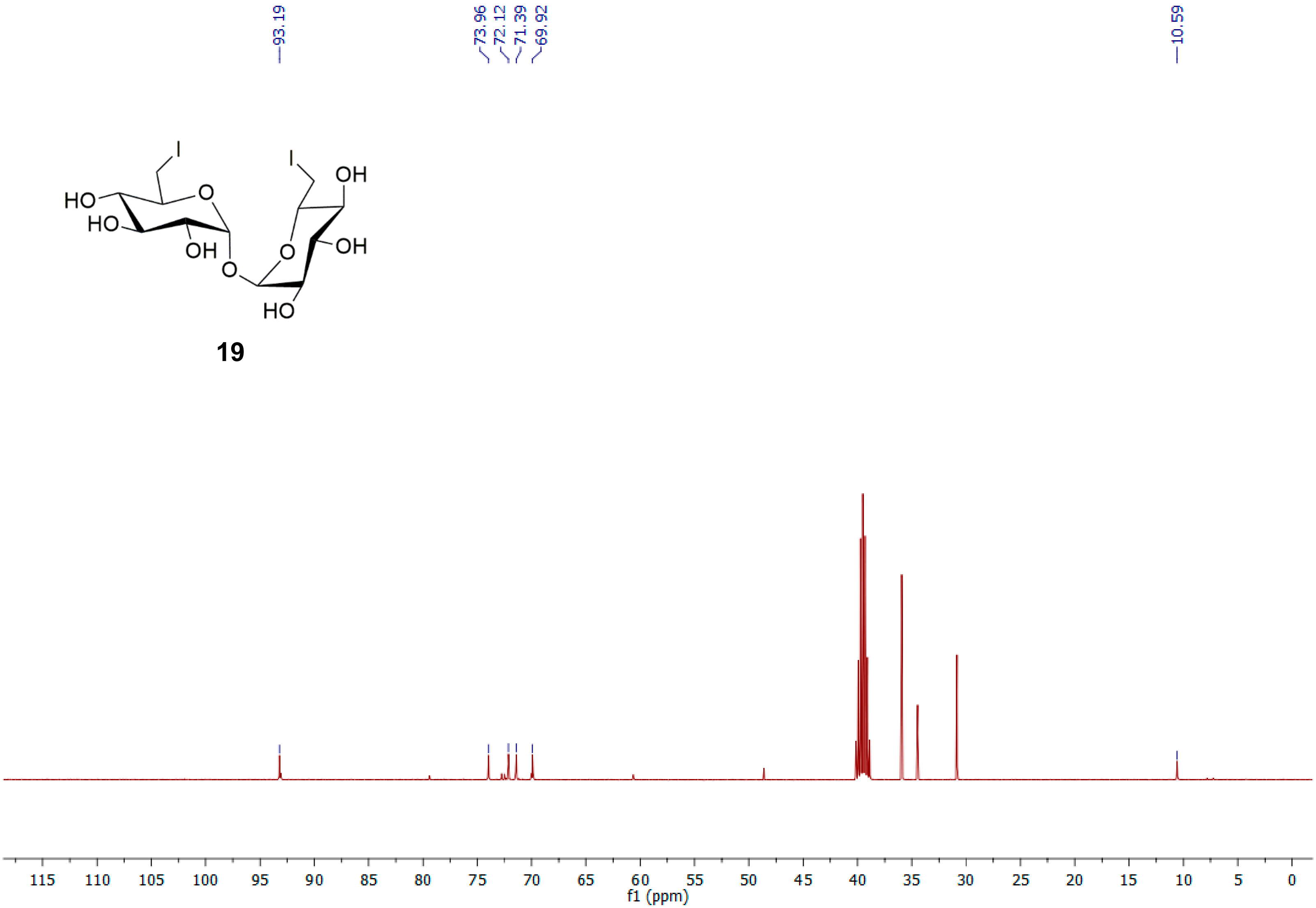

Fig. S30: ^{13}C NMR spectrum of 6,6′-diiodo-6,6′-dideoxy-D-trehalose **19** (100 MHz/DMSO/TMS; δ (ppm)).

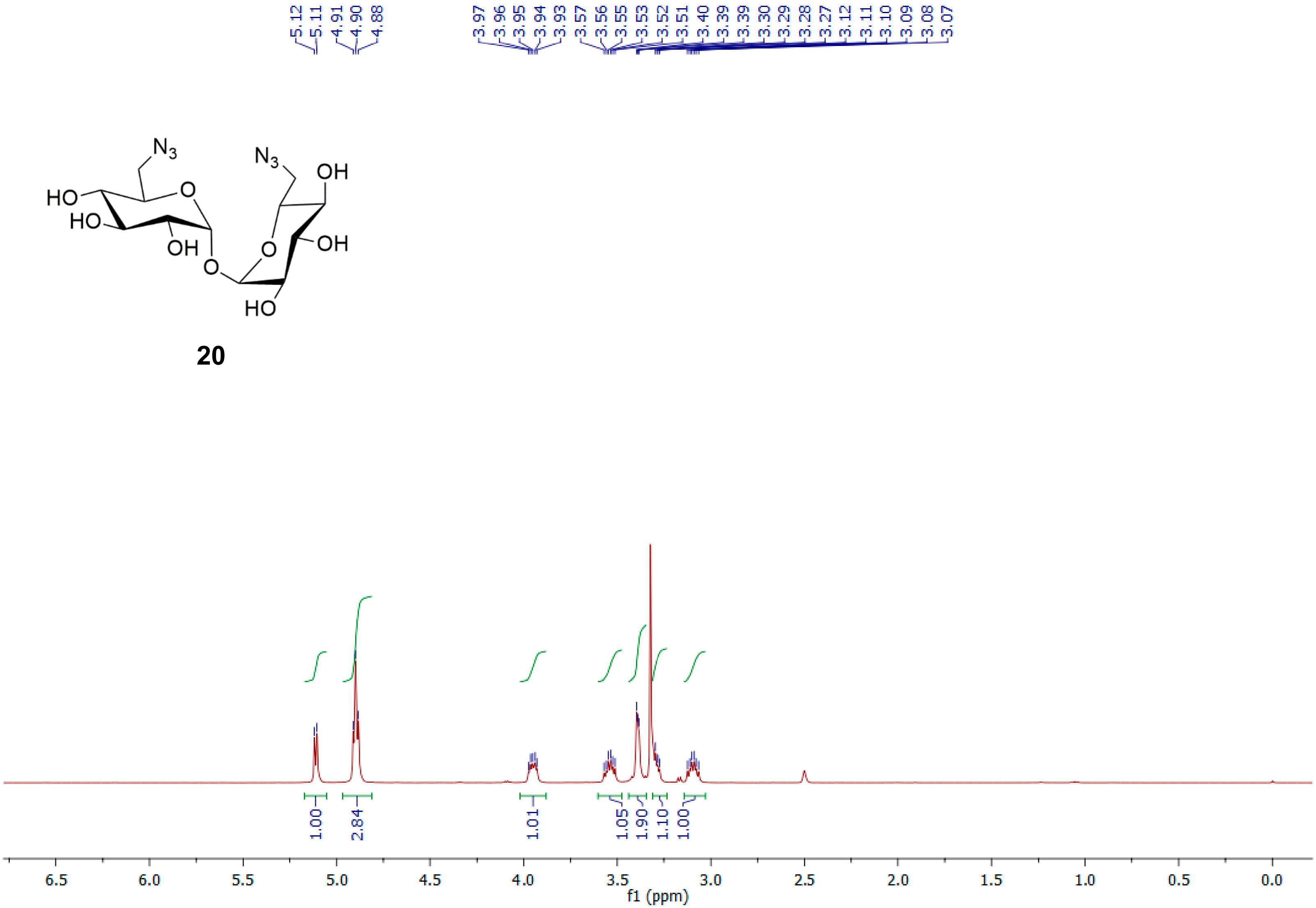

20

Fig. S31: ^{1}H NMR spectrum of 6,6′-diazido-6,6′-dideoxy-D-trehalose **20** (400 MHz/DMSO/TMS; δ (ppm)).

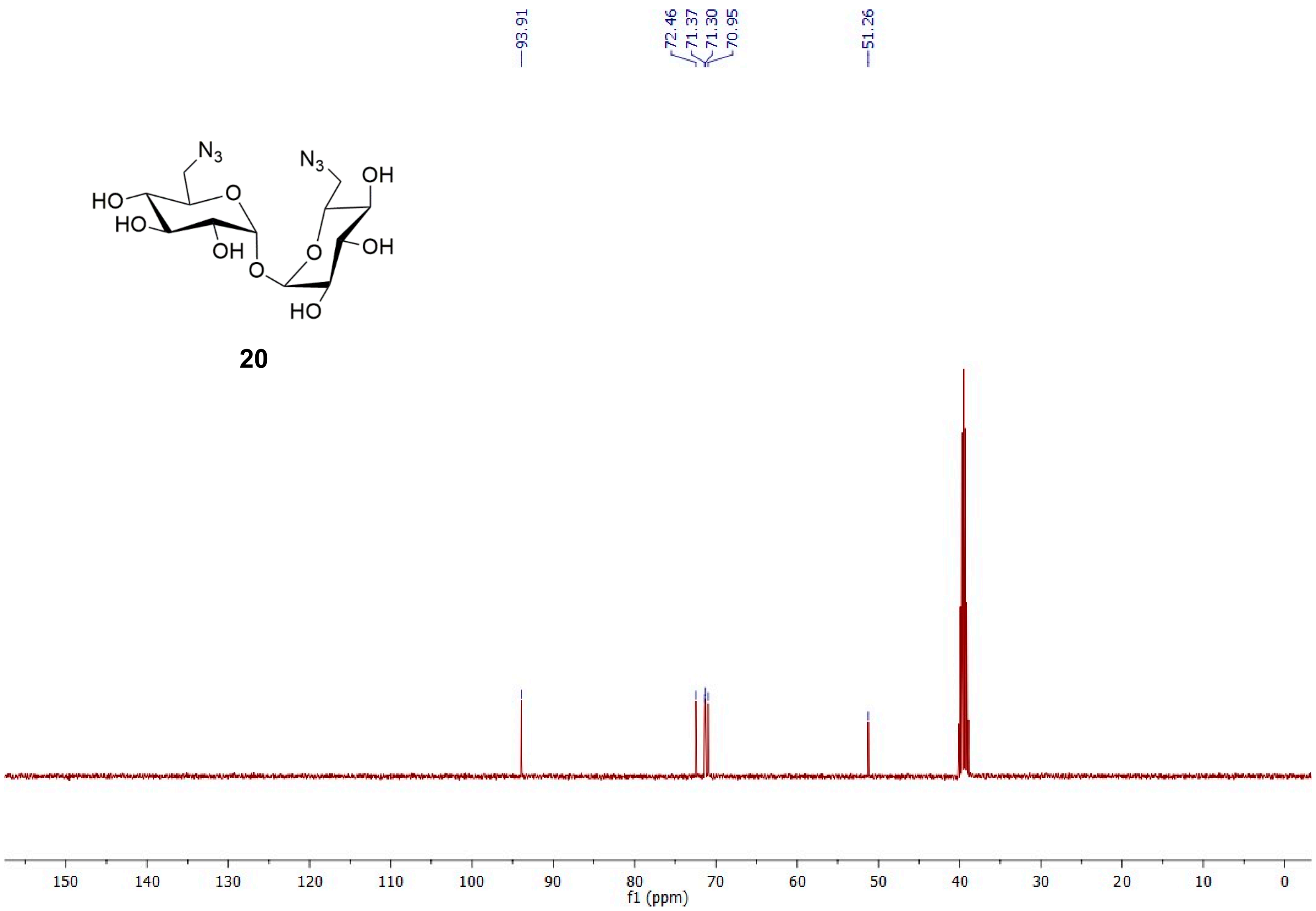

Fig. S32: ^{13}C NMR spectrum of 6,6′-diazido-6,6′-dideoxy-D-trehalose **20** (100 MHz/DMSO/TMS; δ (ppm)).

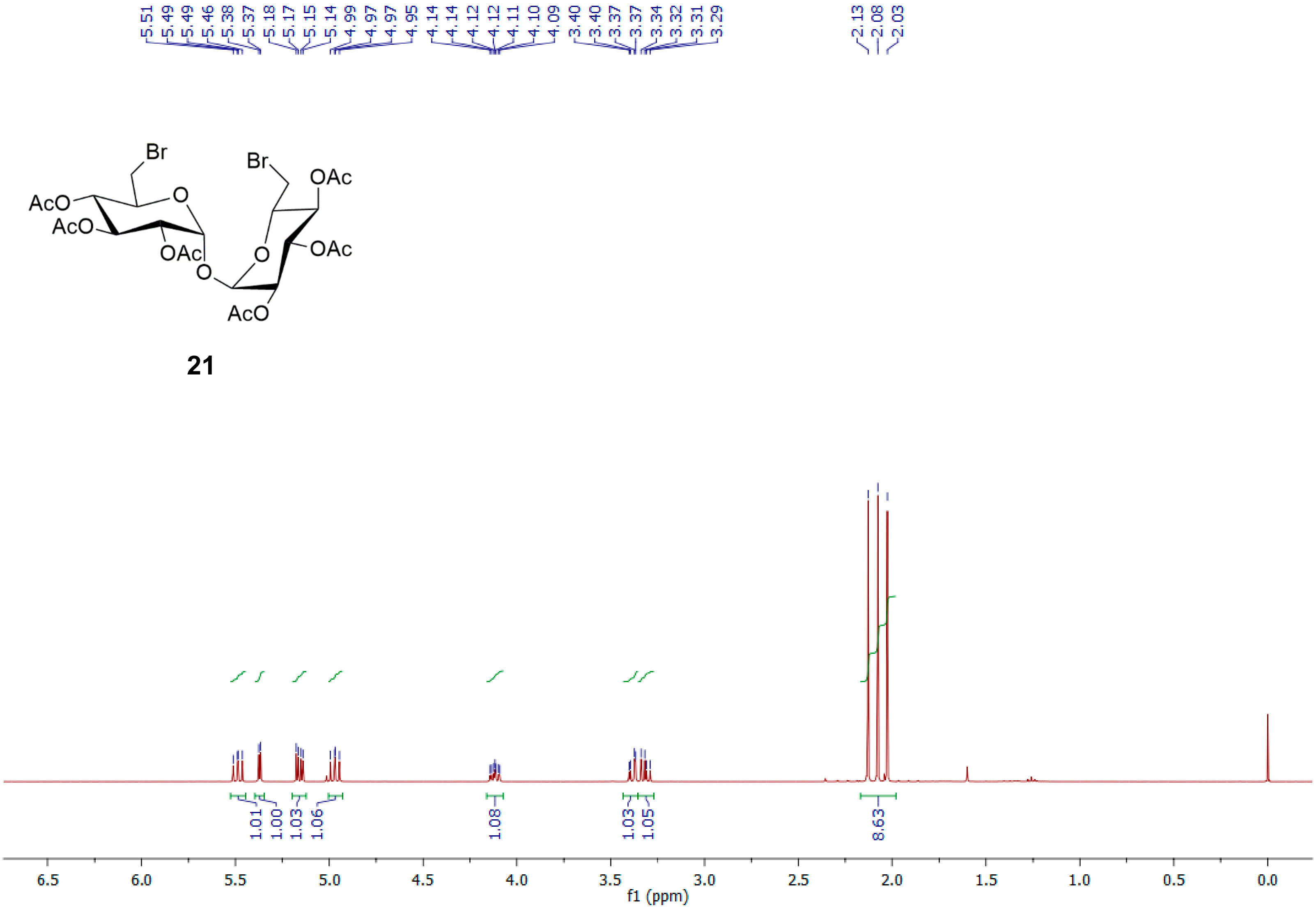

Fig. S33: ^{1}H NMR spectrum of 2,3,4,2′,3′,4′-hexa-*O*-acetyl-6,6′-dibromo-6,6′-dideoxy-D-trehalose **21** (400 MHz/$CDCl_3$/TMS; δ (ppm)).

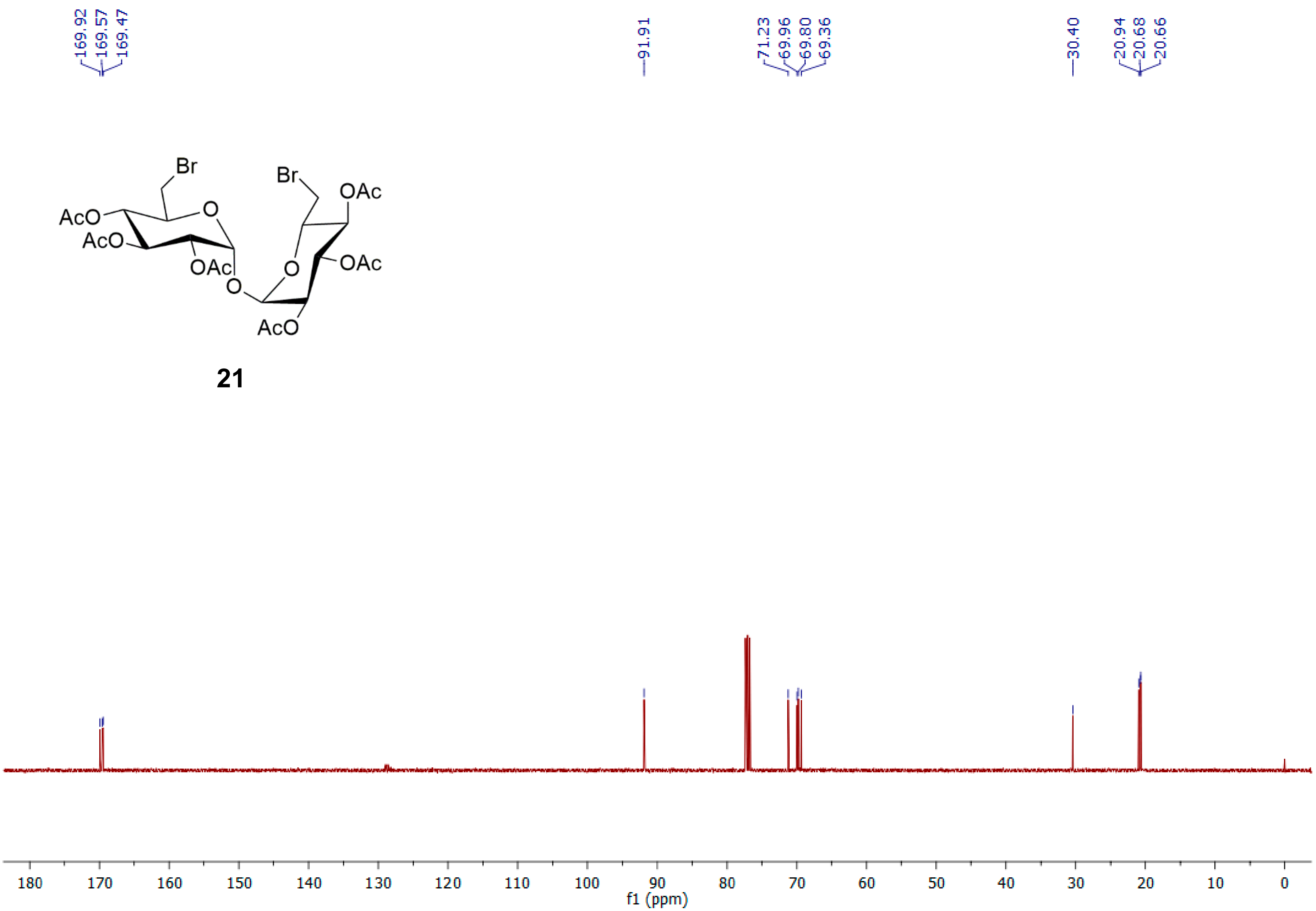

Fig. S34: ^{13}C NMR spectrum of 2,3,4,2′,3′,4′-hexa-*O*-acetyl-6,6′-dibromo-6,6′-dideoxy-D-trehalose **21** (100 MHz/$CDCl_3$/TMS; δ (ppm)).

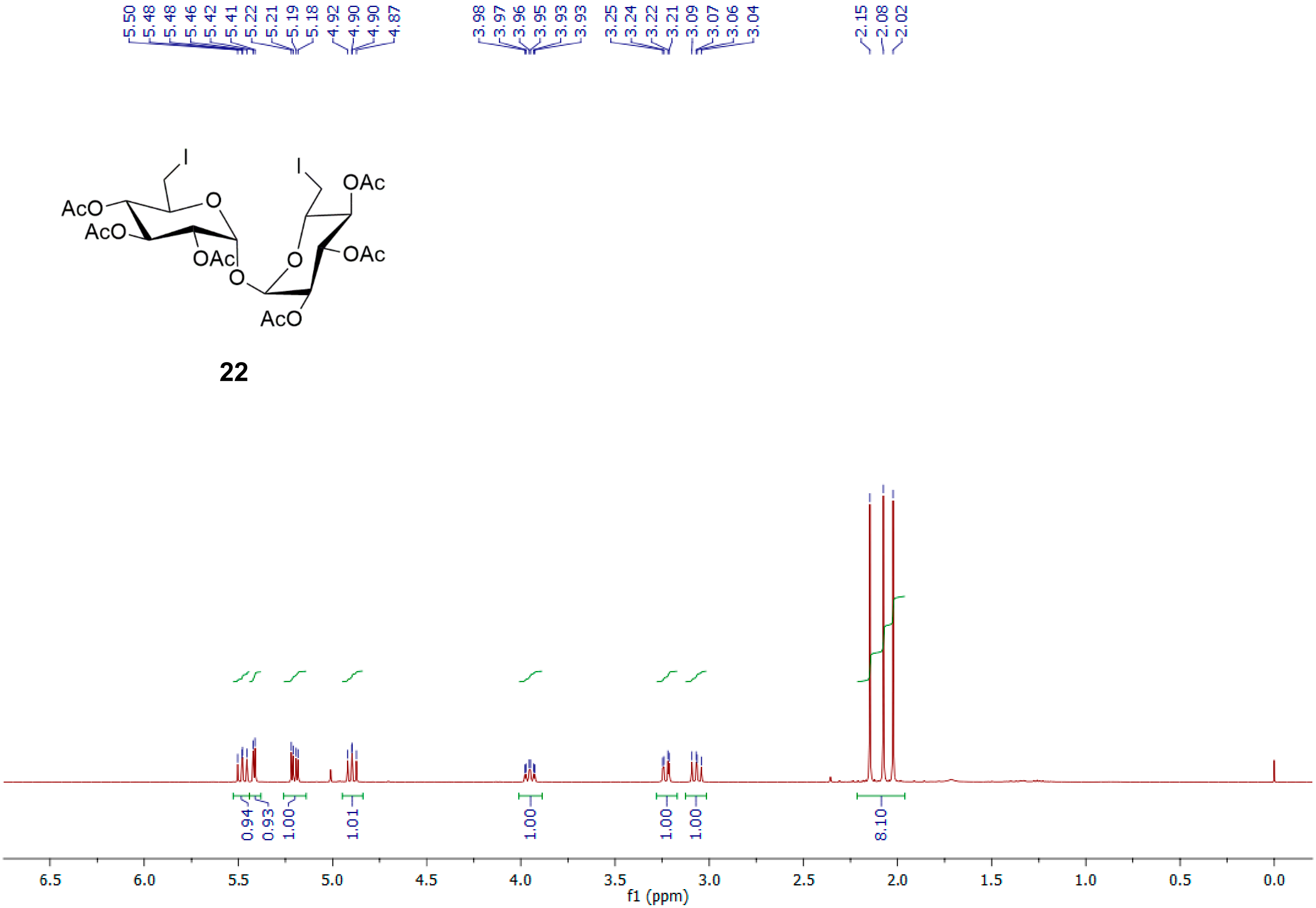

Fig. S35: ^{1}H NMR spectrum of 2,3,4,2′,3′,4′-hexa-*O*-acetyl-6,6′-diiodo-6,6′-dideoxy-D-trehalose **22** (400 MHz/$CDCl_3$/TMS; δ (ppm)).

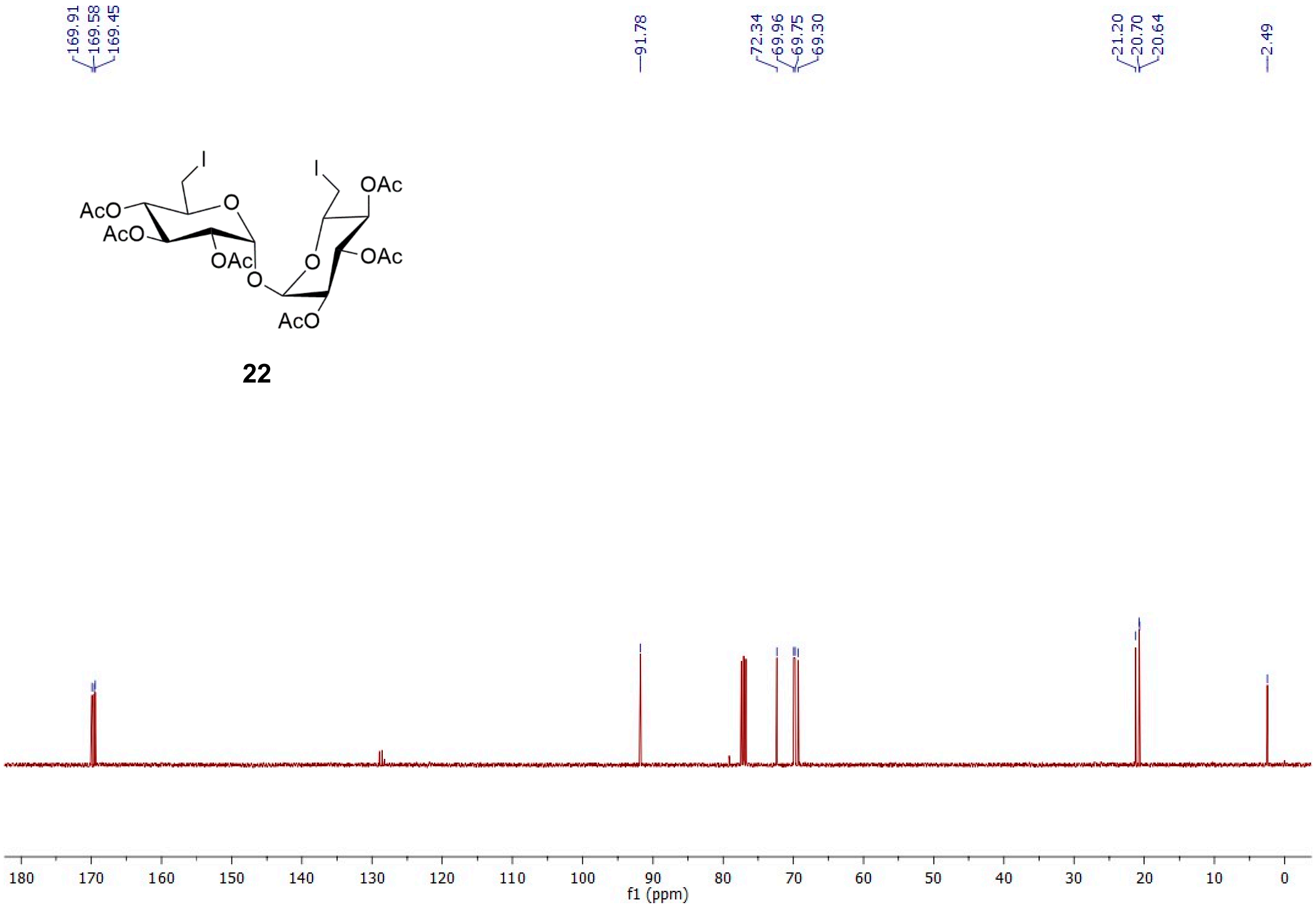

Fig. S36: ^{13}C NMR spectrum of 2,3,4,2′,3′,4′-hexa-*O*-acetyl-6,6′-diiodo-6,6′-dideoxy-D-trehalose **22** (100 MHz/$CDCl_3$/TMS; δ (ppm)).

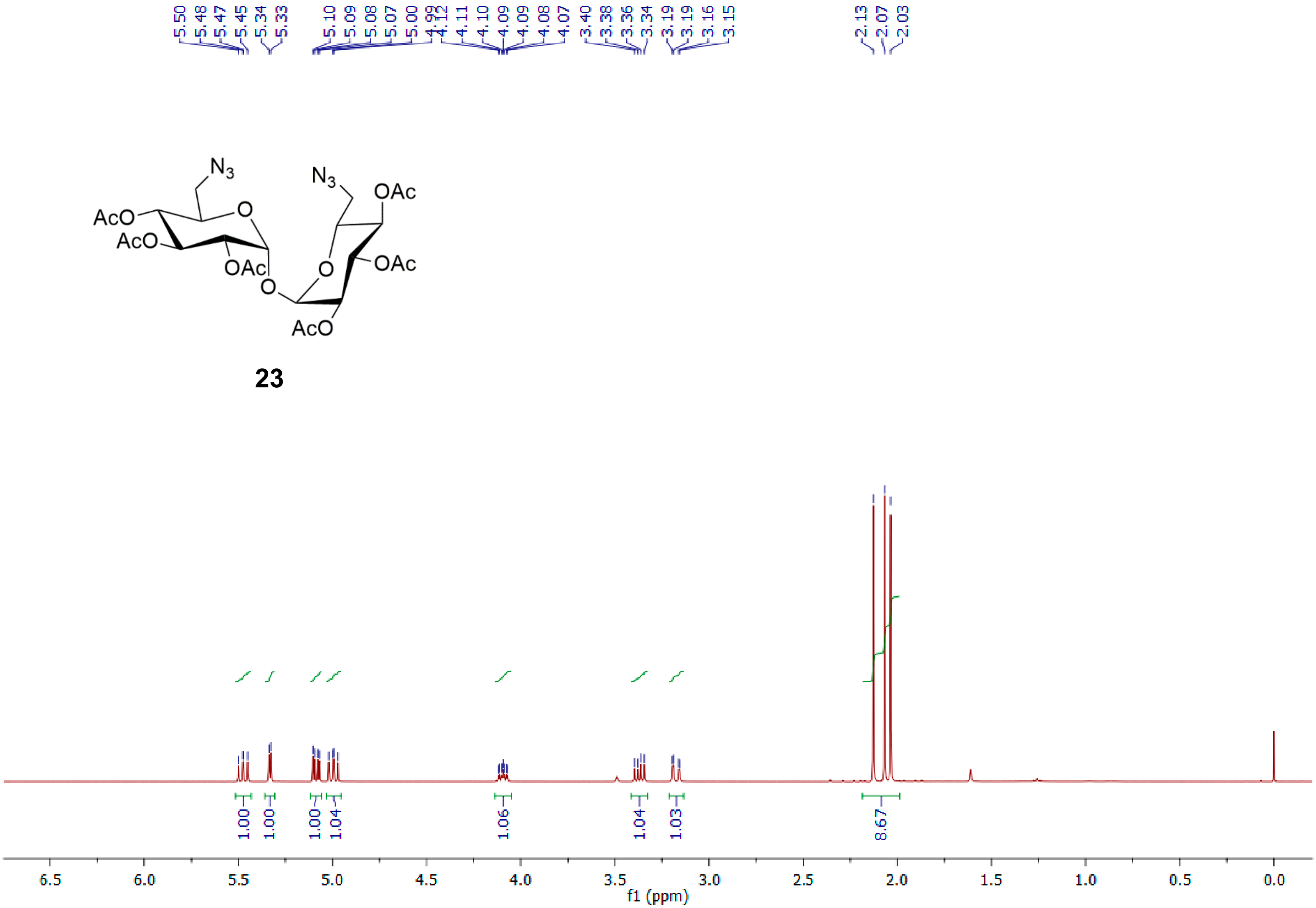

Fig. S37: ¹H NMR spectrum of 2,3,4,2′,3′,4′-hexa-*O*-acetyl-6,6′-diazido-6,6′-dideoxy-D-trehalose **23** (400 MHz/$CDCl_3$/TMS; δ (ppm)).

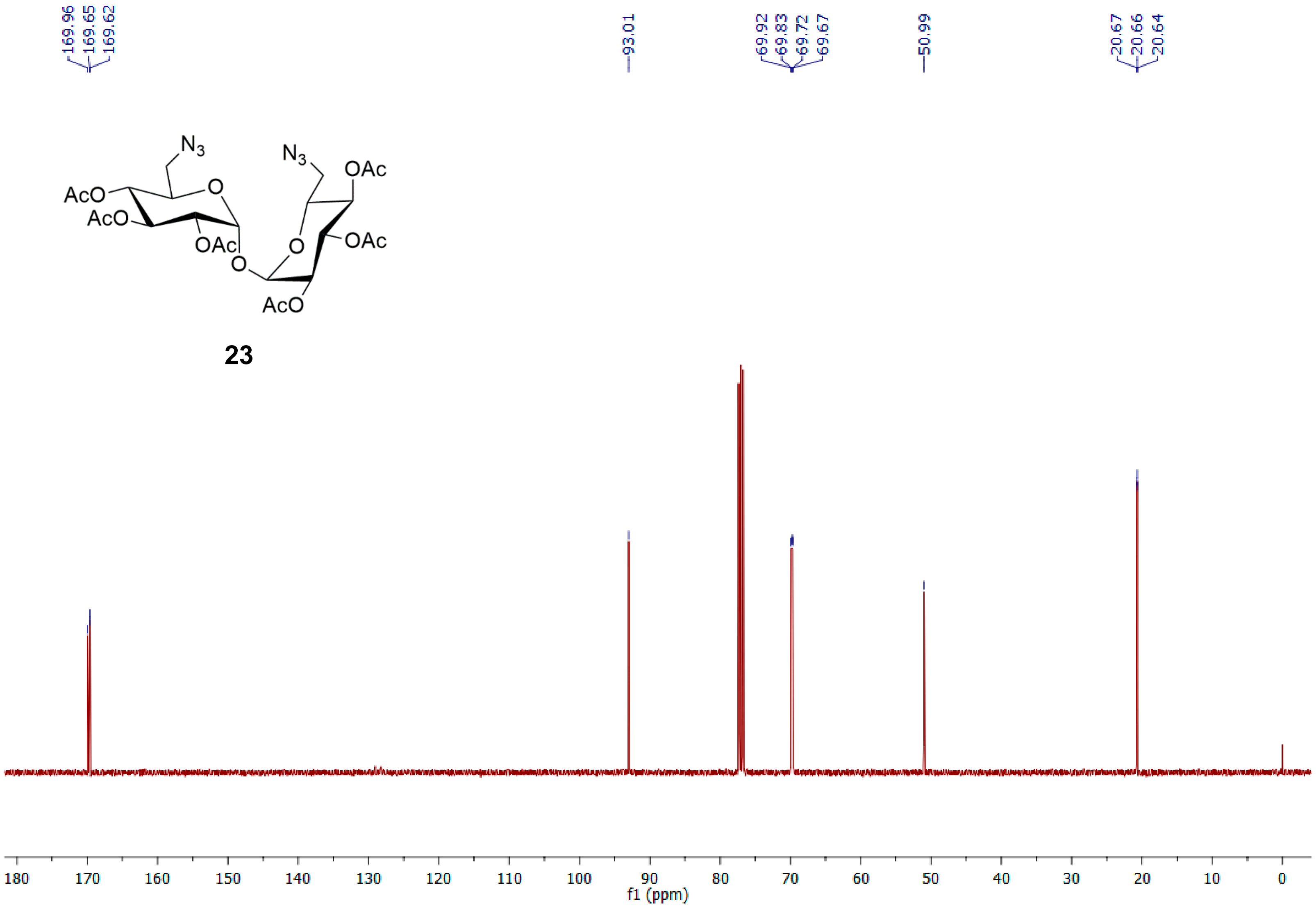

Fig. S38: ^{13}C NMR spectrum of 2,3,4,2′,3′,4′-hexa-*O*-acetyl-6,6′-diazido-6,6′-dideoxy-D-trehalose **23** (100 MHz/$CDCl_3$/TMS; δ (ppm)).

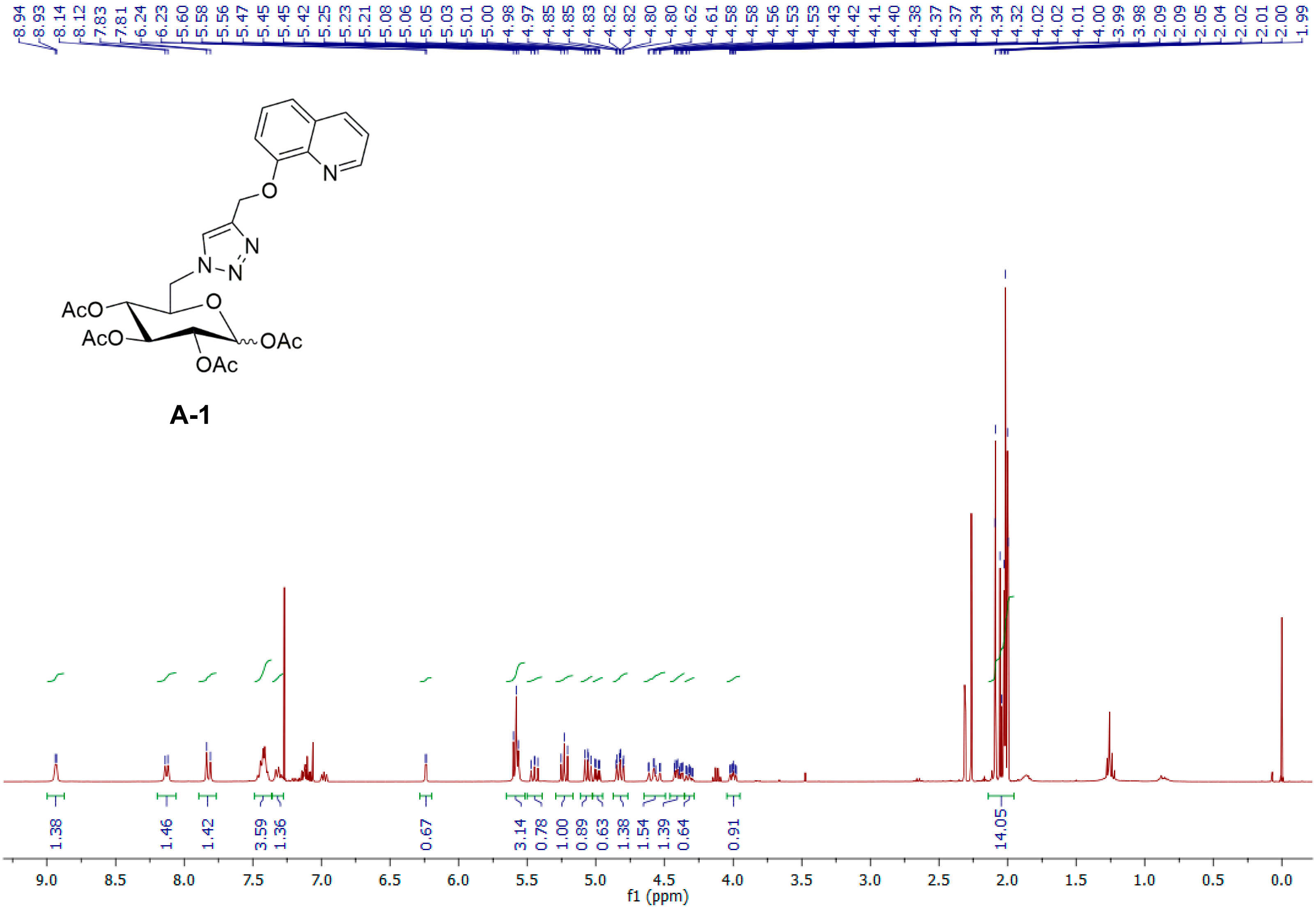

Fig. S39: ^{1}H NMR spectrum of glycoconjugates **A-1** (400 MHz/$CDCl_3$/TMS; δ (ppm)).

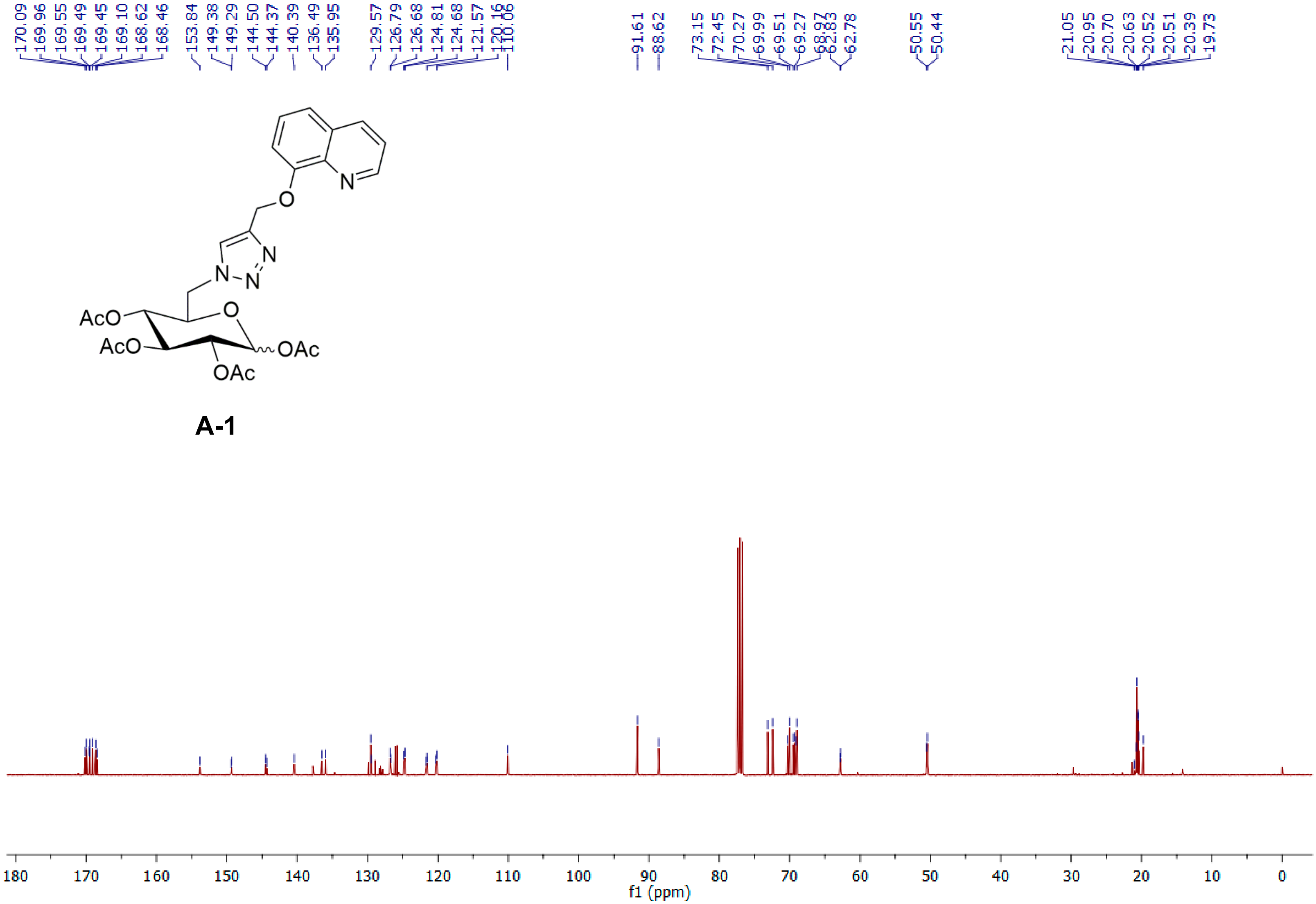

Fig. S40: ^{13}C NMR spectrum of glycoconjugates **A-1** (100 MHz/$CDCl_3$/TMS; δ (ppm)).

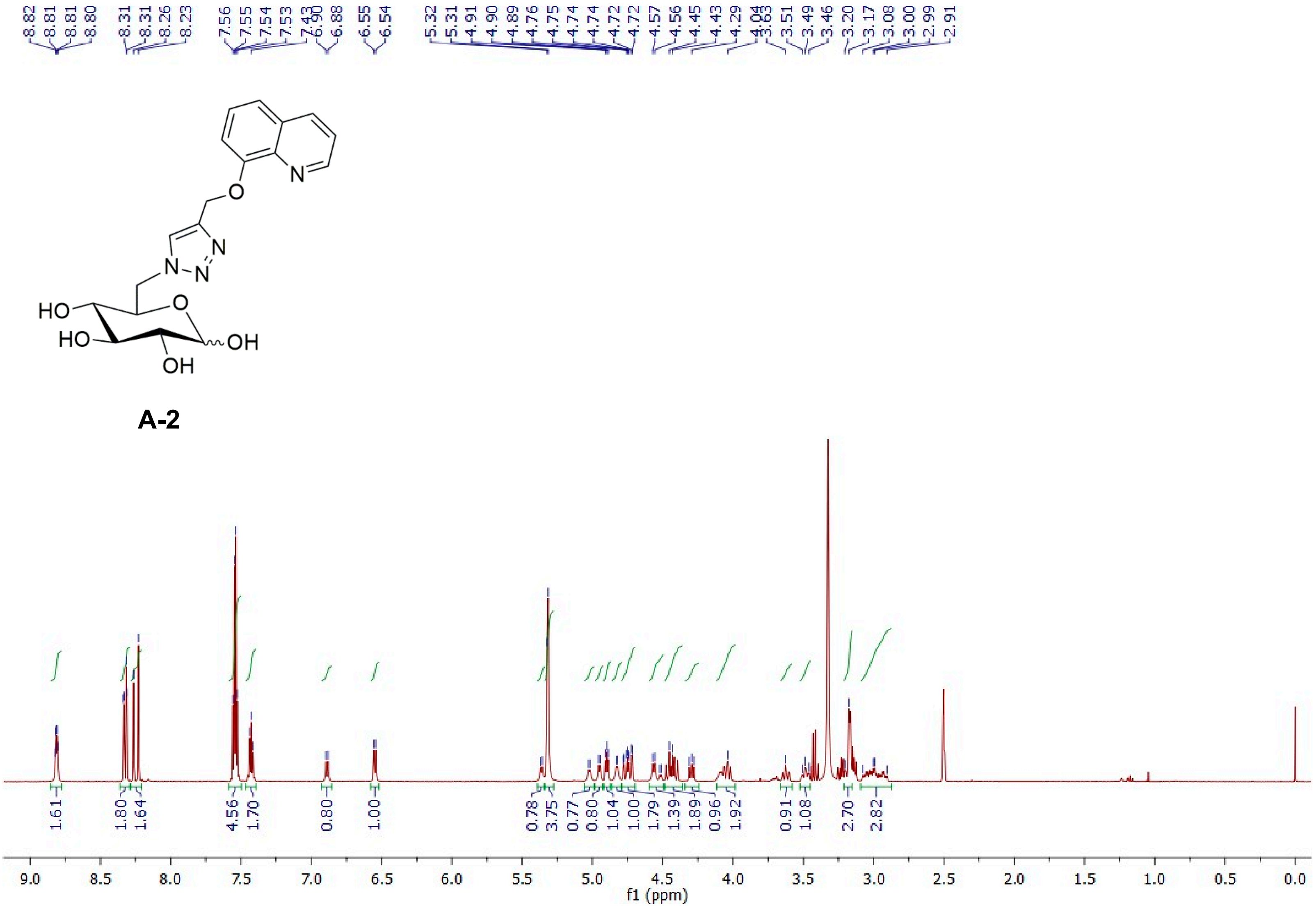

Fig. S41: ^{1}H NMR spectrum of glycoconjugates **A-2** (400 MHz/DMSO/TMS; δ (ppm)).

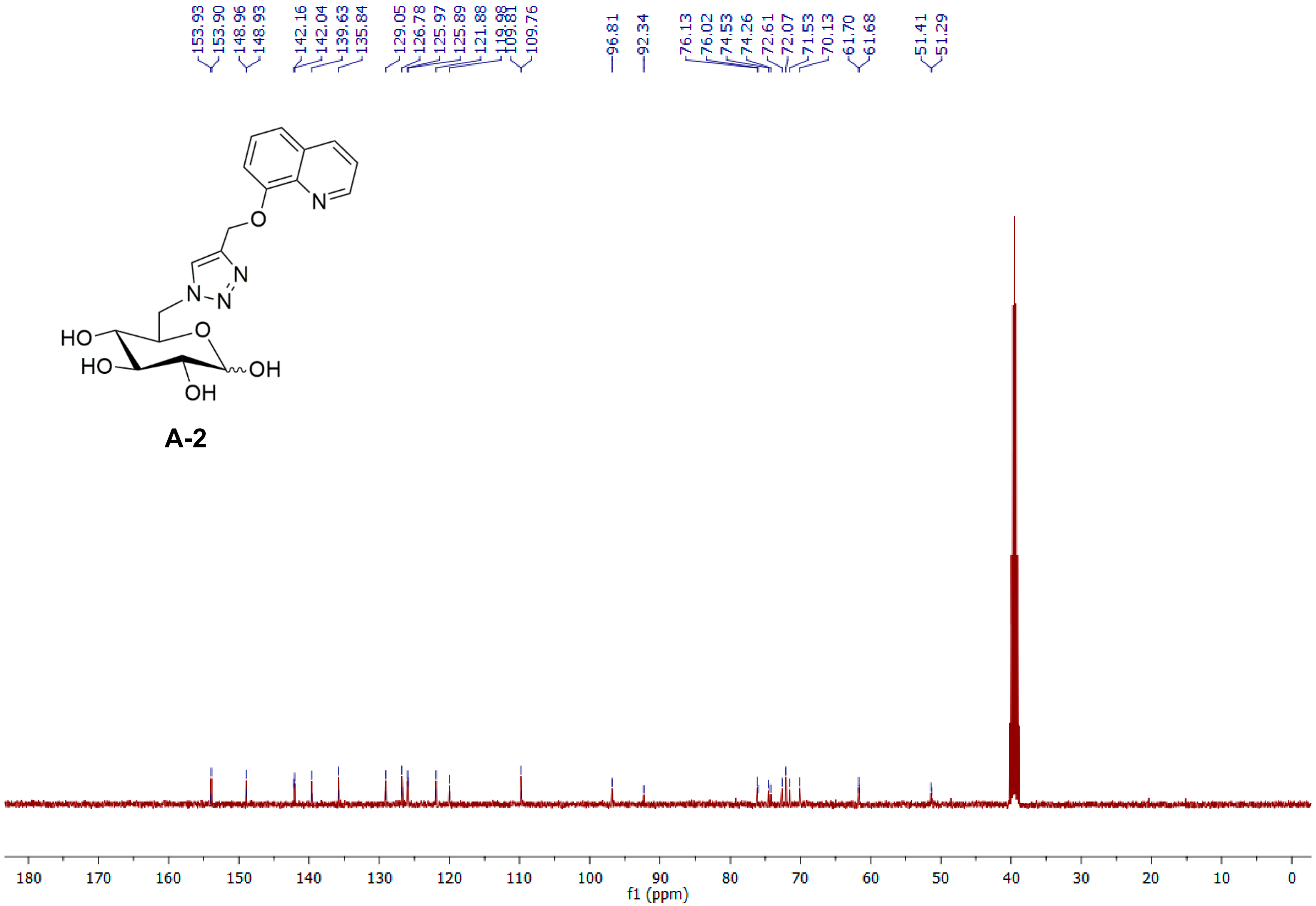

Fig. S42: ^{13}C NMR spectrum of glycoconjugates **A-2** (100 MHz/DMSO/TMS; δ (ppm)).

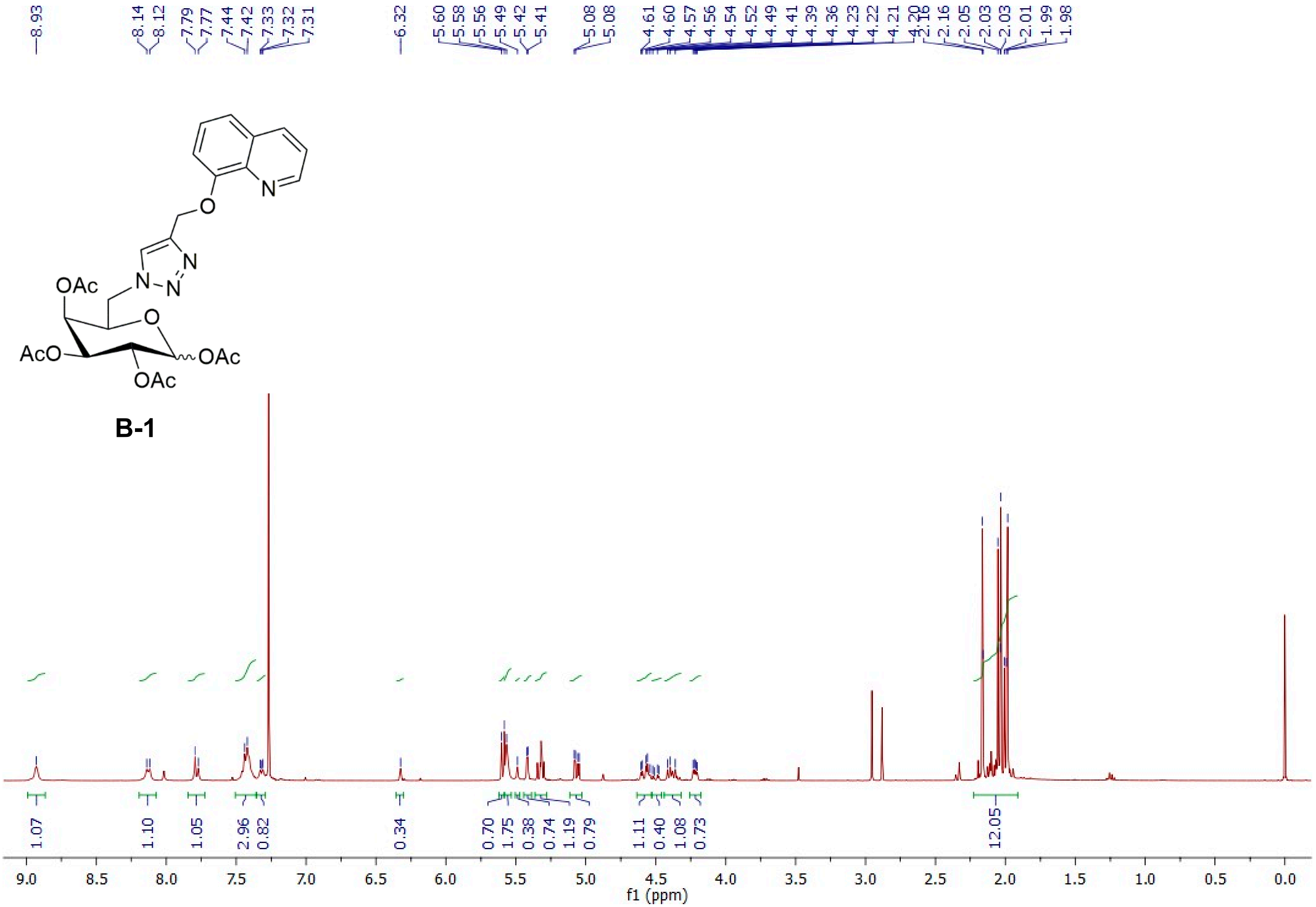

Fig. S43: ^{1}H NMR spectrum of glycoconjugates **B-1** (400 MHz/$CDCl_3$/TMS; δ (ppm)).

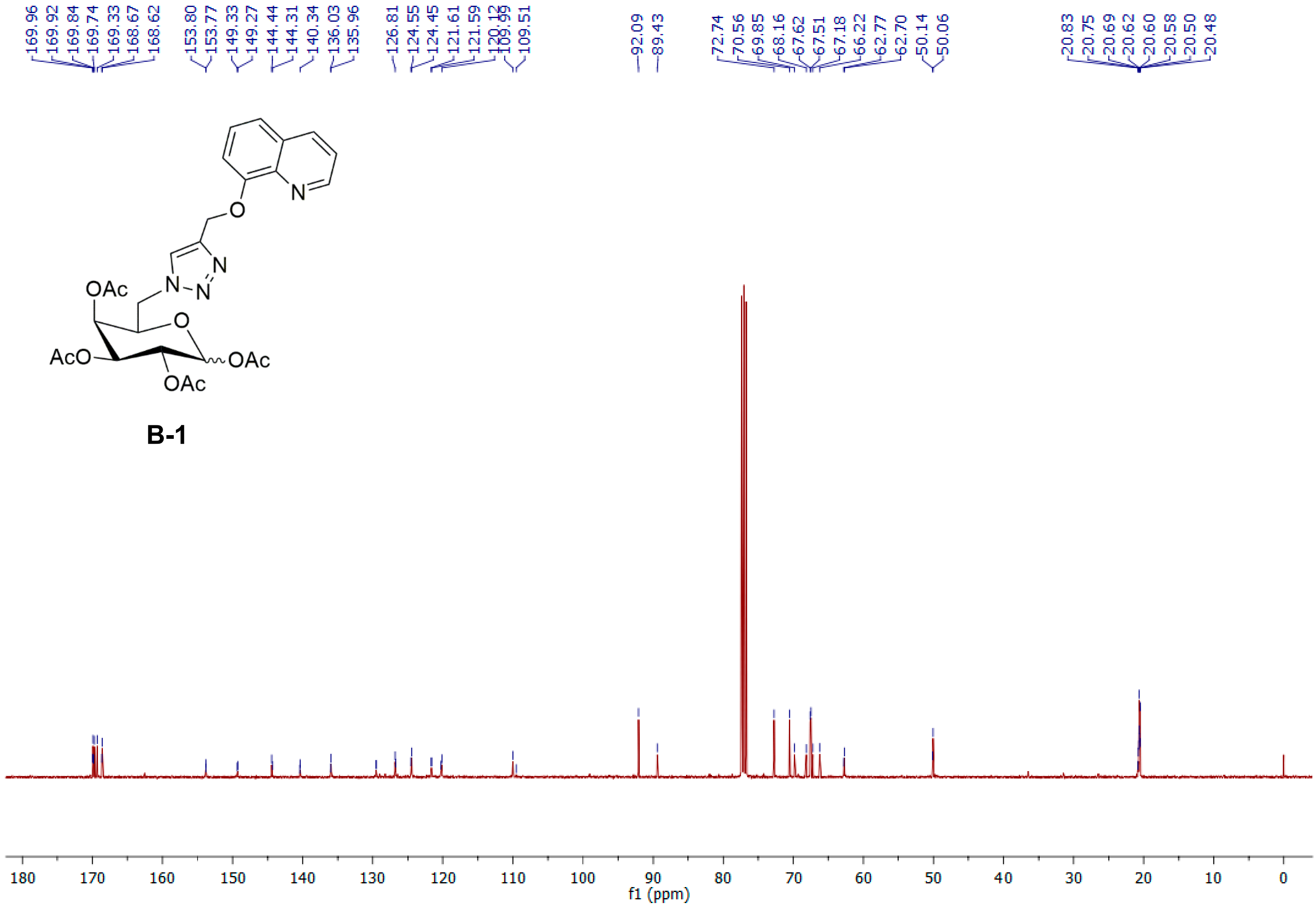

Fig. S44: ^{13}C NMR spectrum of glycoconjugates **B-1** (100 MHz/$CDCl_3$/TMS; δ (ppm)).

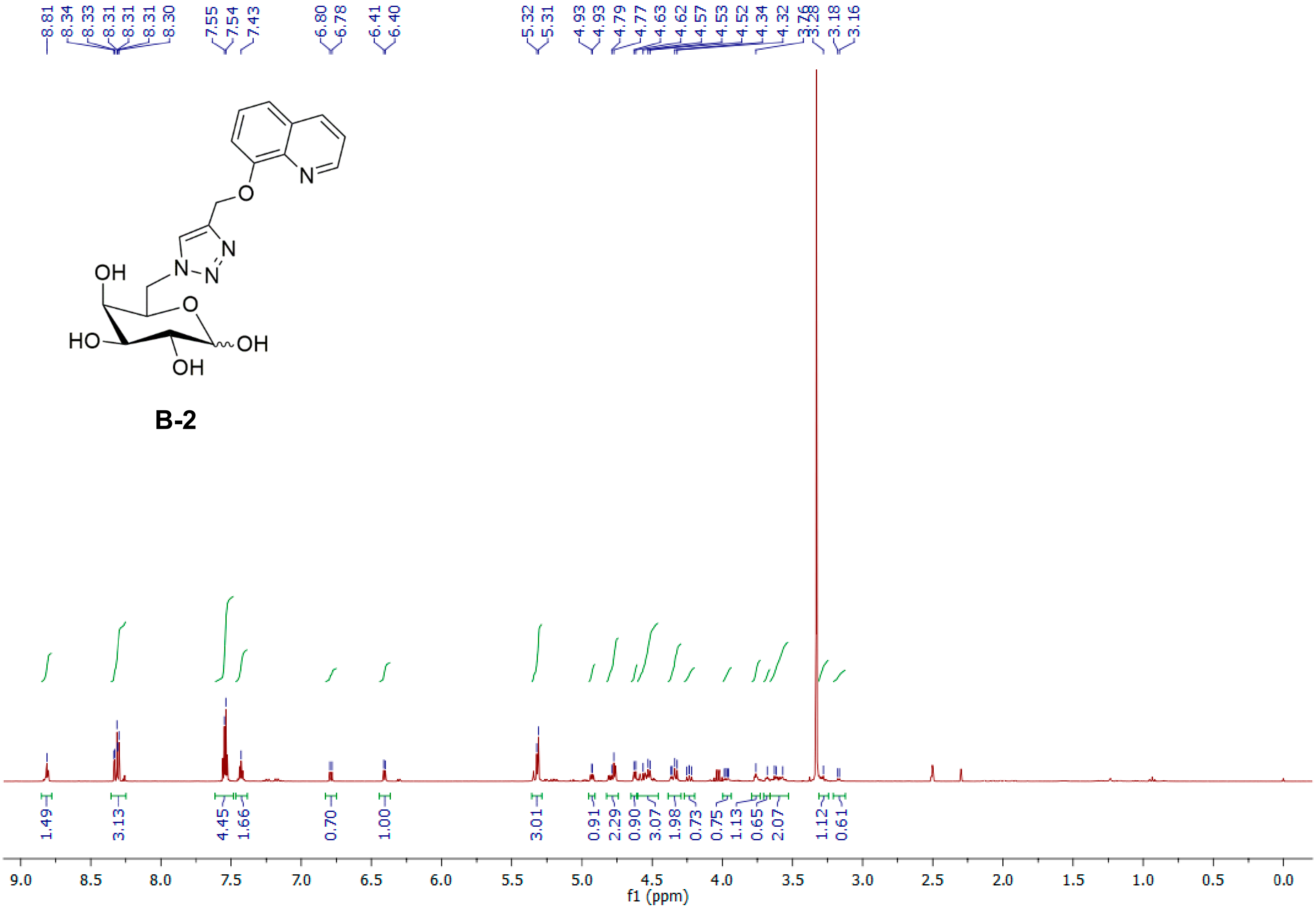

Fig. S45: ^{1}H NMR spectrum of glycoconjugates **B-2** (400 MHz/DMSO/TMS; δ (ppm)).

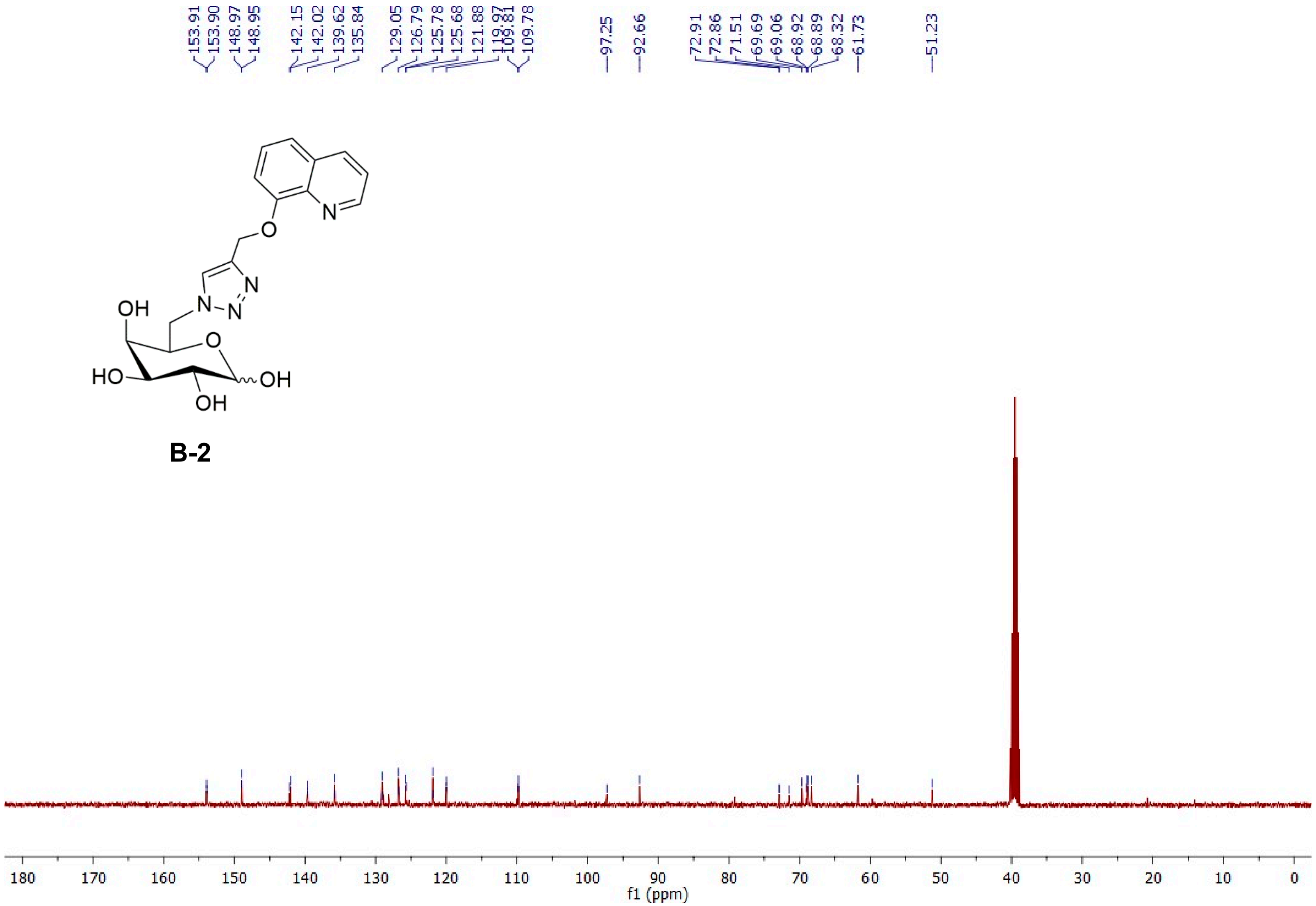

Fig. S46: ^{13}C NMR spectrum of glycoconjugates **B-2** (100 MHz/DMSO/TMS; δ (ppm)).

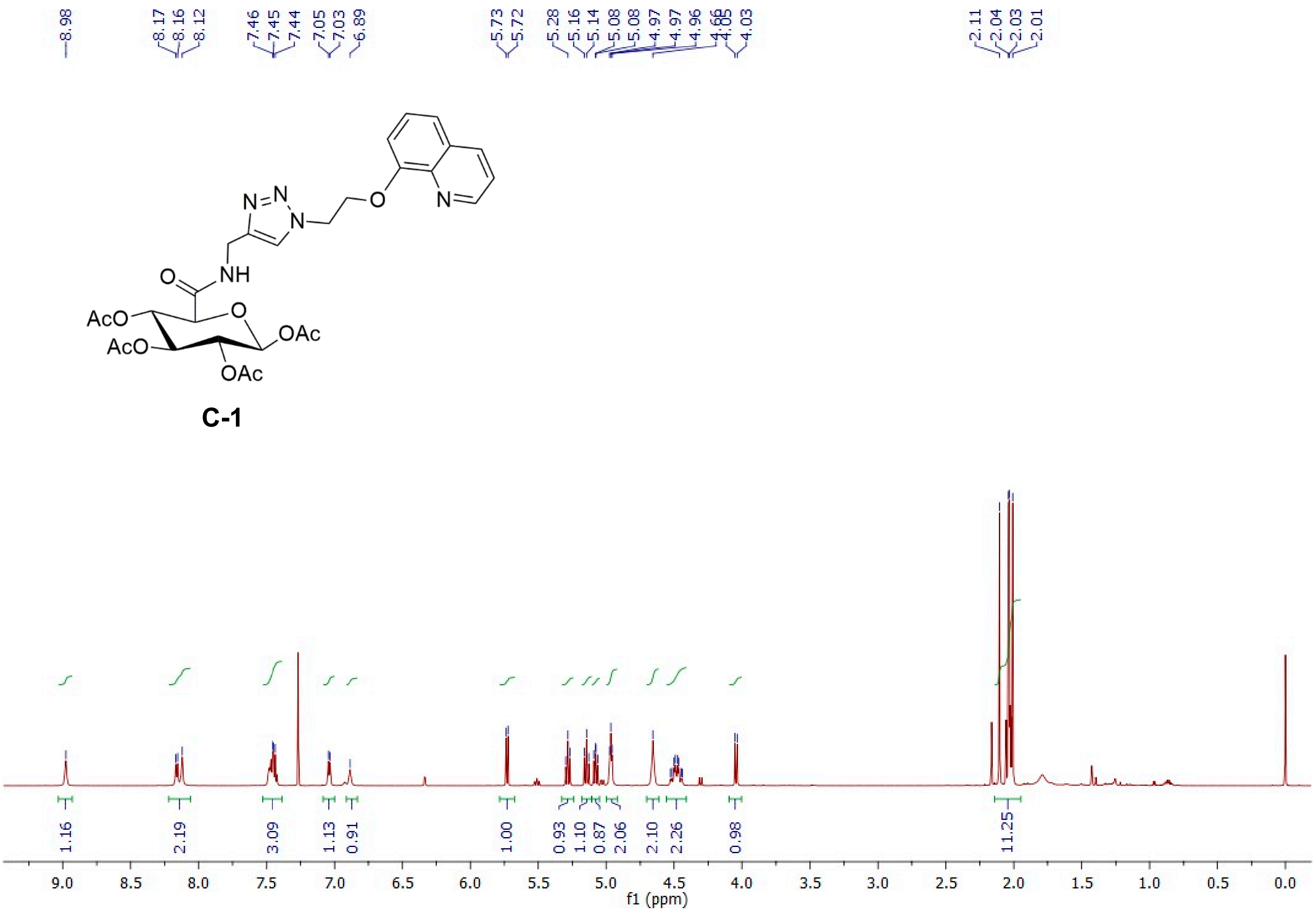

Fig. S47: ^{1}H NMR spectrum of glycoconjugates **C-1** (400 MHz/$CDCl_3$/TMS; δ (ppm)).

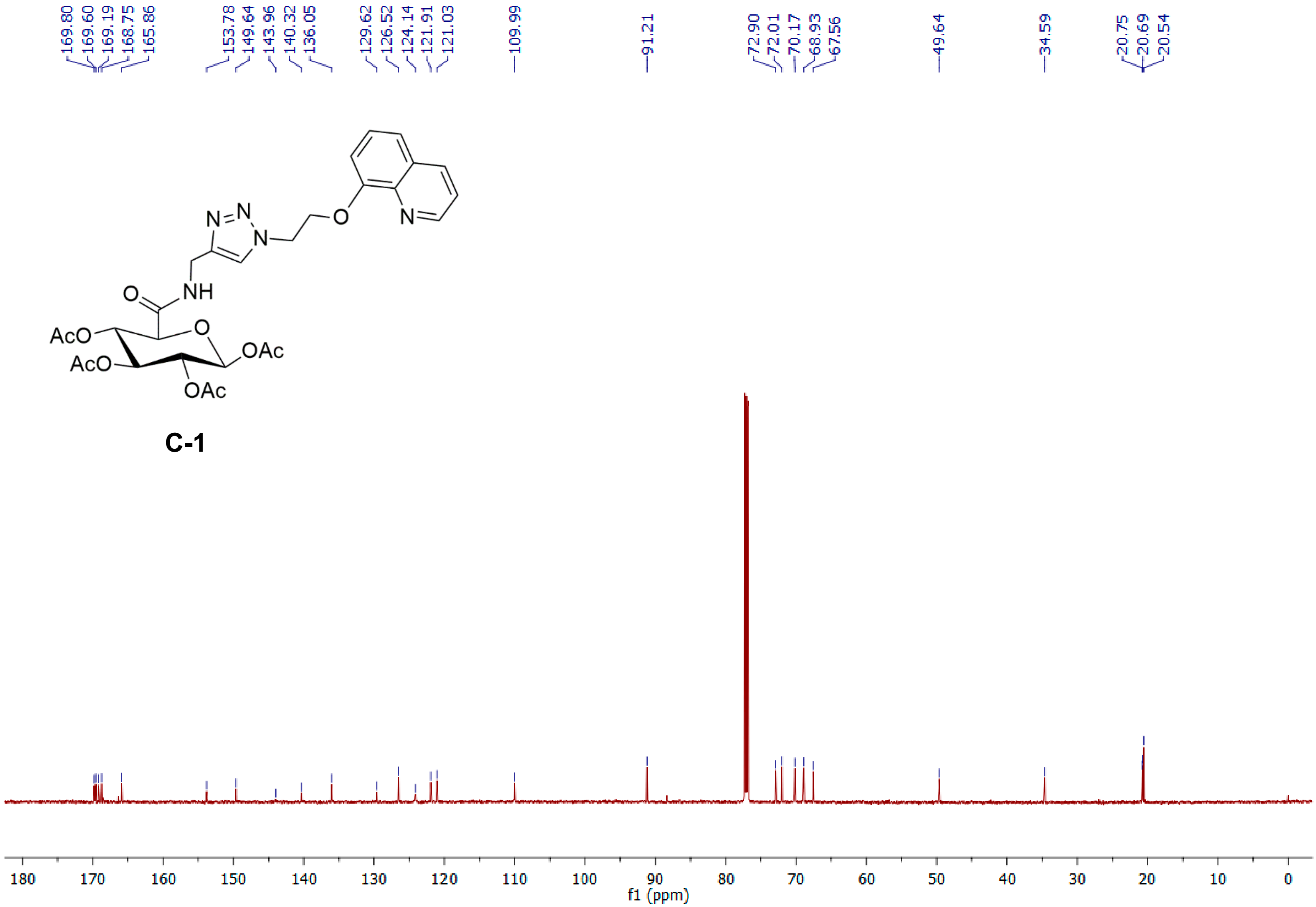

Fig. S48: ^{13}C NMR spectrum of glycoconjugates **C-1** (100 MHz/$CDCl_3$/TMS; δ (ppm)).

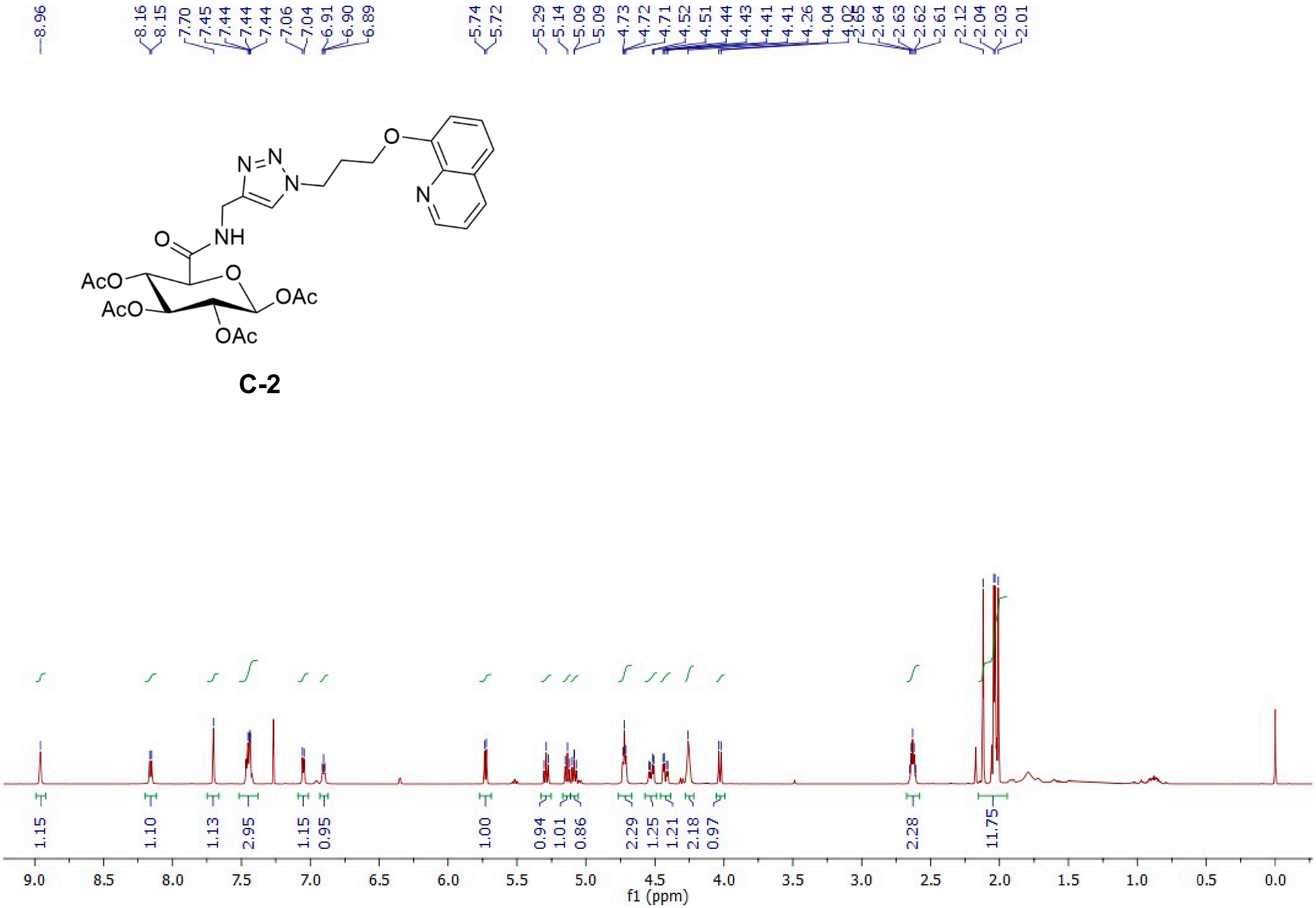

Fig. S49: ¹H NMR spectrum of glycoconjugates **C-1** (400 MHz/CDCl₃/TMS; δ (ppm)).

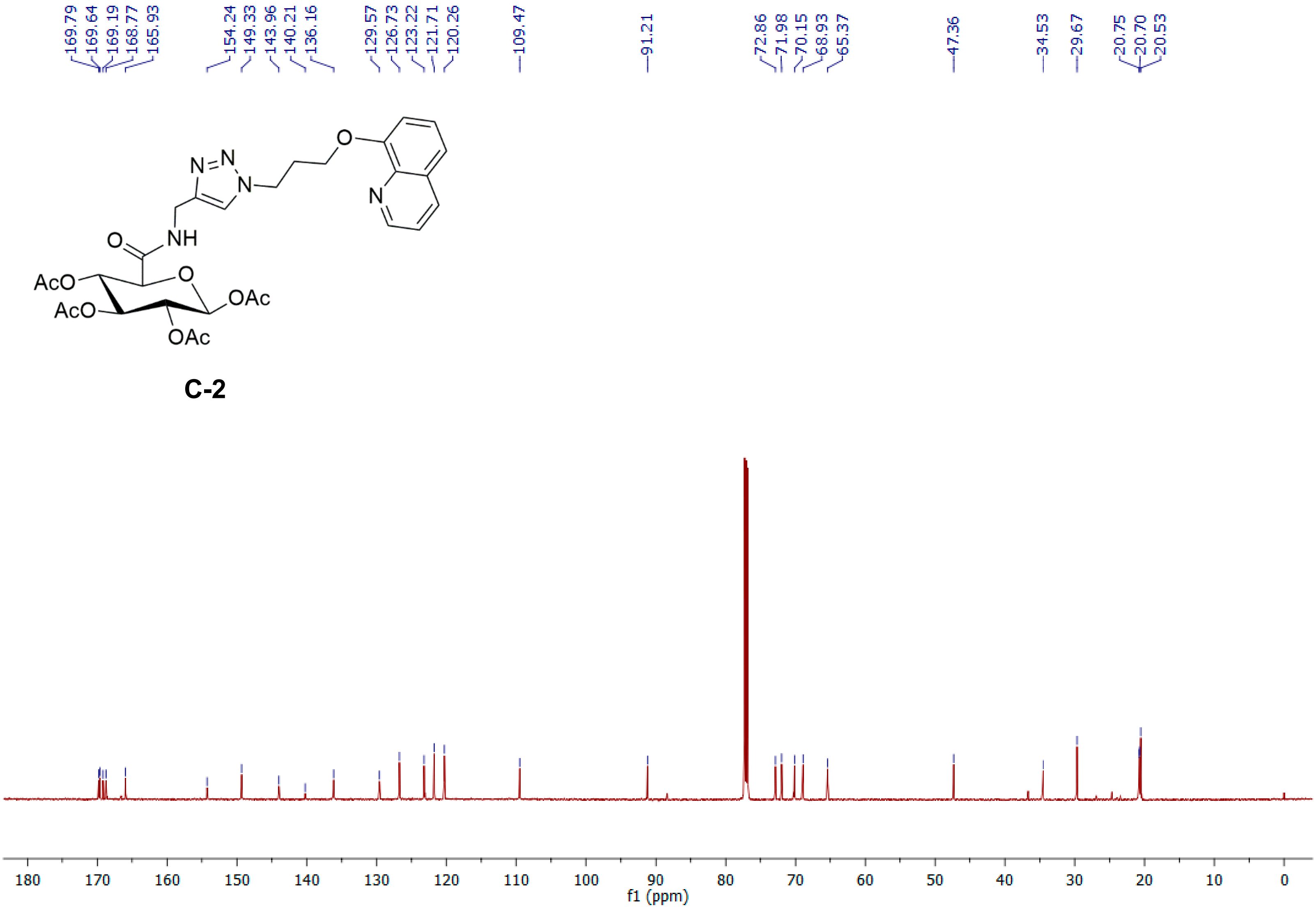

Fig. S50: ^{13}C NMR spectrum of glycoconjugates **C-1** (100 MHz/$CDCl_3$/TMS; δ (ppm)).

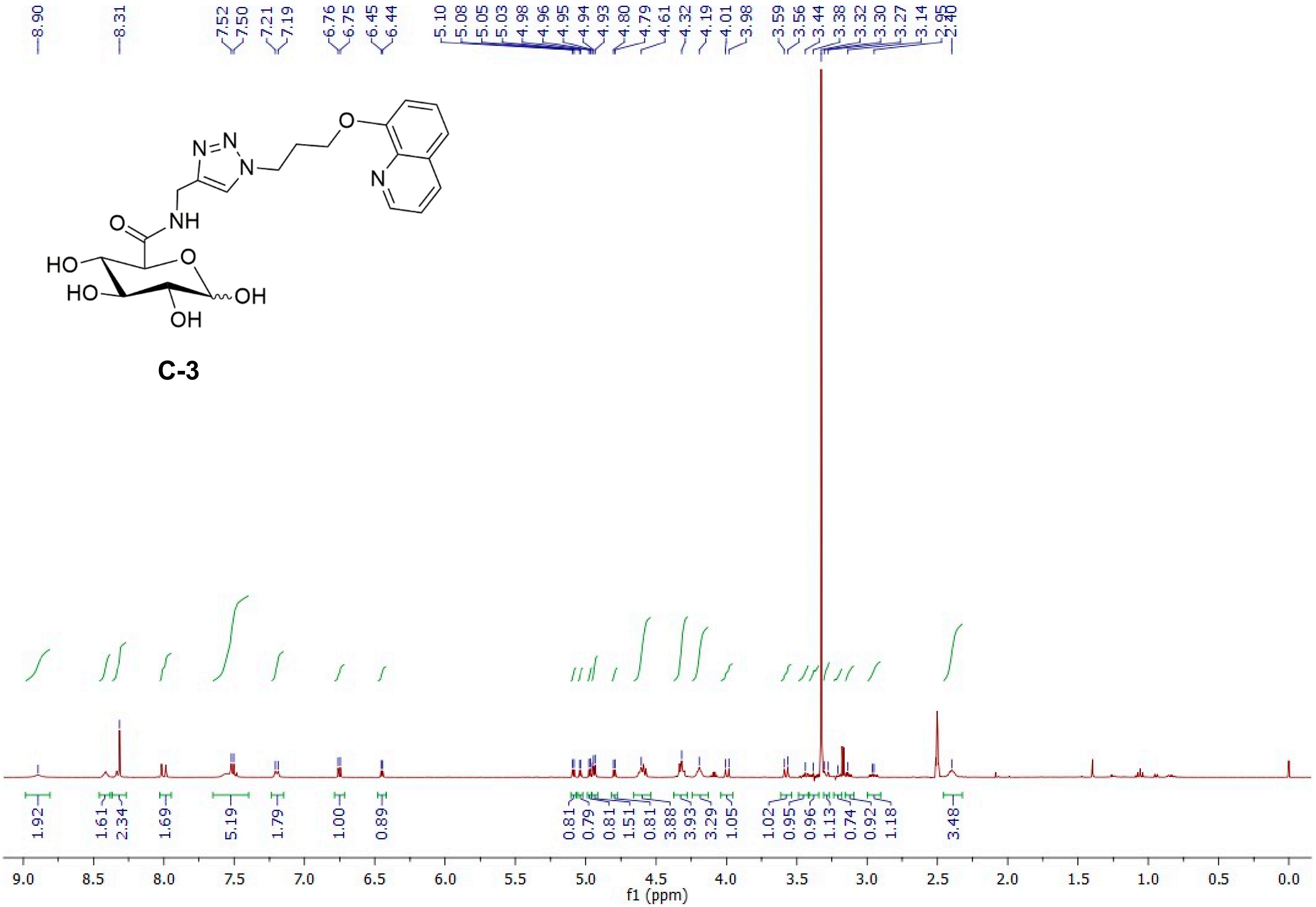

Fig. S51: ^{1}H NMR spectrum of glycoconjugates **C-3** (400 MHz/DMSO/TMS; δ (ppm)).

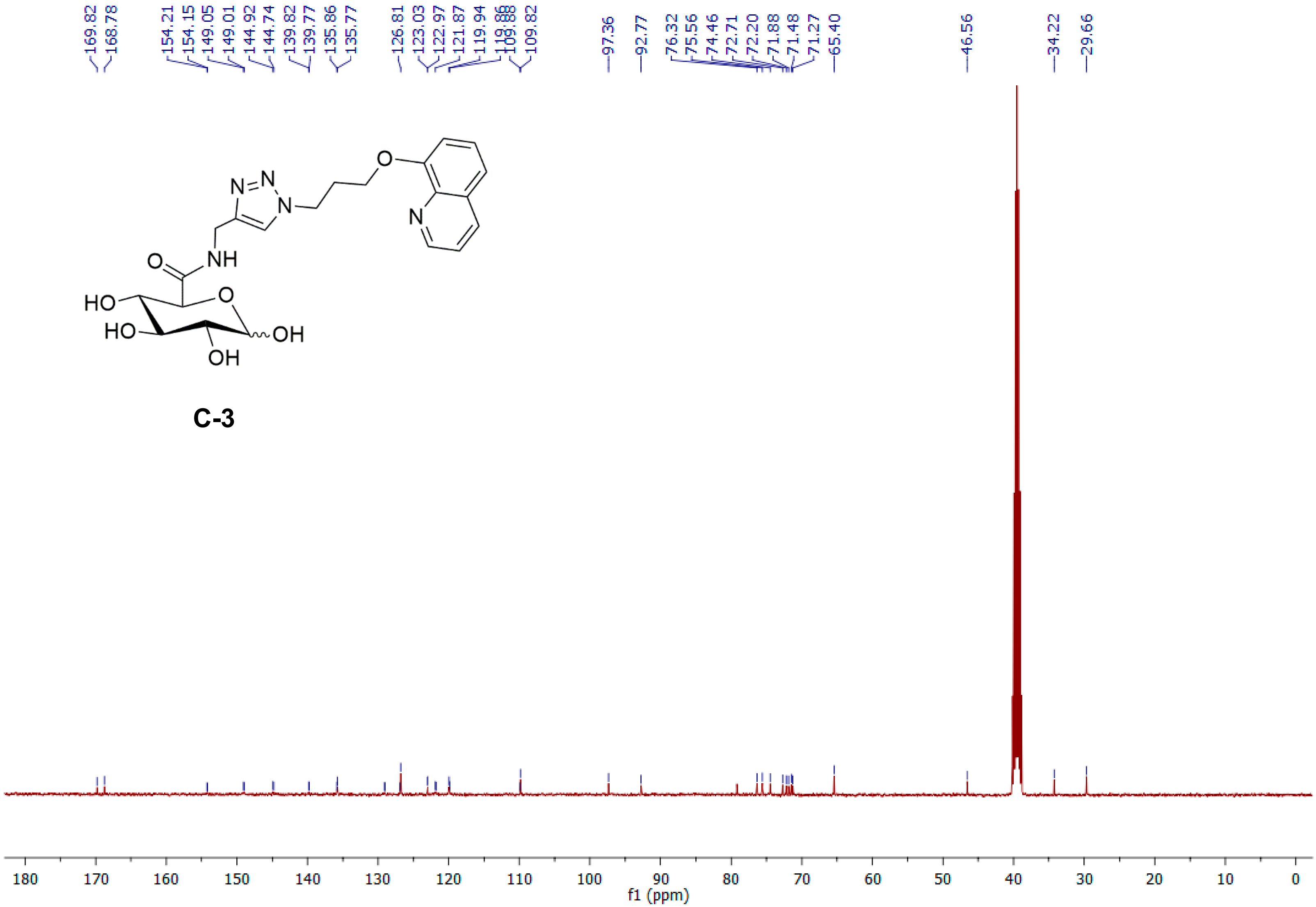

Fig. S52: ^{13}C NMR spectrum of glycoconjugates **C-3** (100 MHz/DMSO/TMS; δ (ppm)).

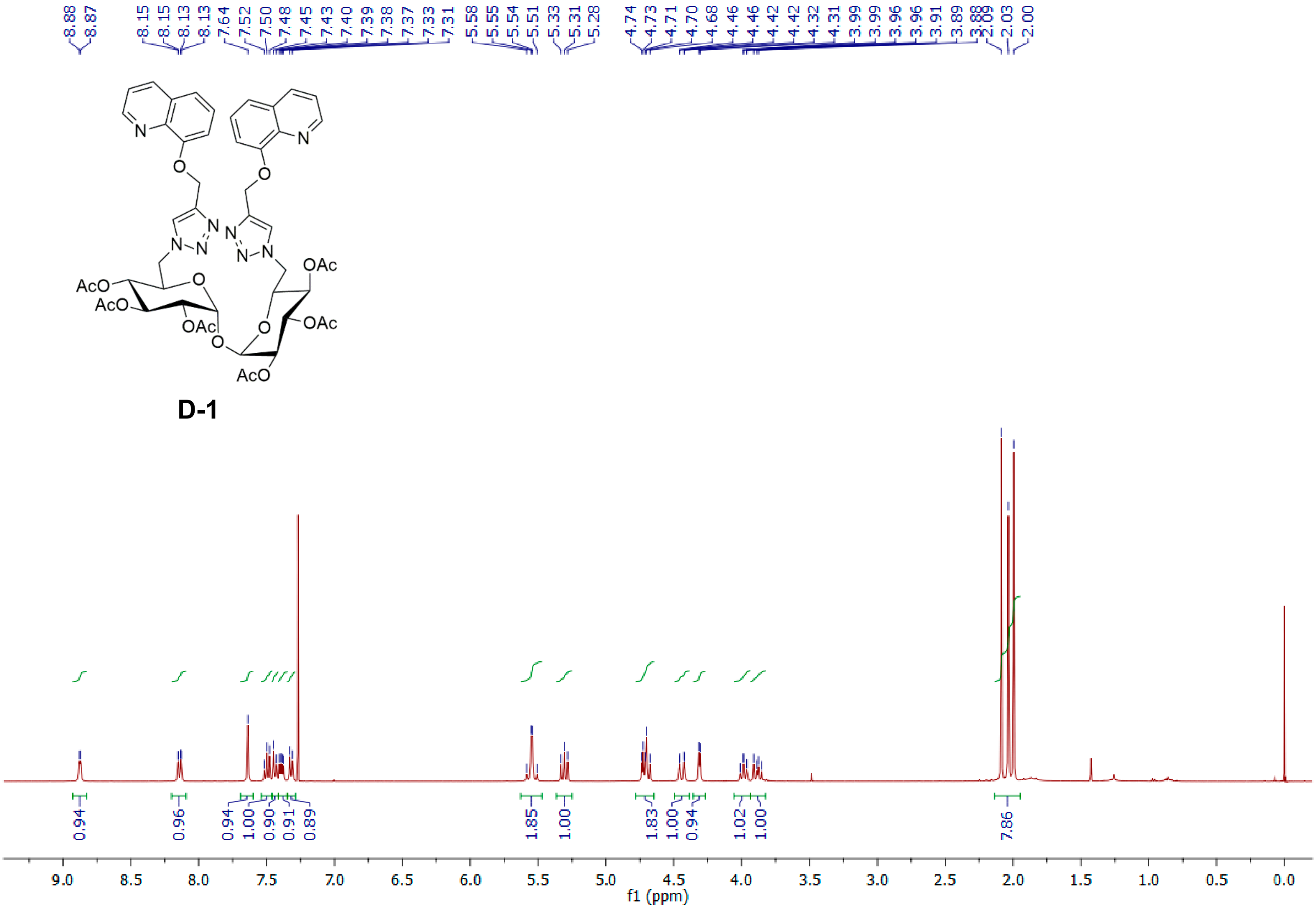

Fig. S53: ^{1}H NMR spectrum of glycoconjugates **D-1** (400 MHz/$CDCl_3$/TMS; δ (ppm)).

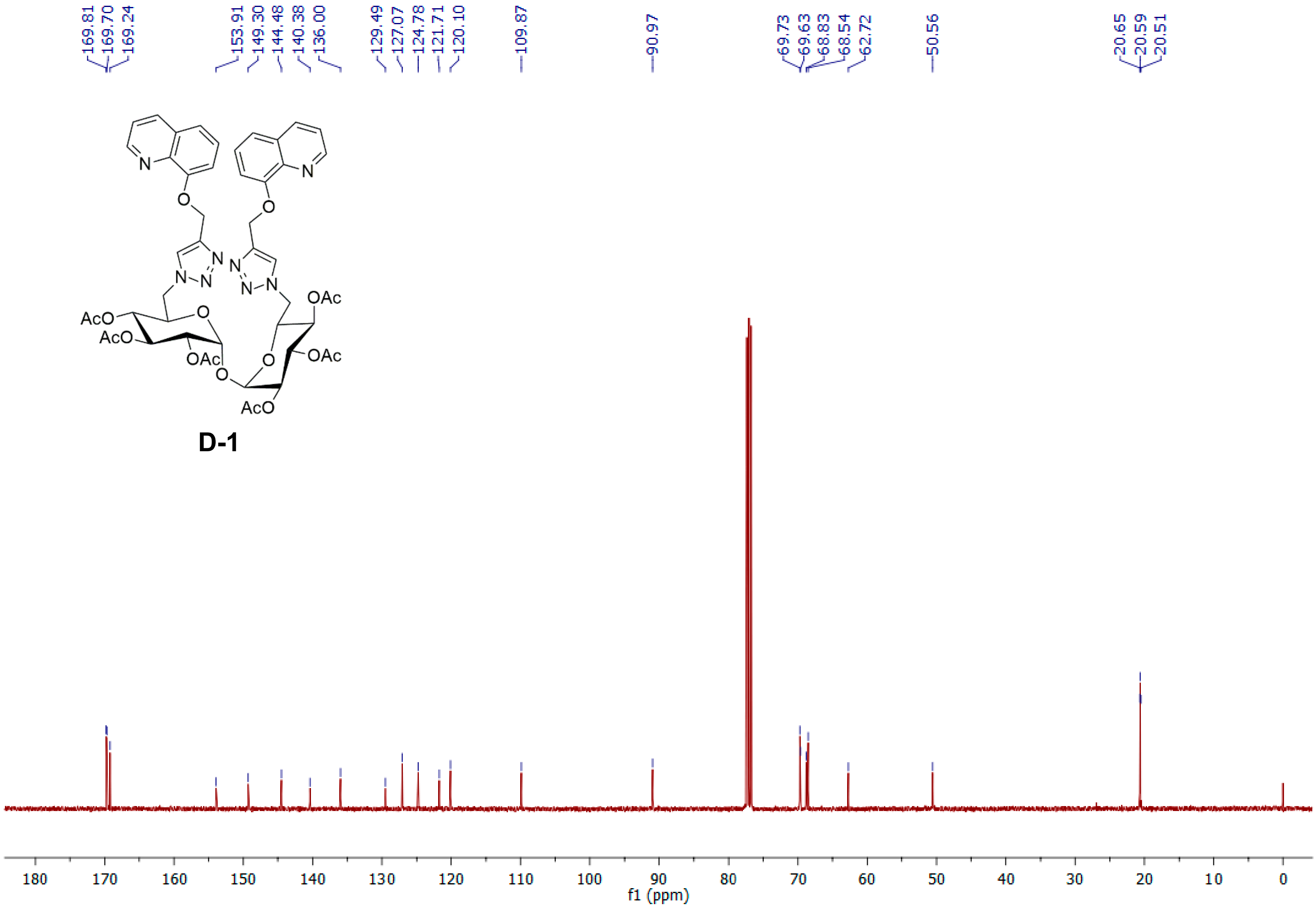

Fig. S54: ^{13}C NMR spectrum of glycoconjugates **D-1** (100 MHz/$CDCl_3$/TMS; δ (ppm)).

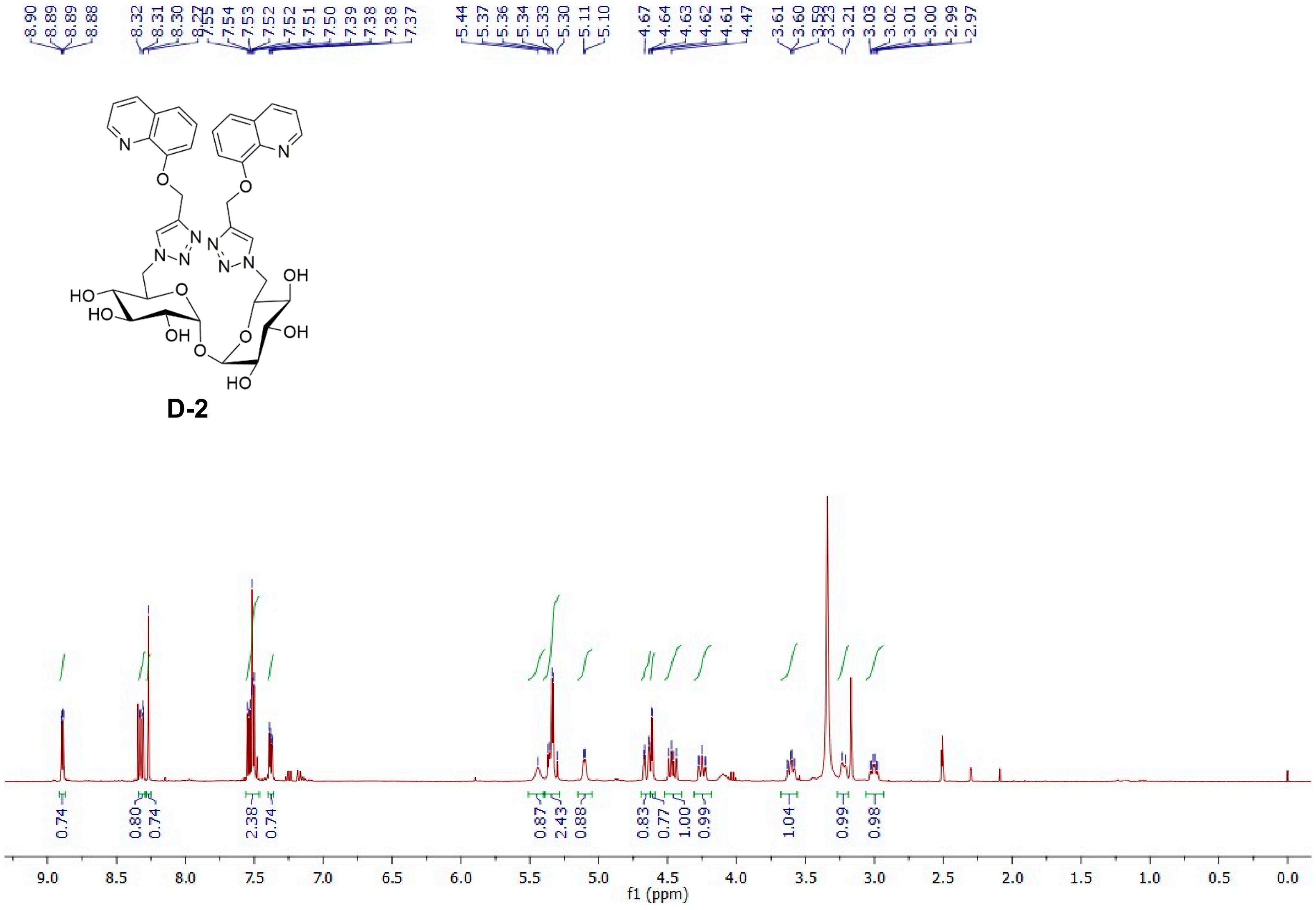

Fig. S55: ¹H NMR spectrum of glycoconjugates **D-2** (400 MHz/DMSO/TMS; δ (ppm)).

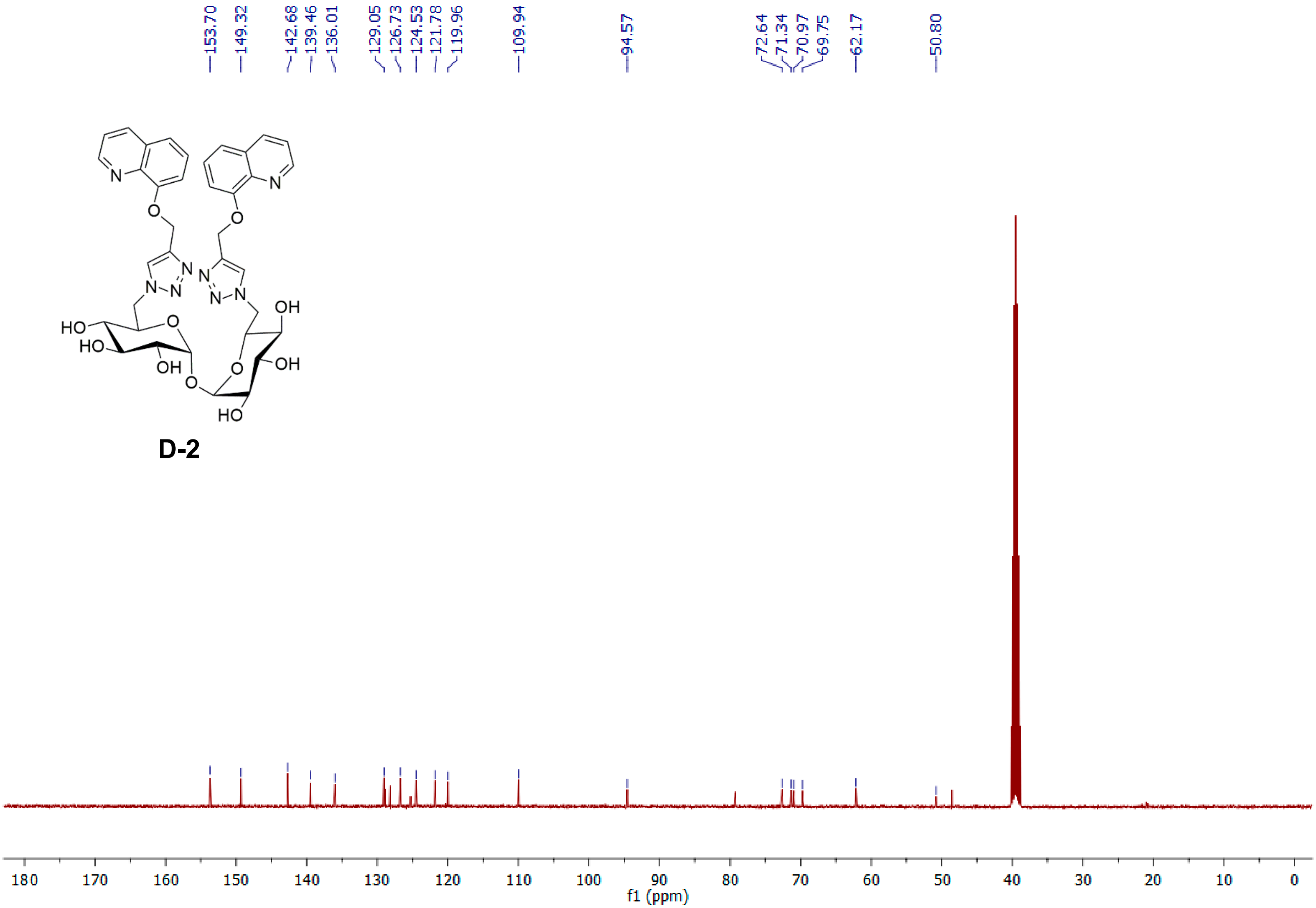

Fig. S56: ^{13}C NMR spectrum of glycoconjugates **D-2** (100 MHz/DMSO/TMS; δ (ppm)).

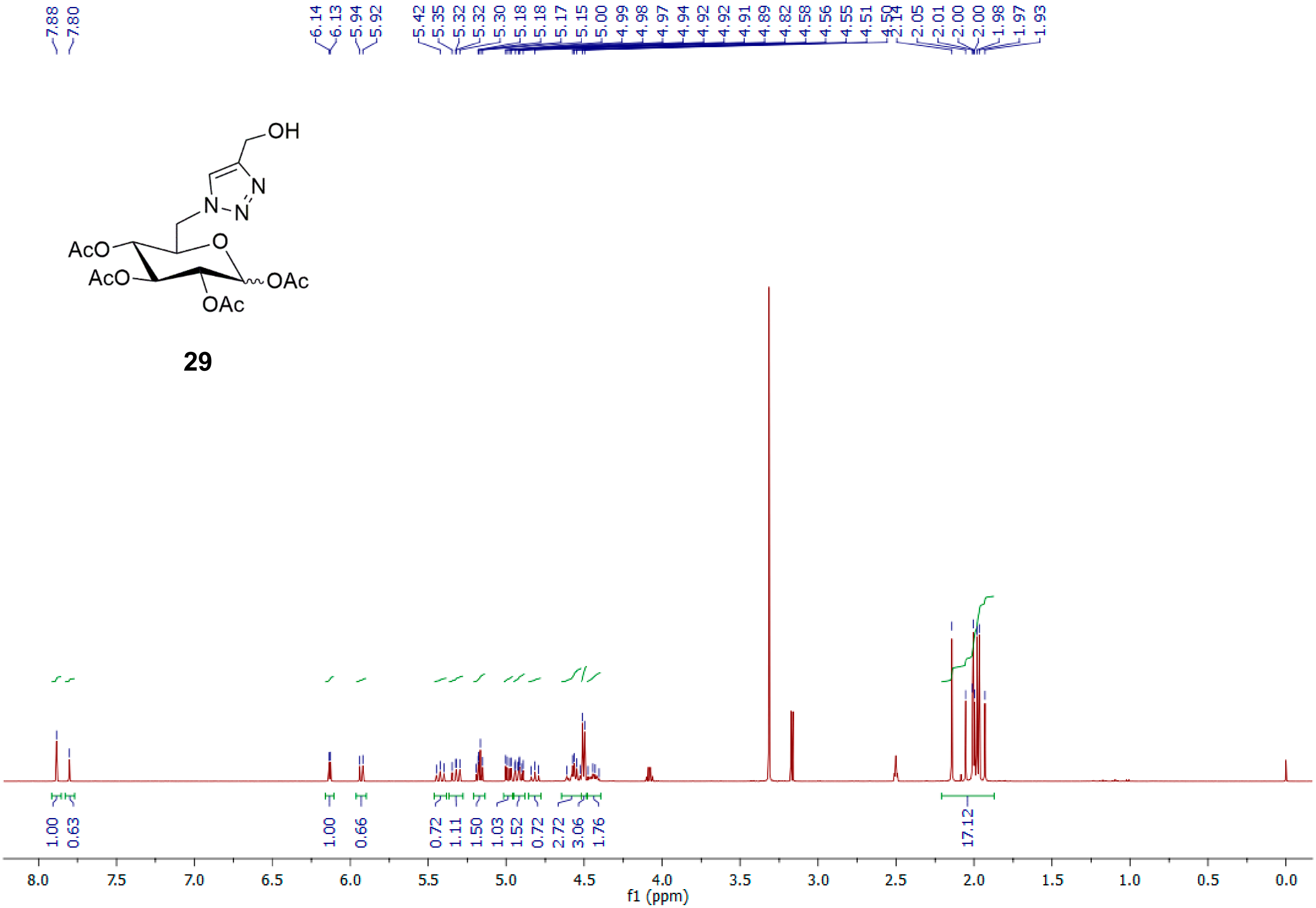

Fig. S57: ^{1}H NMR spectrum of metabolite **29** (400 MHz/DMSO/TMS; δ (ppm)).

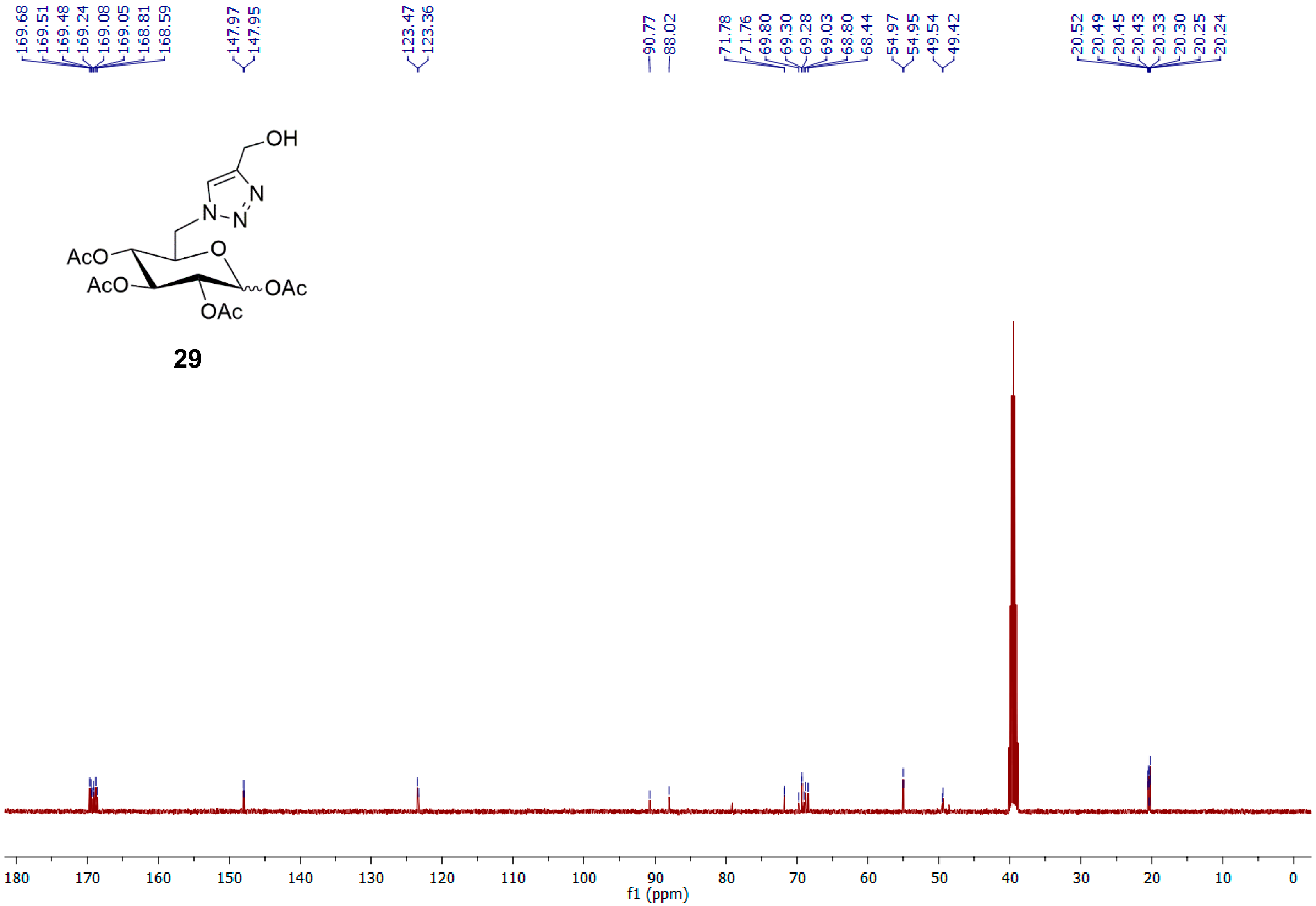

Fig. S58: ^{13}C NMR spectrum of metabolite **29** (100 MHz/DMSO/TMS; δ (ppm)).

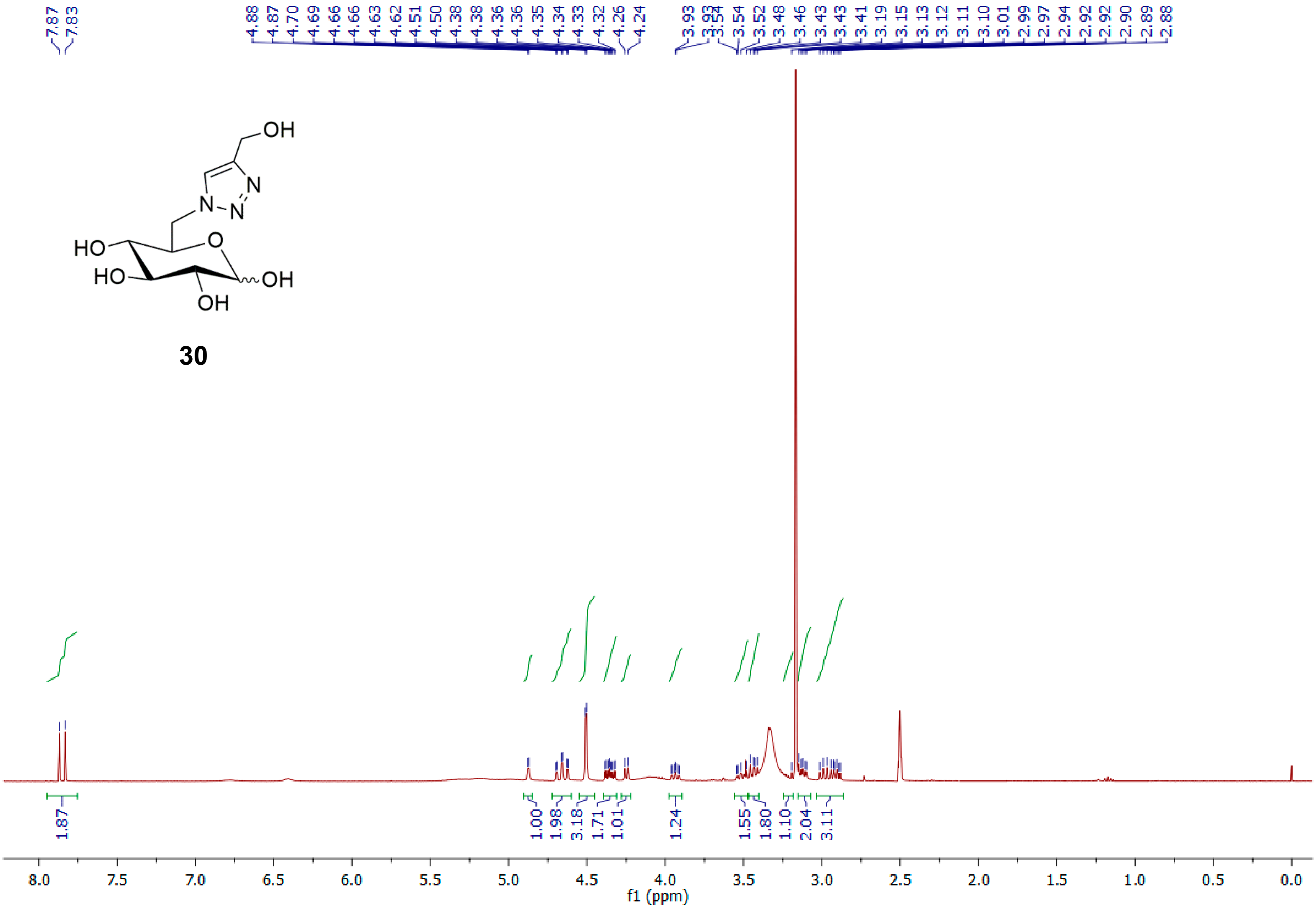

Fig. S59: ^{1}H NMR spectrum of metabolite **29** (400 MHz/DMSO/TMS; δ (ppm)).

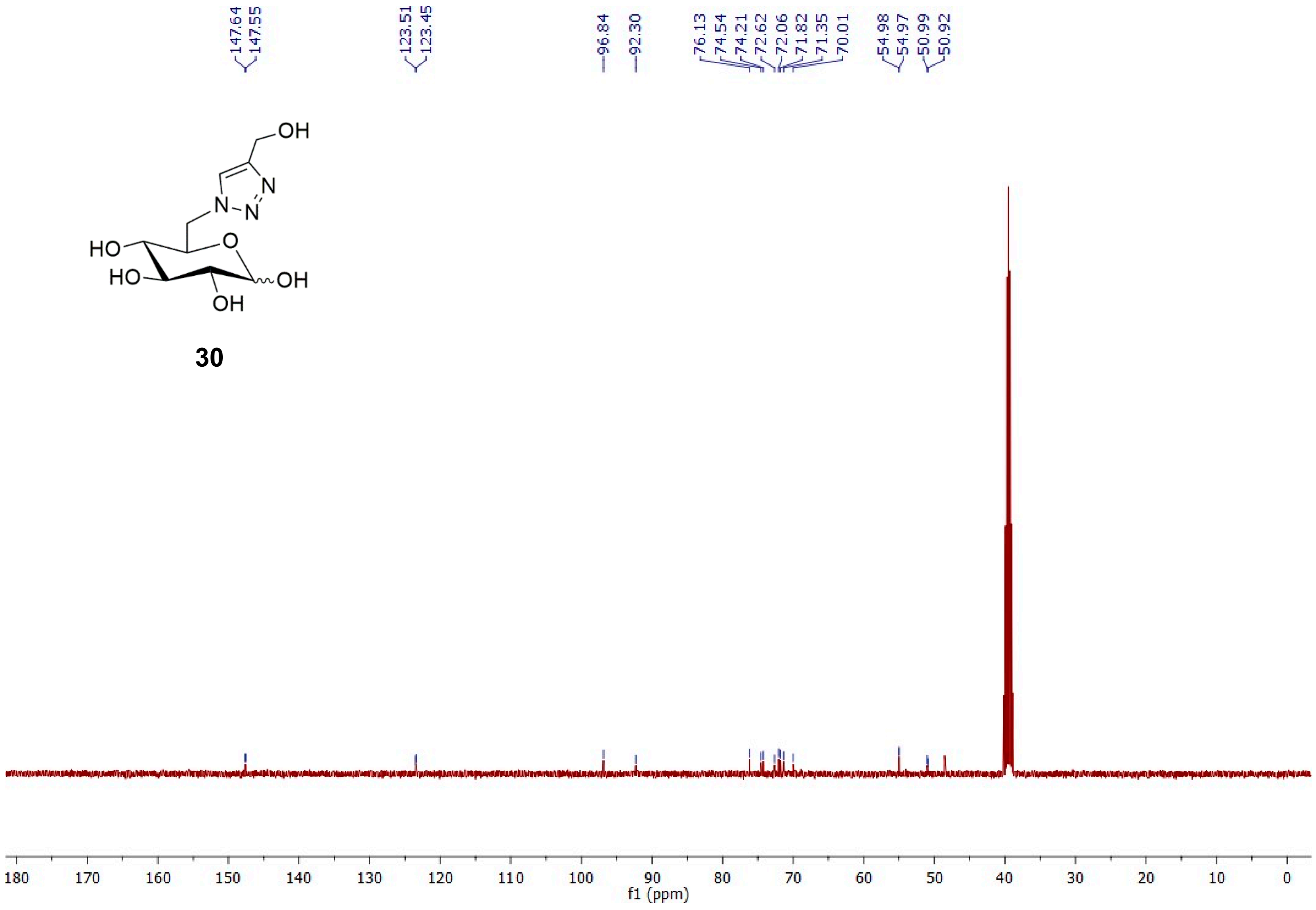

Fig. S60: ^{13}C NMR spectrum of metabolite **29** (100 MHz/DMSO/TMS; δ (ppm)).

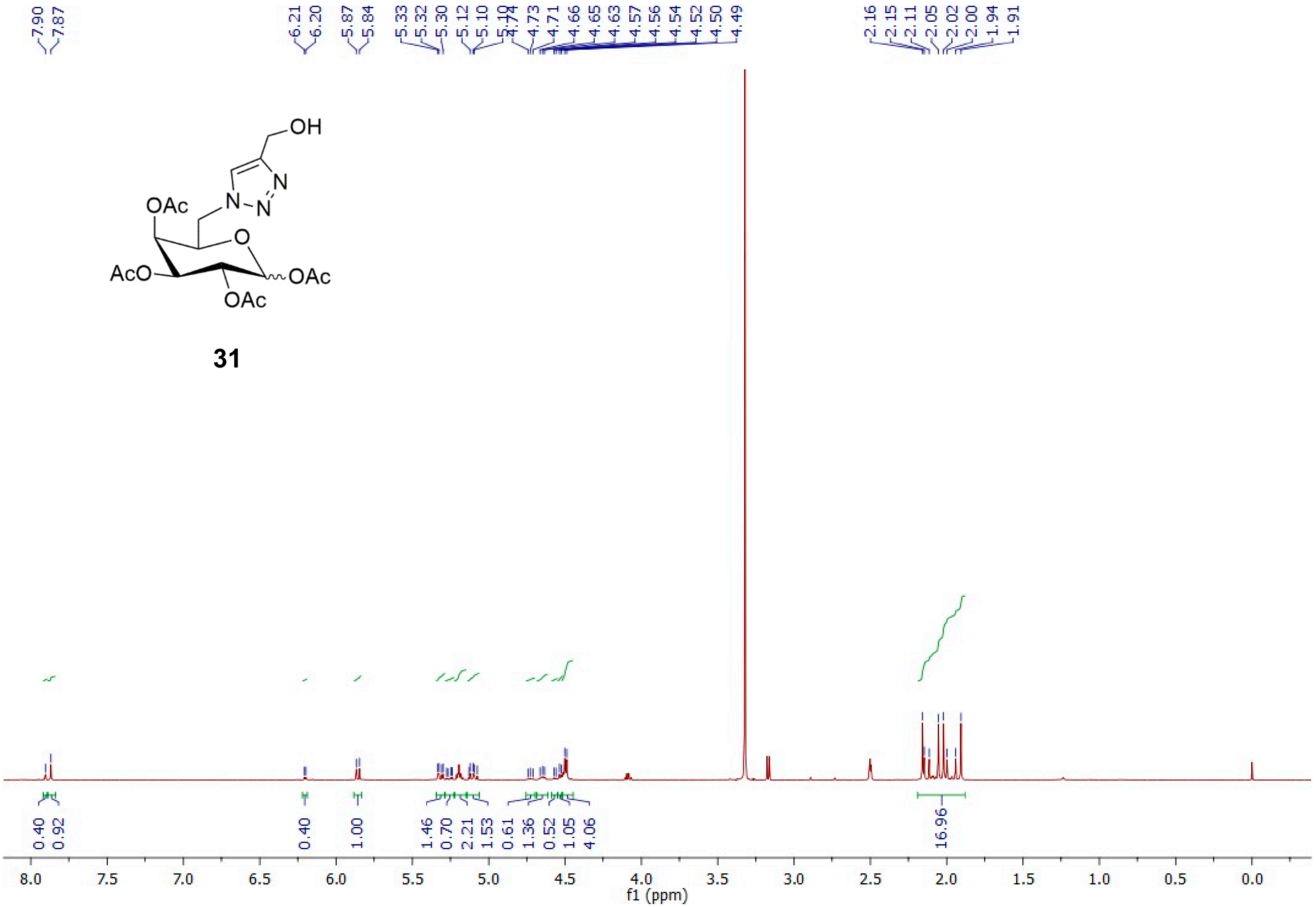

Fig. S61: ^{1}H NMR spectrum of metabolite **32** (400 MHz/DMSO/TMS; δ (ppm)).

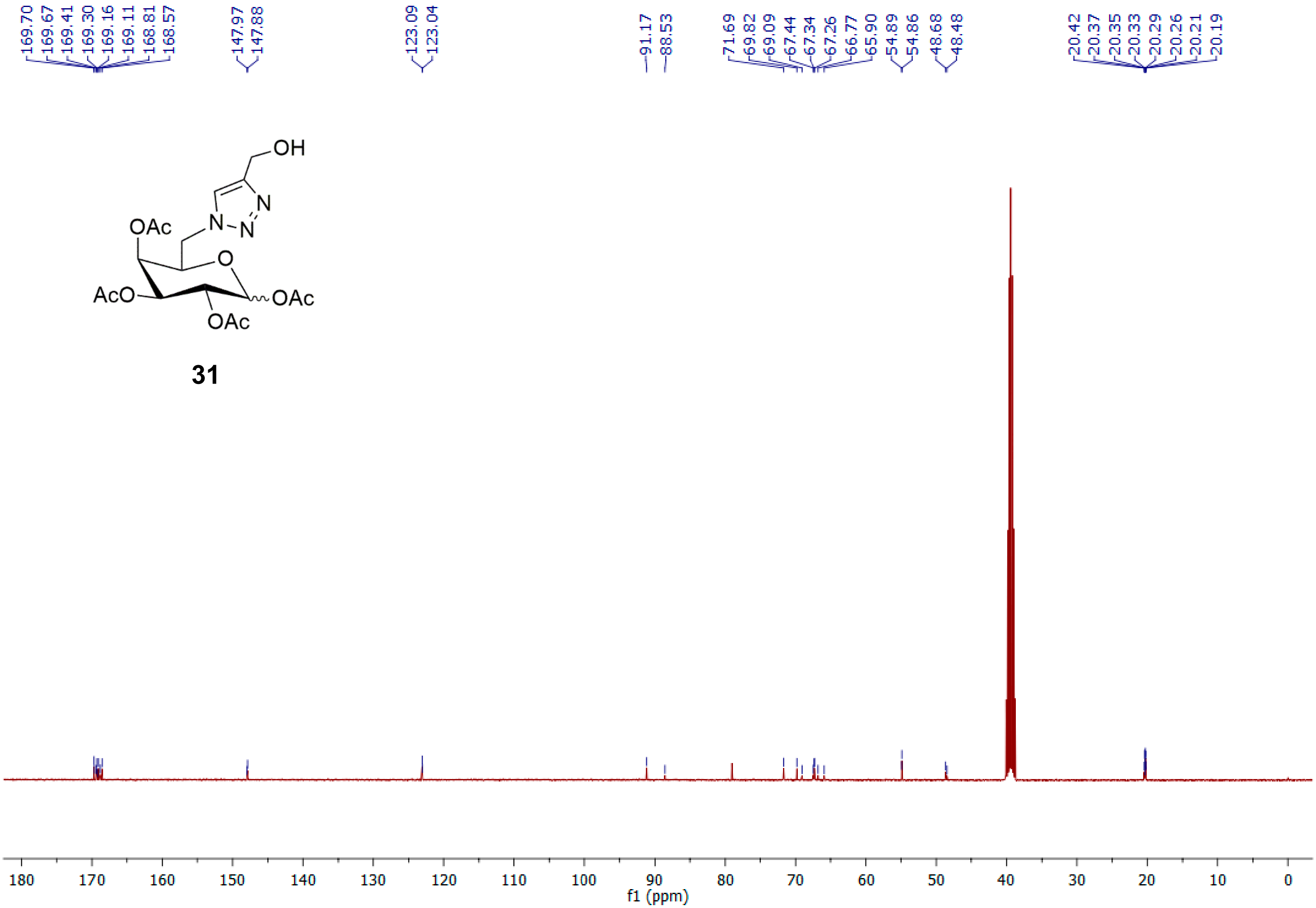

Fig. S62: ^{13}C NMR spectrum of metabolite **31** (100 MHz/DMSO/TMS; δ (ppm)).

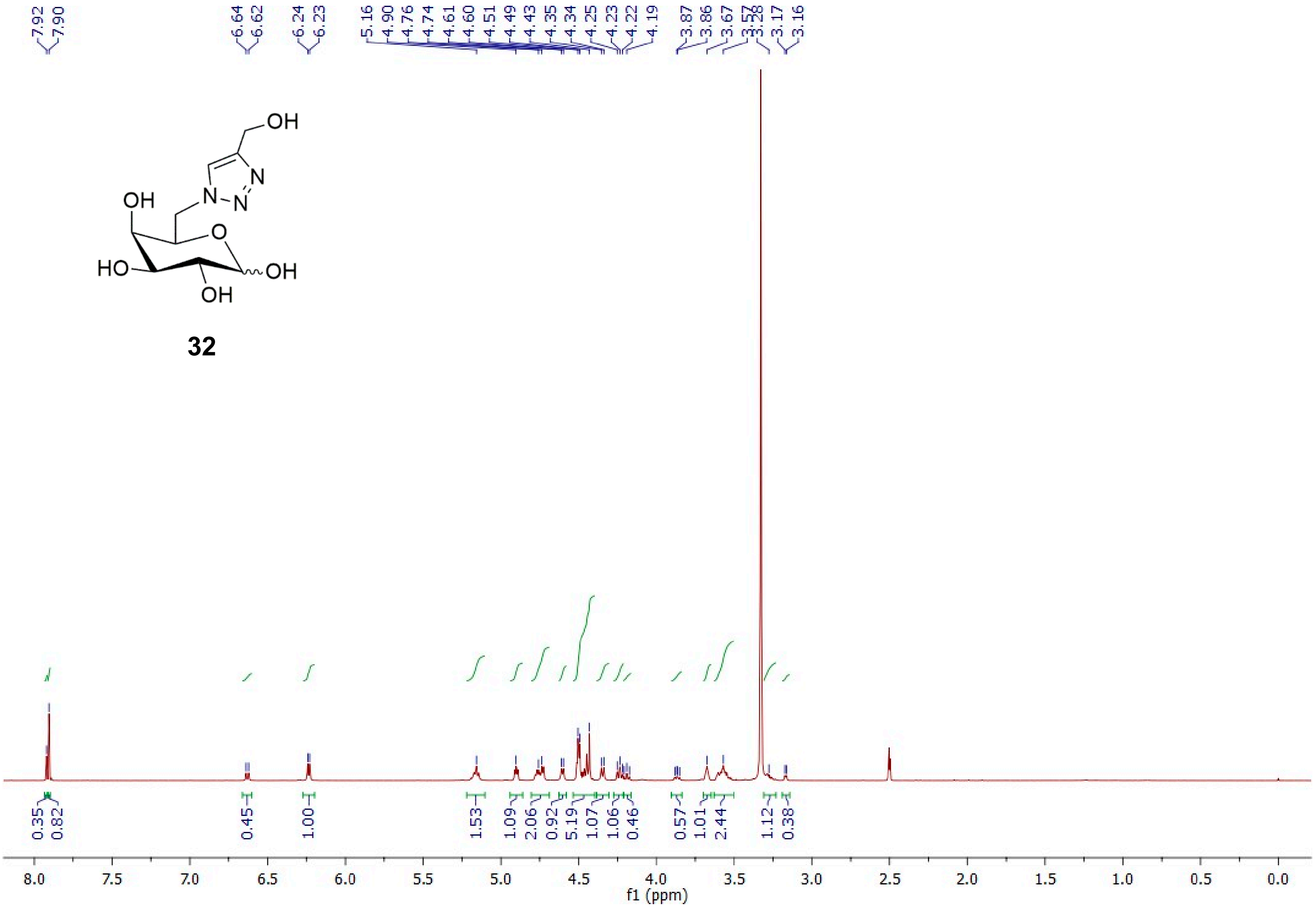

Fig. S63: ^{1}H NMR spectrum of metabolite **32** (400 MHz/DMSO/TMS; δ (ppm)).

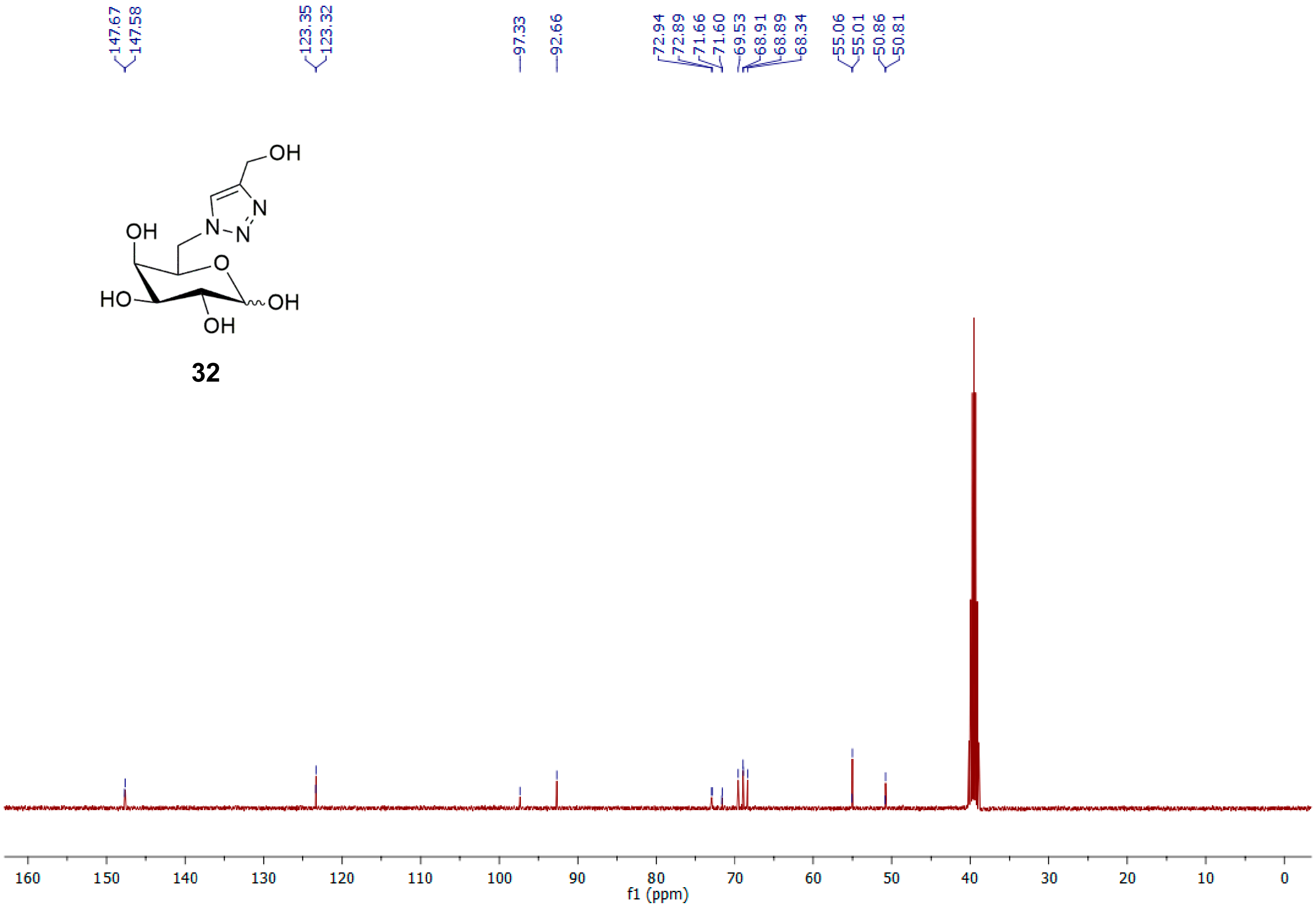

Fig. S64: ^{13}C NMR spectrum of metabolite **31** (100 MHz/DMSO/TMS; δ (ppm)).

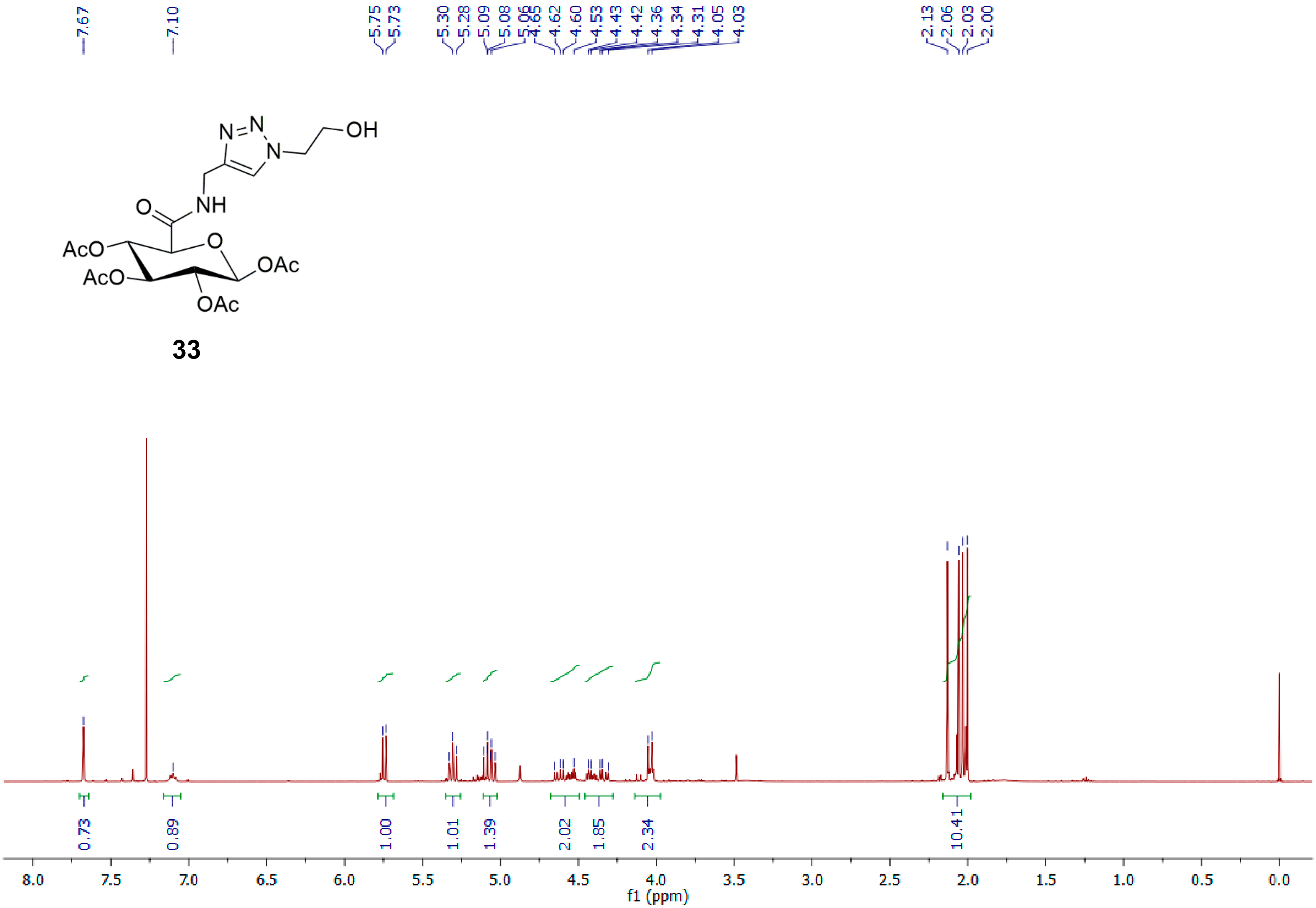

Fig. S65: ^{1}H NMR spectrum of metabolite **33** (400 MHz/CDCl$_3$/TMS; δ (ppm)).

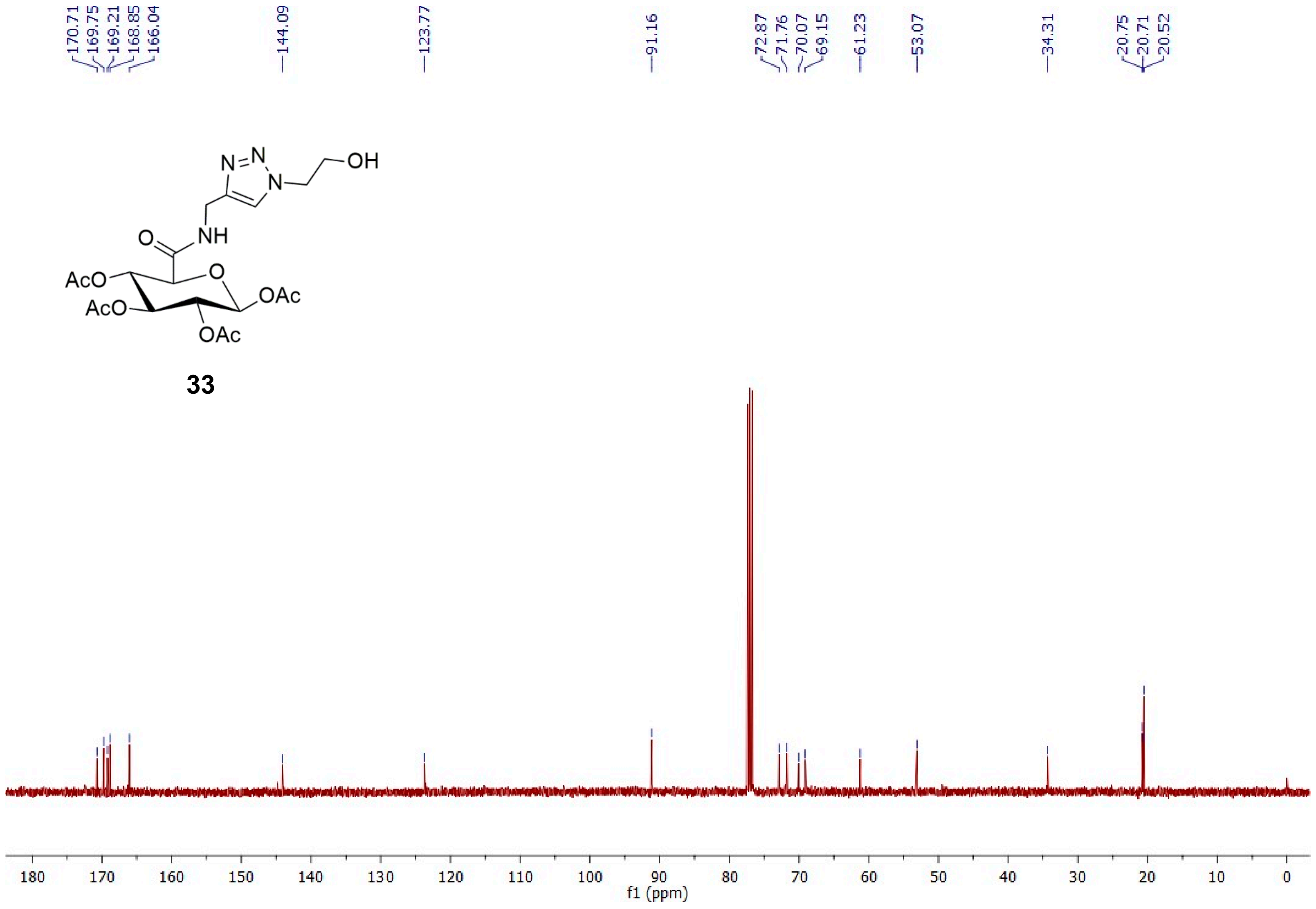

Fig. S66: ^{13}C NMR spectrum of metabolite **33** (100 MHz/$CDCl_3$/TMS; δ (ppm)).

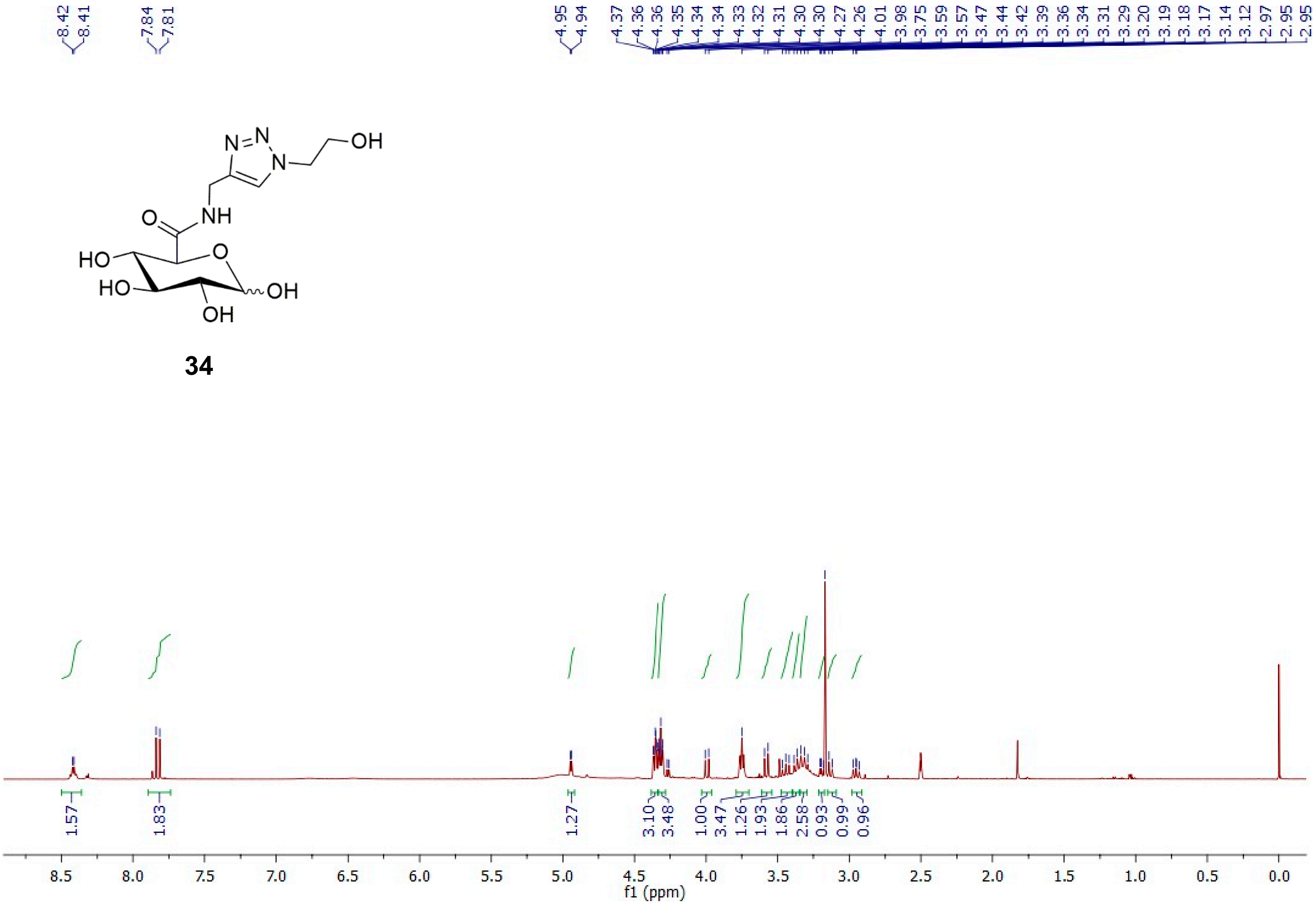

Fig. S67: ¹H NMR spectrum of metabolite **35** (400 MHz/DMSO/TMS; δ (ppm)).

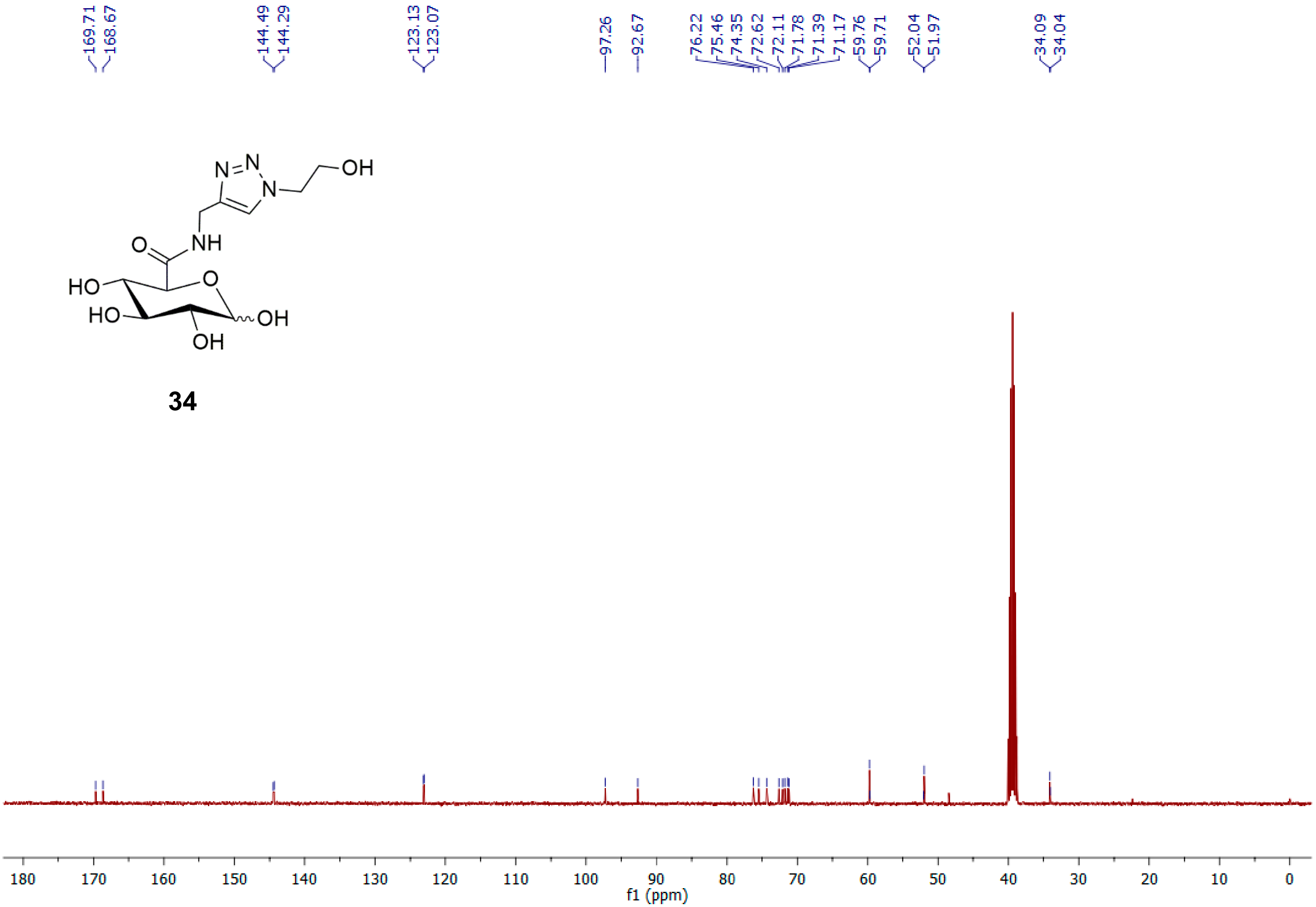

Fig. S68: ^{13}C NMR spectrum of metabolite **34** (100 MHz/DMSO/TMS; δ (ppm)).

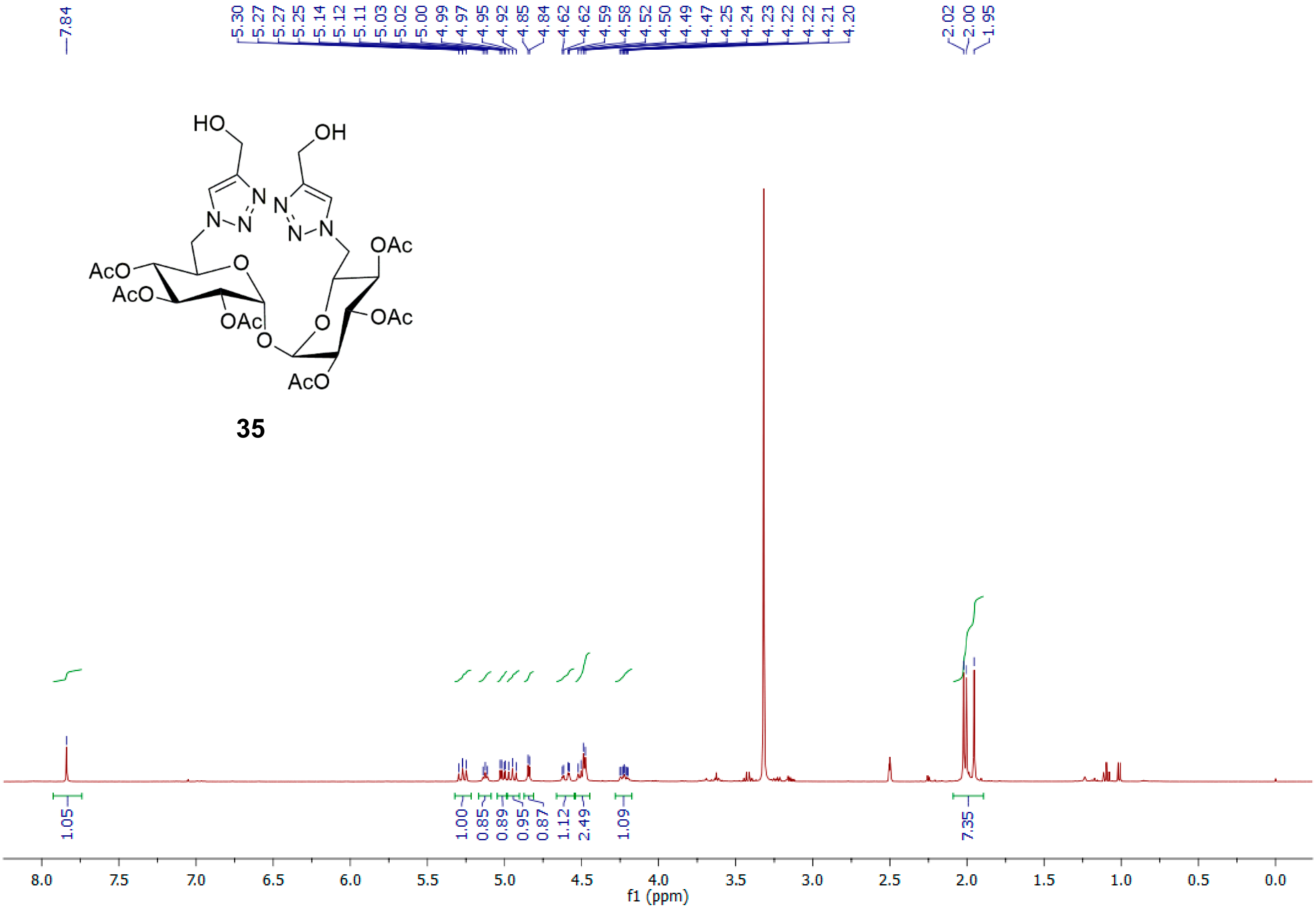

35

Fig. S69: ^{1}H NMR spectrum of metabolite **35** (400 MHz/DMSO/TMS; δ (ppm)).

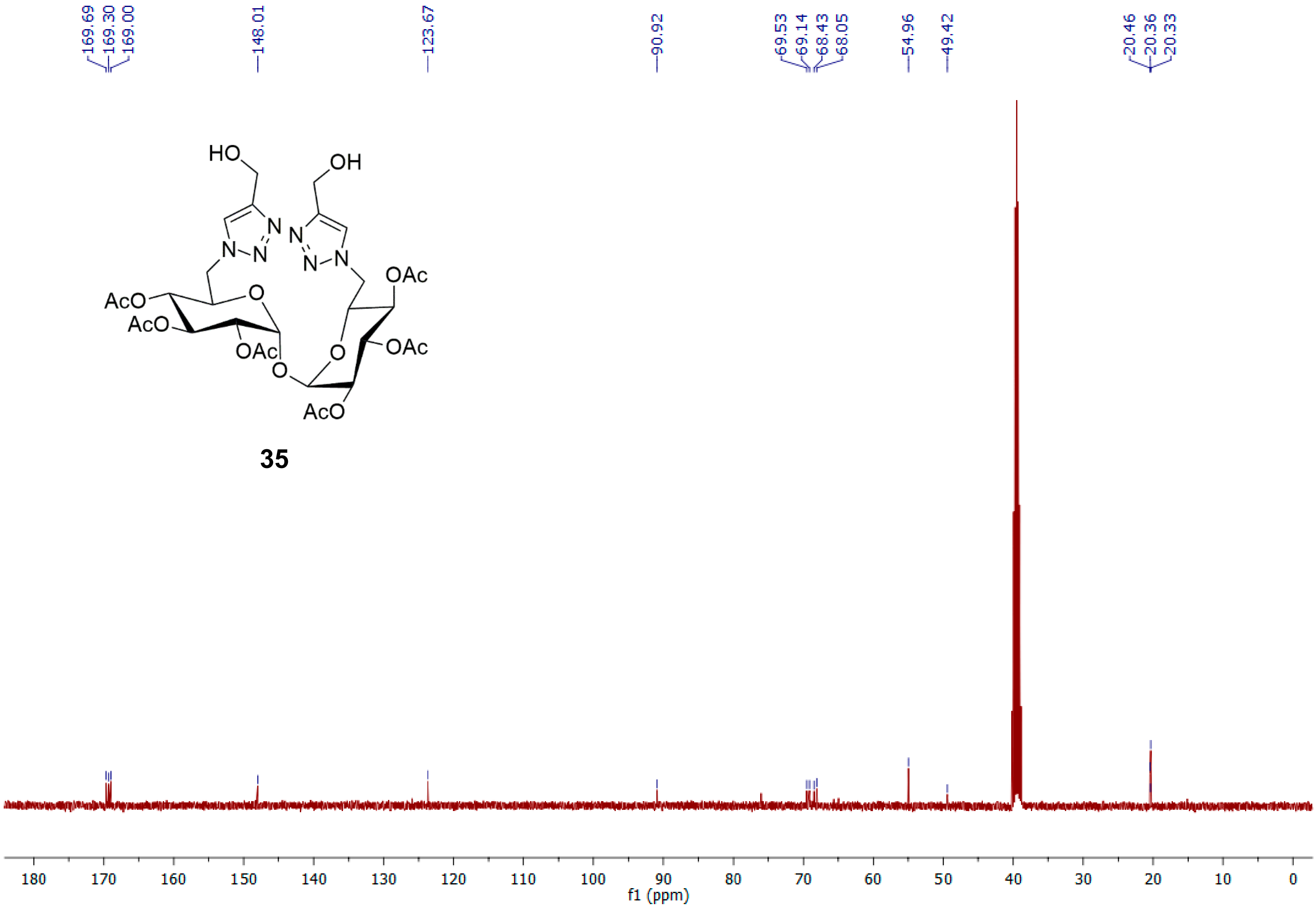

Fig. S70: ^{13}C NMR spectrum of metabolite **35** (100 MHz/DMSO/TMS; δ (ppm)).

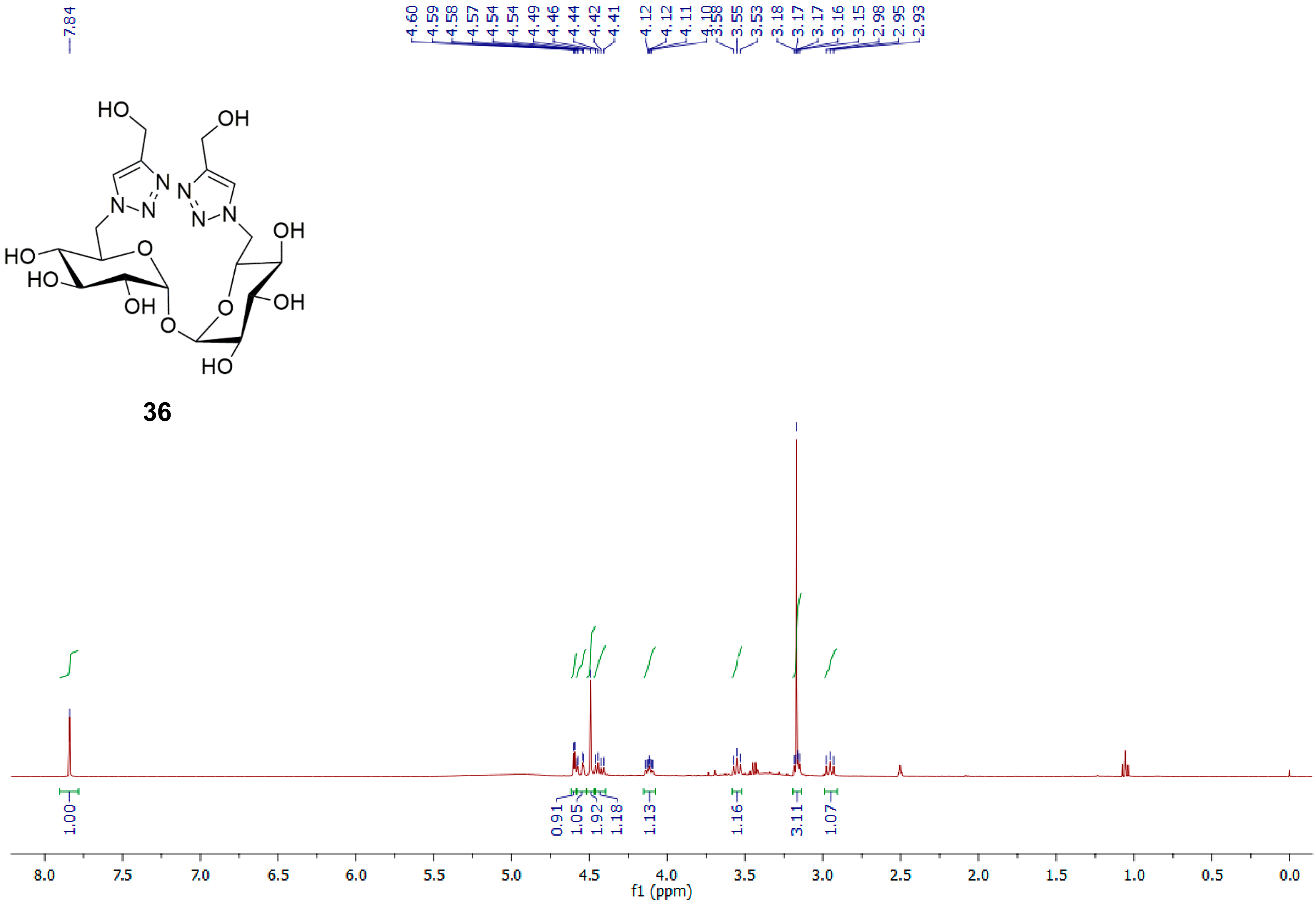

36

Fig. S71: ^{1}H NMR spectrum of metabolite **36** (400 MHz/DMSO/TMS; δ (ppm)).

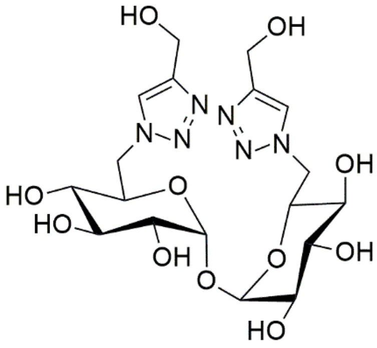

36

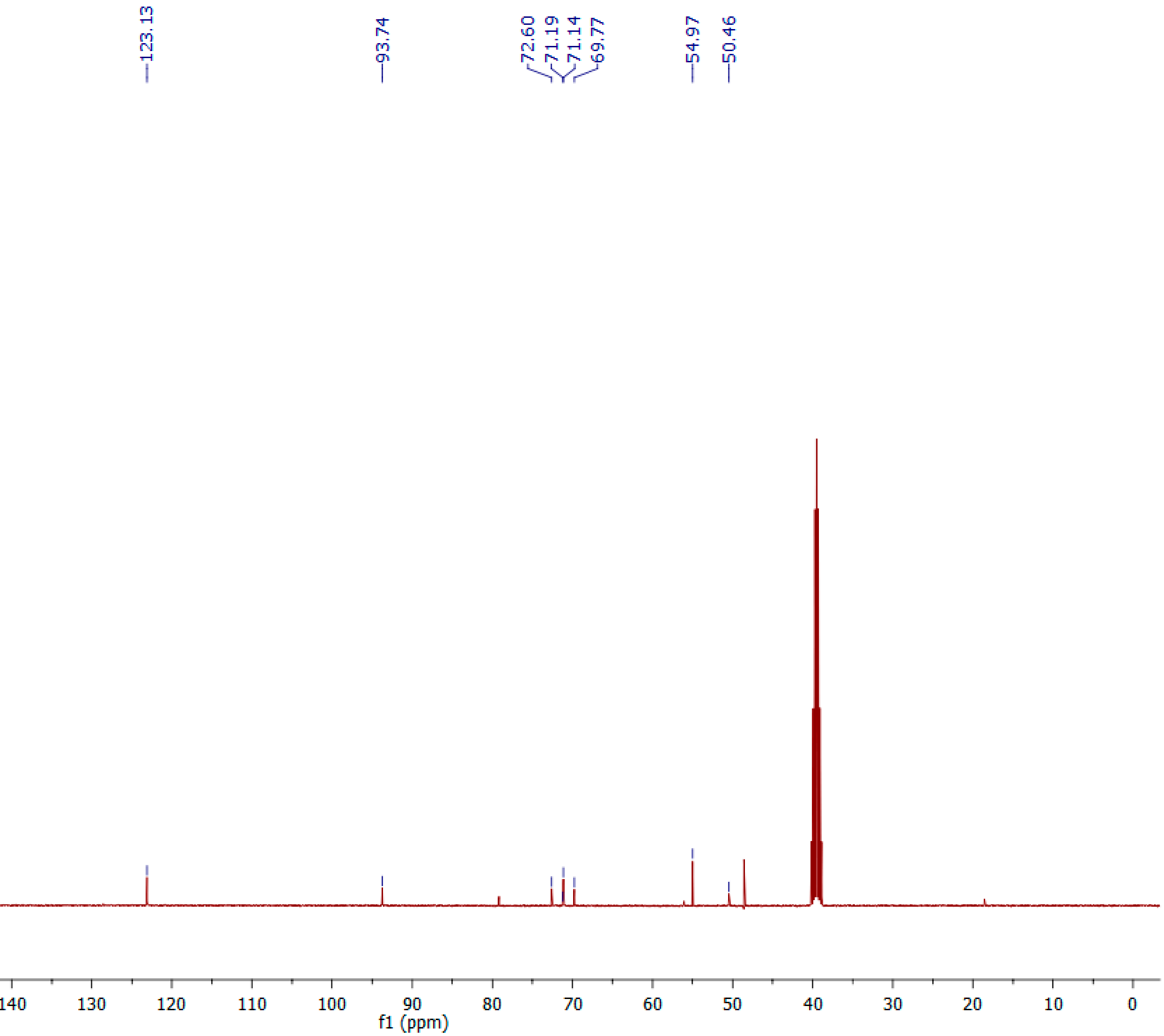

Fig. S72: ^{13}C NMR spectrum of metabolite **36** (100 MHz/DMSO/TMS; δ (ppm))

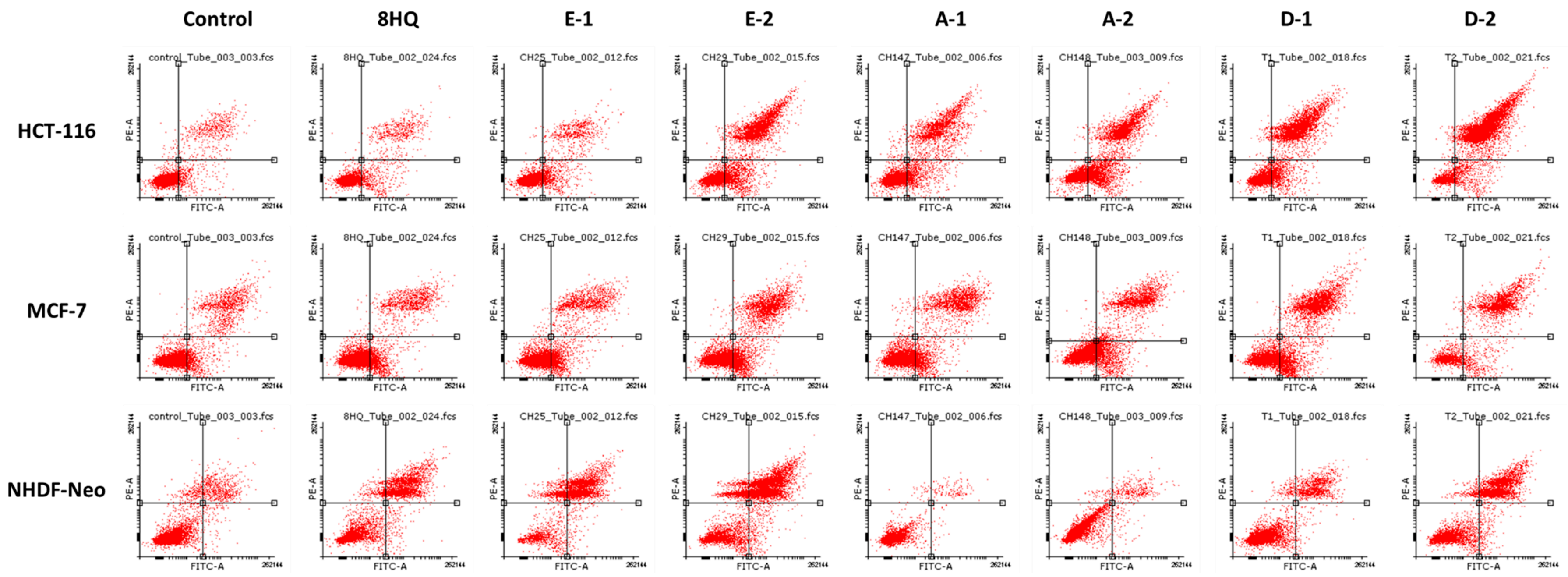

Fig. S73: Representative graphs of Annexin V/PI double staining apoptosis assay.

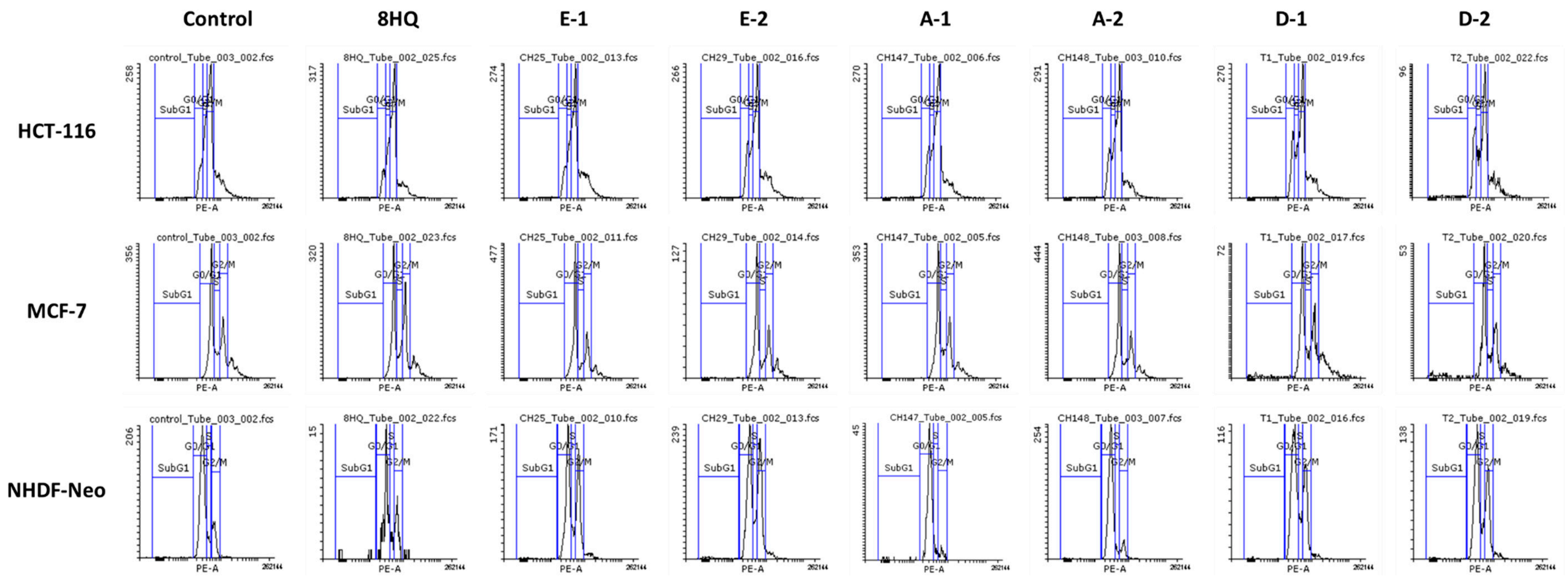

Fig. S74: Representative histograms of PI-stained DNA content.

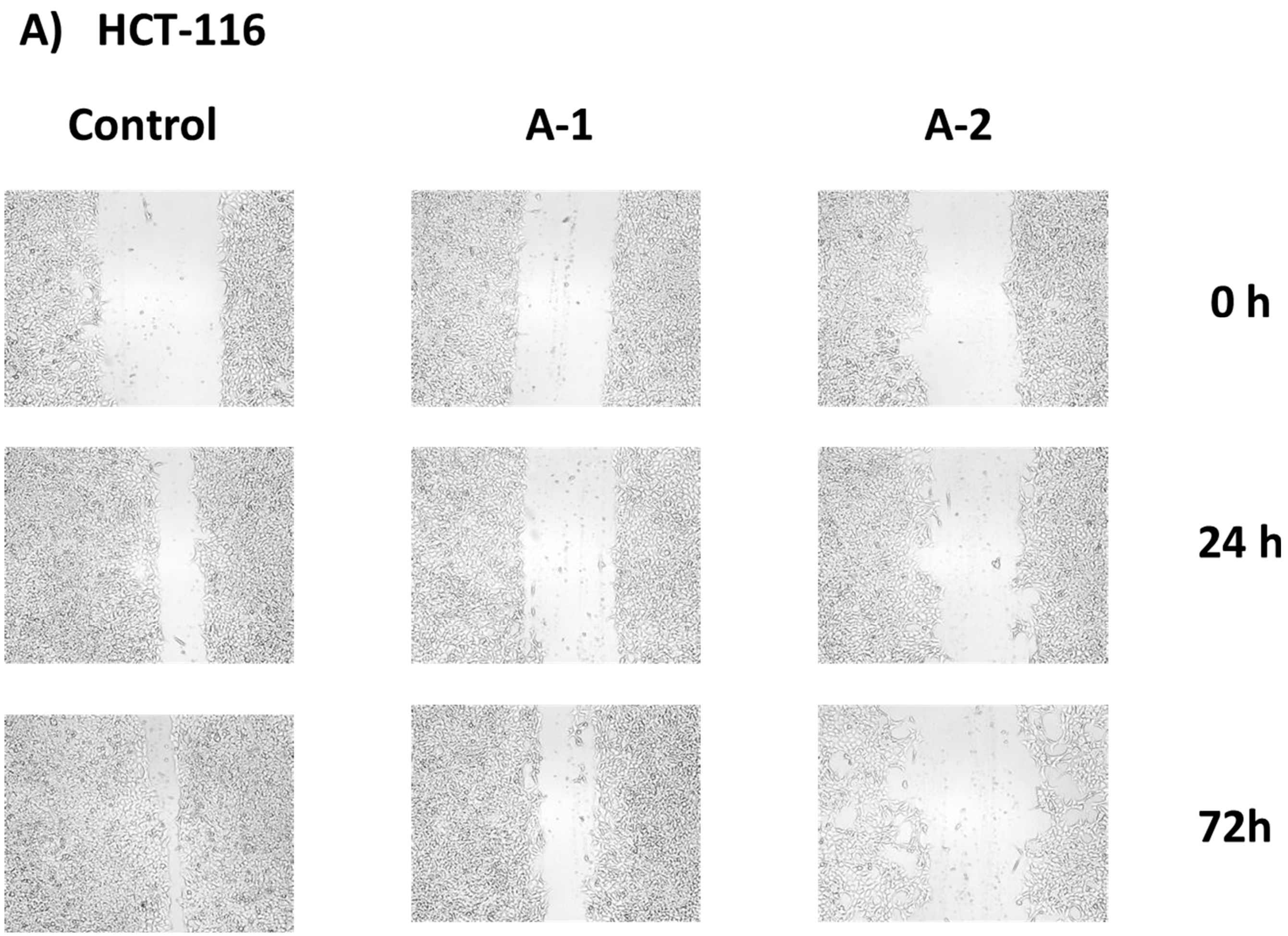
A) HCT-116
Control
A-1
A-2
0 h
24 h
72h

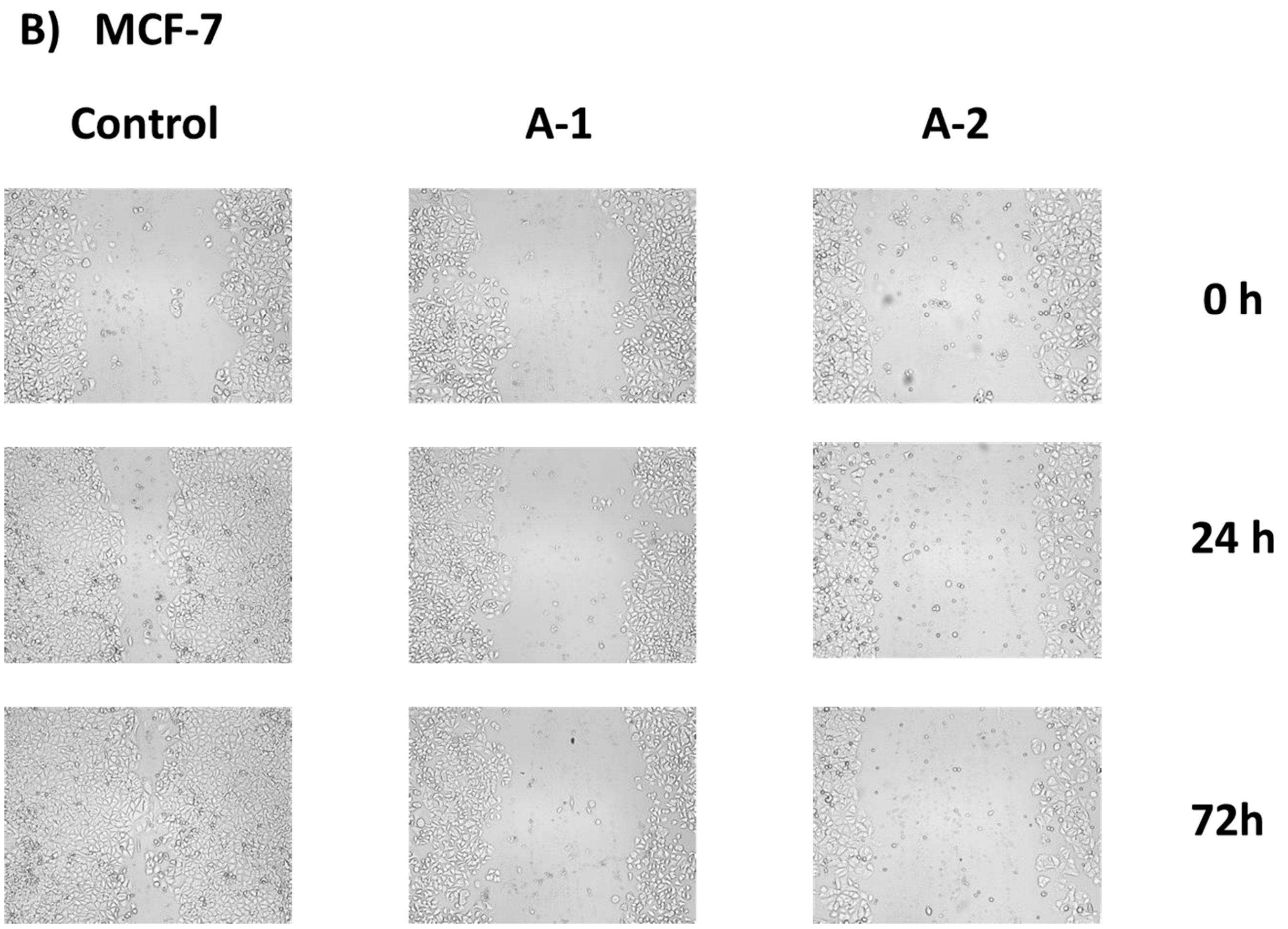
B) MCF-7
Control
A-1
A-2
0 h
24 h
72h

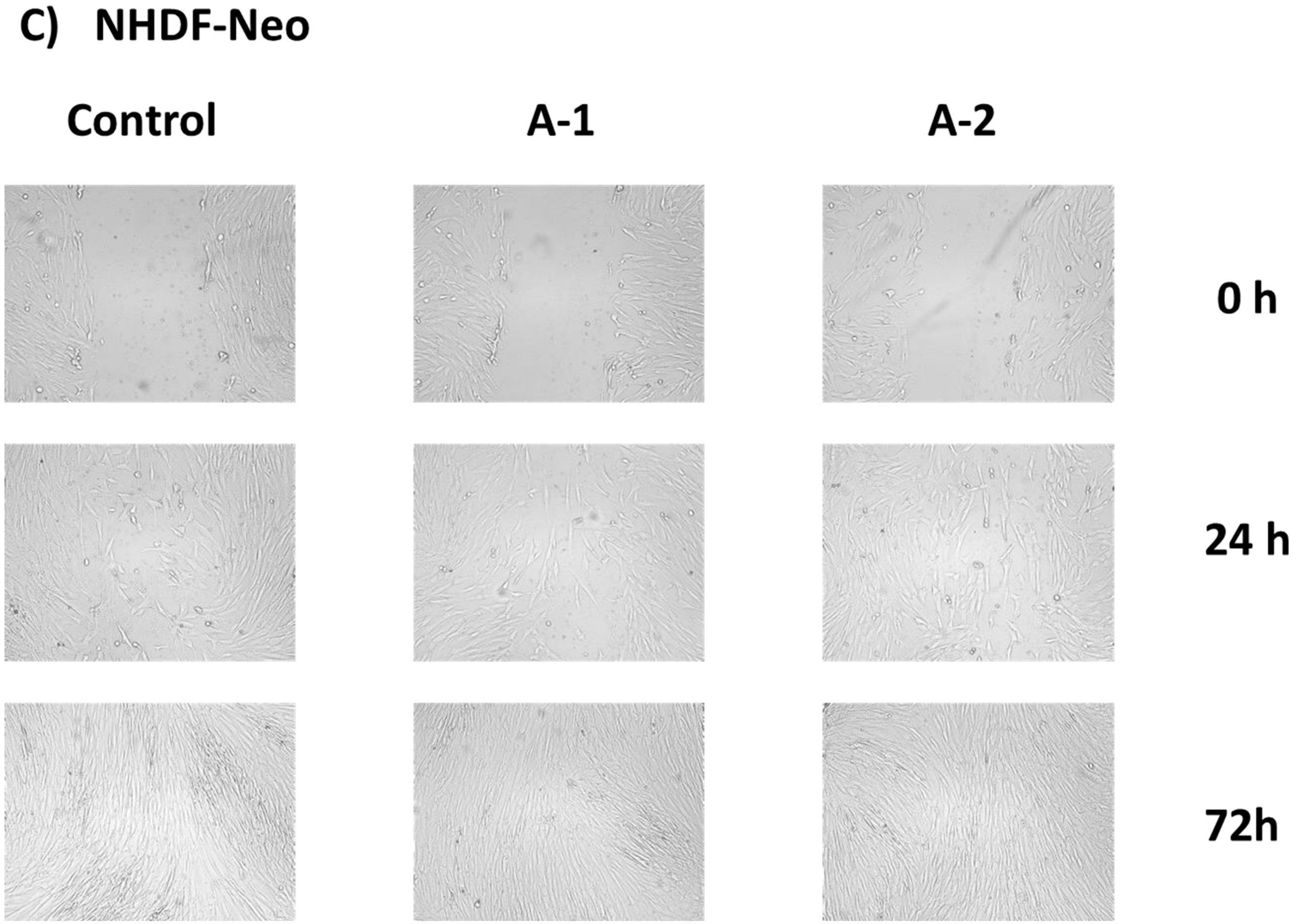

Fig. S75: Representative images of HCT-116 cancer cells (A), MCF-7 cancer cells (B), and NHDF-Neo healthy cells (C) were acquired at time 0 and after 24 h and 72 h incubation with compounds A-1 and A-2 in wound healing assay.
